# Association between sarcopenia-related traits and cardiovascular diseases: a bi-directional Mendelian randomization study

**DOI:** 10.3389/fendo.2023.1237971

**Published:** 2023-10-13

**Authors:** Xu Liu, Yunjie Wang, Zhaojun Wang, Lingzhi Li, Haibo Yang, Juncai Liu, Zhong Li

**Affiliations:** Department of Orthopaedics, The Affiliated Hospital of Southwest Medical University, Sichuan Provincial Laboratory of Orthopaedic Engineering, Luzhou, Sichuan, China

**Keywords:** Mendelian randomization, sarcopenia, appendicular lean mass, handgrip strength, cardiovascular diseases, causal effect

## Abstract

**Background:**

The two geriatric diseases, sarcopenia and cardiovascular disease (CVD), often coexist, yet the causal relationship is unclear. However, few studies focus on the effect of muscle mass on CVD. This comprehensive study is dedicated to unearthing the potential connection between sarcopenia-related traits and CVD at the genetic level.

**Method:**

A two-sample bi-directional Mendelian randomization (MR) study was conducted. In the first stage, we performed MR analysis regarding coronary heart disease (CHD), stroke, and myocardial infarction (MI) as exposure factors to reveal their effect on appendicular lean mass (ALM) and hand grip strength. In the second stage, we reverse the position of exposures and outcomes. The inverse variance weighted (IVW) method was used as the primary approach to reveal the potential causation between the exposure and outcome.

**Results:**

The results of the IVW method revealed a negative causal effect of ALM on CHD (OR = 0.848, 95% CI = 0.804 to 0.894, p = 8.200E-10), stroke (OR = 0.931, 95% CI = 0.890 to 0.975, p = 2.220E-03), and MI (OR = 0.810, 95% CI = 0.694 to 0.901, p = 1.266E-13). Additionally, the left-hand grip strength is a significant protective factor for CHD (OR = 0.737, 95% CI = 0.601 to 0.904, p = 3.353E-03) and MI (OR = 0.631, 95% CI = 0.515 to 0.765, p = 2.575E-06), but is not causally linked to the stroke (OR = 0.971, 95% CI =0.829 to 1.139, p = 0.720). Meanwhile, the same conclusion about the effect of right-hand grip strength on CHD (OR = 0.681, 95% CI = 0.558 to 0.832, p = 1.702E-05), MI (OR = 0.634, 95% CI = 0.518 to 0.776, p = 9.069E-06), and stroke (OR = 1.041, 95% CI = 0.896 to 1.209, p = 0.604) was obtained. However, no significant causal effect of CVD (CHD, stroke, MI) on sarcopenia-related traits (ALM, handgrip strength) was found.

**Conclusion:**

There is a unidirectional causal relationship between sarcopenia and CVD. The loss of muscle mass and strength has a significant causal role in promoting the occurrence and development of CVD, providing a reference for the prevention and treatment of comorbidities in older people.

## 1 Introduction

Sarcopenia, first named by Rosenberg in the late 1980s (1), has gradually become one of the most frequently stated health problems with the rapidly aging process of the population. With the deepening understanding of sarcopenia through clinical work, a consensus on the cognition of sarcopenia was reached. According to the European Working Group on Sarcopenia in Older People (EWGSOP), sarcopenia is defined as a systemic, geriatric syndrome of progressive loss of muscle mass and decreased strength (2), results in disastrous outcomes comprising falls, fractures, and decreased quality and function of life as one of the representatives of chronic diseases (3). Previous reports suggest that about 50 million people worldwide suffer from sarcopenia, and it is expected to reach 500 million people in 2050 (4, 5), leading to inestimable healthcare expenditures. Moreover, the latest systematic review comprising 130 studies summarized the epidemiological data, suggesting that sarcopenia was thought to affect 10%-16% of the older people worldwide, and compared with the general population, this proportion will be higher in people with other underlying diseases such as diabetes and cardiovascular disease (6).

Cardiovascular disease (CVD) is characterized by pathological changes in the circulatory system, including heart and blood vessels, and is the leading cause of death and disability worldwide (7). According to the multicenter survey, about 17.8 million people die annually from CVD, accounting for a third of the global death toll (8). As a primary form of CVD, coronary heart disease (CHD) has created a severe socio-economic burden according to the World Health Organization (9); at the same time, some acute cardiovascular disorders, such as stroke and myocardial infarction (MI), maintain a rising mortality rate (10). Furthermore, the incidence of CVD tends to be younger, so when faced with such inevitable challenges, a comprehensive understanding of CVD pathogenesis is urgently needed.

To our knowledge, extensive research has shown the common coexistence of sarcopenia and CVD (11, 12). A long-term follow-up study involving 15,000 samples showed that compared with the general population, the incidence of CVD in middle-aged and elderly with sarcopenia increased by 72%, and the risk of cardiovascular events increased by 33% (13). Likewise, a recent meta-analysis pooled the corresponding data and found a prevalence of sarcopenia in CVD patients ranging from 10.1% to 68.9% (14). Thus, exploring and clarifying the correlation between these two diseases is of positive significance. The current research on the correlation between sarcopenia and CVD is still exploratory, with no established strong consensus. A different or even opposite relationship between sarcopenia-related traits (muscle mass and handgrip strength) and CVD was obtained from these studies. The grip strength was found to be an independent predictor of CVD in a meta-analysis of community-based populations (12), while prospective research conducted by Gubelmann et al. (15) unexpectedly yielded a negative result. Among studies focusing on muscle mass and CVD, a cross-sectional study concluded that low muscle mass is an independent risk factor for CVD by statistically evaluating the relationship between coronary artery calcification score and skeletal muscle mass index (16). However, this association was reversed in postmenopausal women, according to another research (17). The limitation of the level of evidence and potential confounding factors like body fat mass led to this result, so these speculative conclusions make it hard to determine accurate causality between sarcopenia and CVD. Considering this, one approach to uncover the causality is to use Mendelian randomization (MR) analysis. According to the perspective of evidence-based medicine, MR analysis is second only to randomized controlled trials regarding evidence level.

Since the fundamental tenet adheres to Mendel’s second law, MR analysis is a rational method for determining causation that can validly counteract the confounding bias and reverse causality problems of observational epidemiological investigations. Single nucleotide polymorphisms (SNPs) associated with the exposure of interest were chosen as instrument variables (IVs), and credible causal inference between exposure factors and the outcome can be obtained using genetic variants (18–21). As far as we know, previous MR studies have concentrated on the association between grip strength and CVD rather than muscular mass (12–24). Moreover, no MR analysis has been published on the causality between sarcopenia and other CVDs except coronary artery disease. Therefore, we conducted a bi-directional two-sample MR study to unearth the causal genetic effect between the sarcopenia-related traits (muscle mass and handgrip strength) and CVD (CHD, stroke, and MI).

## 2 Materials and methods

### 2.1 Study design

**Figure 1:**
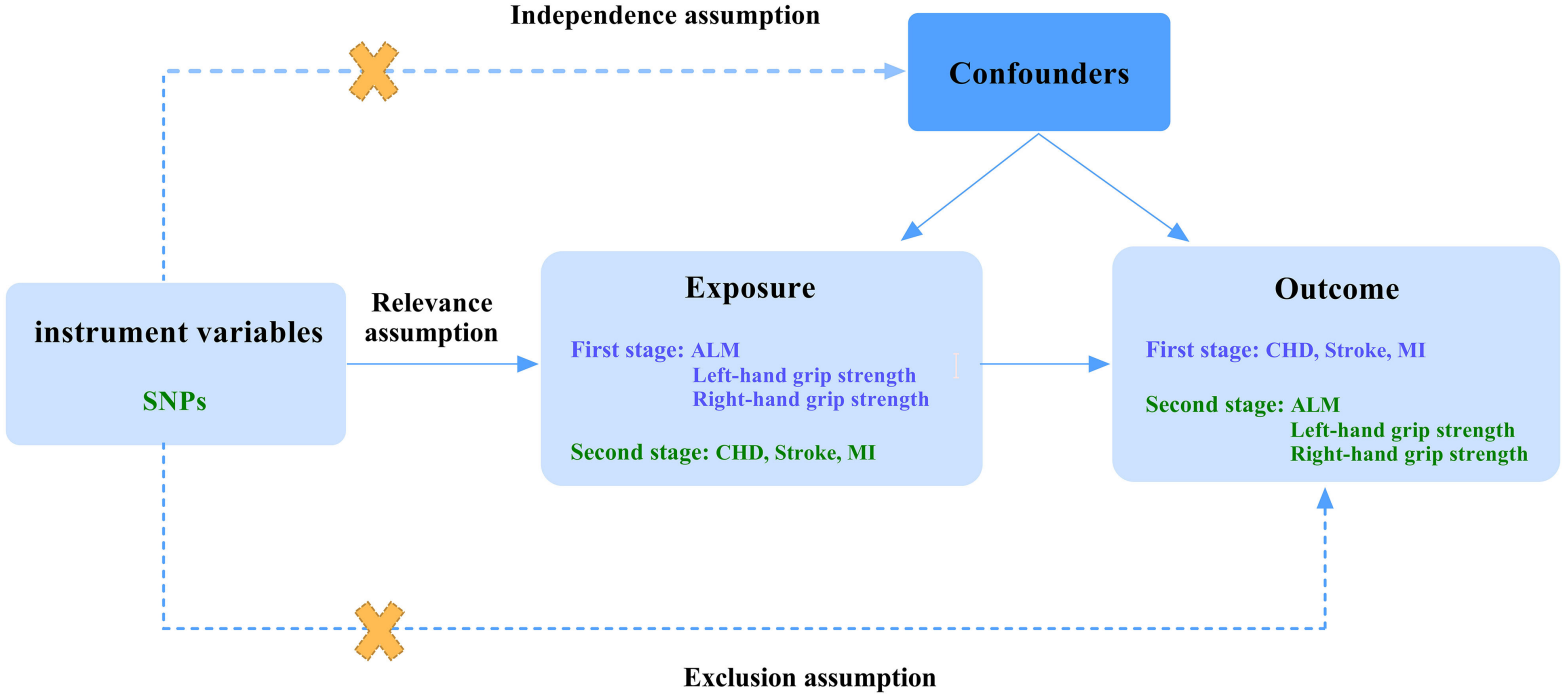
A flow diagram of study design and three basic assumptions of MR analysis. SNPs, single-nucleotide polymorphisms; ALM, appendicular lean mass; CHD, coronary heart disease; MI, myocardial infarction; First stage, a Mendelian analysis regarding CHD, stroke, and MI as the exposure and sarcopenia-related traits as the outcome; Second stage, the reverse Mendelian analysis regarding sarcopenia-related traits as the exposure and CHD, stroke, and MI as the outcome; Relevance assumption, the genetic instruments selected should be robustly associated with the exposure; Independence assumption, the genetic instruments are not associated with confounders; Exclusion assumption, the genetic instruments influence the outcome only through the exposure.

Since the loss of muscle mass and strength serve as the primary diagnostic criteria for sarcopenia, they are adopted to represent the disease measured as appendicular lean mass(ALM) and hand grip strength (5). We excluded walking pace to eliminate potential bias, as CVD directly affects it. We chose the common forms for CVD: CHD, stroke, and MI. In the first stage, we performed a two-sample Mendelian randomization study regarding CHD, stroke, and MI as exposure factors to reveal their effect on ALM and hand grip strength. In the second stage, we reverse the position of exposures and outcomes. Three assumptions below should be met when performing MR analysis: relevance assumption, the genetic instruments selected should be robustly associated with the exposure; independence assumption, the genetic instruments are not associated with confounders; exclusion assumption, the genetic instruments influence the outcome only through the exposure. The detailed process is shown in **Figure 1**.

### 2.2 Data sources

The data on the exposure and outcome in the bi-directional MR study came from the genome-wide association studies (GWAS) database. The ALM-related GWAS summary statistics analyzed by Pei et al. (25) were derived from the UK Biobank with 450,243 participants of European descent and 18,071,518 SNPs. Subsequently, the GWAS summary data of left-hand grip strength (461,089 European individuals, 9,851,867 SNPs) and right-hand grip strength (461,026 European individuals, 9,851,867 SNPs) was obtained through the UK Biobank (26). The CHD, Stoke, and MI data were collected only from European ancestry samples and mainly obtained from the Integrative Epidemiology Unit (IEU) GWAS database. The summary statistics for CHD comprised 184,305 individuals and 9,455,779 SNPs (27). Moreover, the GWAS summary data on stroke included 440,328 individuals and 7,537,579 SNPs (28). Simultaneously, the GWAS summary statistics for MI included 395,795 participants and 10,290,368 SNPs (29). All the raw data are available in the original studies. The datasets we adopted were freely available to the public, and each GWAS engaged had ethical approval from their respective institutions. Under the premise of ensuring GWAS data quality, the data on sarcopenia-related traits and cardiovascular disease came from different databases and consortiums to avoid sample overlap bias in the study results as much as possible. **Table 1** shows the details of GWAS datasets used for MR analysis.

**Table 1:** The GWAS datasets used for MR analysis.

	Traits	No. of SNPs	Sample size	Population	Main consortium
Sarcopenia-related traits	ALM	18,071,518	450,243	European	UK Biobank (Pei YF, et al./PMID:33097823)
	Left-hand grip strength	9,851,867	461,026	European	UK Biobank (https://doi.org/10.5523/bris.pnoat8cxo0u52p6ynfaekeigi)
	Right-hand grip strength	9,851,867	461,089	European	UK Biobank (https://doi.org/10.5523/bris.pnoat8cxo0u52p6ynfaekeigi)
Cardiovascular disease	CHD	9,455,779	184,305	European	CARDIoGRAMplusC4D (Nikpay, et al./PMID:26343387)
	Stroke	7,537,579	440,328	European	CARDIoGRAMplusC4D (Malik, et al./PMID:29531354)
	MI	10,290,368	395,795	European	CARDIoGRAMplusC4D (Hartiala, et al./PMID:33532862)

MR, Mendelian randomization; ALM, appendicular lean mass; CHD, coronary heart disease; MI, myocardial infarction; CARDIoGRAMplusC4D, Coronary ARtery DIsease Genome wide Replication and Meta-analysis plus The Coronary Artery Disease Genetics.

### 2.3 Selection of genetic instrumental variables

All the SNPs selected as instrumental variables in the MR study should obey the three basic assumptions. Firstly, we extracted the independent SNPs without linkage disequilibrium (r2< 0.001) and strongly (*p*< 5 × 10−8) related to the exposure to satisfy the relevance assumption. Next, we removed the SNPs for being palindromic with intermediate allele frequencies (**Supplementary Table 1**). The proxy SNPs were not used in this MR analysis. Under the independence and exclusion assumptions, IVs associated with the outcome and potential confounders (body fat-related traits, smoking, heart rate) (**Supplementary Table 2**) were manually excluded by PhenoScanner (http://www.phenoscanner.medschl.cam.ac.uk).

Additionally, we conducted a pleiotropy test to ensure the effect of horizontal pleiotropy by MR-Egger. If the intercept term is statistically significant, we performed MR-PRESSO (Pleiotropy Residual Sum and Outlier) to remove the abnormal SNPs (**Supplementary Table 3**) and then back to the pleiotropy test again. Detailed information on intercept and *p*-value is shown in **Supplementary Table 4**. Finally, we calculated the F statistic of each SNP to assess the strength through the following formula: $F=R^{2}\left(n-k-1\right)\;÷\left[k\left(1-R^{2}\right)\right]$ , and the *F* value greater than 10 was considered to be of sufficient power to eliminate bias (30).

### 2.4 MR analysis

The inverse variance weighted (IVW) method was used as the primary approach to reveal the potential causation between the exposure and outcome since it was deemed the most reliable method if there was no indication of pleiotropy (31). Subsequently, the results of heterogeneity evaluated by Cochran’s Q analysis were used to determine the models of IVW. We conduct the fixed-effect model IVW if the *p*-value is greater than 0.05; otherwise, the random-effects model IVW is more suitable (32). The weighted median and MR-Egger were also used as complementary approaches to IVW. The weighted median can yield an effective causal assessment when at least 50% of the selected IVs are valid (33). The MR-Egger method was used more on the pleiotropy than the significant causal effect because of its weak statistical power (34).

### 2.5 Sensitivity analysis

In the section on sensitivity analysis, we used Cochran’s Q test to assess the heterogeneity, while the MR-Egger intercept was used to test the pleiotropy. The p-value of Cochran’s Q statistic was used to determine the effect models of IVW. When there is significant pleiotropy (p< 0.05), the MR-PRESSO and “leave-one-out” (**Supplementary Table 5**) tests were performed to remove the outliers that could affect the causal effect evaluation. After removal, we repeated Cochran’s Q test, MR-Egger intercept, and MR analysis until there was no indication of pleiotropy. The final detailed values of heterogeneity and pleiotropy tests are shown in **Supplementary Table 4**.

Both the MR and sensitivity analysis were performed with the R package of “TwoSampleMR” and “MR-PRESSO” in the R statistical software (version 4.2.1).

## 3 Results

### 3.1 Selected instrumental variables

In the first stage, we performed an MR analysis regarding the sarcopenia-related traits (ALM, left and right-hand grip strength) as exposure and CVD (CHD, stroke, MI) as the outcome. In the second stage, we reverse the direction of MR analysis. Subsequently, after an adequate selection process, we obtained the SNPs strongly associated with sarcopenia-related traits and CVD, and the *F*-value of each instrumental variable is greater than 10. We list all the detailed information on SNPs used in this study in **Supplementary Tables 6–23**.

### 3.2 First stage: causal effect of sarcopenia-related traits on CVD

We reported the MR results in the form of odds ratios (OR) with a 95% confidence interval (CI). As shown in **Table 2**, a compelling protective causality exists between the ALM and CHD (IVW OR = 0.848, 95% CI = 0.804 to 0.894, *p* = 8.200E-10), stroke (IVW OR = 0.931, 95% CI = 0.890 to 0.975, *p* = 2.220E-03), and MI (IVW OR = 0.810, 95% CI = 0.694 to 0.901, *p* = 1.266E-13). The results of the weighted median and MR Egger showed a roughly consistent trend.

**Table 2:** The causal association of sarcopenia-related traits on CVD by MR analysis results.

Exposure	Outcome	No.IVs	Method	OR (95% CI)	*p*-value
ALM	CHD	569	IVW	0.848 (0.804, 0.894)	**8.200E-10**
			Weighted median	0.832 (0.772, 0.898)	**1.792E-06**
			MR Egger	0.764(0.676,0.864)	**1.980E-05**
ALM	Stroke	564	IVW	0.931 (0.890, 0.975)	**2.220E-03**
			Weighted median	0.960 (0.897, 1.025)	0.217
			MR Egger	0.939 (0.839, 1.050)	0.270
ALM	MI	580	IVW	0.810 (0.694, 0.901)	**1.266E-13**
			Weighted median	0.822 (0.760, 0.890)	**1.028E-06**
			MR Egger	0.790 (0.707, 0.914)	**4.469E-04**
Left-hand grip strength	CHD	139	IVW	0.737 (0.601, 0.904)	**3.353E-03**
			Weighted median	0.763 (0.593, 0.983)	**0.036**
			MR Egger	0.495 (0.219, 1.119)	0.093
Left-hand grip strength	Stroke	132	IVW	0.971 (0.829, 1.139)	0.720
			Weighted median	0.925 (0.739, 1.157)	0.487
			MR Egger	1.299 (0.676, 2.495)	0.433
Left-hand grip strength	MI	139	IVW	0.631 (0.521, 0.765)	**2.575E-06**
			Weighted median	0.642 (0.495, 0.833)	**8.658E-04**
			MR Egger	0.632 (0.304, 1.312)	**2.204E-04**
Right-hand grip strength	CHD	150	IVW	0.681 (0.558, 0.832)	**1.702E-05**
			Weighted median	0.706 (0.550, 0.907)	**6.377E-03**
			MR Egger	0.544 (0.250, 1.183)	0.127
Right-hand grip strength	Stroke	157	IVW	1.041 (0.896, 1.209)	0.604
			Weighted median	1.019 (0.824, 1.262)	0.858
			MR Egger	1.177 (0.630, 2.198)	0.609
Right-hand grip strength	MI	157	IVW	0.634 (0.518, 0.776)	**9.069E-06**
			Weighted median	0.519 (0.407, 0.661)	**1.072E-07**
			MR Egger	0.577 (0.272, 1.222)	0.153

MR, Mendelian randomization; ALM, appendicular lean mass; CHD, coronary heart disease; IVs, instrument variables; IVW, inverse variance weighted; MI, myocardial infarction; OR, odds ratios; CI, confidence interval; Values in bold represent statistical significance.

According to the MR results (**Table 2**), the left-hand grip strength is a significant protective factor for CHD (IVW OR = 0.737, 95% CI = 0.601 to 0.904, *p* = 3.353E-03) and MI (IVW OR = 0.631, 95% CI = 0.515 to 0.765, *p* = 2.575E-06), but is not causally linked to the stroke (IVW OR = 0.971, 95% CI =0.829 to 1.139, *p* = 0.720). Meanwhile, The MR results listed in **Table 1** illustrated the same conclusion about the effect of right-hand grip strength on CHD (IVW OR = 0.681, 95% CI = 0.558 to 0.832, *p* = 1.702E-05), MI (IVW OR = 0.634, 95% CI = 0.518 to 0.776, *p* = 9.069E-06), and stroke (IVW OR = 1.041, 95% CI = 0.896 to 1.209, *p* = 0.604). Moreover, the IVW, weighted median, and MR Egger results all suggested no causal effect of the hand grip strength on stroke.

**Figure 2:**
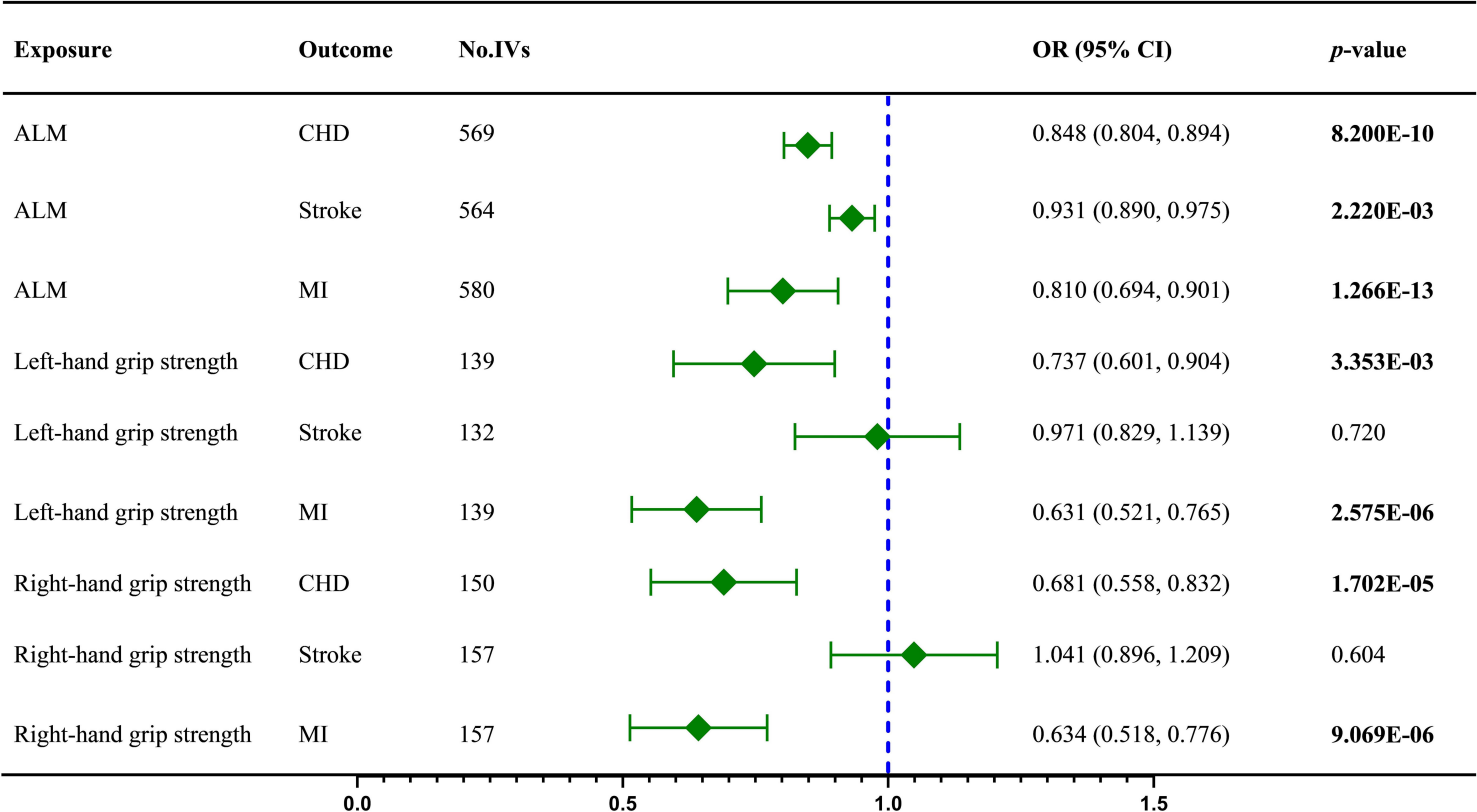
The IVW results regarding the causal effect of sarcopenia-related traits on CVD; ALM, appendicular lean mass; CHD, coronary heart disease; IVs, instrument variables; IVW, inverse variance weighted; MI, myocardial infarction; OR, odds ratios; CI, confidence interval; Values in bold represent statistical significance.

We performed the fixed-effect model IVW for the causal effect of left-hand grip strength on stroke (*Q* = 144.098, *P* = 0.188) and MI (*Q* = 163.913, *P* = 0.058) by Cochran’s Q test results. No significant horizontal pleiotropy was found by the MR-Egger intercept test (**Supplementary Table 4**). The abnormal SNPs removed by MR-PRESSO and “leave-one-out” tests are shown in **Supplementary Table 5**. The funnel plot and “leave-one-out” test diagrams are also provided in the **Supplementary Figures**. The visualized causal association results between sarcopenia-related traits and CVD are shown in **Figure 2**.

### 3.3 Second stage: causal effect of CVD on sarcopenia-related traits

In this stage, we reverse the position of the exposure and outcome. Firstly, the IVW results indicated no dramatic influence of CHD on ALM (IVW OR = 1.008, 95% CI = 0.994 to 1.023, *p* = 0.257), left-hand grip strength (IVW OR = 0.998, 95% CI = 0.991 to 1.005, *p* = 0.520), and right-hand grip strength (IVW OR = 0.997, 95% CI = 0.990 to 1.004, *p* = 0.430), subsequently weighted median and MR Egger method confirmed the results (**Table 2**). Then there was no statistically significant effect of stroke on ALM (IVW OR = 0.977, 95% CI = 0.952 to 1.003, *p* = 0.086), left-hand grip strength (IVW OR = 0.994, 95% CI = 0.973 to 1.015, *p* = 0.558), and right-hand grip strength (IVW OR = 0.994, 95% CI = 0.973 to 1.016, *p* = 0.515). Further analysis by other methods obtained similar results (**Table 3**). No special effect was found for MI as a risk factor and right-hand grip strength as an outcome too, and the results are as follows: ALM (IVW OR = 0.995, 95% CI = 0.972 to 1.019, *p* = 0.693); left-hand grip strength (IVW OR = 0.998, 95% CI =0.989 to 1.007, *p* = 0.659); right-hand grip strength (IVW OR = 1.006, 95% CI = 0.998 to 1.014, *p* = 0.195)(**Table 2**).

**Table 3:** The causal association of CVD on sarcopenia-related traits by MR analysis results.

Exposure	Outcome	No.IVs	Method	OR (95% CI)	*p*-value
CHD	ALM	22	IVW	1.008 (0.994, 1.023)	0.257
			Weighted median	1.008 (0.992, 1.024)	0.323
			MR Egger	1.009 (0.977, 1.041)	0.605
CHD	Left-hand grip strength	28	IVW	0.998 (0.991, 1.005)	0.52
			Weighted median	0.992 (0.981, 1.003)	0.167
			MR Egger	0.984(0.968, 1.003)	0.105
CHD	Right-hand grip strength	28	IVW	0.997 (0.990, 1.004)	0.43
			Weighted median	0.996 (0.985, 1.008)	0.514
			MR Egger	0.991(0.973, 1.010)	0.371
Stroke	ALM	7	IVW	0.977 (0.952, 1.003)	0.086
			Weighted median	0.982 (0.944, 1.022)	0.382
			MR Egger	0.995(0.796, 1.243)	0.965
Stroke	Left-hand grip strength	8	IVW	0.994 (0.973, 1.015)	0.558
			Weighted median	0.992 (0.966, 1.019)	0.566
			MR Egger	0.958 (0.862, 1.065)	0.46
Stroke	Right-hand grip strength	7	IVW	0.994 (0.973, 1.016)	0.615
			Weighted median	0.994 (0.969, 1.020)	0.677
			MR Egger	0.961 (0.860, 1.074)	0.515
MI	ALM	20	IVW	0.995 (0.972, 1.019)	0.693
			Weighted median	0.999 (0.981, 1.018)	0.958
			MR Egger	1.001 (0.951, 1.053)	0.979
MI	Left-hand grip strength	24	IVW	0.998 (0.989, 1.007)	0.659
			Weighted median	0.992(0.981, 1.002)	0.118
			MR Egger	0.985(0.970, 1.001)	0.087
MI	Right-hand grip strength	20	IVW	1.006 (0.998, 1.014)	0.195
			Weighted median	1.001 (0.990, 1.012)	0.865
			MR Egger	0.997 (0.979, 1.016)	0.772

MR, Mendelian randomization; ALM, appendicular lean mass; CHD, coronary heart disease; IVs, instrument variables; IVW, inverse variance weighted; MI, myocardial infarction; OR, odds ratios; CI, confidence interval.

**Figure 3:**
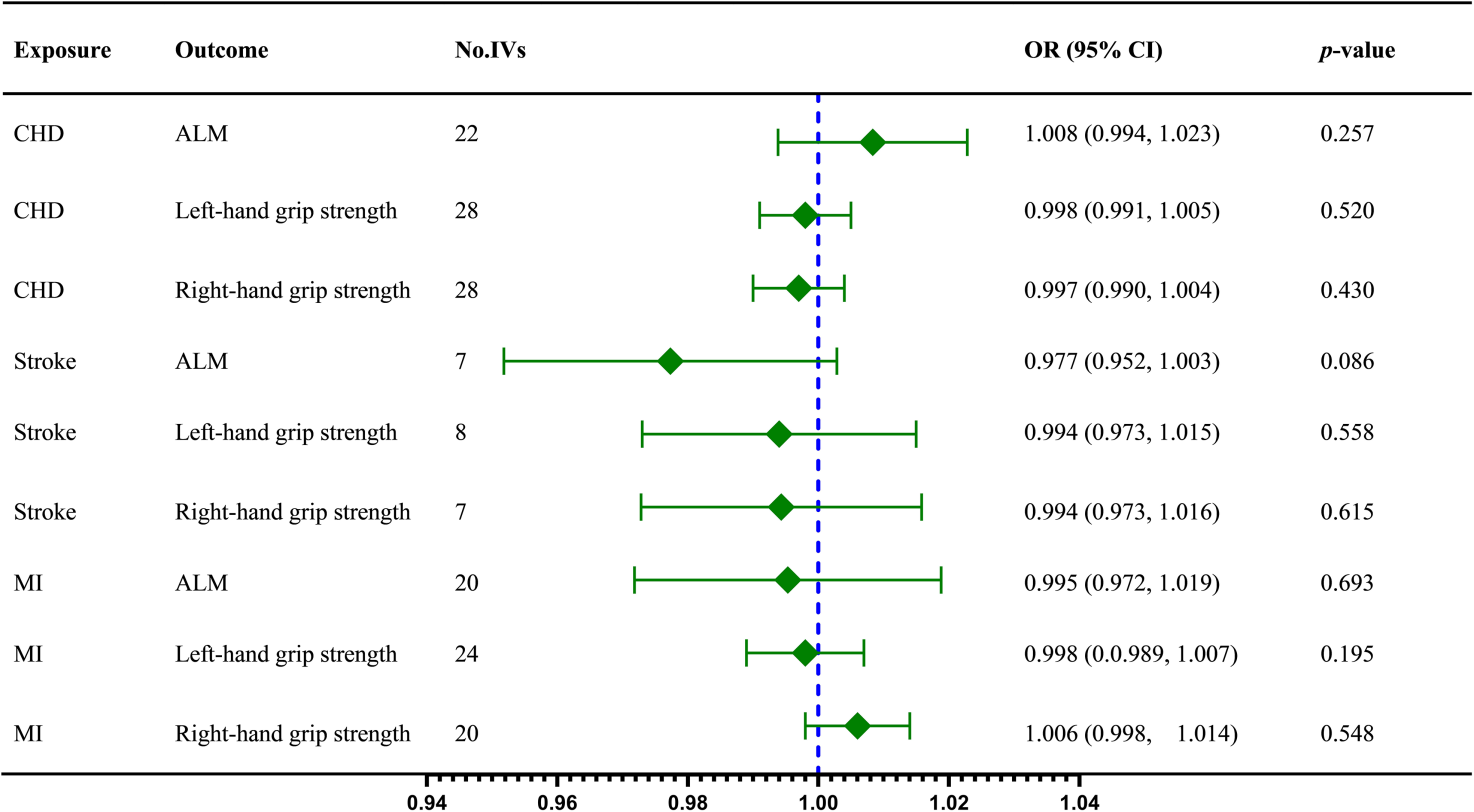
The IVW results regarding the causal effect of CVD on sarcopenia-related traits; ALM, appendicular lean mass; CHD, coronary heart disease; IVs, instrument variables; IVW, inverse variance weighted; MI, myocardial infarction; OR, odds ratios; CI, confidence interval;.

The fixed-effect model IVW was adopted for terms without remarkable heterogeneity, including CHD on left-hand grip strength (*Q* = 36.488, *P* = 0.083), stroke on ALM (*Q* = 9.417, *P* = 0.094), stroke on left-hand grip strength (*Q* =2.814, *P* = 0.832), stroke on right-hand grip strength (*Q* = 1.308, *P* = 0.9684), MI on left-hand grip strength (*Q* =31.154, *P* = 0.093), and MI on right-hand grip strength (*Q* = 19.429, *P* = 0.365). The MR-Egger intercept shows no evidence of horizontal pleiotropy (P > 0.05). All the sensitivity analysis results can be found in **Supplementary Materials**. The visualized causal effect value of CVD on sarcopenia-related traits is shown in **Figure 3**.

## 4 Discussion

This exhaustive MR study aimed to assess the bilateral causality between the sarcopenia-related traits (ALM and handgrip strength) and CVD (CHD, stroke, and MI). The first stage of the MR analysis revealed that increased ALM could reduce the risk of CHD, stroke, and MI, while excellent left or right handgrip strength has been indicated to be a protective factor against CHD and MI. Additionally, no compelling causal effect of CVD (CHD, stroke, and MI) on ALM and left or right handgrip strength was found in the second stage.

Characterized by a persistent loss of muscle mass and strength, sarcopenia leads to the aging and functional decline of the locomotor system. Consequently, physical activity restriction increases the risk of CVD in older patients. Owing to the possible existence of many common pathogeneses, including inflammation, insulin resistance, and oxidative stress (35), sarcopenia and CVD always coexist, interact, and form a vicious circle in the disease process (10, 11).

Among the many similar pathogenetic pathways of sarcopenia and CVD, sarcopenic obesity (SO) is regarded as a core mechanism by which sarcopenia affects CVD (36). The occurrence of sarcopenia usually results in a striking decrease in the resting metabolic rate of the body, a decline in physical activity, and excess dietary energy, thereby causing fat accumulation, especially the gathering of abdominal visceral fat. Whereafter, the chronic inflammation induced by the accumulated fat further accelerates the development of sarcopenia and CVD (37). However, much uncertainty still exists about the relationship between SO and CVD since most current related studies are cross-sectional surveys rather than prospective research. Not only that, the heterogeneity in the definition and classification of SO contributes to the discrepancy in the few prospective studies. In a longitudinal study reported by Stephen (34), the SO assessed by waist circumference and muscle strength was associated with a high risk of CVDs. Surprisingly, no remarkable relationship was found according to a similar study by Atkins et al. (38) with a definition of SO using the circumference of the waist and arm muscles. Noteworthy, a cross-sectional study reported in 2015 by Kim et al. (39) suggests that while the SO was strongly associated with increased CVD risk, no higher CVD risk was observed in the participants with only obesity but no loss of muscle mass. The rather intriguing result further supports the substantial independent effect of muscle mass on CVD, and previous research has established that the loss of muscle mass is an independent risk factor and prognostic predictor for CVD (39, 40).

Nevertheless, confounding factors related to body fat are still inevitable, and the inconsistency of former studies could be attributed to it (41, 42). MR analysis is an ideal method to reveal the potential causal relationship and direction between these two chronic geriatric diseases at the genetic level to minimize bias. Up to now, only one MR study has attempted to investigate the causality between muscle mass and coronary artery disease, and no significant causal relationship exists, according to the study (43). Regrettably, as the author mentioned in the limitation of the research, the body lean mass rather than ALM was adopted to assess the muscle mass condition. Considering the possible effects of visceral fat, ALM is more appropriate and accurate (1). In our study, we use the ALM as a measure of muscle mass, which may partly explain the inconsistent result. Notably, we carefully removed the potential confounders (body fat-related traits, smoking, heart rate) by PhenoScanner. The compelling causal effect of ALM on CHD also corroborates the findings of previous work (17). Additionally, as well as we know, this is the first MR study exploring the causal effect of ALM on stroke and MI.

For the causal role of left and right handgrip strength on CHD and MI, the findings align with those of previous MR studies (22, 23, 43). Besides, a series of cross-sectional studies and meta-analyses have also highlighted the negative effect of low handgrip strength on CVD (12, 15, 44). However, we have not identified an apparent cause role of muscle strength on stroke. Since past studies focused more on low grip strength as a prognostic risk factor for functional recovery after stroke (45), there is abundant room for further progress in determining the relationship between muscle strength and stroke through some high-quality studies.

In this study, we determined the causal influence of muscle mass and strength on MI by MR analysis for the first time, and a coincident result was obtained regarding CHD as the outcome. Following the latest universal definition of MI (46), coronary artery disease plays a crucial part in the pathogenesis of MI. Therefore, the consistent trend in the results of CHD and MI also increases the credibility of the conclusion. However, no significant causal effect of CVD on sarcopenia was found. Despite multiple overlaps in pathogenesis, this study suggests that sarcopenia often precedes CVD and serves as a risk factor for the progression of CVD. The underlying reason can be explained by the fact that loss of muscle mass and strength is the beginning of many pathological mechanisms. Insulin resistance sensitizes the vascular smooth muscle cells through high levels of insulin, interferes with the control of blood pressure by the renin-angiotensin-aldosterone system, increases the resistance of vascular smooth muscle, and leads to hypertension and myocardial remodeling (47). At the same time, as the main tissue of insulin-mediated glucose metabolism, the skeletal muscle has a significant inhibitory effect on insulin resistance, and the loss of muscle mass and strength is often the onset of insulin resistance (48). Skeletal muscles always produce free radicals, and age-related decline in muscle strength contributes to excess free radicals and oxidative stress, causing vascular endothelial dysfunction, which plays a vital role in the pathogenesis of atherosclerosis and CVD (49). Furthermore, sarcopenia can affect the myocardium directly since it is also striated muscle like skeletal muscle, and similar pathophysiological mechanisms result in the atrophy and apoptosis of myocardial fibers and reduction and dysfunction of organelles (50).

Our study explored the causal relationship between sarcopenia-related traits (ALM, left and right-hand grip strength) and CVD (CHD, stroke, MI) through MR analysis for the first time with inspiring results. Compared with traditional observational studies, MR study dramatically reduces the bias caused by confounding factors, and based on previous similar MR studies, this study is more comprehensive and rigorous in the selection of interesting traits. Nevertheless, as MR analysis is supplementary research to clinical studies, the generalisability of these results is also subject to certain limitations. For instance, despite all the genetic tools selected from European ancestry individuals, differences between different groups, such as age, gender, living environment, and education level, are unavoidable, and these uncertainties may bias the results. Secondly, the results of the MR Egger methods were not completely compatible with the IVW method. This may be attributed to its statistical characteristics, including insufficient statistical power, susceptibility to the influence of outlying variants, and larger standard deviations than other methods. Still, the IVW method is prioritized when no pleiotropy and heterogeneity exist. Regrettably, we did not repeat the analysis using the identical phenotypes obtained from other databases, which could make our findings more credible. Furthermore, limited by the current research status, some unreported confounding factors cannot be eliminated, and these unknown confounders could make the results different. Therefore, further randomized controlled trials on more co-factors for sarcopenia and CVD are recommended.

## 5 Conclusions

There is a unidirectional causal relationship between sarcopenia and CVD. The genetic evidence from this Mendelian randomization analysis suggests that the loss of muscle mass and strength has a significant causal role in promoting the occurrence and development of CVD, providing a reference for the prevention and treatment of comorbidities in older people.

## Data availability statement

The datasets presented in this study can be found in online repositories. The names of the repository/repositories and accession number(s) can be found in the article/**Supplementary Material**.

## Author contributions

XL, ZL and JL designed the study. XL drafted the article. YW and ZW performed data analysis. LL and HY revised the manuscript. All authors contributed to the article and approved the submitted version.
