# Supplementary material for: Association between sarcopenia-related traits and cardiovascular diseases: a bi-directional Mendelian randomization study
Source: Front Endocrinol (Lausanne). 2023 Oct 13;14:1237971. doi: 10.3389/fendo.2023.1237971 (PMC10613058; doi:10.3389/fendo.2023.1237971)
Supplement: Supplementary file 1 [file DataSheet_1.zip › Supplementary Tables.DOCX]

Supplementary Material

**[Association](https://pubmed.ncbi.nlm.nih.gov/36902778/) between sarcopenia-related traits and cardiovascular diseases: A bi-directional Mendelian randomization study**

Xu Liu^1†^, Yunjie Wang^1†^, Zhaojun Wang^1†^, Lingzhi Li^1^, Haibo Yang^1^, Juncai Liu^1^*** and** Zhong Li^1^*****

*** Correspondence:** Zhong Li: [lizhong170209@163.com](mailto:lizhong170209@163.com)

**Supplementary Tables**

**Supplementary Table 1.** SNPs removed for being palindromic with intermediate allele frequencies

**Supplementary Table 2.** The removed potential confounder-related SNPs

**Supplementary Table 3.** The removed abnormal SNPs in MR-PRESSO analysis

**Supplementary Table 4.** Heterogeneity and pleiotropy tests between exposure factors and outcome variables.

**Supplementary Table 5.** The removed abnormal SNPs in the one-and-leave method

**Supplementary Table 6.** Characteristics of selected SNPs concerning the causal effect of ALM on CHD

**Supplementary Table 7.** Characteristics of selected SNPs concerning the causal effect of ALM on stroke

**Supplementary Table 8.** Characteristics of selected SNPs concerning the causal effect of ALM on MI

**Supplementary Table 9.** Characteristics of selected SNPs concerning the causal effect of left-hand grip strength on CHD

**Supplementary Table 10.** Characteristics of selected SNPs concerning the causal effect of left-hand grip strength on stroke

**Supplementary Table 11.** Characteristics of selected SNPs concerning the causal effect of left-hand grip strength on MI

**Supplementary Table 12.** Characteristics of selected SNPs concerning the causal effect of right-hand grip strength on CHD

**Supplementary Table 13.** Characteristics of selected SNPs concerning the causal effect of right-hand grip strength on stroke

**Supplementary Table 14.** Characteristics of selected SNPs concerning the causal effect of right-hand grip strength on MI

**Supplementary Table 15.** Characteristics of selected SNPs concerning the causal effect of CHD on ALM

**Supplementary Table 16.** Characteristics of selected SNPs concerning the causal effect of CHD on left-hand grip strength

**Supplementary Table 17.** Characteristics of selected SNPs concerning the causal effect of CHD on right-hand grip strength

**Supplementary Table 18.** Characteristics of selected SNPs concerning the causal effect of stroke on ALM

**Supplementary Table 19.** Characteristics of selected SNPs concerning the causal effect of stroke on left-hand grip strength

**Supplementary Table 20.** Characteristics of selected SNPs concerning the causal effect of stroke on right-hand grip strength

**Supplementary Table 21.** Characteristics of selected SNPs concerning the causal effect of MI on ALM

**Supplementary Table 22.** Characteristics of selected SNPs concerning the causal effect of MI on left-hand grip strength

**Supplementary Table 23.** Characteristics of selected SNPs concerning the causal effect of MI on right-hand grip strength

**Supplementary Table 24.** The lost SNPs after merging outcome and exposure.

**Supplementary Table 1.** SNPs removed for being palindromic with intermediate allele frequencies

| **Exposures** | **Outcomes** | **The removed SNPs** |
| --- | --- | --- |
| ALM | CHD | rs10036789, rs10203386, rs10657263, rs11233117, rs11562101, s12334478, rs2174008, rs3901421, rs4360494, rs7137546, rs7768973, rs7863102, rs7971536, rs861674, rs9343327, rs9640283, rs985136 |
|  | Stroke | rs10036789, rs10203386, rs10657263, rs10962212, rs11233117, rs11562101, rs12334478, rs2174008, rs3901421, rs4360494, rs7137546, rs7768973, rs7863102, rs7971536, rs861674, rs9343327, rs9640283, rs985136 |
|  | MI | rs10036789, rs10203386, rs10657263, rs11233117, rs11562101, rs12334478, rs2174008, rs3901421, rs4360494, rs7137546, rs7768973, rs7863102, rs7971536, rs861674, rs9343327, rs9640283, rs985136 |
| Left-hand grip strength | CHD | rs35054365, rs3959716, rs997850 |
|  | Stroke | rs10788958, rs35054365, rs3959716, rs997850 |
|  | MI | rs35054365, rs3959716 |
| Right-hand grip strength | CHD | rs10798876, rs1440152, rs1635527, rs6063504, rs7652177, rs7953280,rs9639938, rs997850 |
|  | Stroke | rs10798876, rs1440152, rs1635527, rs6063504, rs7652177, rs7953280, rs9639938, rs997850 |
|  | MI | rs10798876, rs1440152, rs1635527, rs6063504, rs7652177, rs7953280, rs9639938 |
| CHD | ALM | rs10139550, rs7568458 |
|  | Left-hand grip strength | rs10139550, rs7568458 |
|  | Right-hand grip strength | rs10139550, rs7568458 |
| Stroke | ALM | NA |
|  | Left-hand grip strength | NA |
|  | Right-hand grip strength | NA |
| MI | ALM | NA |
|  | Left-hand grip strength | NA |
|  | Right-hand grip strength | NA |

ALM, appendicular lean mass; CHD, coronary heart disease; MI, myocardial infarction;

**Supplementary Table 2.** The removed potential confounder-related SNPs

| **Exposures** | **Outcomes** | **The removed SNPs** |
| --- | --- | --- |
| ALM | CHD | rs10807137, rs1291114, rs2287821, rs2764264, rs28678024, rs34517439, rs3764002, rs41271299, rs4985445, rs591668, rs59985551, rs7107356, rs9391254,rs963317, rs42039，rs200739311，rs201570119 |
|  | Stroke | rs10807137, rs1291114, rs2287821, rs2764264, rs7971536, rs861674, rs591668, rs76364830, rs28678024, rs34517439, rs3764002, rs41271299, rs4985445, rs59985551, rs7107356, rs9391254, rs963317 |
|  | MI | rs3184504, rs10807137, rs2287821, rs2764264, rs28678024, rs42039, rs4985445, rs59985551, rs7107356, rs9391254, rs963317rs1291114, rs34517439, rs41271299, rs76895963, rs591668, rs3764002,rs59985551,rs42039 |
| Left-hand grip strength | CHD | rs11642954, rs12889267, rs13107325, rs2431112, rs7196917,rs2850379,rs35175534, rs56060323 |
|  | Stroke | rs10205394, rs1044299, rs10786706, rs11002322 rs13091492, rs13107325, rs1981612, rs2431112, rs3814877, rs4335354, rs7124681, rs7196917 |
|  | MI | rs10205394, rs12889267, rs1981612,rs13091492,rs11002322, rs7124681, rs7196917, rs2850379,rs35175534 |
| Right-hand grip strength | CHD | rs11642954, rs12889267, rs13107325, rs2431112, rs7196917 |
|  | Stroke | rs10205394, rs1044299, rs10786706, rs11002322 rs13091492, rs1981612, rs3814877, rs4335354, rs7124681，rs11642954, rs12889267, rs13107325, rs2431112, rs7196917 |
|  | MI | rs12889267,rs12452505,rs11642954,rs1044299, rs10786706, rs7124681，rs11642954, rs13107325, rs2431112, rs7196917,rs11002322, rs4335354 |
| CHD | ALM | rs11191416, rs1199338, rs2107595 |
|  | Left-hand grip strength | rs1199338, rs2107595, rs515135, rs663129, rs7528419 |
|  | Right-hand grip strength | rs1199338, rs2107595, rs515135, rs663129, rs7528419 |
| Stroke | ALM | rs2107595 |
|  | Left-hand grip strength | rs2107595 |
|  | Right-hand grip strength | rs2107595 |
| MI | ALM | rs698270 |
|  | Left-hand grip strength | rs698270 |
|  | Right-hand grip strength | rs698270 |

ALM, appendicular lean mass; CHD, coronary heart disease; MI, myocardial infarction;

**Supplementary Table 3.** The removed abnormal SNPs in MR-PRESSO analysis

| **Exposures** | **Outcomes** | **IVs selection** | **No. of IVs** | **The removed SNPs** |
| --- | --- | --- | --- | --- |
| ALM | CHD | All | 580 | rs10948, rs11187838, rs165849, rs17773965, rs2112617,rs2289629, rs3184504, rs6849302, rs7229520, rs72841270, rs757834 |
|  |  | Removed (11) | 569 |  |
|  | Stroke | ALL | 569 | rs10948,rs3184504,rs42039, rs4752689,rs650508 |
|  |  | Removed (5) | 564 |  |
|  | MI | ALL | 589 | rs14976, rs1514134, rs2885697, rs3184504, rs4900578, rs604723, rs6849302, rs7144307, rs9647379 |
|  |  | Removed (9) | 580 |  |
| Left-hand grip strength | CHD | ALL | 141 | rs116922558, rs62253653 |
|  |  | Removed (2) | 139 |  |
|  | Stroke | ALL | 132 | NA |
|  |  | Removed (0) | 132 |  |
|  | MI | ALL | 140 | rs4575361 |
|  |  | Removed (1) | 139 |  |
| Right-hand grip strength | CHD | ALL | 155 | rs116922558, rs1952256, rs4751671, rs600038, rs7301953 |
|  |  | Removed (5) | 150 |  |
|  | Stroke | All | 157 | NA |
|  |  | Removed (0) | 157 |  |
|  | MI | All | 161 | rs1442883, rs2226685, rs600038, rs7301953 |
|  |  | Removed (4) | 157 |  |
| CHD | ALM | All | 36 | rs10840293 rs11065979 ,rs11191416 rs115654617 rs11838776 rs17087335, rs28451064 rs3918226, rs56062135, rs56289821 rs56336142 rs663129 rs67180937 rs7528419 |
|  |  | Removed (14) | 22 |  |
|  | Left-hand grip strength | All | 32 | rs11065979, rs11191416, rs2519093, rs9349379 |
|  |  | Removed (4) | 28 |  |
|  | Right-hand grip strength | All | 32 | rs9349379,rs2519093, rs11065979，rs11191416 |
|  |  | Removed (4) | 28 |  |
| Stroke | ALM | All | 16 | rs1053007, rs12445022, rs17035646, rs2005108 rs3184504, rs35436, rs42039, rs4932370, rs7304841 |
|  |  | Removed (9) | 7 |  |
|  | Left-hand grip strength | All | 11 | rs3184504, rs42039, rs4932370 |
|  |  | Removed (3) | 8 |  |
|  | Right-hand grip strength | All | 10 | rs3184504, rs42039,rs4932370 |
|  |  | Removed (3) | 7 |  |
| MI | ALM | All | 31 | rs12740374, rs9349379, rs3918226, rs35350651, rs12906125, rs4846384, rs150289443, rs150289443, rs2886722, rs10404176, rs3127580 |
|  |  | Removed (11) | 20 |  |
|  | Left-hand grip strength | All | 26 | rs12740374, rs9349379 |
|  |  | Removed (2) | 24 |  |
|  | Right-hand grip strength | All | 22 | rs12740374， rs9349379 |
|  |  | Removed (2) | 20 |  |

ALM, appendicular lean mass; CHD, coronary heart disease; MI, myocardial infarction;

**Supplementary Table 4.** pleiotropy and heterogeneity tests between exposure factors and outcome variables.

| **Exposure** | **Outcome** | **No.IVs** | **Heterogeneity test** | **pleiotropy test** |
| --- | --- | --- | --- | --- |
|  |  |  | **Cochran’s Q (*p*)** | **Intercept*(p)*** |
| ALM | CHD | 569 | 859.104 (<0.001) | 2.535 e-03 (0.066) |
|  | Stroke | 564 | 753.974(<0.001) | -1.916 e-05 (0.877) |
|  | MI | 580 | 804.965(<0.001) | 5.951 e-04 (0.686) |
| left-hand grip strength | CHD | 139 | 228.871(<0.001) | 4.790 e-03 (0.325) |
|  | Stroke | 132 | 144.098(0.188)（FE) | -3.462 e-05 (0.370) |
|  | MI | 139 | 163.913( 0.058)（FE) | 4.397 e-03 (0.997) |
| right-hand grip strength | CHD | 150 | 257.708(<0.001) | 4.629 e-03 (0.557) |
|  | Stroke | 157 | 186.054(0.045)（FE) | -1.466 e-03 (0.690) |
|  | MI | 157 | 226.964(<0.001) | 1.161 e-03 (0.798) |
|  |  |  |  |  |
| CHD | ALM | 22 | 50.389(<0.001) | 1.719 e-03 (0.996) |
|  | Left-hand grip strength | 28 | 36.489(0.083)（FE) | 1.455 e-03 (0.119) |
|  | Right-hand grip strength | 28 | 39.480(0.044) | 6.522 e-04 (0.494) |
| Stroke | ALM | 7 | 9.417(0.094)（FE) | 7.750 e-03 (0.882) |
|  | Left-hand grip strength | 8 | 2.814(0.832)（FE) | 2.070 e-03 (0.518) |
|  | Right-hand grip strength | 7 | 1.308(0.968)（FE) | 2.040 e-03 (0.566) |
| MI | ALM | 20 | 17.276(<0.001) | 2.652 e-03 (0.812) |
|  | Left-hand grip strength | 24 | 31.154(0.093)（FE) | 1.707 e-03 (0.085) |
|  | Right-hand grip strength | 20 | 19.429(0.365)（FE) | 9.892 e-05 (0.330) |

ALM, appendicular lean mass; CHD, coronary heart disease; MI, myocardial infarction;

**Supplementary Table 5.** The removed abnormal SNPs in the one-and-leave method

| **Exposures** | **Outcomes** | **The removed SNPs** |
| --- | --- | --- |
| ALM | CHD | NA |
|  | Stroke | NA |
|  | MI | NA |
| left-hand grip strength | CHD | NA |
|  | Stroke | NA |
|  | MI | NA |
| right-hand grip strength | CHD | NA |
|  | Stroke | NA |
|  | MI | NA |
| CHD | ALM | NA |
|  | Left-hand grip strength | NA |
|  | Right-hand grip strength | NA |
| Stroke | ALM | NA |
|  | Left-hand grip strength | rs1052053, rs11957829, rs12445022, rs6847935, rs9526212 |
|  | Right-hand grip strength | rs11957829, s17035646, rs4959130, rs7304841, rs9526212, rs1052053 |
| MI | ALM | NA |
|  | Left-hand grip strength | NA |
|  | Right-hand grip strength | rs10455872， rs3127580， rs73015016， rs7485656 |

ALM, appendicular lean mass; CHD, coronary heart disease; MI, myocardial infarction;

**Supplementary Table 6.** Characteristics of selected SNPs concerning the causal effect of ALM on CHD

| **SNP** | **Beta** | **EAF** | ***P*-value** | **Effect**  **allele** | **Other**  **allele** | **SE** | ***F*-value** |
| --- | --- | --- | --- | --- | --- | --- | --- |
| rs10005035 | -0.0175 | 0.2823 | 6.51E-17 | G | C | 0.0021 | 55.880 |
| rs10019221 | -0.0124 | 0.5974 | 1.22E-10 | T | G | 0.0019 | 33.303 |
| rs1005723 | 0.0161 | 0.1908 | 1.79E-11 | T | C | 0.0024 | 36.041 |
| rs10068640 | 0.0112 | 0.3651 | 1.28E-08 | A | G | 0.002 | 26.185 |
| rs10075249 | 0.0143 | 0.4952 | 4.56E-14 | T | C | 0.0019 | 46.035 |
| rs10107388 | -0.0159 | 0.3691 | 6.95E-16 | C | T | 0.002 | 53.018 |
| rs10112506 | -0.012 | 0.3898 | 5.76E-10 | G | A | 0.0019 | 30.845 |
| rs10123619 | -0.0171 | 0.8425 | 4.05E-11 | G | A | 0.0026 | 34.942 |
| rs10128333 | -0.0146 | 0.1676 | 9.51E-09 | T | C | 0.0025 | 26.780 |
| rs10202701 | 0.0227 | 0.5419 | 3.11E-33 | T | C | 0.0019 | 115.217 |
| rs10202845 | -0.0288 | 0.1133 | 5.35E-22 | G | A | 0.003 | 75.048 |
| rs10203320 | 0.0138 | 0.3289 | 7.86E-12 | C | T | 0.002 | 37.855 |
| rs10205141 | 0.0241 | 0.0476 | 4.71E-08 | G | A | 0.0044 | 23.711 |
| rs10221831 | 0.03 | 0.0321 | 1.80E-08 | T | C | 0.0053 | 25.181 |
| rs10225945 | -0.0146 | 0.1499 | 3.28E-08 | G | A | 0.0026 | 24.461 |
| rs10242866 | 0.0157 | 0.3983 | 3.66E-16 | T | C | 0.0019 | 53.201 |
| rs10283100 | 0.0575 | 0.9445 | 4.11E-44 | G | A | 0.0041 | 156.119 |
| rs1035583 | 0.0148 | 0.6177 | 1.99E-14 | A | G | 0.0019 | 46.583 |
| rs10453441 | -0.0139 | 0.402 | 9.10E-13 | G | A | 0.002 | 41.828 |
| rs10461725 | 0.0134 | 0.6561 | 1.98E-11 | C | G | 0.002 | 36.486 |
| rs10471339 | -0.011 | 0.3823 | 1.45E-08 | G | C | 0.0019 | 25.732 |
| rs1047891 | 0.0233 | 0.3159 | 5.70E-31 | A | C | 0.002 | 105.672 |
| rs10483727 | -0.0368 | 0.6109 | 6.73E-80 | C | T | 0.0019 | 290.056 |
| rs1056747 | -0.0155 | 0.4117 | 8.05E-16 | G | A | 0.0019 | 52.405 |
| rs1063582 | -0.0185 | 0.7649 | 1.12E-16 | G | T | 0.0022 | 55.428 |
| rs10748128 | 0.0255 | 0.3446 | 6.77E-38 | T | G | 0.002 | 132.283 |
| rs10749157 | 0.0113 | 0.3584 | 1.24E-08 | C | T | 0.002 | 26.442 |
| rs10776560 | -0.0157 | 0.4995 | 7.88E-17 | T | C | 0.0019 | 55.497 |
| rs10793931 | -0.0132 | 0.3555 | 3.23E-11 | C | G | 0.002 | 35.952 |
| rs10796828 | 0.0154 | 0.6348 | 5.77E-15 | G | T | 0.002 | 49.514 |
| rs10815274 | 0.0124 | 0.4562 | 6.46E-11 | C | A | 0.0019 | 34.352 |
| rs10822117 | -0.0176 | 0.237 | 4.24E-15 | G | A | 0.0022 | 50.445 |
| rs10824307 | -0.0194 | 0.6471 | 1.79E-22 | C | G | 0.002 | 77.406 |
| rs10829226 | -0.0112 | 0.6359 | 1.33E-08 | A | G | 0.002 | 26.154 |
| rs10832963 | -0.0203 | 0.7445 | 9.23E-21 | G | T | 0.0022 | 70.598 |
| rs10845408 | 0.0255 | 0.3538 | 3.25E-38 | T | C | 0.002 | 133.909 |
| rs10858246 | -0.0188 | 0.3183 | 2.35E-20 | C | G | 0.002 | 69.070 |
| rs10864899 | -0.0112 | 0.5643 | 3.85E-09 | G | A | 0.0019 | 27.774 |
| rs10922475 | 0.0159 | 0.5395 | 2.17E-17 | A | C | 0.0019 | 56.565 |
| rs10962212 | 0.0143 | 0.4184 | 7.47E-14 | C | G | 0.0019 | 44.813 |
| rs10975935 | -0.0121 | 0.2464 | 4.15E-08 | G | A | 0.0022 | 24.482 |
| rs10982888 | -0.0328 | 0.1136 | 3.97E-28 | A | T | 0.003 | 97.572 |
| rs11014285 | 0.0342 | 0.1654 | 2.89E-40 | A | G | 0.0026 | 145.439 |
| rs11042717 | -0.029 | 0.4896 | 4.04E-53 | C | T | 0.0019 | 189.324 |
| rs11049704 | -0.0183 | 0.293 | 1.10E-18 | G | C | 0.0021 | 62.478 |
| rs11060942 | -0.0354 | 0.0344 | 7.49E-12 | A | G | 0.0052 | 37.486 |
| rs11068230 | 0.0238 | 0.8656 | 9.67E-18 | G | C | 0.0028 | 59.348 |
| rs11070842 | -0.0146 | 0.1631 | 1.30E-08 | C | T | 0.0026 | 26.202 |
| rs11098677 | -0.0263 | 0.7875 | 3.94E-30 | T | G | 0.0023 | 104.255 |
| rs11121615 | -0.0202 | 0.6894 | 3.32E-23 | T | C | 0.002 | 78.691 |
| rs111365325 | -0.0271 | 0.2312 | 1.12E-33 | T | C | 0.0022 | 117.579 |
| rs111622870 | -0.0282 | 0.0482 | 1.86E-10 | C | T | 0.0044 | 32.855 |
| rs11175919 | 0.0349 | 0.0262 | 3.16E-09 | A | G | 0.0059 | 27.985 |
| rs11191208 | 0.0147 | 0.2057 | 3.69E-10 | A | G | 0.0024 | 31.795 |
| rs11198591 | 0.0148 | 0.3684 | 4.91E-14 | A | G | 0.002 | 45.899 |
| rs112021215 | -0.0147 | 0.201 | 5.99E-09 | C | T | 0.0025 | 31.252 |
| rs11210892 | 0.0118 | 0.6745 | 3.57E-09 | A | G | 0.002 | 27.530 |
| rs112153300 | 0.0261 | 0.089 | 7.05E-15 | A | G | 0.0034 | 49.741 |
| rs11217863 | -0.0268 | 0.1162 | 1.06E-19 | A | G | 0.003 | 66.431 |
| rs11221657 | 0.0179 | 0.1352 | 1.21E-10 | G | T | 0.0028 | 33.737 |
| rs11243202 | 0.0302 | 0.486 | 2.83E-57 | C | T | 0.0019 | 205.251 |
| rs112537273 | -0.0212 | 0.2299 | 3.34E-21 | C | T | 0.0022 | 71.664 |
| rs11260035 | 0.015 | 0.2761 | 1.86E-12 | A | G | 0.0021 | 40.499 |
| rs11260623 | 0.0117 | 0.5085 | 4.86E-10 | T | G | 0.0019 | 30.810 |
| rs112873218 | 0.0216 | 0.1054 | 4.02E-12 | T | C | 0.0031 | 39.618 |
| rs113146332 | 0.0311 | 0.0381 | 2.99E-10 | C | G | 0.0049 | 31.921 |
| rs113232639 | 0.0327 | 0.493 | 4.79E-64 | A | G | 0.0019 | 240.801 |
| rs113289555 | -0.0206 | 0.2333 | 7.33E-20 | T | G | 0.0023 | 68.362 |
| rs113671109 | -0.015 | 0.22 | 4.23E-11 | C | T | 0.0023 | 34.770 |
| rs113827862 | -0.0235 | 0.0608 | 4.67E-09 | C | T | 0.004 | 28.399 |
| rs113898003 | -0.036 | 0.2635 | 1.19E-63 | C | T | 0.0021 | 226.596 |
| rs11580040 | 0.0325 | 0.08 | 6.76E-21 | G | A | 0.0035 | 70.014 |
| rs11590254 | 0.0186 | 0.3114 | 4.34E-20 | T | A | 0.002 | 66.811 |
| rs115912456 | 0.0577 | 0.0412 | 3.69E-34 | G | A | 0.0047 | 118.458 |
| rs116008080 | -0.0415 | 0.0238 | 4.06E-11 | A | G | 0.0063 | 36.035 |
| rs116052377 | 0.0225 | 0.0809 | 7.89E-11 | A | G | 0.0035 | 33.899 |
| rs11605297 | 0.0146 | 0.2328 | 8.03E-11 | A | G | 0.0022 | 34.285 |
| rs116092985 | -0.0401 | 0.0958 | 1.17E-34 | G | A | 0.0033 | 125.463 |
| rs11612462 | 0.015 | 0.1698 | 2.55E-09 | G | T | 0.0025 | 28.563 |
| rs11629593 | -0.0109 | 0.6158 | 4.42E-08 | G | T | 0.002 | 25.313 |
| rs11633371 | 0.0216 | 0.4764 | 7.49E-30 | T | G | 0.0019 | 104.823 |
| rs116339650 | -0.0175 | 0.127 | 1.05E-09 | G | A | 0.0029 | 30.577 |
| rs116493405 | 0.0287 | 0.0537 | 9.52E-12 | A | G | 0.0042 | 37.695 |
| rs11672848 | -0.0171 | 0.5246 | 7.73E-19 | T | C | 0.0019 | 65.678 |
| rs11684531 | -0.0172 | 0.1328 | 4.17E-10 | G | A | 0.0028 | 30.682 |
| rs1168768 | 0.0332 | 0.975 | 3.61E-08 | T | C | 0.006 | 24.195 |
| rs116919274 | 0.0271 | 0.046 | 3.52E-09 | A | G | 0.0046 | 29.023 |
| rs117068593 | 0.0403 | 0.1895 | 8.83E-62 | T | C | 0.0024 | 224.732 |
| rs117203652 | -0.0346 | 0.03 | 4.32E-10 | A | G | 0.0055 | 31.373 |
| rs11720869 | 0.0141 | 0.6686 | 2.54E-12 | A | G | 0.002 | 39.671 |
| rs11721522 | 0.0106 | 0.4125 | 4.03E-08 | G | A | 0.0019 | 24.521 |
| rs11727162 | -0.017 | 0.4987 | 2.15E-19 | T | C | 0.0019 | 65.069 |
| rs117335233 | -0.0236 | 0.053 | 2.56E-08 | G | T | 0.0042 | 25.174 |
| rs1177765 | -0.0232 | 0.4681 | 1.32E-34 | C | T | 0.0019 | 120.708 |
| rs11778491 | -0.0247 | 0.2518 | 8.39E-30 | C | G | 0.0022 | 103.524 |
| rs117818446 | 0.0423 | 0.0202 | 5.29E-10 | A | G | 0.0068 | 31.892 |
| rs117972846 | 0.0335 | 0.0285 | 5.47E-09 | G | T | 0.0057 | 27.982 |
| rs11867855 | -0.0132 | 0.2372 | 2.93E-09 | T | G | 0.0022 | 28.391 |
| rs1190540 | 0.0125 | 0.6983 | 1.62E-09 | G | A | 0.0021 | 29.644 |
| rs11959466 | 0.038 | 0.0564 | 2.24E-19 | T | C | 0.0042 | 69.211 |
| rs1202186 | -0.012 | 0.6548 | 1.65E-09 | T | C | 0.002 | 29.312 |
| rs12051245 | 0.0299 | 0.2318 | 2.56E-40 | C | T | 0.0022 | 143.398 |
| rs12074850 | 0.0393 | 0.0898 | 2.72E-33 | G | A | 0.0033 | 113.706 |
| rs12099669 | 0.0331 | 0.6962 | 1.39E-58 | A | G | 0.002 | 208.763 |
| rs12150907 | -0.0219 | 0.1977 | 7.22E-20 | A | G | 0.0024 | 68.513 |
| rs12185775 | -0.0167 | 0.1172 | 3.66E-08 | C | G | 0.003 | 25.985 |
| rs12188208 | -0.0195 | 0.2349 | 1.40E-18 | C | A | 0.0022 | 61.547 |
| rs12230946 | 0.0271 | 0.0911 | 1.45E-16 | A | G | 0.0033 | 54.765 |
| rs12340775 | -0.0287 | 0.0552 | 1.73E-11 | A | G | 0.0043 | 38.686 |
| rs12344515 | -0.0163 | 0.2402 | 2.28E-13 | T | C | 0.0022 | 43.668 |
| rs12347137 | -0.046 | 0.2023 | 9.80E-85 | C | A | 0.0024 | 307.697 |
| rs12351226 | 0.0218 | 0.1712 | 9.13E-18 | T | C | 0.0025 | 60.730 |
| rs12423821 | 0.0161 | 0.1575 | 1.17E-09 | C | T | 0.0027 | 30.975 |
| rs12461874 | -0.0181 | 0.2784 | 1.26E-17 | A | C | 0.0021 | 59.273 |
| rs12483401 | -0.0387 | 0.0216 | 9.22E-09 | C | T | 0.0067 | 28.503 |
| rs12512942 | -0.0162 | 0.6376 | 1.58E-16 | A | G | 0.002 | 54.613 |
| rs12517711 | -0.0147 | 0.3917 | 2.79E-14 | C | T | 0.0019 | 46.369 |
| rs12519407 | 0.0181 | 0.2567 | 3.38E-17 | C | A | 0.0022 | 56.296 |
| rs12533452 | 0.0237 | 0.1569 | 1.31E-19 | T | C | 0.0026 | 66.917 |
| rs12536902 | 0.0479 | 0.0143 | 3.66E-09 | A | C | 0.0081 | 29.124 |
| rs12541381 | -0.0319 | 0.2575 | 2.81E-49 | A | G | 0.0022 | 175.267 |
| rs12563442 | 0.0122 | 0.2669 | 9.93E-09 | C | T | 0.0021 | 26.226 |
| rs1260326 | 0.0323 | 0.6045 | 6.16E-64 | C | T | 0.0019 | 224.719 |
| rs12616192 | -0.0261 | 0.0676 | 6.83E-12 | A | G | 0.0038 | 38.667 |
| rs12655296 | -0.011 | 0.6251 | 1.62E-08 | T | C | 0.002 | 25.536 |
| rs12672217 | 0.0139 | 0.3609 | 1.33E-12 | A | G | 0.002 | 40.133 |
| rs12700901 | -0.0184 | 0.4055 | 1.67E-21 | A | C | 0.0019 | 73.506 |
| rs12702693 | 0.0173 | 0.4542 | 6.56E-20 | T | C | 0.0019 | 66.821 |
| rs12713004 | 0.0367 | 0.7254 | 2.40E-68 | G | A | 0.0021 | 241.723 |
| rs12724708 | 0.0243 | 0.3574 | 1.66E-35 | T | A | 0.002 | 122.152 |
| rs12773500 | 0.0171 | 0.1375 | 5.05E-10 | T | C | 0.0028 | 31.229 |
| rs12831751 | 0.0172 | 0.2857 | 1.57E-16 | C | A | 0.0021 | 54.372 |
| rs12882130 | -0.0202 | 0.3836 | 1.88E-24 | G | C | 0.002 | 86.897 |
| rs12907139 | -0.0149 | 0.5245 | 4.89E-15 | A | G | 0.0019 | 49.865 |
| rs1290786 | -0.0143 | 0.4307 | 7.14E-14 | T | C | 0.0019 | 45.155 |
| rs12909863 | 0.0189 | 0.2512 | 6.05E-18 | C | G | 0.0022 | 60.512 |
| rs12926103 | 0.0272 | 0.0661 | 9.60E-13 | A | G | 0.0038 | 41.130 |
| rs12943867 | 0.0184 | 0.3367 | 7.57E-20 | A | G | 0.002 | 68.097 |
| rs12962050 | 0.0153 | 0.6445 | 1.52E-14 | A | G | 0.002 | 48.302 |
| rs12997625 | -0.017 | 0.5266 | 1.50E-19 | T | C | 0.0019 | 64.885 |
| rs13037813 | 0.0292 | 0.2391 | 1.71E-39 | C | T | 0.0022 | 139.728 |
| rs13103161 | -0.0284 | 0.3891 | 2.38E-48 | A | T | 0.0019 | 172.707 |
| rs13109280 | 0.0131 | 0.6618 | 9.15E-11 | G | A | 0.002 | 34.590 |
| rs13123591 | 0.0185 | 0.3375 | 2.35E-20 | G | T | 0.002 | 68.920 |
| rs13127468 | -0.0123 | 0.4629 | 9.86E-11 | A | C | 0.0019 | 33.874 |
| rs13170063 | -0.0152 | 0.5921 | 4.11E-15 | A | G | 0.0019 | 50.253 |
| rs1319012 | -0.052 | 0.9259 | 3.30E-45 | A | T | 0.0037 | 167.119 |
| rs13209685 | 0.0277 | 0.1594 | 7.49E-27 | T | G | 0.0026 | 92.598 |
| rs1324538 | 0.0237 | 0.3836 | 1.73E-34 | A | T | 0.0019 | 119.627 |
| rs1325596 | 0.0287 | 0.5476 | 2.77E-52 | A | G | 0.0019 | 183.824 |
| rs1330826 | 0.0162 | 0.2268 | 1.04E-12 | C | G | 0.0023 | 41.446 |
| rs13316 | 0.0115 | 0.4301 | 3.66E-09 | A | C | 0.0019 | 29.192 |
| rs13391980 | -0.0225 | 0.1198 | 7.60E-15 | A | G | 0.0029 | 48.076 |
| rs1340022 | 0.0118 | 0.4868 | 4.47E-10 | C | T | 0.0019 | 31.326 |
| rs1341215 | 0.0229 | 0.1374 | 6.32E-17 | A | G | 0.0027 | 55.975 |
| rs13430869 | 0.0272 | 0.7427 | 6.37E-37 | T | G | 0.0021 | 127.347 |
| rs139163241 | -0.0164 | 0.1394 | 1.79E-09 | G | T | 0.0027 | 29.057 |
| rs140440099 | 0.0613 | 0.0234 | 1.45E-22 | A | G | 0.0063 | 77.340 |
| rs1405227 | 0.0129 | 0.3167 | 1.57E-10 | A | G | 0.002 | 32.430 |
| rs143384 | 0.0725 | 0.4038 | 1.00E-200 | G | A | 0.0019 | 1142.378 |
| rs143554698 | -0.0257 | 0.1409 | 3.39E-21 | T | C | 0.0027 | 72.005 |
| rs144109601 | -0.0278 | 0.0418 | 5.15E-09 | A | C | 0.0048 | 27.876 |
| rs1443536 | 0.0218 | 0.305 | 1.91E-26 | G | A | 0.0021 | 90.732 |
| rs1444628 | 0.024 | 0.6904 | 6.85E-32 | T | C | 0.002 | 110.894 |
| rs144627572 | 0.0439 | 0.0329 | 1.30E-16 | A | G | 0.0053 | 55.224 |
| rs147110934 | -0.0722 | 0.0243 | 9.39E-32 | T | G | 0.0062 | 111.322 |
| rs147233090 | -0.0446 | 0.0247 | 3.95E-13 | T | C | 0.0061 | 43.154 |
| rs1472852 | -0.0638 | 0.1582 | 8.22E-135 | A | C | 0.0026 | 488.656 |
| rs1478575 | 0.0312 | 0.6843 | 5.20E-54 | A | T | 0.002 | 189.447 |
| rs14976 | 0.0144 | 0.3057 | 1.43E-12 | T | C | 0.002 | 39.635 |
| rs1514134 | -0.0114 | 0.3849 | 3.57E-09 | C | T | 0.0019 | 27.708 |
| rs1556659 | 0.0163 | 0.3818 | 7.19E-17 | T | C | 0.002 | 56.477 |
| rs1584011 | 0.0159 | 0.3559 | 9.78E-16 | G | T | 0.002 | 52.192 |
| rs16989695 | -0.0139 | 0.5165 | 1.93E-13 | A | G | 0.0019 | 43.452 |
| rs17197114 | 0.0177 | 0.1772 | 1.54E-12 | C | T | 0.0025 | 41.136 |
| rs17205463 | -0.0263 | 0.4477 | 4.21E-43 | T | C | 0.0019 | 154.063 |
| rs17246129 | 0.0254 | 0.3044 | 1.27E-35 | A | G | 0.002 | 123.045 |
| rs17278379 | 0.0226 | 0.124 | 2.40E-15 | C | T | 0.0029 | 49.965 |
| rs1730028 | 0.0131 | 0.4174 | 7.39E-12 | G | T | 0.0019 | 37.582 |
| rs173135 | -0.0341 | 0.115 | 3.25E-30 | T | C | 0.003 | 106.593 |
| rs17400325 | 0.0345 | 0.0415 | 2.10E-13 | C | T | 0.0047 | 42.638 |
| rs17478946 | -0.0192 | 0.3004 | 9.98E-21 | G | A | 0.0021 | 69.774 |
| rs17681189 | -0.0131 | 0.4231 | 5.82E-12 | A | C | 0.0019 | 37.722 |
| rs17718736 | 0.0115 | 0.3226 | 1.39E-08 | A | C | 0.002 | 26.026 |
| rs177591 | -0.0191 | 0.1489 | 1.51E-12 | G | C | 0.0027 | 41.635 |
| rs17818592 | -0.0129 | 0.4367 | 1.23E-11 | C | T | 0.0019 | 36.865 |
| rs1786263 | -0.019 | 0.6055 | 1.03E-22 | T | G | 0.0019 | 77.664 |
| rs1797070 | 0.0219 | 0.2684 | 4.93E-25 | A | G | 0.0021 | 84.821 |
| rs1823217 | -0.0181 | 0.6449 | 4.06E-20 | G | A | 0.002 | 67.568 |
| rs182798714 | 0.0376 | 0.0274 | 1.45E-09 | T | A | 0.0062 | 33.929 |
| rs1880318 | 0.0147 | 0.2039 | 6.86E-10 | A | G | 0.0024 | 31.588 |
| rs1899040 | 0.0152 | 0.7962 | 9.04E-11 | T | C | 0.0023 | 33.761 |
| rs190823861 | -0.0345 | 0.0466 | 2.09E-14 | A | G | 0.0045 | 47.623 |
| rs1933081 | 0.0267 | 0.0834 | 5.33E-15 | A | T | 0.0034 | 49.078 |
| rs200439 | -0.0128 | 0.2207 | 1.51E-08 | C | A | 0.0023 | 25.376 |
| rs2005172 | 0.048 | 0.6397 | 2.35E-128 | C | A | 0.002 | 478.696 |
| rs2019203 | 0.0189 | 0.4909 | 1.84E-23 | A | C | 0.0019 | 80.403 |
| rs2025609 | 0.0186 | 0.8511 | 2.00E-12 | G | C | 0.0026 | 39.483 |
| rs2025808 | 0.0122 | 0.2541 | 1.72E-08 | A | C | 0.0022 | 25.404 |
| rs2035901 | 0.024 | 0.4679 | 9.43E-37 | G | A | 0.0019 | 129.172 |
| rs2070598 | 0.0204 | 0.4559 | 6.36E-27 | A | G | 0.0019 | 92.977 |
| rs2071450 | -0.0174 | 0.3675 | 8.85E-19 | T | C | 0.002 | 63.380 |
| rs2089111 | -0.0172 | 0.267 | 1.73E-15 | G | C | 0.0022 | 52.143 |
| rs2101017 | -0.0223 | 0.8695 | 1.44E-15 | T | C | 0.0028 | 50.818 |
| rs2105333 | -0.019 | 0.6648 | 1.70E-21 | G | T | 0.002 | 72.451 |
| rs212526 | 0.0214 | 0.6007 | 3.84E-29 | C | T | 0.0019 | 98.936 |
| rs2138374 | -0.0149 | 0.696 | 2.79E-13 | C | T | 0.002 | 42.303 |
| rs2140619 | 0.0113 | 0.4128 | 5.18E-09 | G | A | 0.0019 | 27.873 |
| rs2142331 | -0.0165 | 0.6023 | 1.38E-17 | T | C | 0.0019 | 58.731 |
| rs2142644 | -0.0181 | 0.672 | 3.37E-19 | A | C | 0.002 | 65.034 |
| rs2181834 | 0.0254 | 0.5503 | 7.69E-41 | T | G | 0.0019 | 143.815 |
| rs2188805 | 0.0114 | 0.3359 | 2.00E-08 | C | A | 0.002 | 26.107 |
| rs2194411 | 0.0443 | 0.1283 | 2.43E-54 | A | G | 0.0029 | 197.727 |
| rs2209098 | 0.024 | 0.3108 | 1.73E-32 | C | T | 0.002 | 111.130 |
| rs2212926 | -0.022 | 0.2106 | 7.75E-21 | A | C | 0.0023 | 72.468 |
| rs2229840 | 0.0341 | 0.1597 | 3.02E-40 | T | C | 0.0026 | 140.559 |
| rs2230033 | -0.0265 | 0.5644 | 3.49E-43 | A | G | 0.0019 | 155.522 |
| rs2236096 | 0.018 | 0.2327 | 1.29E-15 | C | T | 0.0023 | 52.099 |
| rs2236406 | 0.0394 | 0.3493 | 1.26E-87 | C | T | 0.002 | 317.946 |
| rs2237485 | 0.0191 | 0.2234 | 3.73E-17 | A | G | 0.0023 | 57.000 |
| rs2240735 | 0.0189 | 0.748 | 3.99E-18 | T | C | 0.0022 | 60.640 |
| rs2268718 | 0.0141 | 0.2704 | 3.24E-11 | T | C | 0.0021 | 35.321 |
| rs2270894 | -0.0332 | 0.2034 | 1.25E-42 | G | C | 0.0024 | 160.878 |
| rs2274351 | 0.017 | 0.5433 | 3.07E-19 | T | C | 0.0019 | 64.581 |
| rs2283200 | -0.0281 | 0.0559 | 1.48E-11 | T | C | 0.0042 | 37.528 |
| rs2296316 | -0.0192 | 0.4643 | 1.59E-23 | C | T | 0.0019 | 82.580 |
| rs2303423 | 0.0168 | 0.1095 | 2.64E-08 | C | T | 0.003 | 24.784 |
| rs2305141 | 0.0183 | 0.5961 | 1.07E-21 | G | A | 0.0019 | 72.617 |
| rs2324154 | 0.015 | 0.5096 | 1.92E-15 | A | C | 0.0019 | 50.639 |
| rs234640 | -0.0131 | 0.5142 | 3.87E-12 | T | C | 0.0019 | 38.605 |
| rs2347603 | -0.0181 | 0.7418 | 5.65E-17 | A | T | 0.0022 | 56.511 |
| rs2347808 | -0.0125 | 0.5139 | 5.79E-11 | A | G | 0.0019 | 35.151 |
| rs2362487 | 0.0154 | 0.2473 | 3.57E-12 | G | C | 0.0022 | 39.756 |
| rs2390669 | 0.0174 | 0.1289 | 7.00E-10 | C | A | 0.0028 | 30.614 |
| rs244711 | 0.0279 | 0.686 | 1.54E-37 | T | C | 0.0022 | 151.037 |
| rs2454390 | -0.0176 | 0.8459 | 1.74E-11 | C | T | 0.0026 | 36.363 |
| rs246177 | 0.0214 | 0.3681 | 2.04E-27 | T | C | 0.002 | 95.942 |
| rs2490302 | 0.0221 | 0.9137 | 6.24E-11 | A | T | 0.0034 | 34.682 |
| rs249677 | -0.0109 | 0.6333 | 2.40E-08 | A | C | 0.002 | 24.847 |
| rs2521349 | 0.0155 | 0.3847 | 2.01E-15 | A | G | 0.0019 | 51.215 |
| rs2529090 | 0.0136 | 0.181 | 3.39E-08 | G | C | 0.0025 | 24.691 |
| rs2545339 | 0.0115 | 0.6288 | 3.48E-09 | G | A | 0.002 | 27.798 |
| rs2569888 | 0.0133 | 0.2449 | 2.29E-09 | A | G | 0.0022 | 29.458 |
| rs2578565 | -0.0141 | 0.6575 | 1.37E-12 | T | C | 0.002 | 40.319 |
| rs2592208 | -0.0124 | 0.513 | 5.52E-11 | A | C | 0.0019 | 34.594 |
| rs2607234 | -0.0302 | 0.9476 | 2.00E-12 | G | A | 0.0043 | 40.784 |
| rs261223 | 0.0175 | 0.3702 | 2.30E-19 | C | A | 0.0019 | 64.306 |
| rs2648725 | 0.0165 | 0.2134 | 8.20E-13 | A | T | 0.0023 | 41.156 |
| rs2663126 | -0.0139 | 0.6905 | 1.36E-11 | A | G | 0.0021 | 37.185 |
| rs2676298 | -0.0269 | 0.853 | 2.39E-23 | T | C | 0.0027 | 81.719 |
| rs2717008 | -0.0127 | 0.3835 | 4.99E-11 | C | T | 0.0019 | 34.341 |
| rs2748501 | -0.0195 | 0.4395 | 1.31E-24 | A | G | 0.0019 | 84.365 |
| rs2754255 | -0.0153 | 0.2245 | 1.05E-11 | G | A | 0.0023 | 36.702 |
| rs2763263 | -0.017 | 0.2445 | 1.37E-14 | A | T | 0.0022 | 48.076 |
| rs2788213 | 0.0123 | 0.7104 | 3.80E-09 | A | G | 0.0021 | 28.029 |
| rs2789365 | -0.0145 | 0.4807 | 1.14E-14 | T | C | 0.0019 | 47.266 |
| rs2807339 | 0.0162 | 0.7586 | 1.24E-13 | C | T | 0.0022 | 43.281 |
| rs2812208 | 0.1156 | 0.021 | 5.51E-68 | C | G | 0.0066 | 247.532 |
| rs28379706 | 0.0114 | 0.3931 | 4.49E-09 | C | T | 0.002 | 27.921 |
| rs28485212 | -0.0188 | 0.1494 | 1.24E-12 | T | C | 0.0027 | 40.449 |
| rs28529055 | -0.0147 | 0.4368 | 1.88E-14 | T | G | 0.0019 | 47.874 |
| rs28529426 | -0.0168 | 0.1711 | 5.82E-11 | T | C | 0.0026 | 36.048 |
| rs28592876 | 0.03 | 0.2051 | 9.09E-38 | A | G | 0.0023 | 132.167 |
| rs2871865 | -0.0493 | 0.1159 | 3.40E-62 | G | C | 0.003 | 224.373 |
| rs2871960 | 0.0469 | 0.4448 | 2.17E-135 | C | A | 0.0019 | 489.674 |
| rs28736838 | -0.0117 | 0.3017 | 1.07E-08 | T | C | 0.002 | 25.971 |
| rs2885697 | -0.0323 | 0.665 | 9.21E-60 | T | G | 0.002 | 209.386 |
| rs291979 | 0.0242 | 0.2289 | 6.76E-27 | A | G | 0.0023 | 93.101 |
| rs2923411 | 0.0127 | 0.5946 | 4.77E-11 | C | T | 0.0019 | 35.013 |
| rs2925155 | -0.015 | 0.2612 | 5.47E-12 | T | C | 0.0022 | 39.102 |
| rs293517 | -0.013 | 0.7011 | 2.80E-10 | C | T | 0.0021 | 31.893 |
| rs2971857 | -0.0119 | 0.5752 | 3.70E-10 | A | G | 0.0019 | 31.160 |
| rs2978362 | 0.0106 | 0.5326 | 2.85E-08 | T | C | 0.0019 | 25.188 |
| rs301807 | -0.0144 | 0.5815 | 2.53E-14 | G | A | 0.0019 | 45.445 |
| rs3103223 | 0.0126 | 0.7397 | 6.02E-09 | C | T | 0.0022 | 27.528 |
| rs310796 | 0.0142 | 0.6811 | 2.53E-12 | T | G | 0.002 | 39.442 |
| rs3116194 | -0.0295 | 0.0978 | 8.29E-21 | A | T | 0.0032 | 69.156 |
| rs3116602 | -0.0612 | 0.2153 | 9.53E-155 | G | T | 0.0023 | 570.526 |
| rs31196 | -0.0107 | 0.5724 | 2.07E-08 | A | C | 0.0019 | 25.235 |
| rs3205136 | -0.0184 | 0.0952 | 1.85E-08 | A | C | 0.0033 | 26.262 |
| rs331917 | -0.0127 | 0.5803 | 3.54E-11 | G | A | 0.0019 | 35.376 |
| rs332116 | -0.0206 | 0.2804 | 2.89E-22 | T | C | 0.0021 | 77.118 |
| rs336630 | -0.0106 | 0.43 | 2.90E-08 | T | C | 0.0019 | 24.800 |
| rs33973388 | 0.0249 | 0.4353 | 1.45E-38 | T | G | 0.0019 | 137.282 |
| rs34287 | 0.0187 | 0.341 | 1.17E-20 | A | G | 0.002 | 70.773 |
| rs34312629 | -0.017 | 0.2613 | 2.12E-15 | G | C | 0.0021 | 50.238 |
| rs34338597 | -0.0112 | 0.3832 | 7.88E-09 | G | A | 0.0019 | 26.700 |
| rs34345560 | 0.0219 | 0.1952 | 7.10E-20 | A | G | 0.0024 | 67.857 |
| rs34390533 | -0.0257 | 0.248 | 6.69E-32 | A | C | 0.0022 | 110.948 |
| rs34522021 | 0.0126 | 0.4547 | 3.38E-11 | T | C | 0.0019 | 35.450 |
| rs34776209 | -0.0317 | 0.2475 | 1.78E-47 | T | C | 0.0022 | 168.592 |
| rs34879158 | -0.0363 | 0.263 | 1.55E-63 | C | A | 0.0022 | 230.109 |
| rs35073631 | 0.0112 | 0.4331 | 5.92E-09 | C | T | 0.0019 | 27.735 |
| rs350832 | -0.0165 | 0.7709 | 3.44E-13 | A | G | 0.0023 | 43.302 |
| rs35268848 | 0.0737 | 0.0119 | 2.83E-13 | A | C | 0.0101 | 57.519 |
| rs35288270 | -0.0328 | 0.1344 | 3.43E-32 | C | T | 0.0028 | 112.732 |
| rs35732917 | 0.0204 | 0.2843 | 2.08E-22 | C | T | 0.0021 | 76.264 |
| rs35756741 | -0.0378 | 0.0924 | 5.80E-31 | T | C | 0.0033 | 107.927 |
| rs35811052 | -0.0148 | 0.2559 | 8.84E-12 | G | A | 0.0022 | 37.561 |
| rs35816944 | -0.1088 | 0.0066 | 1.27E-20 | A | G | 0.0117 | 69.899 |
| rs35963161 | -0.0157 | 0.4757 | 7.46E-16 | A | G | 0.0019 | 55.366 |
| rs36000545 | -0.022 | 0.3957 | 2.56E-29 | G | A | 0.002 | 104.241 |
| rs36012032 | 0.0298 | 0.091 | 9.93E-20 | A | C | 0.0033 | 66.157 |
| rs36048468 | 0.0254 | 0.2088 | 9.34E-28 | T | C | 0.0023 | 95.996 |
| rs36226649 | 0.0485 | 0.0667 | 3.05E-37 | C | T | 0.0038 | 131.896 |
| rs3768495 | -0.0178 | 0.7173 | 1.07E-17 | T | C | 0.0021 | 57.863 |
| rs3769598 | 0.0171 | 0.1438 | 1.32E-10 | G | A | 0.0027 | 32.421 |
| rs377599 | 0.0217 | 0.383 | 3.50E-29 | T | C | 0.0019 | 100.225 |
| rs3778858 | 0.0108 | 0.3698 | 4.26E-08 | T | G | 0.002 | 24.479 |
| rs3782232 | -0.0339 | 0.0712 | 2.41E-20 | A | G | 0.0037 | 68.445 |
| rs3782811 | -0.0165 | 0.2507 | 3.96E-14 | A | C | 0.0022 | 46.057 |
| rs3792819 | 0.021 | 0.0854 | 4.42E-10 | G | A | 0.0034 | 31.019 |
| rs3818416 | 0.0279 | 0.7645 | 2.01E-35 | C | A | 0.0022 | 126.233 |
| rs3822742 | 0.0162 | 0.3706 | 1.03E-16 | A | C | 0.002 | 55.130 |
| rs3828729 | -0.016 | 0.3094 | 4.67E-15 | G | A | 0.002 | 49.262 |
| rs395980 | -0.0184 | 0.2635 | 1.02E-17 | G | T | 0.0021 | 59.173 |
| rs40270 | 0.0151 | 0.7724 | 1.90E-11 | C | A | 0.0022 | 36.098 |
| rs4073154 | 0.0274 | 0.7784 | 1.92E-33 | G | A | 0.0023 | 116.644 |
| rs4076108 | 0.0174 | 0.2449 | 2.30E-15 | T | A | 0.0022 | 50.421 |
| rs4077103 | -0.0143 | 0.8426 | 4.18E-08 | A | C | 0.0026 | 24.423 |
| rs4121583 | 0.0118 | 0.6192 | 4.62E-09 | T | C | 0.002 | 29.566 |
| rs41311445 | -0.0328 | 0.0958 | 4.73E-24 | C | A | 0.0032 | 83.933 |
| rs4244809 | -0.0262 | 0.2082 | 5.55E-29 | A | G | 0.0023 | 101.923 |
| rs4252548 | -0.0753 | 0.0218 | 2.96E-31 | T | C | 0.0065 | 108.907 |
| rs4274112 | -0.0217 | 0.3733 | 2.47E-28 | G | A | 0.002 | 99.222 |
| rs4282339 | -0.0311 | 0.2075 | 6.16E-41 | A | G | 0.0023 | 143.269 |
| rs4287835 | 0.0147 | 0.534 | 9.94E-15 | C | T | 0.0019 | 48.427 |
| rs4380799 | -0.0255 | 0.39 | 6.45E-33 | G | T | 0.0021 | 139.343 |
| rs4383083 | 0.0111 | 0.6466 | 2.91E-08 | A | G | 0.002 | 25.354 |
| rs447352 | -0.0181 | 0.1408 | 6.62E-10 | T | C | 0.0029 | 35.691 |
| rs4504126 | 0.046 | 0.0275 | 1.62E-15 | C | A | 0.0058 | 50.964 |
| rs45474992 | -0.0617 | 0.0362 | 2.17E-33 | T | C | 0.0051 | 119.634 |
| rs45528934 | 0.0262 | 0.1623 | 1.97E-24 | T | C | 0.0026 | 84.055 |
| rs4602848 | 0.016 | 0.6812 | 3.14E-15 | G | A | 0.002 | 50.068 |
| rs4622329 | 0.0149 | 0.3496 | 8.62E-14 | A | G | 0.002 | 45.461 |
| rs4640244 | -0.02 | 0.3993 | 3.81E-25 | G | A | 0.0019 | 86.412 |
| rs4644481 | -0.0112 | 0.4328 | 3.54E-09 | T | C | 0.0019 | 27.731 |
| rs4655345 | -0.0246 | 0.3974 | 5.81E-38 | G | A | 0.0019 | 130.535 |
| rs4682483 | -0.0165 | 0.1534 | 2.65E-10 | A | G | 0.0026 | 31.840 |
| rs4683435 | 0.0144 | 0.7723 | 1.60E-10 | G | A | 0.0022 | 32.838 |
| rs4735761 | 0.0331 | 0.2857 | 3.66E-56 | C | A | 0.0021 | 201.426 |
| rs4748008 | -0.0125 | 0.4346 | 8.95E-11 | C | T | 0.0019 | 34.576 |
| rs4752689 | 0.0205 | 0.5839 | 1.36E-26 | A | G | 0.0019 | 91.962 |
| rs4752829 | 0.0262 | 0.2861 | 5.90E-36 | A | G | 0.0021 | 126.286 |
| rs4788218 | 0.0275 | 0.401 | 5.52E-46 | C | T | 0.0019 | 163.632 |
| rs4807472 | -0.0158 | 0.6764 | 8.18E-15 | C | T | 0.002 | 49.209 |
| rs4815952 | -0.0161 | 0.5221 | 1.24E-16 | C | T | 0.0019 | 58.247 |
| rs4818280 | -0.0124 | 0.6269 | 2.84E-10 | T | C | 0.002 | 32.387 |
| rs4847378 | 0.0136 | 0.6121 | 1.64E-12 | T | G | 0.0019 | 39.549 |
| rs4852257 | -0.0231 | 0.5763 | 6.21E-34 | G | T | 0.0019 | 117.360 |
| rs4865956 | -0.0258 | 0.6965 | 3.85E-36 | A | T | 0.0021 | 126.741 |
| rs4870941 | -0.0297 | 0.238 | 1.09E-39 | C | G | 0.0023 | 144.098 |
| rs488621 | 0.0191 | 0.4688 | 2.86E-24 | G | A | 0.0019 | 81.821 |
| rs4932439 | -0.0151 | 0.8254 | 1.43E-09 | G | A | 0.0025 | 29.591 |
| rs4938359 | -0.0156 | 0.2024 | 3.24E-11 | G | A | 0.0024 | 35.380 |
| rs4940874 | 0.0148 | 0.8119 | 1.24E-09 | G | A | 0.0024 | 30.124 |
| rs4965298 | -0.0119 | 0.7127 | 1.81E-08 | T | C | 0.0021 | 26.112 |
| rs496783 | -0.0124 | 0.4648 | 8.13E-11 | G | A | 0.0019 | 34.446 |
| rs4976262 | -0.0245 | 0.3156 | 4.37E-33 | C | T | 0.002 | 116.780 |
| rs532499 | -0.0127 | 0.7413 | 4.90E-09 | C | T | 0.0022 | 27.855 |
| rs543650 | 0.025 | 0.5987 | 1.49E-37 | G | T | 0.002 | 135.258 |
| rs544136 | 0.0121 | 0.7482 | 2.50E-08 | G | T | 0.0022 | 24.840 |
| rs545104 | 0.0127 | 0.6164 | 8.08E-11 | C | T | 0.002 | 34.344 |
| rs55717234 | 0.0122 | 0.5732 | 1.34E-10 | G | A | 0.0019 | 32.791 |
| rs55758152 | 0.0145 | 0.3263 | 1.05E-12 | A | G | 0.002 | 41.623 |
| rs55852614 | -0.0393 | 0.2474 | 3.29E-73 | C | T | 0.0022 | 259.104 |
| rs55872725 | 0.0222 | 0.4037 | 1.46E-30 | T | C | 0.0019 | 106.858 |
| rs56112295 | 0.0154 | 0.2257 | 1.12E-10 | T | C | 0.0024 | 37.324 |
| rs56207600 | 0.0192 | 0.111 | 2.50E-10 | A | G | 0.003 | 32.759 |
| rs56239180 | -0.0459 | 0.0237 | 1.85E-13 | G | T | 0.0062 | 43.901 |
| rs56363908 | -0.0382 | 0.0422 | 3.88E-16 | G | A | 0.0047 | 53.118 |
| rs568267 | 0.0122 | 0.7431 | 2.23E-08 | T | C | 0.0022 | 25.588 |
| rs57059662 | 0.0118 | 0.6774 | 5.63E-09 | C | T | 0.002 | 27.402 |
| rs5742915 | 0.0248 | 0.4605 | 9.33E-39 | C | T | 0.0019 | 137.636 |
| rs57513571 | -0.0191 | 0.2004 | 7.02E-16 | T | C | 0.0024 | 52.646 |
| rs5753518 | 0.0242 | 0.0899 | 5.04E-13 | A | G | 0.0033 | 43.152 |
| rs57696574 | 0.0173 | 0.3981 | 4.50E-18 | C | A | 0.002 | 64.587 |
| rs577289 | -0.0125 | 0.2808 | 5.39E-09 | T | A | 0.0021 | 28.416 |
| rs57791149 | -0.0173 | 0.4024 | 3.26E-19 | C | T | 0.0019 | 64.818 |
| rs599004 | -0.0157 | 0.2807 | 6.53E-14 | T | C | 0.0021 | 44.820 |
| rs59950280 | -0.0254 | 0.3316 | 7.32E-36 | A | G | 0.002 | 128.801 |
| rs6000886 | 0.0131 | 0.6482 | 5.88E-11 | C | T | 0.002 | 35.242 |
| rs6028716 | -0.021 | 0.2587 | 4.58E-22 | A | G | 0.0022 | 76.169 |
| rs60389750 | -0.0175 | 0.3139 | 1.06E-16 | T | C | 0.0021 | 59.400 |
| rs60408354 | 0.0259 | 0.0732 | 1.15E-12 | A | G | 0.0036 | 40.984 |
| rs604723 | -0.0166 | 0.7248 | 8.16E-15 | C | T | 0.0021 | 49.500 |
| rs6054390 | -0.0188 | 0.628 | 1.45E-21 | A | G | 0.002 | 74.364 |
| rs6054491 | -0.0142 | 0.2404 | 2.15E-10 | G | C | 0.0022 | 33.159 |
| rs6066122 | 0.0127 | 0.7631 | 1.76E-08 | G | C | 0.0023 | 26.258 |
| rs60804050 | -0.0217 | 0.2559 | 5.01E-24 | A | G | 0.0021 | 80.756 |
| rs6082354 | -0.024 | 0.6675 | 1.19E-32 | C | A | 0.002 | 115.147 |
| rs610694 | 0.0136 | 0.4903 | 4.29E-13 | C | T | 0.0019 | 41.626 |
| rs6142059 | 0.0116 | 0.4926 | 1.19E-09 | C | T | 0.0019 | 30.288 |
| rs61729527 | -0.0346 | 0.0519 | 4.86E-16 | T | C | 0.0043 | 53.052 |
| rs61732778 | 0.023 | 0.071 | 3.13E-10 | A | G | 0.0037 | 31.422 |
| rs61878760 | 0.019 | 0.0828 | 3.74E-08 | A | G | 0.0034 | 24.689 |
| rs61919240 | 0.0137 | 0.3244 | 9.77E-12 | A | T | 0.002 | 37.044 |
| rs61944841 | 0.0253 | 0.4138 | 3.54E-37 | A | G | 0.002 | 139.858 |
| rs62033029 | -0.0141 | 0.2064 | 1.73E-09 | A | G | 0.0023 | 29.326 |
| rs62103240 | 0.0212 | 0.071 | 1.40E-08 | A | G | 0.0037 | 26.696 |
| rs62106258 | -0.0504 | 0.0486 | 6.45E-31 | C | T | 0.0044 | 105.788 |
| rs62143873 | -0.0115 | 0.5033 | 1.19E-09 | A | G | 0.0019 | 29.773 |
| rs62370472 | -0.0253 | 0.209 | 1.45E-27 | C | T | 0.0023 | 95.308 |
| rs62466110 | -0.0371 | 0.067 | 5.74E-20 | C | T | 0.0041 | 77.491 |
| rs62501195 | -0.0198 | 0.1712 | 8.11E-15 | C | A | 0.0025 | 50.096 |
| rs62515437 | 0.0369 | 0.2253 | 8.79E-60 | T | G | 0.0023 | 214.106 |
| rs62621812 | 0.0743 | 0.0204 | 3.16E-27 | A | G | 0.0069 | 99.364 |
| rs6425817 | 0.0157 | 0.6724 | 2.98E-15 | G | A | 0.002 | 48.898 |
| rs6470771 | -0.0268 | 0.1694 | 1.54E-26 | C | A | 0.0025 | 91.020 |
| rs6502935 | -0.0125 | 0.7381 | 6.31E-09 | T | C | 0.0022 | 27.200 |
| rs650508 | -0.013 | 0.3008 | 1.86E-10 | C | G | 0.002 | 32.009 |
| rs6505216 | -0.0498 | 0.233 | 1.83E-101 | T | G | 0.0023 | 399.457 |
| rs6543146 | 0.0154 | 0.5579 | 4.19E-16 | G | T | 0.0019 | 52.680 |
| rs655113 | 0.0188 | 0.3006 | 7.42E-20 | C | T | 0.0021 | 66.922 |
| rs6570509 | -0.0244 | 0.2868 | 1.27E-31 | T | G | 0.0021 | 109.686 |
| rs6582398 | 0.014 | 0.6002 | 1.14E-12 | T | C | 0.002 | 42.356 |
| rs6593210 | 0.0146 | 0.2081 | 4.88E-10 | A | G | 0.0024 | 31.634 |
| rs664317 | -0.0177 | 0.8375 | 4.50E-12 | C | A | 0.0026 | 38.397 |
| rs6675858 | -0.0137 | 0.2141 | 2.40E-09 | T | C | 0.0023 | 28.440 |
| rs6693481 | -0.0143 | 0.6951 | 2.21E-12 | C | T | 0.002 | 39.029 |
| rs670318 | 0.0413 | 0.9515 | 2.52E-21 | C | T | 0.0044 | 70.891 |
| rs6721191 | -0.0144 | 0.5779 | 3.17E-14 | G | A | 0.0019 | 45.552 |
| rs6738207 | 0.0127 | 0.4007 | 4.14E-11 | A | G | 0.0019 | 34.880 |
| rs6739278 | -0.021 | 0.8079 | 1.27E-18 | C | T | 0.0024 | 61.639 |
| rs67527161 | -0.0182 | 0.209 | 5.79E-15 | C | T | 0.0023 | 49.316 |
| rs67551338 | 0.0576 | 0.0615 | 1.04E-47 | T | C | 0.004 | 172.503 |
| rs6762851 | -0.0218 | 0.3573 | 1.50E-28 | C | T | 0.002 | 98.293 |
| rs67716382 | 0.0226 | 0.2214 | 1.65E-23 | C | G | 0.0023 | 79.298 |
| rs68049170 | -0.0259 | 0.2764 | 2.69E-34 | A | G | 0.0021 | 120.845 |
| rs680882 | 0.0133 | 0.7606 | 1.98E-09 | G | T | 0.0022 | 29.006 |
| rs6821305 | 0.0204 | 0.399 | 3.15E-26 | C | A | 0.0019 | 89.881 |
| rs684905 | -0.0118 | 0.4176 | 7.10E-10 | T | C | 0.0019 | 30.497 |
| rs6860245 | 0.0589 | 0.248 | 9.66E-160 | C | G | 0.0022 | 583.361 |
| rs6874142 | 0.0288 | 0.1138 | 5.15E-20 | G | T | 0.0031 | 75.337 |
| rs6902109 | -0.0167 | 0.5401 | 1.03E-18 | G | A | 0.0019 | 62.389 |
| rs6931421 | -0.0279 | 0.3224 | 2.31E-43 | G | T | 0.002 | 153.179 |
| rs6977416 | 0.0457 | 0.3338 | 1.43E-113 | A | G | 0.002 | 418.603 |
| rs700677 | 0.0173 | 0.3505 | 1.13E-18 | A | C | 0.002 | 61.361 |
| rs7014590 | -0.0228 | 0.2614 | 4.48E-26 | C | T | 0.0022 | 90.395 |
| rs7020491 | -0.0178 | 0.4266 | 1.22E-20 | T | C | 0.0019 | 69.801 |
| rs702886 | 0.012 | 0.3505 | 1.11E-09 | G | A | 0.002 | 29.521 |
| rs704660 | 0.0153 | 0.4095 | 2.28E-15 | T | C | 0.0019 | 50.978 |
| rs7082659 | 0.0156 | 0.8663 | 2.27E-08 | C | T | 0.0028 | 25.383 |
| rs7095472 | 0.0267 | 0.5335 | 7.66E-45 | G | A | 0.0019 | 159.822 |
| rs7129320 | -0.0389 | 0.1661 | 7.29E-53 | A | G | 0.0025 | 188.816 |
| rs713467 | 0.0146 | 0.4345 | 3.09E-14 | A | G | 0.0019 | 47.168 |
| rs71414738 | 0.015 | 0.1763 | 1.00E-09 | T | C | 0.0025 | 29.424 |
| rs7144307 | -0.0122 | 0.3853 | 6.10E-10 | C | T | 0.002 | 31.746 |
| rs7185244 | -0.0148 | 0.7759 | 1.40E-10 | C | T | 0.0023 | 34.299 |
| rs718603 | 0.0131 | 0.2774 | 6.68E-10 | T | C | 0.0021 | 30.978 |
| rs7220127 | -0.0105 | 0.4264 | 4.60E-08 | C | T | 0.0019 | 24.283 |
| rs7228151 | -0.0185 | 0.206 | 3.19E-15 | C | T | 0.0023 | 50.414 |
| rs723149 | -0.0276 | 0.5628 | 1.43E-47 | G | A | 0.0019 | 168.846 |
| rs72656010 | -0.0668 | 0.1322 | 7.31E-126 | C | T | 0.0028 | 461.449 |
| rs72657800 | -0.0219 | 0.0773 | 6.39E-10 | C | T | 0.0035 | 30.806 |
| rs72695791 | -0.0297 | 0.036 | 4.58E-09 | G | C | 0.0051 | 27.567 |
| rs72721979 | -0.0229 | 0.143 | 2.20E-17 | G | T | 0.0027 | 57.879 |
| rs72726050 | -0.0192 | 0.0958 | 1.84E-08 | C | T | 0.0034 | 28.756 |
| rs72771070 | 0.015 | 0.283 | 1.30E-12 | T | C | 0.0021 | 41.115 |
| rs72801843 | 0.0313 | 0.3015 | 8.83E-52 | A | T | 0.0021 | 185.865 |
| rs72809820 | -0.0111 | 0.324 | 3.13E-08 | T | C | 0.002 | 24.302 |
| rs72829852 | 0.0309 | 0.0621 | 3.74E-15 | T | C | 0.0039 | 50.083 |
| rs7286917 | 0.0171 | 0.7461 | 5.18E-14 | G | A | 0.0023 | 49.886 |
| rs72894003 | -0.0423 | 0.0647 | 1.90E-28 | T | C | 0.0038 | 97.523 |
| rs73006226 | -0.0182 | 0.1267 | 2.23E-10 | A | C | 0.0029 | 33.006 |
| rs7301341 | -0.0255 | 0.3268 | 9.27E-37 | C | T | 0.002 | 128.856 |
| rs73052033 | -0.0151 | 0.1851 | 4.79E-10 | C | T | 0.0024 | 30.972 |
| rs73125634 | -0.0195 | 0.2782 | 5.11E-20 | T | G | 0.0021 | 68.768 |
| rs73197345 | 0.0211 | 0.1367 | 3.55E-14 | A | T | 0.0028 | 47.317 |
| rs7320878 | -0.015 | 0.6034 | 1.34E-14 | A | G | 0.0019 | 48.491 |
| rs7321635 | -0.0132 | 0.6465 | 2.58E-11 | C | A | 0.002 | 35.860 |
| rs7328187 | 0.0116 | 0.4976 | 1.19E-09 | G | T | 0.0019 | 30.294 |
| rs73384223 | -0.0205 | 0.1967 | 1.27E-17 | C | T | 0.0024 | 59.803 |
| rs73413540 | -0.0124 | 0.2248 | 4.42E-08 | T | C | 0.0023 | 24.130 |
| rs7367519 | 0.0164 | 0.6846 | 4.68E-16 | C | T | 0.002 | 52.301 |
| rs73696333 | 0.0191 | 0.2012 | 3.20E-15 | G | C | 0.0024 | 52.803 |
| rs73856768 | -0.0247 | 0.0805 | 1.55E-12 | C | T | 0.0035 | 40.668 |
| rs7418410 | 0.0155 | 0.4089 | 5.64E-16 | T | C | 0.0019 | 52.296 |
| rs74379684 | -0.0272 | 0.0751 | 4.39E-14 | T | C | 0.0036 | 46.280 |
| rs74458759 | 0.0171 | 0.2886 | 3.12E-15 | G | C | 0.0022 | 54.067 |
| rs74494415 | -0.0417 | 0.0398 | 1.82E-17 | T | C | 0.0049 | 59.848 |
| rs7485647 | -0.0261 | 0.844 | 1.04E-23 | A | G | 0.0026 | 80.779 |
| rs75022676 | -0.0163 | 0.2079 | 2.84E-12 | A | G | 0.0023 | 39.402 |
| rs7522400 | 0.0129 | 0.7683 | 5.62E-09 | G | A | 0.0022 | 26.677 |
| rs7543136 | -0.021 | 0.7209 | 9.96E-24 | T | C | 0.0021 | 79.914 |
| rs7543202 | 0.0129 | 0.6229 | 2.86E-11 | G | A | 0.0019 | 35.202 |
| rs75508358 | 0.0266 | 0.0457 | 4.29E-09 | T | C | 0.0045 | 27.789 |
| rs7563362 | 0.0352 | 0.8566 | 3.27E-39 | G | A | 0.0027 | 137.094 |
| rs7570235 | -0.0168 | 0.5911 | 2.08E-18 | C | T | 0.0019 | 61.437 |
| rs75702986 | -0.0163 | 0.1861 | 3.14E-11 | A | G | 0.0025 | 36.241 |
| rs7598430 | -0.016 | 0.5046 | 1.37E-17 | T | C | 0.0019 | 57.633 |
| rs7610055 | -0.0373 | 0.1207 | 3.55E-38 | A | G | 0.0029 | 133.004 |
| rs7633464 | 0.0175 | 0.4786 | 1.27E-20 | A | G | 0.0019 | 68.827 |
| rs76364830 | -0.0471 | 0.0634 | 2.70E-33 | A | G | 0.0039 | 118.652 |
| rs76517946 | -0.0368 | 0.0814 | 1.69E-26 | A | C | 0.0035 | 91.203 |
| rs7679276 | -0.033 | 0.9544 | 5.93E-12 | G | A | 0.0048 | 42.681 |
| rs7689420 | 0.0466 | 0.8312 | 1.50E-76 | C | T | 0.0025 | 274.530 |
| rs76895963 | 0.1639 | 0.0207 | 8.22E-112 | G | T | 0.0073 | 490.899 |
| rs7701233 | -0.0179 | 0.4283 | 5.12E-21 | C | T | 0.0019 | 70.659 |
| rs77013652 | 0.049 | 0.015 | 1.50E-09 | G | T | 0.0081 | 31.947 |
| rs772222 | 0.0121 | 0.2651 | 1.55E-08 | G | A | 0.0021 | 25.687 |
| rs7731023 | 0.0166 | 0.5749 | 3.48E-18 | G | A | 0.0019 | 60.650 |
| rs7735891 | 0.0259 | 0.4629 | 1.14E-42 | T | C | 0.0019 | 150.232 |
| rs77364196 | -0.033 | 0.053 | 8.35E-15 | A | G | 0.0043 | 49.224 |
| rs77447813 | 0.0224 | 0.087 | 3.21E-11 | C | G | 0.0034 | 35.892 |
| rs7768382 | -0.0201 | 0.4771 | 1.57E-26 | C | T | 0.0019 | 90.778 |
| rs77809369 | 0.0237 | 0.0642 | 9.83E-10 | T | C | 0.0039 | 30.389 |
| rs78051210 | 0.0263 | 0.0771 | 1.63E-13 | C | T | 0.0036 | 44.324 |
| rs7816345 | 0.0255 | 0.1682 | 6.22E-24 | T | C | 0.0025 | 81.937 |
| rs781669 | 0.0164 | 0.5236 | 3.14E-18 | T | C | 0.0019 | 60.422 |
| rs7826059 | 0.0114 | 0.6421 | 7.61E-09 | C | T | 0.002 | 26.895 |
| rs7828086 | 0.0135 | 0.2391 | 1.11E-09 | C | T | 0.0022 | 29.859 |
| rs78378222 | 0.138 | 0.0123 | 4.51E-56 | G | T | 0.0087 | 208.432 |
| rs78457529 | -0.0904 | 0.0117 | 1.22E-24 | T | C | 0.0088 | 85.108 |
| rs7858712 | 0.0347 | 0.9144 | 1.04E-24 | G | A | 0.0034 | 84.884 |
| rs78766798 | 0.0319 | 0.0847 | 2.61E-20 | C | T | 0.0035 | 71.051 |
| rs7893378 | 0.0175 | 0.1143 | 2.36E-08 | A | G | 0.0031 | 27.920 |
| rs7902 | 0.0149 | 0.4469 | 5.22E-15 | G | A | 0.0019 | 49.421 |
| rs79441499 | -0.0138 | 0.4055 | 9.67E-13 | T | C | 0.0019 | 41.344 |
| rs7952436 | -0.0453 | 0.0823 | 1.62E-39 | T | C | 0.0034 | 139.607 |
| rs798548 | -0.0359 | 0.3006 | 2.86E-68 | C | T | 0.0021 | 244.126 |
| rs8000973 | 0.0134 | 0.5334 | 2.05E-12 | C | T | 0.0019 | 40.246 |
| rs80132799 | 0.0231 | 0.0679 | 1.31E-09 | T | C | 0.0038 | 30.413 |
| rs8017006 | 0.0122 | 0.3275 | 2.22E-09 | G | A | 0.002 | 29.521 |
| rs8018486 | -0.0138 | 0.1914 | 1.18E-08 | G | A | 0.0024 | 26.542 |
| rs8019890 | 0.025 | 0.5313 | 1.96E-38 | A | C | 0.0019 | 140.193 |
| rs8020095 | -0.0145 | 0.15 | 4.53E-08 | A | G | 0.0027 | 24.140 |
| rs80280630 | -0.0168 | 0.1115 | 2.28E-08 | T | C | 0.003 | 25.180 |
| rs80295797 | -0.0198 | 0.3265 | 3.83E-23 | T | C | 0.002 | 77.643 |
| rs8042578 | 0.0287 | 0.2426 | 2.29E-38 | G | C | 0.0022 | 136.328 |
| rs8054549 | -0.0251 | 0.4486 | 3.37E-39 | A | C | 0.0019 | 140.373 |
| rs8084413 | -0.0127 | 0.4693 | 3.25E-11 | A | G | 0.0019 | 36.176 |
| rs8136517 | 0.0267 | 0.0652 | 6.58E-12 | C | T | 0.0039 | 39.129 |
| rs822530 | 0.0255 | 0.7953 | 2.36E-27 | T | A | 0.0024 | 95.345 |
| rs839255 | -0.0126 | 0.6847 | 8.38E-10 | G | T | 0.0021 | 30.865 |
| rs867529 | 0.0184 | 0.2796 | 1.00E-18 | C | G | 0.0021 | 61.416 |
| rs876122 | 0.0162 | 0.8788 | 2.19E-08 | G | A | 0.0029 | 25.172 |
| rs8904 | -0.0157 | 0.363 | 1.52E-15 | A | G | 0.002 | 51.330 |
| rs900399 | 0.0164 | 0.3986 | 1.35E-17 | G | A | 0.0019 | 58.066 |
| rs905938 | 0.0394 | 0.2649 | 8.43E-77 | C | T | 0.0021 | 272.370 |
| rs909220 | -0.015 | 0.4443 | 3.24E-15 | A | G | 0.0019 | 50.029 |
| rs9266244 | -0.0427 | 0.7077 | 1.21E-94 | A | G | 0.0021 | 339.889 |
| rs9344126 | -0.0185 | 0.5137 | 2.21E-22 | C | T | 0.0019 | 77.003 |
| rs9375188 | 0.0136 | 0.4844 | 6.80E-13 | T | C | 0.0019 | 41.602 |
| rs9385002 | -0.0147 | 0.2394 | 3.26E-11 | T | A | 0.0022 | 35.434 |
| rs9388490 | 0.0462 | 0.4394 | 1.33E-130 | T | C | 0.0019 | 473.946 |
| rs947099 | 0.0117 | 0.3536 | 2.71E-09 | A | G | 0.002 | 28.177 |
| rs951366 | 0.0205 | 0.3932 | 9.15E-27 | C | T | 0.0019 | 90.309 |
| rs9517483 | -0.0181 | 0.699 | 2.26E-18 | G | A | 0.0021 | 62.078 |
| rs9525326 | -0.0184 | 0.1875 | 3.62E-14 | G | A | 0.0024 | 46.449 |
| rs9568031 | -0.0115 | 0.706 | 3.34E-08 | T | C | 0.0021 | 24.720 |
| rs9590328 | 0.0153 | 0.1417 | 2.02E-08 | G | A | 0.0027 | 25.638 |
| rs9594714 | 0.0144 | 0.3052 | 2.65E-12 | T | G | 0.0021 | 39.599 |
| rs9610447 | 0.0152 | 0.7471 | 5.29E-12 | T | C | 0.0022 | 39.312 |
| rs9634212 | 0.0471 | 0.221 | 8.59E-95 | A | C | 0.0023 | 344.174 |
| rs9636364 | 0.011 | 0.5429 | 5.09E-09 | A | G | 0.0019 | 27.041 |
| rs9647379 | 0.0215 | 0.4111 | 5.55E-29 | C | G | 0.0019 | 100.795 |
| rs9669278 | -0.0496 | 0.5183 | 5.25E-151 | C | T | 0.0019 | 553.771 |
| rs9809116 | -0.016 | 0.4081 | 1.31E-16 | G | A | 0.0019 | 55.691 |
| rs9828525 | 0.0121 | 0.4113 | 2.51E-10 | T | C | 0.0019 | 31.925 |
| rs9832919 | -0.0179 | 0.3574 | 7.90E-20 | G | A | 0.002 | 66.274 |
| rs9838614 | -0.0185 | 0.3881 | 1.21E-21 | G | T | 0.0019 | 73.200 |
| rs987666 | 0.0185 | 0.1188 | 2.33E-10 | A | G | 0.0029 | 32.266 |
| rs9890062 | 0.0267 | 0.0615 | 1.20E-11 | A | G | 0.0039 | 37.055 |
| rs9894577 | -0.031 | 0.318 | 1.40E-52 | A | G | 0.002 | 187.755 |
| rs9898189 | -0.0163 | 0.6446 | 1.88E-15 | G | C | 0.0021 | 54.816 |
| rs990315 | -0.0115 | 0.6229 | 5.07E-09 | C | T | 0.002 | 27.975 |
| rs9905385 | -0.0339 | 0.6707 | 1.94E-63 | G | A | 0.002 | 228.673 |
| rs9957318 | 0.0187 | 0.348 | 1.02E-20 | G | A | 0.002 | 71.459 |

SNP, single nucleotide polymorphism; Beta coefficients are in standard deviation (SD) unit per allele; EAF, effect allele frequency; SE, standard error;

**Supplementary Table 7.** Characteristics of selected SNPs concerning the causal effect of ALM on stroke

| **SNP** | **Beta** | **EAF** | ***P*-value** | **Effect**  **allele** | **Other**  **allele** | **SE** | ***F*-value** |
| --- | --- | --- | --- | --- | --- | --- | --- |
| rs10005035 | -0.0175 | 0.2823 | 6.51E-17 | G | C | 0.0021 | 55.880 |
| rs10019221 | -0.0124 | 0.5974 | 1.22E-10 | T | G | 0.0019 | 33.303 |
| rs1005723 | 0.0161 | 0.1908 | 1.79E-11 | T | C | 0.0024 | 36.041 |
| rs10068640 | 0.0112 | 0.3651 | 1.28E-08 | A | G | 0.002 | 26.185 |
| rs10075249 | 0.0143 | 0.4952 | 4.56E-14 | T | C | 0.0019 | 46.035 |
| rs10107388 | -0.0159 | 0.3691 | 6.95E-16 | C | T | 0.002 | 53.018 |
| rs10112506 | -0.012 | 0.3898 | 5.76E-10 | G | A | 0.0019 | 30.845 |
| rs10123619 | -0.0171 | 0.8425 | 4.05E-11 | G | A | 0.0026 | 34.942 |
| rs10128333 | -0.0146 | 0.1676 | 9.51E-09 | T | C | 0.0025 | 26.780 |
| rs10202701 | 0.0227 | 0.5419 | 3.11E-33 | T | C | 0.0019 | 115.217 |
| rs10202845 | -0.0288 | 0.1133 | 5.35E-22 | G | A | 0.003 | 75.048 |
| rs10203320 | 0.0138 | 0.3289 | 7.86E-12 | C | T | 0.002 | 37.855 |
| rs10205141 | 0.0241 | 0.0476 | 4.71E-08 | G | A | 0.0044 | 23.711 |
| rs10221831 | 0.03 | 0.0321 | 1.80E-08 | T | C | 0.0053 | 25.181 |
| rs10225945 | -0.0146 | 0.1499 | 3.28E-08 | G | A | 0.0026 | 24.461 |
| rs10242866 | 0.0157 | 0.3983 | 3.66E-16 | T | C | 0.0019 | 53.201 |
| rs10283100 | 0.0575 | 0.9445 | 4.11E-44 | G | A | 0.0041 | 156.119 |
| rs1035583 | 0.0148 | 0.6177 | 1.99E-14 | A | G | 0.0019 | 46.583 |
| rs10453441 | -0.0139 | 0.402 | 9.10E-13 | G | A | 0.002 | 41.828 |
| rs10461725 | 0.0134 | 0.6561 | 1.98E-11 | C | G | 0.002 | 36.486 |
| rs10471339 | -0.011 | 0.3823 | 1.45E-08 | G | C | 0.0019 | 25.732 |
| rs1047891 | 0.0233 | 0.3159 | 5.70E-31 | A | C | 0.002 | 105.672 |
| rs10483727 | -0.0368 | 0.6109 | 6.73E-80 | C | T | 0.0019 | 290.056 |
| rs1056747 | -0.0155 | 0.4117 | 8.05E-16 | G | A | 0.0019 | 52.405 |
| rs1063582 | -0.0185 | 0.7649 | 1.12E-16 | G | T | 0.0022 | 55.428 |
| rs10748128 | 0.0255 | 0.3446 | 6.77E-38 | T | G | 0.002 | 132.283 |
| rs10749157 | 0.0113 | 0.3584 | 1.24E-08 | C | T | 0.002 | 26.442 |
| rs10776560 | -0.0157 | 0.4995 | 7.88E-17 | T | C | 0.0019 | 55.497 |
| rs10793931 | -0.0132 | 0.3555 | 3.23E-11 | C | G | 0.002 | 35.952 |
| rs10796828 | 0.0154 | 0.6348 | 5.77E-15 | G | T | 0.002 | 49.514 |
| rs10815274 | 0.0124 | 0.4562 | 6.46E-11 | C | A | 0.0019 | 34.352 |
| rs10822117 | -0.0176 | 0.237 | 4.24E-15 | G | A | 0.0022 | 50.445 |
| rs10824307 | -0.0194 | 0.6471 | 1.79E-22 | C | G | 0.002 | 77.406 |
| rs10829226 | -0.0112 | 0.6359 | 1.33E-08 | A | G | 0.002 | 26.154 |
| rs10832963 | -0.0203 | 0.7445 | 9.23E-21 | G | T | 0.0022 | 70.598 |
| rs10845408 | 0.0255 | 0.3538 | 3.25E-38 | T | C | 0.002 | 133.909 |
| rs10858246 | -0.0188 | 0.3183 | 2.35E-20 | C | G | 0.002 | 69.070 |
| rs10864899 | -0.0112 | 0.5643 | 3.85E-09 | G | A | 0.0019 | 27.774 |
| rs10922475 | 0.0159 | 0.5395 | 2.17E-17 | A | C | 0.0019 | 56.565 |
| rs10975935 | -0.0121 | 0.2464 | 4.15E-08 | G | A | 0.0022 | 24.482 |
| rs10982888 | -0.0328 | 0.1136 | 3.97E-28 | A | T | 0.003 | 97.572 |
| rs11014285 | 0.0342 | 0.1654 | 2.89E-40 | A | G | 0.0026 | 145.439 |
| rs11042717 | -0.029 | 0.4896 | 4.04E-53 | C | T | 0.0019 | 189.324 |
| rs11049704 | -0.0183 | 0.293 | 1.10E-18 | G | C | 0.0021 | 62.478 |
| rs11060942 | -0.0354 | 0.0344 | 7.49E-12 | A | G | 0.0052 | 37.486 |
| rs11068230 | 0.0238 | 0.8656 | 9.67E-18 | G | C | 0.0028 | 59.348 |
| rs11070842 | -0.0146 | 0.1631 | 1.30E-08 | C | T | 0.0026 | 26.202 |
| rs11098677 | -0.0263 | 0.7875 | 3.94E-30 | T | G | 0.0023 | 104.255 |
| rs11121615 | -0.0202 | 0.6894 | 3.32E-23 | T | C | 0.002 | 78.691 |
| rs111365325 | -0.0271 | 0.2312 | 1.12E-33 | T | C | 0.0022 | 117.579 |
| rs111622870 | -0.0282 | 0.0482 | 1.86E-10 | C | T | 0.0044 | 32.855 |
| rs11175919 | 0.0349 | 0.0262 | 3.16E-09 | A | G | 0.0059 | 27.985 |
| rs11187838 | 0.0394 | 0.4347 | 1.16E-94 | A | G | 0.0019 | 343.770 |
| rs11191208 | 0.0147 | 0.2057 | 3.69E-10 | A | G | 0.0024 | 31.795 |
| rs11198591 | 0.0148 | 0.3684 | 4.91E-14 | A | G | 0.002 | 45.899 |
| rs112021215 | -0.0147 | 0.201 | 5.99E-09 | C | T | 0.0025 | 31.252 |
| rs11210892 | 0.0118 | 0.6745 | 3.57E-09 | A | G | 0.002 | 27.530 |
| rs112153300 | 0.0261 | 0.089 | 7.05E-15 | A | G | 0.0034 | 49.741 |
| rs11217863 | -0.0268 | 0.1162 | 1.06E-19 | A | G | 0.003 | 66.431 |
| rs11243202 | 0.0302 | 0.486 | 2.83E-57 | C | T | 0.0019 | 205.251 |
| rs112537273 | -0.0212 | 0.2299 | 3.34E-21 | C | T | 0.0022 | 71.664 |
| rs11260035 | 0.015 | 0.2761 | 1.86E-12 | A | G | 0.0021 | 40.499 |
| rs11260623 | 0.0117 | 0.5085 | 4.86E-10 | T | G | 0.0019 | 30.810 |
| rs112873218 | 0.0216 | 0.1054 | 4.02E-12 | T | C | 0.0031 | 39.618 |
| rs113146332 | 0.0311 | 0.0381 | 2.99E-10 | C | G | 0.0049 | 31.921 |
| rs113232639 | 0.0327 | 0.493 | 4.79E-64 | A | G | 0.0019 | 240.801 |
| rs113289555 | -0.0206 | 0.2333 | 7.33E-20 | T | G | 0.0023 | 68.362 |
| rs113671109 | -0.015 | 0.22 | 4.23E-11 | C | T | 0.0023 | 34.770 |
| rs113827862 | -0.0235 | 0.0608 | 4.67E-09 | C | T | 0.004 | 28.399 |
| rs113898003 | -0.036 | 0.2635 | 1.19E-63 | C | T | 0.0021 | 226.596 |
| rs11580040 | 0.0325 | 0.08 | 6.76E-21 | G | A | 0.0035 | 70.014 |
| rs11590254 | 0.0186 | 0.3114 | 4.34E-20 | T | A | 0.002 | 66.811 |
| rs115912456 | 0.0577 | 0.0412 | 3.69E-34 | G | A | 0.0047 | 118.458 |
| rs116008080 | -0.0415 | 0.0238 | 4.06E-11 | A | G | 0.0063 | 36.035 |
| rs116052377 | 0.0225 | 0.0809 | 7.89E-11 | A | G | 0.0035 | 33.899 |
| rs11605297 | 0.0146 | 0.2328 | 8.03E-11 | A | G | 0.0022 | 34.285 |
| rs116092985 | -0.0401 | 0.0958 | 1.17E-34 | G | A | 0.0033 | 125.463 |
| rs11612462 | 0.015 | 0.1698 | 2.55E-09 | G | T | 0.0025 | 28.563 |
| rs11629593 | -0.0109 | 0.6158 | 4.42E-08 | G | T | 0.002 | 25.313 |
| rs11633371 | 0.0216 | 0.4764 | 7.49E-30 | T | G | 0.0019 | 104.823 |
| rs116339650 | -0.0175 | 0.127 | 1.05E-09 | G | A | 0.0029 | 30.577 |
| rs116493405 | 0.0287 | 0.0537 | 9.52E-12 | A | G | 0.0042 | 37.695 |
| rs11672848 | -0.0171 | 0.5246 | 7.73E-19 | T | C | 0.0019 | 65.678 |
| rs1168768 | 0.0332 | 0.975 | 3.61E-08 | T | C | 0.006 | 24.195 |
| rs116919274 | 0.0271 | 0.046 | 3.52E-09 | A | G | 0.0046 | 29.023 |
| rs117068593 | 0.0403 | 0.1895 | 8.83E-62 | T | C | 0.0024 | 224.732 |
| rs117203652 | -0.0346 | 0.03 | 4.32E-10 | A | G | 0.0055 | 31.373 |
| rs11720869 | 0.0141 | 0.6686 | 2.54E-12 | A | G | 0.002 | 39.671 |
| rs11721522 | 0.0106 | 0.4125 | 4.03E-08 | G | A | 0.0019 | 24.521 |
| rs11727162 | -0.017 | 0.4987 | 2.15E-19 | T | C | 0.0019 | 65.069 |
| rs117335233 | -0.0236 | 0.053 | 2.56E-08 | G | T | 0.0042 | 25.174 |
| rs1177765 | -0.0232 | 0.4681 | 1.32E-34 | C | T | 0.0019 | 120.708 |
| rs11778491 | -0.0247 | 0.2518 | 8.39E-30 | C | G | 0.0022 | 103.524 |
| rs117972846 | 0.0335 | 0.0285 | 5.47E-09 | G | T | 0.0057 | 27.982 |
| rs11867855 | -0.0132 | 0.2372 | 2.93E-09 | T | G | 0.0022 | 28.391 |
| rs1190540 | 0.0125 | 0.6983 | 1.62E-09 | G | A | 0.0021 | 29.644 |
| rs11959466 | 0.038 | 0.0564 | 2.24E-19 | T | C | 0.0042 | 69.211 |
| rs1202186 | -0.012 | 0.6548 | 1.65E-09 | T | C | 0.002 | 29.312 |
| rs12051245 | 0.0299 | 0.2318 | 2.56E-40 | C | T | 0.0022 | 143.398 |
| rs12074850 | 0.0393 | 0.0898 | 2.72E-33 | G | A | 0.0033 | 113.706 |
| rs12099669 | 0.0331 | 0.6962 | 1.39E-58 | A | G | 0.002 | 208.763 |
| rs12150907 | -0.0219 | 0.1977 | 7.22E-20 | A | G | 0.0024 | 68.513 |
| rs12185775 | -0.0167 | 0.1172 | 3.66E-08 | C | G | 0.003 | 25.985 |
| rs12188208 | -0.0195 | 0.2349 | 1.40E-18 | C | A | 0.0022 | 61.547 |
| rs12230946 | 0.0271 | 0.0911 | 1.45E-16 | A | G | 0.0033 | 54.765 |
| rs12340775 | -0.0287 | 0.0552 | 1.73E-11 | A | G | 0.0043 | 38.686 |
| rs12344515 | -0.0163 | 0.2402 | 2.28E-13 | T | C | 0.0022 | 43.668 |
| rs12347137 | -0.046 | 0.2023 | 9.80E-85 | C | A | 0.0024 | 307.697 |
| rs12351226 | 0.0218 | 0.1712 | 9.13E-18 | T | C | 0.0025 | 60.730 |
| rs12423821 | 0.0161 | 0.1575 | 1.17E-09 | C | T | 0.0027 | 30.975 |
| rs12461874 | -0.0181 | 0.2784 | 1.26E-17 | A | C | 0.0021 | 59.273 |
| rs12483401 | -0.0387 | 0.0216 | 9.22E-09 | C | T | 0.0067 | 28.503 |
| rs12512942 | -0.0162 | 0.6376 | 1.58E-16 | A | G | 0.002 | 54.613 |
| rs12517711 | -0.0147 | 0.3917 | 2.79E-14 | C | T | 0.0019 | 46.369 |
| rs12519407 | 0.0181 | 0.2567 | 3.38E-17 | C | A | 0.0022 | 56.296 |
| rs12533452 | 0.0237 | 0.1569 | 1.31E-19 | T | C | 0.0026 | 66.917 |
| rs12541381 | -0.0319 | 0.2575 | 2.81E-49 | A | G | 0.0022 | 175.267 |
| rs12563442 | 0.0122 | 0.2669 | 9.93E-09 | C | T | 0.0021 | 26.226 |
| rs1260326 | 0.0323 | 0.6045 | 6.16E-64 | C | T | 0.0019 | 224.719 |
| rs12616192 | -0.0261 | 0.0676 | 6.83E-12 | A | G | 0.0038 | 38.667 |
| rs12655296 | -0.011 | 0.6251 | 1.62E-08 | T | C | 0.002 | 25.536 |
| rs12672217 | 0.0139 | 0.3609 | 1.33E-12 | A | G | 0.002 | 40.133 |
| rs12700901 | -0.0184 | 0.4055 | 1.67E-21 | A | C | 0.0019 | 73.506 |
| rs12702693 | 0.0173 | 0.4542 | 6.56E-20 | T | C | 0.0019 | 66.821 |
| rs12713004 | 0.0367 | 0.7254 | 2.40E-68 | G | A | 0.0021 | 241.723 |
| rs12724708 | 0.0243 | 0.3574 | 1.66E-35 | T | A | 0.002 | 122.152 |
| rs12773500 | 0.0171 | 0.1375 | 5.05E-10 | T | C | 0.0028 | 31.229 |
| rs12831751 | 0.0172 | 0.2857 | 1.57E-16 | C | A | 0.0021 | 54.372 |
| rs12882130 | -0.0202 | 0.3836 | 1.88E-24 | G | C | 0.002 | 86.897 |
| rs12907139 | -0.0149 | 0.5245 | 4.89E-15 | A | G | 0.0019 | 49.865 |
| rs1290786 | -0.0143 | 0.4307 | 7.14E-14 | T | C | 0.0019 | 45.155 |
| rs12909863 | 0.0189 | 0.2512 | 6.05E-18 | C | G | 0.0022 | 60.512 |
| rs12926103 | 0.0272 | 0.0661 | 9.60E-13 | A | G | 0.0038 | 41.130 |
| rs12943867 | 0.0184 | 0.3367 | 7.57E-20 | A | G | 0.002 | 68.097 |
| rs12962050 | 0.0153 | 0.6445 | 1.52E-14 | A | G | 0.002 | 48.302 |
| rs12997625 | -0.017 | 0.5266 | 1.50E-19 | T | C | 0.0019 | 64.885 |
| rs13037813 | 0.0292 | 0.2391 | 1.71E-39 | C | T | 0.0022 | 139.728 |
| rs13103161 | -0.0284 | 0.3891 | 2.38E-48 | A | T | 0.0019 | 172.707 |
| rs13109280 | 0.0131 | 0.6618 | 9.15E-11 | G | A | 0.002 | 34.590 |
| rs13123591 | 0.0185 | 0.3375 | 2.35E-20 | G | T | 0.002 | 68.920 |
| rs13127468 | -0.0123 | 0.4629 | 9.86E-11 | A | C | 0.0019 | 33.874 |
| rs13170063 | -0.0152 | 0.5921 | 4.11E-15 | A | G | 0.0019 | 50.253 |
| rs1319012 | -0.052 | 0.9259 | 3.30E-45 | A | T | 0.0037 | 167.119 |
| rs13209685 | 0.0277 | 0.1594 | 7.49E-27 | T | G | 0.0026 | 92.598 |
| rs1324538 | 0.0237 | 0.3836 | 1.73E-34 | A | T | 0.0019 | 119.627 |
| rs1325596 | 0.0287 | 0.5476 | 2.77E-52 | A | G | 0.0019 | 183.824 |
| rs1330826 | 0.0162 | 0.2268 | 1.04E-12 | C | G | 0.0023 | 41.446 |
| rs13316 | 0.0115 | 0.4301 | 3.66E-09 | A | C | 0.0019 | 29.192 |
| rs13391980 | -0.0225 | 0.1198 | 7.60E-15 | A | G | 0.0029 | 48.076 |
| rs1340022 | 0.0118 | 0.4868 | 4.47E-10 | C | T | 0.0019 | 31.326 |
| rs1341215 | 0.0229 | 0.1374 | 6.32E-17 | A | G | 0.0027 | 55.975 |
| rs13430869 | 0.0272 | 0.7427 | 6.37E-37 | T | G | 0.0021 | 127.347 |
| rs139163241 | -0.0164 | 0.1394 | 1.79E-09 | G | T | 0.0027 | 29.057 |
| rs140440099 | 0.0613 | 0.0234 | 1.45E-22 | A | G | 0.0063 | 77.340 |
| rs1405227 | 0.0129 | 0.3167 | 1.57E-10 | A | G | 0.002 | 32.430 |
| rs143554698 | -0.0257 | 0.1409 | 3.39E-21 | T | C | 0.0027 | 72.005 |
| rs144109601 | -0.0278 | 0.0418 | 5.15E-09 | A | C | 0.0048 | 27.876 |
| rs1443536 | 0.0218 | 0.305 | 1.91E-26 | G | A | 0.0021 | 90.732 |
| rs1444628 | 0.024 | 0.6904 | 6.85E-32 | T | C | 0.002 | 110.894 |
| rs144627572 | 0.0439 | 0.0329 | 1.30E-16 | A | G | 0.0053 | 55.224 |
| rs147233090 | -0.0446 | 0.0247 | 3.95E-13 | T | C | 0.0061 | 43.154 |
| rs1472852 | -0.0638 | 0.1582 | 8.22E-135 | A | C | 0.0026 | 488.656 |
| rs1478575 | 0.0312 | 0.6843 | 5.20E-54 | A | T | 0.002 | 189.447 |
| rs14976 | 0.0144 | 0.3057 | 1.43E-12 | T | C | 0.002 | 39.635 |
| rs1514134 | -0.0114 | 0.3849 | 3.57E-09 | C | T | 0.0019 | 27.708 |
| rs1556659 | 0.0163 | 0.3818 | 7.19E-17 | T | C | 0.002 | 56.477 |
| rs1584011 | 0.0159 | 0.3559 | 9.78E-16 | G | T | 0.002 | 52.192 |
| rs165849 | 0.0157 | 0.6975 | 4.57E-14 | A | G | 0.0021 | 46.837 |
| rs16989695 | -0.0139 | 0.5165 | 1.93E-13 | A | G | 0.0019 | 43.452 |
| rs17197114 | 0.0177 | 0.1772 | 1.54E-12 | C | T | 0.0025 | 41.136 |
| rs17205463 | -0.0263 | 0.4477 | 4.21E-43 | T | C | 0.0019 | 154.063 |
| rs17246129 | 0.0254 | 0.3044 | 1.27E-35 | A | G | 0.002 | 123.045 |
| rs17278379 | 0.0226 | 0.124 | 2.40E-15 | C | T | 0.0029 | 49.965 |
| rs1730028 | 0.0131 | 0.4174 | 7.39E-12 | G | T | 0.0019 | 37.582 |
| rs173135 | -0.0341 | 0.115 | 3.25E-30 | T | C | 0.003 | 106.593 |
| rs17400325 | 0.0345 | 0.0415 | 2.10E-13 | C | T | 0.0047 | 42.638 |
| rs17478946 | -0.0192 | 0.3004 | 9.98E-21 | G | A | 0.0021 | 69.774 |
| rs17681189 | -0.0131 | 0.4231 | 5.82E-12 | A | C | 0.0019 | 37.722 |
| rs17718736 | 0.0115 | 0.3226 | 1.39E-08 | A | C | 0.002 | 26.026 |
| rs177591 | -0.0191 | 0.1489 | 1.51E-12 | G | C | 0.0027 | 41.635 |
| rs17773965 | -0.0163 | 0.1412 | 1.51E-09 | T | C | 0.0027 | 29.014 |
| rs17818592 | -0.0129 | 0.4367 | 1.23E-11 | C | T | 0.0019 | 36.865 |
| rs1786263 | -0.019 | 0.6055 | 1.03E-22 | T | G | 0.0019 | 77.664 |
| rs1797070 | 0.0219 | 0.2684 | 4.93E-25 | A | G | 0.0021 | 84.821 |
| rs1823217 | -0.0181 | 0.6449 | 4.06E-20 | G | A | 0.002 | 67.568 |
| rs1880318 | 0.0147 | 0.2039 | 6.86E-10 | A | G | 0.0024 | 31.588 |
| rs1899040 | 0.0152 | 0.7962 | 9.04E-11 | T | C | 0.0023 | 33.761 |
| rs190823861 | -0.0345 | 0.0466 | 2.09E-14 | A | G | 0.0045 | 47.623 |
| rs1933081 | 0.0267 | 0.0834 | 5.33E-15 | A | T | 0.0034 | 49.078 |
| rs200439 | -0.0128 | 0.2207 | 1.51E-08 | C | A | 0.0023 | 25.376 |
| rs2005172 | 0.048 | 0.6397 | 2.35E-128 | C | A | 0.002 | 478.696 |
| rs2019203 | 0.0189 | 0.4909 | 1.84E-23 | A | C | 0.0019 | 80.403 |
| rs2025609 | 0.0186 | 0.8511 | 2.00E-12 | G | C | 0.0026 | 39.483 |
| rs2025808 | 0.0122 | 0.2541 | 1.72E-08 | A | C | 0.0022 | 25.404 |
| rs2035901 | 0.024 | 0.4679 | 9.43E-37 | G | A | 0.0019 | 129.172 |
| rs2070598 | 0.0204 | 0.4559 | 6.36E-27 | A | G | 0.0019 | 92.977 |
| rs2071450 | -0.0174 | 0.3675 | 8.85E-19 | T | C | 0.002 | 63.380 |
| rs2089111 | -0.0172 | 0.267 | 1.73E-15 | G | C | 0.0022 | 52.143 |
| rs2101017 | -0.0223 | 0.8695 | 1.44E-15 | T | C | 0.0028 | 50.818 |
| rs2105333 | -0.019 | 0.6648 | 1.70E-21 | G | T | 0.002 | 72.451 |
| rs2112617 | -0.0167 | 0.5294 | 1.07E-18 | A | G | 0.0019 | 62.575 |
| rs212526 | 0.0214 | 0.6007 | 3.84E-29 | C | T | 0.0019 | 98.936 |
| rs2138374 | -0.0149 | 0.696 | 2.79E-13 | C | T | 0.002 | 42.303 |
| rs2140619 | 0.0113 | 0.4128 | 5.18E-09 | G | A | 0.0019 | 27.873 |
| rs2142331 | -0.0165 | 0.6023 | 1.38E-17 | T | C | 0.0019 | 58.731 |
| rs2142644 | -0.0181 | 0.672 | 3.37E-19 | A | C | 0.002 | 65.034 |
| rs2181834 | 0.0254 | 0.5503 | 7.69E-41 | T | G | 0.0019 | 143.815 |
| rs2188805 | 0.0114 | 0.3359 | 2.00E-08 | C | A | 0.002 | 26.107 |
| rs2194411 | 0.0443 | 0.1283 | 2.43E-54 | A | G | 0.0029 | 197.727 |
| rs2209098 | 0.024 | 0.3108 | 1.73E-32 | C | T | 0.002 | 111.130 |
| rs2212926 | -0.022 | 0.2106 | 7.75E-21 | A | C | 0.0023 | 72.468 |
| rs2229840 | 0.0341 | 0.1597 | 3.02E-40 | T | C | 0.0026 | 140.559 |
| rs2230033 | -0.0265 | 0.5644 | 3.49E-43 | A | G | 0.0019 | 155.522 |
| rs2236096 | 0.018 | 0.2327 | 1.29E-15 | C | T | 0.0023 | 52.099 |
| rs2236406 | 0.0394 | 0.3493 | 1.26E-87 | C | T | 0.002 | 317.946 |
| rs2237485 | 0.0191 | 0.2234 | 3.73E-17 | A | G | 0.0023 | 57.000 |
| rs2240735 | 0.0189 | 0.748 | 3.99E-18 | T | C | 0.0022 | 60.640 |
| rs2268718 | 0.0141 | 0.2704 | 3.24E-11 | T | C | 0.0021 | 35.321 |
| rs2270894 | -0.0332 | 0.2034 | 1.25E-42 | G | C | 0.0024 | 160.878 |
| rs2274351 | 0.017 | 0.5433 | 3.07E-19 | T | C | 0.0019 | 64.581 |
| rs2283200 | -0.0281 | 0.0559 | 1.48E-11 | T | C | 0.0042 | 37.528 |
| rs2289629 | -0.0148 | 0.3451 | 8.02E-14 | A | G | 0.002 | 44.582 |
| rs2296316 | -0.0192 | 0.4643 | 1.59E-23 | C | T | 0.0019 | 82.580 |
| rs2303423 | 0.0168 | 0.1095 | 2.64E-08 | C | T | 0.003 | 24.784 |
| rs2305141 | 0.0183 | 0.5961 | 1.07E-21 | G | A | 0.0019 | 72.617 |
| rs2324154 | 0.015 | 0.5096 | 1.92E-15 | A | C | 0.0019 | 50.639 |
| rs234640 | -0.0131 | 0.5142 | 3.87E-12 | T | C | 0.0019 | 38.605 |
| rs2347603 | -0.0181 | 0.7418 | 5.65E-17 | A | T | 0.0022 | 56.511 |
| rs2347808 | -0.0125 | 0.5139 | 5.79E-11 | A | G | 0.0019 | 35.151 |
| rs2362487 | 0.0154 | 0.2473 | 3.57E-12 | G | C | 0.0022 | 39.756 |
| rs2390669 | 0.0174 | 0.1289 | 7.00E-10 | C | A | 0.0028 | 30.614 |
| rs244711 | 0.0279 | 0.686 | 1.54E-37 | T | C | 0.0022 | 151.037 |
| rs2454390 | -0.0176 | 0.8459 | 1.74E-11 | C | T | 0.0026 | 36.363 |
| rs246177 | 0.0214 | 0.3681 | 2.04E-27 | T | C | 0.002 | 95.942 |
| rs2490302 | 0.0221 | 0.9137 | 6.24E-11 | A | T | 0.0034 | 34.682 |
| rs249677 | -0.0109 | 0.6333 | 2.40E-08 | A | C | 0.002 | 24.847 |
| rs2521349 | 0.0155 | 0.3847 | 2.01E-15 | A | G | 0.0019 | 51.215 |
| rs2529090 | 0.0136 | 0.181 | 3.39E-08 | G | C | 0.0025 | 24.691 |
| rs2545339 | 0.0115 | 0.6288 | 3.48E-09 | G | A | 0.002 | 27.798 |
| rs2569888 | 0.0133 | 0.2449 | 2.29E-09 | A | G | 0.0022 | 29.458 |
| rs2578565 | -0.0141 | 0.6575 | 1.37E-12 | T | C | 0.002 | 40.319 |
| rs2592208 | -0.0124 | 0.513 | 5.52E-11 | A | C | 0.0019 | 34.594 |
| rs2607234 | -0.0302 | 0.9476 | 2.00E-12 | G | A | 0.0043 | 40.784 |
| rs261223 | 0.0175 | 0.3702 | 2.30E-19 | C | A | 0.0019 | 64.306 |
| rs2648725 | 0.0165 | 0.2134 | 8.20E-13 | A | T | 0.0023 | 41.156 |
| rs2663126 | -0.0139 | 0.6905 | 1.36E-11 | A | G | 0.0021 | 37.185 |
| rs2676298 | -0.0269 | 0.853 | 2.39E-23 | T | C | 0.0027 | 81.719 |
| rs2717008 | -0.0127 | 0.3835 | 4.99E-11 | C | T | 0.0019 | 34.341 |
| rs2748501 | -0.0195 | 0.4395 | 1.31E-24 | A | G | 0.0019 | 84.365 |
| rs2754255 | -0.0153 | 0.2245 | 1.05E-11 | G | A | 0.0023 | 36.702 |
| rs2763263 | -0.017 | 0.2445 | 1.37E-14 | A | T | 0.0022 | 48.076 |
| rs2788213 | 0.0123 | 0.7104 | 3.80E-09 | A | G | 0.0021 | 28.029 |
| rs2789365 | -0.0145 | 0.4807 | 1.14E-14 | T | C | 0.0019 | 47.266 |
| rs2807339 | 0.0162 | 0.7586 | 1.24E-13 | C | T | 0.0022 | 43.281 |
| rs2812208 | 0.1156 | 0.021 | 5.51E-68 | C | G | 0.0066 | 247.532 |
| rs28379706 | 0.0114 | 0.3931 | 4.49E-09 | C | T | 0.002 | 27.921 |
| rs28485212 | -0.0188 | 0.1494 | 1.24E-12 | T | C | 0.0027 | 40.449 |
| rs28529055 | -0.0147 | 0.4368 | 1.88E-14 | T | G | 0.0019 | 47.874 |
| rs28529426 | -0.0168 | 0.1711 | 5.82E-11 | T | C | 0.0026 | 36.048 |
| rs28592876 | 0.03 | 0.2051 | 9.09E-38 | A | G | 0.0023 | 132.167 |
| rs2871865 | -0.0493 | 0.1159 | 3.40E-62 | G | C | 0.003 | 224.373 |
| rs2871960 | 0.0469 | 0.4448 | 2.17E-135 | C | A | 0.0019 | 489.674 |
| rs28736838 | -0.0117 | 0.3017 | 1.07E-08 | T | C | 0.002 | 25.971 |
| rs2885697 | -0.0323 | 0.665 | 9.21E-60 | T | G | 0.002 | 209.386 |
| rs291979 | 0.0242 | 0.2289 | 6.76E-27 | A | G | 0.0023 | 93.101 |
| rs2923411 | 0.0127 | 0.5946 | 4.77E-11 | C | T | 0.0019 | 35.013 |
| rs2925155 | -0.015 | 0.2612 | 5.47E-12 | T | C | 0.0022 | 39.102 |
| rs293517 | -0.013 | 0.7011 | 2.80E-10 | C | T | 0.0021 | 31.893 |
| rs2971857 | -0.0119 | 0.5752 | 3.70E-10 | A | G | 0.0019 | 31.160 |
| rs2978362 | 0.0106 | 0.5326 | 2.85E-08 | T | C | 0.0019 | 25.188 |
| rs301807 | -0.0144 | 0.5815 | 2.53E-14 | G | A | 0.0019 | 45.445 |
| rs3103223 | 0.0126 | 0.7397 | 6.02E-09 | C | T | 0.0022 | 27.528 |
| rs310796 | 0.0142 | 0.6811 | 2.53E-12 | T | G | 0.002 | 39.442 |
| rs3116194 | -0.0295 | 0.0978 | 8.29E-21 | A | T | 0.0032 | 69.156 |
| rs3116602 | -0.0612 | 0.2153 | 9.53E-155 | G | T | 0.0023 | 570.526 |
| rs31196 | -0.0107 | 0.5724 | 2.07E-08 | A | C | 0.0019 | 25.235 |
| rs3205136 | -0.0184 | 0.0952 | 1.85E-08 | A | C | 0.0033 | 26.262 |
| rs331917 | -0.0127 | 0.5803 | 3.54E-11 | G | A | 0.0019 | 35.376 |
| rs332116 | -0.0206 | 0.2804 | 2.89E-22 | T | C | 0.0021 | 77.118 |
| rs336630 | -0.0106 | 0.43 | 2.90E-08 | T | C | 0.0019 | 24.800 |
| rs33973388 | 0.0249 | 0.4353 | 1.45E-38 | T | G | 0.0019 | 137.282 |
| rs34287 | 0.0187 | 0.341 | 1.17E-20 | A | G | 0.002 | 70.773 |
| rs34312629 | -0.017 | 0.2613 | 2.12E-15 | G | C | 0.0021 | 50.238 |
| rs34338597 | -0.0112 | 0.3832 | 7.88E-09 | G | A | 0.0019 | 26.700 |
| rs34345560 | 0.0219 | 0.1952 | 7.10E-20 | A | G | 0.0024 | 67.857 |
| rs34390533 | -0.0257 | 0.248 | 6.69E-32 | A | C | 0.0022 | 110.948 |
| rs34522021 | 0.0126 | 0.4547 | 3.38E-11 | T | C | 0.0019 | 35.450 |
| rs34776209 | -0.0317 | 0.2475 | 1.78E-47 | T | C | 0.0022 | 168.592 |
| rs34879158 | -0.0363 | 0.263 | 1.55E-63 | C | A | 0.0022 | 230.109 |
| rs35073631 | 0.0112 | 0.4331 | 5.92E-09 | C | T | 0.0019 | 27.735 |
| rs350832 | -0.0165 | 0.7709 | 3.44E-13 | A | G | 0.0023 | 43.302 |
| rs35288270 | -0.0328 | 0.1344 | 3.43E-32 | C | T | 0.0028 | 112.732 |
| rs35732917 | 0.0204 | 0.2843 | 2.08E-22 | C | T | 0.0021 | 76.264 |
| rs35756741 | -0.0378 | 0.0924 | 5.80E-31 | T | C | 0.0033 | 107.927 |
| rs35811052 | -0.0148 | 0.2559 | 8.84E-12 | G | A | 0.0022 | 37.561 |
| rs35963161 | -0.0157 | 0.4757 | 7.46E-16 | A | G | 0.0019 | 55.366 |
| rs36000545 | -0.022 | 0.3957 | 2.56E-29 | G | A | 0.002 | 104.241 |
| rs36012032 | 0.0298 | 0.091 | 9.93E-20 | A | C | 0.0033 | 66.157 |
| rs36048468 | 0.0254 | 0.2088 | 9.34E-28 | T | C | 0.0023 | 95.996 |
| rs36226649 | 0.0485 | 0.0667 | 3.05E-37 | C | T | 0.0038 | 131.896 |
| rs3768495 | -0.0178 | 0.7173 | 1.07E-17 | T | C | 0.0021 | 57.863 |
| rs3769598 | 0.0171 | 0.1438 | 1.32E-10 | G | A | 0.0027 | 32.421 |
| rs377599 | 0.0217 | 0.383 | 3.50E-29 | T | C | 0.0019 | 100.225 |
| rs3778858 | 0.0108 | 0.3698 | 4.26E-08 | T | G | 0.002 | 24.479 |
| rs3782232 | -0.0339 | 0.0712 | 2.41E-20 | A | G | 0.0037 | 68.445 |
| rs3782811 | -0.0165 | 0.2507 | 3.96E-14 | A | C | 0.0022 | 46.057 |
| rs3792819 | 0.021 | 0.0854 | 4.42E-10 | G | A | 0.0034 | 31.019 |
| rs3818416 | 0.0279 | 0.7645 | 2.01E-35 | C | A | 0.0022 | 126.233 |
| rs3822742 | 0.0162 | 0.3706 | 1.03E-16 | A | C | 0.002 | 55.130 |
| rs3828729 | -0.016 | 0.3094 | 4.67E-15 | G | A | 0.002 | 49.262 |
| rs395980 | -0.0184 | 0.2635 | 1.02E-17 | G | T | 0.0021 | 59.173 |
| rs40270 | 0.0151 | 0.7724 | 1.90E-11 | C | A | 0.0022 | 36.098 |
| rs4073154 | 0.0274 | 0.7784 | 1.92E-33 | G | A | 0.0023 | 116.644 |
| rs4076108 | 0.0174 | 0.2449 | 2.30E-15 | T | A | 0.0022 | 50.421 |
| rs4077103 | -0.0143 | 0.8426 | 4.18E-08 | A | C | 0.0026 | 24.423 |
| rs4121583 | 0.0118 | 0.6192 | 4.62E-09 | T | C | 0.002 | 29.566 |
| rs41311445 | -0.0328 | 0.0958 | 4.73E-24 | C | A | 0.0032 | 83.933 |
| rs4244809 | -0.0262 | 0.2082 | 5.55E-29 | A | G | 0.0023 | 101.923 |
| rs4252548 | -0.0753 | 0.0218 | 2.96E-31 | T | C | 0.0065 | 108.907 |
| rs4274112 | -0.0217 | 0.3733 | 2.47E-28 | G | A | 0.002 | 99.222 |
| rs4282339 | -0.0311 | 0.2075 | 6.16E-41 | A | G | 0.0023 | 143.269 |
| rs4287835 | 0.0147 | 0.534 | 9.94E-15 | C | T | 0.0019 | 48.427 |
| rs4380799 | -0.0255 | 0.39 | 6.45E-33 | G | T | 0.0021 | 139.343 |
| rs4383083 | 0.0111 | 0.6466 | 2.91E-08 | A | G | 0.002 | 25.354 |
| rs447352 | -0.0181 | 0.1408 | 6.62E-10 | T | C | 0.0029 | 35.691 |
| rs4504126 | 0.046 | 0.0275 | 1.62E-15 | C | A | 0.0058 | 50.964 |
| rs45474992 | -0.0617 | 0.0362 | 2.17E-33 | T | C | 0.0051 | 119.634 |
| rs45528934 | 0.0262 | 0.1623 | 1.97E-24 | T | C | 0.0026 | 84.055 |
| rs4602848 | 0.016 | 0.6812 | 3.14E-15 | G | A | 0.002 | 50.068 |
| rs4622329 | 0.0149 | 0.3496 | 8.62E-14 | A | G | 0.002 | 45.461 |
| rs4640244 | -0.02 | 0.3993 | 3.81E-25 | G | A | 0.0019 | 86.412 |
| rs4644481 | -0.0112 | 0.4328 | 3.54E-09 | T | C | 0.0019 | 27.731 |
| rs4655345 | -0.0246 | 0.3974 | 5.81E-38 | G | A | 0.0019 | 130.535 |
| rs4682483 | -0.0165 | 0.1534 | 2.65E-10 | A | G | 0.0026 | 31.840 |
| rs4683435 | 0.0144 | 0.7723 | 1.60E-10 | G | A | 0.0022 | 32.838 |
| rs4735761 | 0.0331 | 0.2857 | 3.66E-56 | C | A | 0.0021 | 201.426 |
| rs4748008 | -0.0125 | 0.4346 | 8.95E-11 | C | T | 0.0019 | 34.576 |
| rs4752829 | 0.0262 | 0.2861 | 5.90E-36 | A | G | 0.0021 | 126.286 |
| rs4788218 | 0.0275 | 0.401 | 5.52E-46 | C | T | 0.0019 | 163.632 |
| rs4807472 | -0.0158 | 0.6764 | 8.18E-15 | C | T | 0.002 | 49.209 |
| rs4815952 | -0.0161 | 0.5221 | 1.24E-16 | C | T | 0.0019 | 58.247 |
| rs4818280 | -0.0124 | 0.6269 | 2.84E-10 | T | C | 0.002 | 32.387 |
| rs4847378 | 0.0136 | 0.6121 | 1.64E-12 | T | G | 0.0019 | 39.549 |
| rs4852257 | -0.0231 | 0.5763 | 6.21E-34 | G | T | 0.0019 | 117.360 |
| rs4865956 | -0.0258 | 0.6965 | 3.85E-36 | A | T | 0.0021 | 126.741 |
| rs4870941 | -0.0297 | 0.238 | 1.09E-39 | C | G | 0.0023 | 144.098 |
| rs488621 | 0.0191 | 0.4688 | 2.86E-24 | G | A | 0.0019 | 81.821 |
| rs4932439 | -0.0151 | 0.8254 | 1.43E-09 | G | A | 0.0025 | 29.591 |
| rs4938359 | -0.0156 | 0.2024 | 3.24E-11 | G | A | 0.0024 | 35.380 |
| rs4940874 | 0.0148 | 0.8119 | 1.24E-09 | G | A | 0.0024 | 30.124 |
| rs4965298 | -0.0119 | 0.7127 | 1.81E-08 | T | C | 0.0021 | 26.112 |
| rs496783 | -0.0124 | 0.4648 | 8.13E-11 | G | A | 0.0019 | 34.446 |
| rs4976262 | -0.0245 | 0.3156 | 4.37E-33 | C | T | 0.002 | 116.780 |
| rs532499 | -0.0127 | 0.7413 | 4.90E-09 | C | T | 0.0022 | 27.855 |
| rs543650 | 0.025 | 0.5987 | 1.49E-37 | G | T | 0.002 | 135.258 |
| rs544136 | 0.0121 | 0.7482 | 2.50E-08 | G | T | 0.0022 | 24.840 |
| rs545104 | 0.0127 | 0.6164 | 8.08E-11 | C | T | 0.002 | 34.344 |
| rs55717234 | 0.0122 | 0.5732 | 1.34E-10 | G | A | 0.0019 | 32.791 |
| rs55758152 | 0.0145 | 0.3263 | 1.05E-12 | A | G | 0.002 | 41.623 |
| rs55852614 | -0.0393 | 0.2474 | 3.29E-73 | C | T | 0.0022 | 259.104 |
| rs55872725 | 0.0222 | 0.4037 | 1.46E-30 | T | C | 0.0019 | 106.858 |
| rs56112295 | 0.0154 | 0.2257 | 1.12E-10 | T | C | 0.0024 | 37.324 |
| rs56207600 | 0.0192 | 0.111 | 2.50E-10 | A | G | 0.003 | 32.759 |
| rs56239180 | -0.0459 | 0.0237 | 1.85E-13 | G | T | 0.0062 | 43.901 |
| rs56363908 | -0.0382 | 0.0422 | 3.88E-16 | G | A | 0.0047 | 53.118 |
| rs568267 | 0.0122 | 0.7431 | 2.23E-08 | T | C | 0.0022 | 25.588 |
| rs57059662 | 0.0118 | 0.6774 | 5.63E-09 | C | T | 0.002 | 27.402 |
| rs5742915 | 0.0248 | 0.4605 | 9.33E-39 | C | T | 0.0019 | 137.636 |
| rs57513571 | -0.0191 | 0.2004 | 7.02E-16 | T | C | 0.0024 | 52.646 |
| rs5753518 | 0.0242 | 0.0899 | 5.04E-13 | A | G | 0.0033 | 43.152 |
| rs57696574 | 0.0173 | 0.3981 | 4.50E-18 | C | A | 0.002 | 64.587 |
| rs577289 | -0.0125 | 0.2808 | 5.39E-09 | T | A | 0.0021 | 28.416 |
| rs57791149 | -0.0173 | 0.4024 | 3.26E-19 | C | T | 0.0019 | 64.818 |
| rs599004 | -0.0157 | 0.2807 | 6.53E-14 | T | C | 0.0021 | 44.820 |
| rs59950280 | -0.0254 | 0.3316 | 7.32E-36 | A | G | 0.002 | 128.801 |
| rs6000886 | 0.0131 | 0.6482 | 5.88E-11 | C | T | 0.002 | 35.242 |
| rs6028716 | -0.021 | 0.2587 | 4.58E-22 | A | G | 0.0022 | 76.169 |
| rs60389750 | -0.0175 | 0.3139 | 1.06E-16 | T | C | 0.0021 | 59.400 |
| rs60408354 | 0.0259 | 0.0732 | 1.15E-12 | A | G | 0.0036 | 40.984 |
| rs604723 | -0.0166 | 0.7248 | 8.16E-15 | C | T | 0.0021 | 49.500 |
| rs6054390 | -0.0188 | 0.628 | 1.45E-21 | A | G | 0.002 | 74.364 |
| rs6054491 | -0.0142 | 0.2404 | 2.15E-10 | G | C | 0.0022 | 33.159 |
| rs6066122 | 0.0127 | 0.7631 | 1.76E-08 | G | C | 0.0023 | 26.258 |
| rs60804050 | -0.0217 | 0.2559 | 5.01E-24 | A | G | 0.0021 | 80.756 |
| rs6082354 | -0.024 | 0.6675 | 1.19E-32 | C | A | 0.002 | 115.147 |
| rs610694 | 0.0136 | 0.4903 | 4.29E-13 | C | T | 0.0019 | 41.626 |
| rs6142059 | 0.0116 | 0.4926 | 1.19E-09 | C | T | 0.0019 | 30.288 |
| rs61729527 | -0.0346 | 0.0519 | 4.86E-16 | T | C | 0.0043 | 53.052 |
| rs61732778 | 0.023 | 0.071 | 3.13E-10 | A | G | 0.0037 | 31.422 |
| rs61827272 | 0.0144 | 0.2754 | 7.89E-12 | C | T | 0.0021 | 37.265 |
| rs61878760 | 0.019 | 0.0828 | 3.74E-08 | A | G | 0.0034 | 24.689 |
| rs61919240 | 0.0137 | 0.3244 | 9.77E-12 | A | T | 0.002 | 37.044 |
| rs61944841 | 0.0253 | 0.4138 | 3.54E-37 | A | G | 0.002 | 139.858 |
| rs62033029 | -0.0141 | 0.2064 | 1.73E-09 | A | G | 0.0023 | 29.326 |
| rs62103240 | 0.0212 | 0.071 | 1.40E-08 | A | G | 0.0037 | 26.696 |
| rs62106258 | -0.0504 | 0.0486 | 6.45E-31 | C | T | 0.0044 | 105.788 |
| rs62143873 | -0.0115 | 0.5033 | 1.19E-09 | A | G | 0.0019 | 29.773 |
| rs62370472 | -0.0253 | 0.209 | 1.45E-27 | C | T | 0.0023 | 95.308 |
| rs62501195 | -0.0198 | 0.1712 | 8.11E-15 | C | A | 0.0025 | 50.096 |
| rs62515437 | 0.0369 | 0.2253 | 8.79E-60 | T | G | 0.0023 | 214.106 |
| rs62621812 | 0.0743 | 0.0204 | 3.16E-27 | A | G | 0.0069 | 99.364 |
| rs6425817 | 0.0157 | 0.6724 | 2.98E-15 | G | A | 0.002 | 48.898 |
| rs6470771 | -0.0268 | 0.1694 | 1.54E-26 | C | A | 0.0025 | 91.020 |
| rs6502935 | -0.0125 | 0.7381 | 6.31E-09 | T | C | 0.0022 | 27.200 |
| rs6505216 | -0.0498 | 0.233 | 1.83E-101 | T | G | 0.0023 | 399.457 |
| rs6543146 | 0.0154 | 0.5579 | 4.19E-16 | G | T | 0.0019 | 52.680 |
| rs655113 | 0.0188 | 0.3006 | 7.42E-20 | C | T | 0.0021 | 66.922 |
| rs6570509 | -0.0244 | 0.2868 | 1.27E-31 | T | G | 0.0021 | 109.686 |
| rs6582398 | 0.014 | 0.6002 | 1.14E-12 | T | C | 0.002 | 42.356 |
| rs6593210 | 0.0146 | 0.2081 | 4.88E-10 | A | G | 0.0024 | 31.634 |
| rs664317 | -0.0177 | 0.8375 | 4.50E-12 | C | A | 0.0026 | 38.397 |
| rs6675858 | -0.0137 | 0.2141 | 2.40E-09 | T | C | 0.0023 | 28.440 |
| rs6693481 | -0.0143 | 0.6951 | 2.21E-12 | C | T | 0.002 | 39.029 |
| rs670318 | 0.0413 | 0.9515 | 2.52E-21 | C | T | 0.0044 | 70.891 |
| rs6721191 | -0.0144 | 0.5779 | 3.17E-14 | G | A | 0.0019 | 45.552 |
| rs6738207 | 0.0127 | 0.4007 | 4.14E-11 | A | G | 0.0019 | 34.880 |
| rs6739278 | -0.021 | 0.8079 | 1.27E-18 | C | T | 0.0024 | 61.639 |
| rs67527161 | -0.0182 | 0.209 | 5.79E-15 | C | T | 0.0023 | 49.316 |
| rs67551338 | 0.0576 | 0.0615 | 1.04E-47 | T | C | 0.004 | 172.503 |
| rs6762851 | -0.0218 | 0.3573 | 1.50E-28 | C | T | 0.002 | 98.293 |
| rs67716382 | 0.0226 | 0.2214 | 1.65E-23 | C | G | 0.0023 | 79.298 |
| rs68049170 | -0.0259 | 0.2764 | 2.69E-34 | A | G | 0.0021 | 120.845 |
| rs680882 | 0.0133 | 0.7606 | 1.98E-09 | G | T | 0.0022 | 29.006 |
| rs6821305 | 0.0204 | 0.399 | 3.15E-26 | C | A | 0.0019 | 89.881 |
| rs684905 | -0.0118 | 0.4176 | 7.10E-10 | T | C | 0.0019 | 30.497 |
| rs6849302 | 0.0155 | 0.1977 | 7.11E-11 | G | A | 0.0024 | 34.317 |
| rs6860245 | 0.0589 | 0.248 | 9.66E-160 | C | G | 0.0022 | 583.361 |
| rs6874142 | 0.0288 | 0.1138 | 5.15E-20 | G | T | 0.0031 | 75.337 |
| rs6902109 | -0.0167 | 0.5401 | 1.03E-18 | G | A | 0.0019 | 62.389 |
| rs6931421 | -0.0279 | 0.3224 | 2.31E-43 | G | T | 0.002 | 153.179 |
| rs6977416 | 0.0457 | 0.3338 | 1.43E-113 | A | G | 0.002 | 418.603 |
| rs700677 | 0.0173 | 0.3505 | 1.13E-18 | A | C | 0.002 | 61.361 |
| rs7014590 | -0.0228 | 0.2614 | 4.48E-26 | C | T | 0.0022 | 90.395 |
| rs7020491 | -0.0178 | 0.4266 | 1.22E-20 | T | C | 0.0019 | 69.801 |
| rs702886 | 0.012 | 0.3505 | 1.11E-09 | G | A | 0.002 | 29.521 |
| rs704660 | 0.0153 | 0.4095 | 2.28E-15 | T | C | 0.0019 | 50.978 |
| rs7082659 | 0.0156 | 0.8663 | 2.27E-08 | C | T | 0.0028 | 25.383 |
| rs7095472 | 0.0267 | 0.5335 | 7.66E-45 | G | A | 0.0019 | 159.822 |
| rs7129320 | -0.0389 | 0.1661 | 7.29E-53 | A | G | 0.0025 | 188.816 |
| rs713467 | 0.0146 | 0.4345 | 3.09E-14 | A | G | 0.0019 | 47.168 |
| rs71414738 | 0.015 | 0.1763 | 1.00E-09 | T | C | 0.0025 | 29.424 |
| rs7144307 | -0.0122 | 0.3853 | 6.10E-10 | C | T | 0.002 | 31.746 |
| rs7185244 | -0.0148 | 0.7759 | 1.40E-10 | C | T | 0.0023 | 34.299 |
| rs718603 | 0.0131 | 0.2774 | 6.68E-10 | T | C | 0.0021 | 30.978 |
| rs7220127 | -0.0105 | 0.4264 | 4.60E-08 | C | T | 0.0019 | 24.283 |
| rs7228151 | -0.0185 | 0.206 | 3.19E-15 | C | T | 0.0023 | 50.414 |
| rs7229520 | -0.0224 | 0.6616 | 9.21E-29 | A | G | 0.002 | 101.180 |
| rs723149 | -0.0276 | 0.5628 | 1.43E-47 | G | A | 0.0019 | 168.846 |
| rs72656010 | -0.0668 | 0.1322 | 7.31E-126 | C | T | 0.0028 | 461.449 |
| rs72657800 | -0.0219 | 0.0773 | 6.39E-10 | C | T | 0.0035 | 30.806 |
| rs72695791 | -0.0297 | 0.036 | 4.58E-09 | G | C | 0.0051 | 27.567 |
| rs72721979 | -0.0229 | 0.143 | 2.20E-17 | G | T | 0.0027 | 57.879 |
| rs72726050 | -0.0192 | 0.0958 | 1.84E-08 | C | T | 0.0034 | 28.756 |
| rs72771070 | 0.015 | 0.283 | 1.30E-12 | T | C | 0.0021 | 41.115 |
| rs72801843 | 0.0313 | 0.3015 | 8.83E-52 | A | T | 0.0021 | 185.865 |
| rs72809820 | -0.0111 | 0.324 | 3.13E-08 | T | C | 0.002 | 24.302 |
| rs72829852 | 0.0309 | 0.0621 | 3.74E-15 | T | C | 0.0039 | 50.083 |
| rs72841270 | 0.0294 | 0.1354 | 2.25E-26 | G | T | 0.0028 | 91.136 |
| rs7286917 | 0.0171 | 0.7461 | 5.18E-14 | G | A | 0.0023 | 49.886 |
| rs72894003 | -0.0423 | 0.0647 | 1.90E-28 | T | C | 0.0038 | 97.523 |
| rs73006226 | -0.0182 | 0.1267 | 2.23E-10 | A | C | 0.0029 | 33.006 |
| rs7301341 | -0.0255 | 0.3268 | 9.27E-37 | C | T | 0.002 | 128.856 |
| rs73052033 | -0.0151 | 0.1851 | 4.79E-10 | C | T | 0.0024 | 30.972 |
| rs73125634 | -0.0195 | 0.2782 | 5.11E-20 | T | G | 0.0021 | 68.768 |
| rs73197345 | 0.0211 | 0.1367 | 3.55E-14 | A | T | 0.0028 | 47.317 |
| rs7320878 | -0.015 | 0.6034 | 1.34E-14 | A | G | 0.0019 | 48.491 |
| rs7321635 | -0.0132 | 0.6465 | 2.58E-11 | C | A | 0.002 | 35.860 |
| rs7328187 | 0.0116 | 0.4976 | 1.19E-09 | G | T | 0.0019 | 30.294 |
| rs73384223 | -0.0205 | 0.1967 | 1.27E-17 | C | T | 0.0024 | 59.803 |
| rs73413540 | -0.0124 | 0.2248 | 4.42E-08 | T | C | 0.0023 | 24.130 |
| rs7367519 | 0.0164 | 0.6846 | 4.68E-16 | C | T | 0.002 | 52.301 |
| rs73696333 | 0.0191 | 0.2012 | 3.20E-15 | G | C | 0.0024 | 52.803 |
| rs73856768 | -0.0247 | 0.0805 | 1.55E-12 | C | T | 0.0035 | 40.668 |
| rs7418410 | 0.0155 | 0.4089 | 5.64E-16 | T | C | 0.0019 | 52.296 |
| rs74379684 | -0.0272 | 0.0751 | 4.39E-14 | T | C | 0.0036 | 46.280 |
| rs74458759 | 0.0171 | 0.2886 | 3.12E-15 | G | C | 0.0022 | 54.067 |
| rs7448554 | -0.0132 | 0.4999 | 1.77E-11 | A | C | 0.002 | 39.228 |
| rs74494415 | -0.0417 | 0.0398 | 1.82E-17 | T | C | 0.0049 | 59.848 |
| rs7485647 | -0.0261 | 0.844 | 1.04E-23 | A | G | 0.0026 | 80.779 |
| rs75022676 | -0.0163 | 0.2079 | 2.84E-12 | A | G | 0.0023 | 39.402 |
| rs7522400 | 0.0129 | 0.7683 | 5.62E-09 | G | A | 0.0022 | 26.677 |
| rs7543136 | -0.021 | 0.7209 | 9.96E-24 | T | C | 0.0021 | 79.914 |
| rs7543202 | 0.0129 | 0.6229 | 2.86E-11 | G | A | 0.0019 | 35.202 |
| rs75508358 | 0.0266 | 0.0457 | 4.29E-09 | T | C | 0.0045 | 27.789 |
| rs7563362 | 0.0352 | 0.8566 | 3.27E-39 | G | A | 0.0027 | 137.094 |
| rs7570235 | -0.0168 | 0.5911 | 2.08E-18 | C | T | 0.0019 | 61.437 |
| rs75702986 | -0.0163 | 0.1861 | 3.14E-11 | A | G | 0.0025 | 36.241 |
| rs757834 | 0.0256 | 0.1856 | 1.25E-25 | C | T | 0.0024 | 89.219 |
| rs7598430 | -0.016 | 0.5046 | 1.37E-17 | T | C | 0.0019 | 57.633 |
| rs7610055 | -0.0373 | 0.1207 | 3.55E-38 | A | G | 0.0029 | 133.004 |
| rs7633464 | 0.0175 | 0.4786 | 1.27E-20 | A | G | 0.0019 | 68.827 |
| rs76517946 | -0.0368 | 0.0814 | 1.69E-26 | A | C | 0.0035 | 91.203 |
| rs7679276 | -0.033 | 0.9544 | 5.93E-12 | G | A | 0.0048 | 42.681 |
| rs7689420 | 0.0466 | 0.8312 | 1.50E-76 | C | T | 0.0025 | 274.530 |
| rs7701233 | -0.0179 | 0.4283 | 5.12E-21 | C | T | 0.0019 | 70.659 |
| rs77013652 | 0.049 | 0.015 | 1.50E-09 | G | T | 0.0081 | 31.947 |
| rs772222 | 0.0121 | 0.2651 | 1.55E-08 | G | A | 0.0021 | 25.687 |
| rs7731023 | 0.0166 | 0.5749 | 3.48E-18 | G | A | 0.0019 | 60.650 |
| rs7735891 | 0.0259 | 0.4629 | 1.14E-42 | T | C | 0.0019 | 150.232 |
| rs77364196 | -0.033 | 0.053 | 8.35E-15 | A | G | 0.0043 | 49.224 |
| rs77447813 | 0.0224 | 0.087 | 3.21E-11 | C | G | 0.0034 | 35.892 |
| rs7768382 | -0.0201 | 0.4771 | 1.57E-26 | C | T | 0.0019 | 90.778 |
| rs77809369 | 0.0237 | 0.0642 | 9.83E-10 | T | C | 0.0039 | 30.389 |
| rs78051210 | 0.0263 | 0.0771 | 1.63E-13 | C | T | 0.0036 | 44.324 |
| rs7816345 | 0.0255 | 0.1682 | 6.22E-24 | T | C | 0.0025 | 81.937 |
| rs781669 | 0.0164 | 0.5236 | 3.14E-18 | T | C | 0.0019 | 60.422 |
| rs7826059 | 0.0114 | 0.6421 | 7.61E-09 | C | T | 0.002 | 26.895 |
| rs7828086 | 0.0135 | 0.2391 | 1.11E-09 | C | T | 0.0022 | 29.859 |
| rs78378222 | 0.138 | 0.0123 | 4.51E-56 | G | T | 0.0087 | 208.432 |
| rs7858712 | 0.0347 | 0.9144 | 1.04E-24 | G | A | 0.0034 | 84.884 |
| rs78766798 | 0.0319 | 0.0847 | 2.61E-20 | C | T | 0.0035 | 71.051 |
| rs7893378 | 0.0175 | 0.1143 | 2.36E-08 | A | G | 0.0031 | 27.920 |
| rs7902 | 0.0149 | 0.4469 | 5.22E-15 | G | A | 0.0019 | 49.421 |
| rs79441499 | -0.0138 | 0.4055 | 9.67E-13 | T | C | 0.0019 | 41.344 |
| rs7952436 | -0.0453 | 0.0823 | 1.62E-39 | T | C | 0.0034 | 139.607 |
| rs798548 | -0.0359 | 0.3006 | 2.86E-68 | C | T | 0.0021 | 244.126 |
| rs8000973 | 0.0134 | 0.5334 | 2.05E-12 | C | T | 0.0019 | 40.246 |
| rs80132799 | 0.0231 | 0.0679 | 1.31E-09 | T | C | 0.0038 | 30.413 |
| rs8017006 | 0.0122 | 0.3275 | 2.22E-09 | G | A | 0.002 | 29.521 |
| rs8018486 | -0.0138 | 0.1914 | 1.18E-08 | G | A | 0.0024 | 26.542 |
| rs8019890 | 0.025 | 0.5313 | 1.96E-38 | A | C | 0.0019 | 140.193 |
| rs8020095 | -0.0145 | 0.15 | 4.53E-08 | A | G | 0.0027 | 24.140 |
| rs80280630 | -0.0168 | 0.1115 | 2.28E-08 | T | C | 0.003 | 25.180 |
| rs80295797 | -0.0198 | 0.3265 | 3.83E-23 | T | C | 0.002 | 77.643 |
| rs8042578 | 0.0287 | 0.2426 | 2.29E-38 | G | C | 0.0022 | 136.328 |
| rs8054549 | -0.0251 | 0.4486 | 3.37E-39 | A | C | 0.0019 | 140.373 |
| rs8084413 | -0.0127 | 0.4693 | 3.25E-11 | A | G | 0.0019 | 36.176 |
| rs8136517 | 0.0267 | 0.0652 | 6.58E-12 | C | T | 0.0039 | 39.129 |
| rs822530 | 0.0255 | 0.7953 | 2.36E-27 | T | A | 0.0024 | 95.345 |
| rs839255 | -0.0126 | 0.6847 | 8.38E-10 | G | T | 0.0021 | 30.865 |
| rs867529 | 0.0184 | 0.2796 | 1.00E-18 | C | G | 0.0021 | 61.416 |
| rs876122 | 0.0162 | 0.8788 | 2.19E-08 | G | A | 0.0029 | 25.172 |
| rs8904 | -0.0157 | 0.363 | 1.52E-15 | A | G | 0.002 | 51.330 |
| rs900399 | 0.0164 | 0.3986 | 1.35E-17 | G | A | 0.0019 | 58.066 |
| rs905938 | 0.0394 | 0.2649 | 8.43E-77 | C | T | 0.0021 | 272.370 |
| rs909220 | -0.015 | 0.4443 | 3.24E-15 | A | G | 0.0019 | 50.029 |
| rs9266244 | -0.0427 | 0.7077 | 1.21E-94 | A | G | 0.0021 | 339.889 |
| rs9344126 | -0.0185 | 0.5137 | 2.21E-22 | C | T | 0.0019 | 77.003 |
| rs9375188 | 0.0136 | 0.4844 | 6.80E-13 | T | C | 0.0019 | 41.602 |
| rs9385002 | -0.0147 | 0.2394 | 3.26E-11 | T | A | 0.0022 | 35.434 |
| rs9388490 | 0.0462 | 0.4394 | 1.33E-130 | T | C | 0.0019 | 473.946 |
| rs947099 | 0.0117 | 0.3536 | 2.71E-09 | A | G | 0.002 | 28.177 |
| rs951366 | 0.0205 | 0.3932 | 9.15E-27 | C | T | 0.0019 | 90.309 |
| rs9517483 | -0.0181 | 0.699 | 2.26E-18 | G | A | 0.0021 | 62.078 |
| rs9525326 | -0.0184 | 0.1875 | 3.62E-14 | G | A | 0.0024 | 46.449 |
| rs9568031 | -0.0115 | 0.706 | 3.34E-08 | T | C | 0.0021 | 24.720 |
| rs9590328 | 0.0153 | 0.1417 | 2.02E-08 | G | A | 0.0027 | 25.638 |
| rs9594714 | 0.0144 | 0.3052 | 2.65E-12 | T | G | 0.0021 | 39.599 |
| rs9610447 | 0.0152 | 0.7471 | 5.29E-12 | T | C | 0.0022 | 39.312 |
| rs9634212 | 0.0471 | 0.221 | 8.59E-95 | A | C | 0.0023 | 344.174 |
| rs9636364 | 0.011 | 0.5429 | 5.09E-09 | A | G | 0.0019 | 27.041 |
| rs9647379 | 0.0215 | 0.4111 | 5.55E-29 | C | G | 0.0019 | 100.795 |
| rs9669278 | -0.0496 | 0.5183 | 5.25E-151 | C | T | 0.0019 | 553.771 |
| rs9809116 | -0.016 | 0.4081 | 1.31E-16 | G | A | 0.0019 | 55.691 |
| rs9828525 | 0.0121 | 0.4113 | 2.51E-10 | T | C | 0.0019 | 31.925 |
| rs9832919 | -0.0179 | 0.3574 | 7.90E-20 | G | A | 0.002 | 66.274 |
| rs9838614 | -0.0185 | 0.3881 | 1.21E-21 | G | T | 0.0019 | 73.200 |
| rs987666 | 0.0185 | 0.1188 | 2.33E-10 | A | G | 0.0029 | 32.266 |
| rs9890062 | 0.0267 | 0.0615 | 1.20E-11 | A | G | 0.0039 | 37.055 |
| rs9894577 | -0.031 | 0.318 | 1.40E-52 | A | G | 0.002 | 187.755 |
| rs9898189 | -0.0163 | 0.6446 | 1.88E-15 | G | C | 0.0021 | 54.816 |
| rs990315 | -0.0115 | 0.6229 | 5.07E-09 | C | T | 0.002 | 27.975 |
| rs9905385 | -0.0339 | 0.6707 | 1.94E-63 | G | A | 0.002 | 228.673 |
| rs9957318 | 0.0187 | 0.348 | 1.02E-20 | G | A | 0.002 | 71.459 |

SNP, single nucleotide polymorphism; Beta coefficients are in standard deviation (SD) unit per allele; EAF, effect allele frequency; SE, standard error;

**Supplementary Table 8.** Characteristics of selected SNPs concerning the causal effect of ALM on MI

| **SNP** | **Beta** | **EAF** | ***P*-value** | **Effect**  **allele** | **Other**  **allele** | **SE** | ***F*-value** |
| --- | --- | --- | --- | --- | --- | --- | --- |
| rs10019221 | -0.0124 | 0.5974 | 1.22E-10 | T | G | 0.0019 | 55.880 |
| rs1005723 | 0.0161 | 0.1908 | 1.79E-11 | T | C | 0.0024 | 33.303 |
| rs10068640 | 0.0112 | 0.3651 | 1.28E-08 | A | G | 0.002 | 36.041 |
| rs10075249 | 0.0143 | 0.4952 | 4.56E-14 | T | C | 0.0019 | 26.185 |
| rs10107388 | -0.0159 | 0.3691 | 6.95E-16 | C | T | 0.002 | 46.035 |
| rs10112506 | -0.012 | 0.3898 | 5.76E-10 | G | A | 0.0019 | 53.018 |
| rs10123619 | -0.0171 | 0.8425 | 4.05E-11 | G | A | 0.0026 | 30.845 |
| rs10128333 | -0.0146 | 0.1676 | 9.51E-09 | T | C | 0.0025 | 34.942 |
| rs10171272 | 0.0136 | 0.3035 | 2.87E-11 | A | C | 0.002 | 26.780 |
| rs10202701 | 0.0227 | 0.5419 | 3.11E-33 | T | C | 0.0019 | 35.210 |
| rs10202845 | -0.0288 | 0.1133 | 5.35E-22 | G | A | 0.003 | 115.217 |
| rs10203320 | 0.0138 | 0.3289 | 7.86E-12 | C | T | 0.002 | 75.048 |
| rs10205141 | 0.0241 | 0.0476 | 4.71E-08 | G | A | 0.0044 | 37.855 |
| rs10221831 | 0.03 | 0.0321 | 1.80E-08 | T | C | 0.0053 | 23.711 |
| rs10225945 | -0.0146 | 0.1499 | 3.28E-08 | G | A | 0.0026 | 25.181 |
| rs10242866 | 0.0157 | 0.3983 | 3.66E-16 | T | C | 0.0019 | 24.461 |
| rs10283100 | 0.0575 | 0.9445 | 4.11E-44 | G | A | 0.0041 | 53.201 |
| rs1035583 | 0.0148 | 0.6177 | 1.99E-14 | A | G | 0.0019 | 156.119 |
| rs10421750 | -0.0145 | 0.312 | 5.73E-12 | T | G | 0.0021 | 46.583 |
| rs10453441 | -0.0139 | 0.402 | 9.10E-13 | G | A | 0.002 | 40.644 |
| rs10461725 | 0.0134 | 0.6561 | 1.98E-11 | C | G | 0.002 | 41.828 |
| rs10471339 | -0.011 | 0.3823 | 1.45E-08 | G | C | 0.0019 | 36.486 |
| rs1047891 | 0.0233 | 0.3159 | 5.70E-31 | A | C | 0.002 | 25.732 |
| rs10483727 | -0.0368 | 0.6109 | 6.73E-80 | C | T | 0.0019 | 105.672 |
| rs1056747 | -0.0155 | 0.4117 | 8.05E-16 | G | A | 0.0019 | 290.056 |
| rs1063582 | -0.0185 | 0.7649 | 1.12E-16 | G | T | 0.0022 | 52.405 |
| rs10748128 | 0.0255 | 0.3446 | 6.77E-38 | T | G | 0.002 | 55.428 |
| rs10749157 | 0.0113 | 0.3584 | 1.24E-08 | C | T | 0.002 | 132.283 |
| rs10776560 | -0.0157 | 0.4995 | 7.88E-17 | T | C | 0.0019 | 26.442 |
| rs10793931 | -0.0132 | 0.3555 | 3.23E-11 | C | G | 0.002 | 55.497 |
| rs10796828 | 0.0154 | 0.6348 | 5.77E-15 | G | T | 0.002 | 35.952 |
| rs10815274 | 0.0124 | 0.4562 | 6.46E-11 | C | A | 0.0019 | 49.514 |
| rs10822117 | -0.0176 | 0.237 | 4.24E-15 | G | A | 0.0022 | 34.352 |
| rs10824307 | -0.0194 | 0.6471 | 1.79E-22 | C | G | 0.002 | 50.445 |
| rs10829226 | -0.0112 | 0.6359 | 1.33E-08 | A | G | 0.002 | 77.406 |
| rs10832963 | -0.0203 | 0.7445 | 9.23E-21 | G | T | 0.0022 | 26.154 |
| rs10845408 | 0.0255 | 0.3538 | 3.25E-38 | T | C | 0.002 | 70.598 |
| rs10858246 | -0.0188 | 0.3183 | 2.35E-20 | C | G | 0.002 | 133.909 |
| rs10864899 | -0.0112 | 0.5643 | 3.85E-09 | G | A | 0.0019 | 69.070 |
| rs10922475 | 0.0159 | 0.5395 | 2.17E-17 | A | C | 0.0019 | 27.774 |
| rs10948 | -0.0252 | 0.6638 | 3.43E-36 | T | G | 0.002 | 56.565 |
| rs10962212 | 0.0143 | 0.4184 | 7.47E-14 | C | G | 0.0019 | 127.654 |
| rs10975935 | -0.0121 | 0.2464 | 4.15E-08 | G | A | 0.0022 | 44.813 |
| rs10982888 | -0.0328 | 0.1136 | 3.97E-28 | A | T | 0.003 | 24.482 |
| rs11009928 | -0.0148 | 0.2547 | 1.04E-11 | G | A | 0.0022 | 97.572 |
| rs11014285 | 0.0342 | 0.1654 | 2.89E-40 | A | G | 0.0026 | 37.445 |
| rs11042717 | -0.029 | 0.4896 | 4.04E-53 | C | T | 0.0019 | 145.439 |
| rs11049704 | -0.0183 | 0.293 | 1.10E-18 | G | C | 0.0021 | 189.324 |
| rs11060942 | -0.0354 | 0.0344 | 7.49E-12 | A | G | 0.0052 | 62.478 |
| rs11068230 | 0.0238 | 0.8656 | 9.67E-18 | G | C | 0.0028 | 37.486 |
| rs11070842 | -0.0146 | 0.1631 | 1.30E-08 | C | T | 0.0026 | 59.348 |
| rs11098677 | -0.0263 | 0.7875 | 3.94E-30 | T | G | 0.0023 | 26.202 |
| rs11121615 | -0.0202 | 0.6894 | 3.32E-23 | T | C | 0.002 | 104.255 |
| rs111365325 | -0.0271 | 0.2312 | 1.12E-33 | T | C | 0.0022 | 78.691 |
| rs111622870 | -0.0282 | 0.0482 | 1.86E-10 | C | T | 0.0044 | 117.579 |
| rs11175919 | 0.0349 | 0.0262 | 3.16E-09 | A | G | 0.0059 | 32.855 |
| rs111901094 | -0.0253 | 0.182 | 4.04E-24 | T | G | 0.0025 | 27.985 |
| rs11191208 | 0.0147 | 0.2057 | 3.69E-10 | A | G | 0.0024 | 85.827 |
| rs11198591 | 0.0148 | 0.3684 | 4.91E-14 | A | G | 0.002 | 31.795 |
| rs112021215 | -0.0147 | 0.201 | 5.99E-09 | C | T | 0.0025 | 45.899 |
| rs11210892 | 0.0118 | 0.6745 | 3.57E-09 | A | G | 0.002 | 31.252 |
| rs112153300 | 0.0261 | 0.089 | 7.05E-15 | A | G | 0.0034 | 27.530 |
| rs11217863 | -0.0268 | 0.1162 | 1.06E-19 | A | G | 0.003 | 49.741 |
| rs11221657 | 0.0179 | 0.1352 | 1.21E-10 | G | T | 0.0028 | 66.431 |
| rs11243202 | 0.0302 | 0.486 | 2.83E-57 | C | T | 0.0019 | 33.737 |
| rs112537273 | -0.0212 | 0.2299 | 3.34E-21 | C | T | 0.0022 | 205.251 |
| rs11260035 | 0.015 | 0.2761 | 1.86E-12 | A | G | 0.0021 | 71.664 |
| rs11260623 | 0.0117 | 0.5085 | 4.86E-10 | T | G | 0.0019 | 40.499 |
| rs112873218 | 0.0216 | 0.1054 | 4.02E-12 | T | C | 0.0031 | 30.810 |
| rs113107560 | -0.019 | 0.4118 | 6.99E-23 | G | T | 0.0019 | 39.618 |
| rs113146332 | 0.0311 | 0.0381 | 2.99E-10 | C | G | 0.0049 | 78.753 |
| rs113232639 | 0.0327 | 0.493 | 4.79E-64 | A | G | 0.0019 | 31.921 |
| rs113289555 | -0.0206 | 0.2333 | 7.33E-20 | T | G | 0.0023 | 240.801 |
| rs113671109 | -0.015 | 0.22 | 4.23E-11 | C | T | 0.0023 | 68.362 |
| rs113827862 | -0.0235 | 0.0608 | 4.67E-09 | C | T | 0.004 | 34.770 |
| rs113898003 | -0.036 | 0.2635 | 1.19E-63 | C | T | 0.0021 | 28.399 |
| rs11580040 | 0.0325 | 0.08 | 6.76E-21 | G | A | 0.0035 | 226.596 |
| rs11590254 | 0.0186 | 0.3114 | 4.34E-20 | T | A | 0.002 | 70.014 |
| rs115912456 | 0.0577 | 0.0412 | 3.69E-34 | G | A | 0.0047 | 66.811 |
| rs116008080 | -0.0415 | 0.0238 | 4.06E-11 | A | G | 0.0063 | 118.458 |
| rs116052377 | 0.0225 | 0.0809 | 7.89E-11 | A | G | 0.0035 | 36.035 |
| rs11605297 | 0.0146 | 0.2328 | 8.03E-11 | A | G | 0.0022 | 33.899 |
| rs116092985 | -0.0401 | 0.0958 | 1.17E-34 | G | A | 0.0033 | 34.285 |
| rs11612462 | 0.015 | 0.1698 | 2.55E-09 | G | T | 0.0025 | 125.463 |
| rs11629593 | -0.0109 | 0.6158 | 4.42E-08 | G | T | 0.002 | 28.563 |
| rs11633371 | 0.0216 | 0.4764 | 7.49E-30 | T | G | 0.0019 | 25.313 |
| rs116339650 | -0.0175 | 0.127 | 1.05E-09 | G | A | 0.0029 | 104.823 |
| rs116493405 | 0.0287 | 0.0537 | 9.52E-12 | A | G | 0.0042 | 30.577 |
| rs11672848 | -0.0171 | 0.5246 | 7.73E-19 | T | C | 0.0019 | 37.695 |
| rs11684531 | -0.0172 | 0.1328 | 4.17E-10 | G | A | 0.0028 | 65.678 |
| rs1168768 | 0.0332 | 0.975 | 3.61E-08 | T | C | 0.006 | 30.682 |
| rs116919274 | 0.0271 | 0.046 | 3.52E-09 | A | G | 0.0046 | 24.195 |
| rs117068593 | 0.0403 | 0.1895 | 8.83E-62 | T | C | 0.0024 | 29.023 |
| rs117203652 | -0.0346 | 0.03 | 4.32E-10 | A | G | 0.0055 | 224.732 |
| rs11720869 | 0.0141 | 0.6686 | 2.54E-12 | A | G | 0.002 | 31.373 |
| rs11721522 | 0.0106 | 0.4125 | 4.03E-08 | G | A | 0.0019 | 39.671 |
| rs11727162 | -0.017 | 0.4987 | 2.15E-19 | T | C | 0.0019 | 24.521 |
| rs117335233 | -0.0236 | 0.053 | 2.56E-08 | G | T | 0.0042 | 65.069 |
| rs1177765 | -0.0232 | 0.4681 | 1.32E-34 | C | T | 0.0019 | 25.174 |
| rs11778491 | -0.0247 | 0.2518 | 8.39E-30 | C | G | 0.0022 | 120.708 |
| rs117818446 | 0.0423 | 0.0202 | 5.29E-10 | A | G | 0.0068 | 103.524 |
| rs117972846 | 0.0335 | 0.0285 | 5.47E-09 | G | T | 0.0057 | 31.892 |
| rs11867855 | -0.0132 | 0.2372 | 2.93E-09 | T | G | 0.0022 | 27.982 |
| rs1190540 | 0.0125 | 0.6983 | 1.62E-09 | G | A | 0.0021 | 28.391 |
| rs11959466 | 0.038 | 0.0564 | 2.24E-19 | T | C | 0.0042 | 29.644 |
| rs1202186 | -0.012 | 0.6548 | 1.65E-09 | T | C | 0.002 | 69.211 |
| rs12051245 | 0.0299 | 0.2318 | 2.56E-40 | C | T | 0.0022 | 29.312 |
| rs12074850 | 0.0393 | 0.0898 | 2.72E-33 | G | A | 0.0033 | 143.398 |
| rs12099669 | 0.0331 | 0.6962 | 1.39E-58 | A | G | 0.002 | 113.706 |
| rs12150907 | -0.0219 | 0.1977 | 7.22E-20 | A | G | 0.0024 | 208.763 |
| rs12185775 | -0.0167 | 0.1172 | 3.66E-08 | C | G | 0.003 | 68.513 |
| rs12188208 | -0.0195 | 0.2349 | 1.40E-18 | C | A | 0.0022 | 25.985 |
| rs12230946 | 0.0271 | 0.0911 | 1.45E-16 | A | G | 0.0033 | 61.547 |
| rs12340775 | -0.0287 | 0.0552 | 1.73E-11 | A | G | 0.0043 | 54.765 |
| rs12344515 | -0.0163 | 0.2402 | 2.28E-13 | T | C | 0.0022 | 38.686 |
| rs12347137 | -0.046 | 0.2023 | 9.80E-85 | C | A | 0.0024 | 43.668 |
| rs12351226 | 0.0218 | 0.1712 | 9.13E-18 | T | C | 0.0025 | 307.697 |
| rs12423821 | 0.0161 | 0.1575 | 1.17E-09 | C | T | 0.0027 | 60.730 |
| rs12461874 | -0.0181 | 0.2784 | 1.26E-17 | A | C | 0.0021 | 30.975 |
| rs12483401 | -0.0387 | 0.0216 | 9.22E-09 | C | T | 0.0067 | 59.273 |
| rs12512942 | -0.0162 | 0.6376 | 1.58E-16 | A | G | 0.002 | 28.503 |
| rs12517711 | -0.0147 | 0.3917 | 2.79E-14 | C | T | 0.0019 | 54.613 |
| rs12519407 | 0.0181 | 0.2567 | 3.38E-17 | C | A | 0.0022 | 46.369 |
| rs12533452 | 0.0237 | 0.1569 | 1.31E-19 | T | C | 0.0026 | 56.296 |
| rs12536902 | 0.0479 | 0.0143 | 3.66E-09 | A | C | 0.0081 | 66.917 |
| rs12541381 | -0.0319 | 0.2575 | 2.81E-49 | A | G | 0.0022 | 29.124 |
| rs12563442 | 0.0122 | 0.2669 | 9.93E-09 | C | T | 0.0021 | 175.267 |
| rs1260326 | 0.0323 | 0.6045 | 6.16E-64 | C | T | 0.0019 | 26.226 |
| rs12616192 | -0.0261 | 0.0676 | 6.83E-12 | A | G | 0.0038 | 224.719 |
| rs12655296 | -0.011 | 0.6251 | 1.62E-08 | T | C | 0.002 | 38.667 |
| rs12672217 | 0.0139 | 0.3609 | 1.33E-12 | A | G | 0.002 | 25.536 |
| rs12700901 | -0.0184 | 0.4055 | 1.67E-21 | A | C | 0.0019 | 40.133 |
| rs12702693 | 0.0173 | 0.4542 | 6.56E-20 | T | C | 0.0019 | 73.506 |
| rs12713004 | 0.0367 | 0.7254 | 2.40E-68 | G | A | 0.0021 | 66.821 |
| rs12724708 | 0.0243 | 0.3574 | 1.66E-35 | T | A | 0.002 | 241.723 |
| rs12773500 | 0.0171 | 0.1375 | 5.05E-10 | T | C | 0.0028 | 122.152 |
| rs12831751 | 0.0172 | 0.2857 | 1.57E-16 | C | A | 0.0021 | 31.229 |
| rs12882130 | -0.0202 | 0.3836 | 1.88E-24 | G | C | 0.002 | 54.372 |
| rs12907139 | -0.0149 | 0.5245 | 4.89E-15 | A | G | 0.0019 | 86.897 |
| rs1290786 | -0.0143 | 0.4307 | 7.14E-14 | T | C | 0.0019 | 49.865 |
| rs12909863 | 0.0189 | 0.2512 | 6.05E-18 | C | G | 0.0022 | 45.155 |
| rs12926103 | 0.0272 | 0.0661 | 9.60E-13 | A | G | 0.0038 | 60.512 |
| rs12943867 | 0.0184 | 0.3367 | 7.57E-20 | A | G | 0.002 | 41.130 |
| rs12962050 | 0.0153 | 0.6445 | 1.52E-14 | A | G | 0.002 | 68.097 |
| rs12997625 | -0.017 | 0.5266 | 1.50E-19 | T | C | 0.0019 | 48.302 |
| rs13037813 | 0.0292 | 0.2391 | 1.71E-39 | C | T | 0.0022 | 64.885 |
| rs13103161 | -0.0284 | 0.3891 | 2.38E-48 | A | T | 0.0019 | 139.728 |
| rs13109280 | 0.0131 | 0.6618 | 9.15E-11 | G | A | 0.002 | 172.707 |
| rs13123591 | 0.0185 | 0.3375 | 2.35E-20 | G | T | 0.002 | 34.590 |
| rs13127468 | -0.0123 | 0.4629 | 9.86E-11 | A | C | 0.0019 | 68.920 |
| rs13170063 | -0.0152 | 0.5921 | 4.11E-15 | A | G | 0.0019 | 33.874 |
| rs1319012 | -0.052 | 0.9259 | 3.30E-45 | A | T | 0.0037 | 50.253 |
| rs13209685 | 0.0277 | 0.1594 | 7.49E-27 | T | G | 0.0026 | 167.119 |
| rs1324538 | 0.0237 | 0.3836 | 1.73E-34 | A | T | 0.0019 | 92.598 |
| rs1325596 | 0.0287 | 0.5476 | 2.77E-52 | A | G | 0.0019 | 119.627 |
| rs1330826 | 0.0162 | 0.2268 | 1.04E-12 | C | G | 0.0023 | 183.824 |
| rs13316 | 0.0115 | 0.4301 | 3.66E-09 | A | C | 0.0019 | 41.446 |
| rs13391980 | -0.0225 | 0.1198 | 7.60E-15 | A | G | 0.0029 | 29.192 |
| rs1340022 | 0.0118 | 0.4868 | 4.47E-10 | C | T | 0.0019 | 48.076 |
| rs1341215 | 0.0229 | 0.1374 | 6.32E-17 | A | G | 0.0027 | 31.326 |
| rs13430869 | 0.0272 | 0.7427 | 6.37E-37 | T | G | 0.0021 | 55.975 |
| rs139163241 | -0.0164 | 0.1394 | 1.79E-09 | G | T | 0.0027 | 127.347 |
| rs140440099 | 0.0613 | 0.0234 | 1.45E-22 | A | G | 0.0063 | 29.057 |
| rs1405227 | 0.0129 | 0.3167 | 1.57E-10 | A | G | 0.002 | 77.340 |
| rs143076454 | -0.0499 | 0.0186 | 1.06E-12 | A | G | 0.007 | 32.430 |
| rs143384 | 0.0725 | 0.4038 | 1.00E-200 | G | A | 0.0019 | 40.933 |
| rs143554698 | -0.0257 | 0.1409 | 3.39E-21 | T | C | 0.0027 | 1142.378 |
| rs144109601 | -0.0278 | 0.0418 | 5.15E-09 | A | C | 0.0048 | 72.005 |
| rs1443536 | 0.0218 | 0.305 | 1.91E-26 | G | A | 0.0021 | 27.876 |
| rs1444628 | 0.024 | 0.6904 | 6.85E-32 | T | C | 0.002 | 90.732 |
| rs144627572 | 0.0439 | 0.0329 | 1.30E-16 | A | G | 0.0053 | 110.894 |
| rs147110934 | -0.0722 | 0.0243 | 9.39E-32 | T | G | 0.0062 | 55.224 |
| rs147233090 | -0.0446 | 0.0247 | 3.95E-13 | T | C | 0.0061 | 111.322 |
| rs1472852 | -0.0638 | 0.1582 | 8.22E-135 | A | C | 0.0026 | 43.154 |
| rs1478575 | 0.0312 | 0.6843 | 5.20E-54 | A | T | 0.002 | 488.656 |
| rs1556659 | 0.0163 | 0.3818 | 7.19E-17 | T | C | 0.002 | 189.447 |
| rs1584011 | 0.0159 | 0.3559 | 9.78E-16 | G | T | 0.002 | 56.477 |
| rs16989695 | -0.0139 | 0.5165 | 1.93E-13 | A | G | 0.0019 | 52.192 |
| rs17197114 | 0.0177 | 0.1772 | 1.54E-12 | C | T | 0.0025 | 43.452 |
| rs17205463 | -0.0263 | 0.4477 | 4.21E-43 | T | C | 0.0019 | 41.136 |
| rs17246129 | 0.0254 | 0.3044 | 1.27E-35 | A | G | 0.002 | 154.063 |
| rs17278379 | 0.0226 | 0.124 | 2.40E-15 | C | T | 0.0029 | 123.045 |
| rs1730028 | 0.0131 | 0.4174 | 7.39E-12 | G | T | 0.0019 | 49.965 |
| rs173135 | -0.0341 | 0.115 | 3.25E-30 | T | C | 0.003 | 37.582 |
| rs17400325 | 0.0345 | 0.0415 | 2.10E-13 | C | T | 0.0047 | 106.593 |
| rs17408561 | 0.0123 | 0.3908 | 6.00E-10 | G | A | 0.002 | 42.638 |
| rs17478946 | -0.0192 | 0.3004 | 9.98E-21 | G | A | 0.0021 | 32.436 |
| rs17681189 | -0.0131 | 0.4231 | 5.82E-12 | A | C | 0.0019 | 69.774 |
| rs17718736 | 0.0115 | 0.3226 | 1.39E-08 | A | C | 0.002 | 37.722 |
| rs177591 | -0.0191 | 0.1489 | 1.51E-12 | G | C | 0.0027 | 26.026 |
| rs17818592 | -0.0129 | 0.4367 | 1.23E-11 | C | T | 0.0019 | 41.635 |
| rs1786263 | -0.019 | 0.6055 | 1.03E-22 | T | G | 0.0019 | 36.865 |
| rs1797070 | 0.0219 | 0.2684 | 4.93E-25 | A | G | 0.0021 | 77.664 |
| rs1823217 | -0.0181 | 0.6449 | 4.06E-20 | G | A | 0.002 | 84.821 |
| rs182798714 | 0.0376 | 0.0274 | 1.45E-09 | T | A | 0.0062 | 67.568 |
| rs1880318 | 0.0147 | 0.2039 | 6.86E-10 | A | G | 0.0024 | 33.929 |
| rs188617336 | 0.0138 | 0.2983 | 6.66E-11 | T | C | 0.0021 | 31.588 |
| rs1899040 | 0.0152 | 0.7962 | 9.04E-11 | T | C | 0.0023 | 35.898 |
| rs190823861 | -0.0345 | 0.0466 | 2.09E-14 | A | G | 0.0045 | 33.761 |
| rs1933081 | 0.0267 | 0.0834 | 5.33E-15 | A | T | 0.0034 | 47.623 |
| rs200439 | -0.0128 | 0.2207 | 1.51E-08 | C | A | 0.0023 | 49.078 |
| rs2005172 | 0.048 | 0.6397 | 2.35E-128 | C | A | 0.002 | 25.376 |
| rs200739311 | -0.0128 | 0.6188 | 1.55E-10 | C | T | 0.002 | 478.696 |
| rs201570119 | 0.0194 | 0.2098 | 5.48E-17 | C | T | 0.0023 | 34.804 |
| rs2019203 | 0.0189 | 0.4909 | 1.84E-23 | A | C | 0.0019 | 56.192 |
| rs2025609 | 0.0186 | 0.8511 | 2.00E-12 | G | C | 0.0026 | 80.403 |
| rs2025808 | 0.0122 | 0.2541 | 1.72E-08 | A | C | 0.0022 | 39.483 |
| rs2035901 | 0.024 | 0.4679 | 9.43E-37 | G | A | 0.0019 | 25.404 |
| rs2070598 | 0.0204 | 0.4559 | 6.36E-27 | A | G | 0.0019 | 129.172 |
| rs2071450 | -0.0174 | 0.3675 | 8.85E-19 | T | C | 0.002 | 92.977 |
| rs2089111 | -0.0172 | 0.267 | 1.73E-15 | G | C | 0.0022 | 63.380 |
| rs2101017 | -0.0223 | 0.8695 | 1.44E-15 | T | C | 0.0028 | 52.143 |
| rs2105333 | -0.019 | 0.6648 | 1.70E-21 | G | T | 0.002 | 50.818 |
| rs212526 | 0.0214 | 0.6007 | 3.84E-29 | C | T | 0.0019 | 72.451 |
| rs2138374 | -0.0149 | 0.696 | 2.79E-13 | C | T | 0.002 | 98.936 |
| rs2140619 | 0.0113 | 0.4128 | 5.18E-09 | G | A | 0.0019 | 42.303 |
| rs2142331 | -0.0165 | 0.6023 | 1.38E-17 | T | C | 0.0019 | 27.873 |
| rs2142644 | -0.0181 | 0.672 | 3.37E-19 | A | C | 0.002 | 58.731 |
| rs2181834 | 0.0254 | 0.5503 | 7.69E-41 | T | G | 0.0019 | 65.034 |
| rs2188805 | 0.0114 | 0.3359 | 2.00E-08 | C | A | 0.002 | 143.815 |
| rs2194411 | 0.0443 | 0.1283 | 2.43E-54 | A | G | 0.0029 | 26.107 |
| rs2209098 | 0.024 | 0.3108 | 1.73E-32 | C | T | 0.002 | 197.727 |
| rs2212926 | -0.022 | 0.2106 | 7.75E-21 | A | C | 0.0023 | 111.130 |
| rs2229840 | 0.0341 | 0.1597 | 3.02E-40 | T | C | 0.0026 | 72.468 |
| rs2230033 | -0.0265 | 0.5644 | 3.49E-43 | A | G | 0.0019 | 140.559 |
| rs2236096 | 0.018 | 0.2327 | 1.29E-15 | C | T | 0.0023 | 155.522 |
| rs2236406 | 0.0394 | 0.3493 | 1.26E-87 | C | T | 0.002 | 52.099 |
| rs2237485 | 0.0191 | 0.2234 | 3.73E-17 | A | G | 0.0023 | 317.946 |
| rs2240735 | 0.0189 | 0.748 | 3.99E-18 | T | C | 0.0022 | 57.000 |
| rs2268718 | 0.0141 | 0.2704 | 3.24E-11 | T | C | 0.0021 | 60.640 |
| rs2270894 | -0.0332 | 0.2034 | 1.25E-42 | G | C | 0.0024 | 35.321 |
| rs2274351 | 0.017 | 0.5433 | 3.07E-19 | T | C | 0.0019 | 160.878 |
| rs2283200 | -0.0281 | 0.0559 | 1.48E-11 | T | C | 0.0042 | 64.581 |
| rs2289629 | -0.0148 | 0.3451 | 8.02E-14 | A | G | 0.002 | 37.528 |
| rs2296316 | -0.0192 | 0.4643 | 1.59E-23 | C | T | 0.0019 | 44.582 |
| rs2303423 | 0.0168 | 0.1095 | 2.64E-08 | C | T | 0.003 | 82.580 |
| rs2305141 | 0.0183 | 0.5961 | 1.07E-21 | G | A | 0.0019 | 24.784 |
| rs2324154 | 0.015 | 0.5096 | 1.92E-15 | A | C | 0.0019 | 72.617 |
| rs234640 | -0.0131 | 0.5142 | 3.87E-12 | T | C | 0.0019 | 50.639 |
| rs2347603 | -0.0181 | 0.7418 | 5.65E-17 | A | T | 0.0022 | 38.605 |
| rs2347808 | -0.0125 | 0.5139 | 5.79E-11 | A | G | 0.0019 | 56.511 |
| rs2362487 | 0.0154 | 0.2473 | 3.57E-12 | G | C | 0.0022 | 35.151 |
| rs2390669 | 0.0174 | 0.1289 | 7.00E-10 | C | A | 0.0028 | 39.756 |
| rs244711 | 0.0279 | 0.686 | 1.54E-37 | T | C | 0.0022 | 30.614 |
| rs2454390 | -0.0176 | 0.8459 | 1.74E-11 | C | T | 0.0026 | 151.037 |
| rs246177 | 0.0214 | 0.3681 | 2.04E-27 | T | C | 0.002 | 36.363 |
| rs2490302 | 0.0221 | 0.9137 | 6.24E-11 | A | T | 0.0034 | 95.942 |
| rs249677 | -0.0109 | 0.6333 | 2.40E-08 | A | C | 0.002 | 34.682 |
| rs2521349 | 0.0155 | 0.3847 | 2.01E-15 | A | G | 0.0019 | 24.847 |
| rs2529090 | 0.0136 | 0.181 | 3.39E-08 | G | C | 0.0025 | 51.215 |
| rs2545339 | 0.0115 | 0.6288 | 3.48E-09 | G | A | 0.002 | 24.691 |
| rs2569888 | 0.0133 | 0.2449 | 2.29E-09 | A | G | 0.0022 | 27.798 |
| rs2578565 | -0.0141 | 0.6575 | 1.37E-12 | T | C | 0.002 | 29.458 |
| rs2592208 | -0.0124 | 0.513 | 5.52E-11 | A | C | 0.0019 | 40.319 |
| rs2607234 | -0.0302 | 0.9476 | 2.00E-12 | G | A | 0.0043 | 34.594 |
| rs261223 | 0.0175 | 0.3702 | 2.30E-19 | C | A | 0.0019 | 40.784 |
| rs2648725 | 0.0165 | 0.2134 | 8.20E-13 | A | T | 0.0023 | 64.306 |
| rs2663126 | -0.0139 | 0.6905 | 1.36E-11 | A | G | 0.0021 | 41.156 |
| rs2676298 | -0.0269 | 0.853 | 2.39E-23 | T | C | 0.0027 | 37.185 |
| rs2717008 | -0.0127 | 0.3835 | 4.99E-11 | C | T | 0.0019 | 81.719 |
| rs2748501 | -0.0195 | 0.4395 | 1.31E-24 | A | G | 0.0019 | 34.341 |
| rs2754255 | -0.0153 | 0.2245 | 1.05E-11 | G | A | 0.0023 | 84.365 |
| rs2763263 | -0.017 | 0.2445 | 1.37E-14 | A | T | 0.0022 | 36.702 |
| rs2788213 | 0.0123 | 0.7104 | 3.80E-09 | A | G | 0.0021 | 48.076 |
| rs2789365 | -0.0145 | 0.4807 | 1.14E-14 | T | C | 0.0019 | 28.029 |
| rs2807339 | 0.0162 | 0.7586 | 1.24E-13 | C | T | 0.0022 | 47.266 |
| rs2812208 | 0.1156 | 0.021 | 5.51E-68 | C | G | 0.0066 | 43.281 |
| rs28379706 | 0.0114 | 0.3931 | 4.49E-09 | C | T | 0.002 | 247.532 |
| rs28485212 | -0.0188 | 0.1494 | 1.24E-12 | T | C | 0.0027 | 27.921 |
| rs28529055 | -0.0147 | 0.4368 | 1.88E-14 | T | G | 0.0019 | 40.449 |
| rs28529426 | -0.0168 | 0.1711 | 5.82E-11 | T | C | 0.0026 | 47.874 |
| rs28592876 | 0.03 | 0.2051 | 9.09E-38 | A | G | 0.0023 | 36.048 |
| rs2871865 | -0.0493 | 0.1159 | 3.40E-62 | G | C | 0.003 | 132.167 |
| rs2871960 | 0.0469 | 0.4448 | 2.17E-135 | C | A | 0.0019 | 224.373 |
| rs28736838 | -0.0117 | 0.3017 | 1.07E-08 | T | C | 0.002 | 489.674 |
| rs291979 | 0.0242 | 0.2289 | 6.76E-27 | A | G | 0.0023 | 25.971 |
| rs2923411 | 0.0127 | 0.5946 | 4.77E-11 | C | T | 0.0019 | 93.101 |
| rs2925155 | -0.015 | 0.2612 | 5.47E-12 | T | C | 0.0022 | 35.013 |
| rs293517 | -0.013 | 0.7011 | 2.80E-10 | C | T | 0.0021 | 39.102 |
| rs2971857 | -0.0119 | 0.5752 | 3.70E-10 | A | G | 0.0019 | 31.893 |
| rs2978362 | 0.0106 | 0.5326 | 2.85E-08 | T | C | 0.0019 | 31.160 |
| rs301807 | -0.0144 | 0.5815 | 2.53E-14 | G | A | 0.0019 | 25.188 |
| rs3103223 | 0.0126 | 0.7397 | 6.02E-09 | C | T | 0.0022 | 45.445 |
| rs310796 | 0.0142 | 0.6811 | 2.53E-12 | T | G | 0.002 | 27.528 |
| rs3116194 | -0.0295 | 0.0978 | 8.29E-21 | A | T | 0.0032 | 39.442 |
| rs3116602 | -0.0612 | 0.2153 | 9.53E-155 | G | T | 0.0023 | 69.156 |
| rs31196 | -0.0107 | 0.5724 | 2.07E-08 | A | C | 0.0019 | 570.526 |
| rs3205136 | -0.0184 | 0.0952 | 1.85E-08 | A | C | 0.0033 | 25.235 |
| rs331917 | -0.0127 | 0.5803 | 3.54E-11 | G | A | 0.0019 | 26.262 |
| rs332116 | -0.0206 | 0.2804 | 2.89E-22 | T | C | 0.0021 | 35.376 |
| rs336630 | -0.0106 | 0.43 | 2.90E-08 | T | C | 0.0019 | 77.118 |
| rs33973388 | 0.0249 | 0.4353 | 1.45E-38 | T | G | 0.0019 | 24.800 |
| rs34287 | 0.0187 | 0.341 | 1.17E-20 | A | G | 0.002 | 137.282 |
| rs34312629 | -0.017 | 0.2613 | 2.12E-15 | G | C | 0.0021 | 70.773 |
| rs34338597 | -0.0112 | 0.3832 | 7.88E-09 | G | A | 0.0019 | 50.238 |
| rs34345560 | 0.0219 | 0.1952 | 7.10E-20 | A | G | 0.0024 | 26.700 |
| rs34390533 | -0.0257 | 0.248 | 6.69E-32 | A | C | 0.0022 | 67.857 |
| rs34522021 | 0.0126 | 0.4547 | 3.38E-11 | T | C | 0.0019 | 110.948 |
| rs34776209 | -0.0317 | 0.2475 | 1.78E-47 | T | C | 0.0022 | 35.450 |
| rs34879158 | -0.0363 | 0.263 | 1.55E-63 | C | A | 0.0022 | 168.592 |
| rs35073631 | 0.0112 | 0.4331 | 5.92E-09 | C | T | 0.0019 | 230.109 |
| rs350832 | -0.0165 | 0.7709 | 3.44E-13 | A | G | 0.0023 | 27.735 |
| rs35288270 | -0.0328 | 0.1344 | 3.43E-32 | C | T | 0.0028 | 43.302 |
| rs35732917 | 0.0204 | 0.2843 | 2.08E-22 | C | T | 0.0021 | 112.732 |
| rs35756741 | -0.0378 | 0.0924 | 5.80E-31 | T | C | 0.0033 | 76.264 |
| rs35811052 | -0.0148 | 0.2559 | 8.84E-12 | G | A | 0.0022 | 107.927 |
| rs35816944 | -0.1088 | 0.0066 | 1.27E-20 | A | G | 0.0117 | 37.561 |
| rs35963161 | -0.0157 | 0.4757 | 7.46E-16 | A | G | 0.0019 | 69.899 |
| rs36000545 | -0.022 | 0.3957 | 2.56E-29 | G | A | 0.002 | 55.366 |
| rs36012032 | 0.0298 | 0.091 | 9.93E-20 | A | C | 0.0033 | 104.241 |
| rs36048468 | 0.0254 | 0.2088 | 9.34E-28 | T | C | 0.0023 | 66.157 |
| rs36226649 | 0.0485 | 0.0667 | 3.05E-37 | C | T | 0.0038 | 95.996 |
| rs3768495 | -0.0178 | 0.7173 | 1.07E-17 | T | C | 0.0021 | 131.896 |
| rs3769598 | 0.0171 | 0.1438 | 1.32E-10 | G | A | 0.0027 | 57.863 |
| rs377599 | 0.0217 | 0.383 | 3.50E-29 | T | C | 0.0019 | 32.421 |
| rs3778858 | 0.0108 | 0.3698 | 4.26E-08 | T | G | 0.002 | 100.225 |
| rs3782232 | -0.0339 | 0.0712 | 2.41E-20 | A | G | 0.0037 | 24.479 |
| rs3782811 | -0.0165 | 0.2507 | 3.96E-14 | A | C | 0.0022 | 68.445 |
| rs3792819 | 0.021 | 0.0854 | 4.42E-10 | G | A | 0.0034 | 46.057 |
| rs3818416 | 0.0279 | 0.7645 | 2.01E-35 | C | A | 0.0022 | 31.019 |
| rs3822742 | 0.0162 | 0.3706 | 1.03E-16 | A | C | 0.002 | 126.233 |
| rs3828729 | -0.016 | 0.3094 | 4.67E-15 | G | A | 0.002 | 55.130 |
| rs395980 | -0.0184 | 0.2635 | 1.02E-17 | G | T | 0.0021 | 49.262 |
| rs40270 | 0.0151 | 0.7724 | 1.90E-11 | C | A | 0.0022 | 59.173 |
| rs4073154 | 0.0274 | 0.7784 | 1.92E-33 | G | A | 0.0023 | 36.098 |
| rs4076108 | 0.0174 | 0.2449 | 2.30E-15 | T | A | 0.0022 | 116.644 |
| rs4077103 | -0.0143 | 0.8426 | 4.18E-08 | A | C | 0.0026 | 50.421 |
| rs4121583 | 0.0118 | 0.6192 | 4.62E-09 | T | C | 0.002 | 24.423 |
| rs41311445 | -0.0328 | 0.0958 | 4.73E-24 | C | A | 0.0032 | 29.566 |
| rs4244809 | -0.0262 | 0.2082 | 5.55E-29 | A | G | 0.0023 | 83.933 |
| rs4252548 | -0.0753 | 0.0218 | 2.96E-31 | T | C | 0.0065 | 101.923 |
| rs4274112 | -0.0217 | 0.3733 | 2.47E-28 | G | A | 0.002 | 108.907 |
| rs4282339 | -0.0311 | 0.2075 | 6.16E-41 | A | G | 0.0023 | 99.222 |
| rs4287835 | 0.0147 | 0.534 | 9.94E-15 | C | T | 0.0019 | 143.269 |
| rs4380799 | -0.0255 | 0.39 | 6.45E-33 | G | T | 0.0021 | 48.427 |
| rs4383083 | 0.0111 | 0.6466 | 2.91E-08 | A | G | 0.002 | 139.343 |
| rs447352 | -0.0181 | 0.1408 | 6.62E-10 | T | C | 0.0029 | 25.354 |
| rs4504126 | 0.046 | 0.0275 | 1.62E-15 | C | A | 0.0058 | 35.691 |
| rs45474992 | -0.0617 | 0.0362 | 2.17E-33 | T | C | 0.0051 | 50.964 |
| rs45528934 | 0.0262 | 0.1623 | 1.97E-24 | T | C | 0.0026 | 119.634 |
| rs4602848 | 0.016 | 0.6812 | 3.14E-15 | G | A | 0.002 | 84.055 |
| rs4622329 | 0.0149 | 0.3496 | 8.62E-14 | A | G | 0.002 | 50.068 |
| rs4640244 | -0.02 | 0.3993 | 3.81E-25 | G | A | 0.0019 | 45.461 |
| rs4644481 | -0.0112 | 0.4328 | 3.54E-09 | T | C | 0.0019 | 86.412 |
| rs4655345 | -0.0246 | 0.3974 | 5.81E-38 | G | A | 0.0019 | 27.731 |
| rs4682483 | -0.0165 | 0.1534 | 2.65E-10 | A | G | 0.0026 | 130.535 |
| rs4683435 | 0.0144 | 0.7723 | 1.60E-10 | G | A | 0.0022 | 31.840 |
| rs4735761 | 0.0331 | 0.2857 | 3.66E-56 | C | A | 0.0021 | 32.838 |
| rs4748008 | -0.0125 | 0.4346 | 8.95E-11 | C | T | 0.0019 | 201.426 |
| rs4752689 | 0.0205 | 0.5839 | 1.36E-26 | A | G | 0.0019 | 34.576 |
| rs4752829 | 0.0262 | 0.2861 | 5.90E-36 | A | G | 0.0021 | 91.962 |
| rs4788218 | 0.0275 | 0.401 | 5.52E-46 | C | T | 0.0019 | 126.286 |
| rs4807472 | -0.0158 | 0.6764 | 8.18E-15 | C | T | 0.002 | 163.632 |
| rs4815952 | -0.0161 | 0.5221 | 1.24E-16 | C | T | 0.0019 | 49.209 |
| rs4818280 | -0.0124 | 0.6269 | 2.84E-10 | T | C | 0.002 | 58.247 |
| rs4847378 | 0.0136 | 0.6121 | 1.64E-12 | T | G | 0.0019 | 32.387 |
| rs4852257 | -0.0231 | 0.5763 | 6.21E-34 | G | T | 0.0019 | 39.549 |
| rs4865956 | -0.0258 | 0.6965 | 3.85E-36 | A | T | 0.0021 | 117.360 |
| rs4870941 | -0.0297 | 0.238 | 1.09E-39 | C | G | 0.0023 | 126.741 |
| rs488621 | 0.0191 | 0.4688 | 2.86E-24 | G | A | 0.0019 | 144.098 |
| rs4932439 | -0.0151 | 0.8254 | 1.43E-09 | G | A | 0.0025 | 81.821 |
| rs4938359 | -0.0156 | 0.2024 | 3.24E-11 | G | A | 0.0024 | 29.591 |
| rs4940874 | 0.0148 | 0.8119 | 1.24E-09 | G | A | 0.0024 | 35.380 |
| rs4965298 | -0.0119 | 0.7127 | 1.81E-08 | T | C | 0.0021 | 30.124 |
| rs496783 | -0.0124 | 0.4648 | 8.13E-11 | G | A | 0.0019 | 26.112 |
| rs4976262 | -0.0245 | 0.3156 | 4.37E-33 | C | T | 0.002 | 34.446 |
| rs532499 | -0.0127 | 0.7413 | 4.90E-09 | C | T | 0.0022 | 116.780 |
| rs543650 | 0.025 | 0.5987 | 1.49E-37 | G | T | 0.002 | 27.855 |
| rs544136 | 0.0121 | 0.7482 | 2.50E-08 | G | T | 0.0022 | 135.258 |
| rs545104 | 0.0127 | 0.6164 | 8.08E-11 | C | T | 0.002 | 24.840 |
| rs55717234 | 0.0122 | 0.5732 | 1.34E-10 | G | A | 0.0019 | 34.344 |
| rs55758152 | 0.0145 | 0.3263 | 1.05E-12 | A | G | 0.002 | 32.791 |
| rs55852614 | -0.0393 | 0.2474 | 3.29E-73 | C | T | 0.0022 | 41.623 |
| rs55872725 | 0.0222 | 0.4037 | 1.46E-30 | T | C | 0.0019 | 259.104 |
| rs56112295 | 0.0154 | 0.2257 | 1.12E-10 | T | C | 0.0024 | 106.858 |
| rs56207600 | 0.0192 | 0.111 | 2.50E-10 | A | G | 0.003 | 37.324 |
| rs56239180 | -0.0459 | 0.0237 | 1.85E-13 | G | T | 0.0062 | 32.759 |
| rs56363908 | -0.0382 | 0.0422 | 3.88E-16 | G | A | 0.0047 | 43.901 |
| rs568267 | 0.0122 | 0.7431 | 2.23E-08 | T | C | 0.0022 | 53.118 |
| rs57059662 | 0.0118 | 0.6774 | 5.63E-09 | C | T | 0.002 | 25.588 |
| rs5742915 | 0.0248 | 0.4605 | 9.33E-39 | C | T | 0.0019 | 27.402 |
| rs57513571 | -0.0191 | 0.2004 | 7.02E-16 | T | C | 0.0024 | 137.636 |
| rs5753518 | 0.0242 | 0.0899 | 5.04E-13 | A | G | 0.0033 | 52.646 |
| rs57696574 | 0.0173 | 0.3981 | 4.50E-18 | C | A | 0.002 | 43.152 |
| rs577289 | -0.0125 | 0.2808 | 5.39E-09 | T | A | 0.0021 | 64.587 |
| rs57791149 | -0.0173 | 0.4024 | 3.26E-19 | C | T | 0.0019 | 28.416 |
| rs599004 | -0.0157 | 0.2807 | 6.53E-14 | T | C | 0.0021 | 64.818 |
| rs59950280 | -0.0254 | 0.3316 | 7.32E-36 | A | G | 0.002 | 44.820 |
| rs6000886 | 0.0131 | 0.6482 | 5.88E-11 | C | T | 0.002 | 128.801 |
| rs6028716 | -0.021 | 0.2587 | 4.58E-22 | A | G | 0.0022 | 35.242 |
| rs60389750 | -0.0175 | 0.3139 | 1.06E-16 | T | C | 0.0021 | 76.169 |
| rs60408354 | 0.0259 | 0.0732 | 1.15E-12 | A | G | 0.0036 | 59.400 |
| rs6054390 | -0.0188 | 0.628 | 1.45E-21 | A | G | 0.002 | 40.984 |
| rs6054491 | -0.0142 | 0.2404 | 2.15E-10 | G | C | 0.0022 | 74.364 |
| rs6066122 | 0.0127 | 0.7631 | 1.76E-08 | G | C | 0.0023 | 33.159 |
| rs60804050 | -0.0217 | 0.2559 | 5.01E-24 | A | G | 0.0021 | 26.258 |
| rs6082354 | -0.024 | 0.6675 | 1.19E-32 | C | A | 0.002 | 80.756 |
| rs610694 | 0.0136 | 0.4903 | 4.29E-13 | C | T | 0.0019 | 115.147 |
| rs61397287 | 0.0235 | 0.0747 | 4.45E-11 | T | A | 0.0036 | 41.626 |
| rs6142059 | 0.0116 | 0.4926 | 1.19E-09 | C | T | 0.0019 | 34.375 |
| rs61528919 | 0.014 | 0.3609 | 3.33E-12 | C | T | 0.002 | 30.288 |
| rs61729527 | -0.0346 | 0.0519 | 4.86E-16 | T | C | 0.0043 | 40.712 |
| rs61732778 | 0.023 | 0.071 | 3.13E-10 | A | G | 0.0037 | 53.052 |
| rs61827272 | 0.0144 | 0.2754 | 7.89E-12 | C | T | 0.0021 | 31.422 |
| rs61878760 | 0.019 | 0.0828 | 3.74E-08 | A | G | 0.0034 | 37.265 |
| rs61919240 | 0.0137 | 0.3244 | 9.77E-12 | A | T | 0.002 | 24.689 |
| rs61944841 | 0.0253 | 0.4138 | 3.54E-37 | A | G | 0.002 | 37.044 |
| rs62033029 | -0.0141 | 0.2064 | 1.73E-09 | A | G | 0.0023 | 139.858 |
| rs62103240 | 0.0212 | 0.071 | 1.40E-08 | A | G | 0.0037 | 29.326 |
| rs62106258 | -0.0504 | 0.0486 | 6.45E-31 | C | T | 0.0044 | 26.696 |
| rs62143873 | -0.0115 | 0.5033 | 1.19E-09 | A | G | 0.0019 | 105.788 |
| rs62370472 | -0.0253 | 0.209 | 1.45E-27 | C | T | 0.0023 | 29.773 |
| rs62466110 | -0.0371 | 0.067 | 5.74E-20 | C | T | 0.0041 | 95.308 |
| rs62501195 | -0.0198 | 0.1712 | 8.11E-15 | C | A | 0.0025 | 77.491 |
| rs62515437 | 0.0369 | 0.2253 | 8.79E-60 | T | G | 0.0023 | 50.096 |
| rs62621812 | 0.0743 | 0.0204 | 3.16E-27 | A | G | 0.0069 | 214.106 |
| rs6425817 | 0.0157 | 0.6724 | 2.98E-15 | G | A | 0.002 | 99.364 |
| rs6470771 | -0.0268 | 0.1694 | 1.54E-26 | C | A | 0.0025 | 48.898 |
| rs6502935 | -0.0125 | 0.7381 | 6.31E-09 | T | C | 0.0022 | 91.020 |
| rs650508 | -0.013 | 0.3008 | 1.86E-10 | C | G | 0.002 | 27.200 |
| rs6505216 | -0.0498 | 0.233 | 1.83E-101 | T | G | 0.0023 | 32.009 |
| rs6543146 | 0.0154 | 0.5579 | 4.19E-16 | G | T | 0.0019 | 399.457 |
| rs655113 | 0.0188 | 0.3006 | 7.42E-20 | C | T | 0.0021 | 52.680 |
| rs6570509 | -0.0244 | 0.2868 | 1.27E-31 | T | G | 0.0021 | 66.922 |
| rs6582398 | 0.014 | 0.6002 | 1.14E-12 | T | C | 0.002 | 109.686 |
| rs6593210 | 0.0146 | 0.2081 | 4.88E-10 | A | G | 0.0024 | 42.356 |
| rs664317 | -0.0177 | 0.8375 | 4.50E-12 | C | A | 0.0026 | 31.634 |
| rs6675858 | -0.0137 | 0.2141 | 2.40E-09 | T | C | 0.0023 | 38.397 |
| rs6693481 | -0.0143 | 0.6951 | 2.21E-12 | C | T | 0.002 | 28.440 |
| rs670318 | 0.0413 | 0.9515 | 2.52E-21 | C | T | 0.0044 | 39.029 |
| rs6721191 | -0.0144 | 0.5779 | 3.17E-14 | G | A | 0.0019 | 70.891 |
| rs6738207 | 0.0127 | 0.4007 | 4.14E-11 | A | G | 0.0019 | 45.552 |
| rs6739278 | -0.021 | 0.8079 | 1.27E-18 | C | T | 0.0024 | 34.880 |
| rs67527161 | -0.0182 | 0.209 | 5.79E-15 | C | T | 0.0023 | 61.639 |
| rs67551338 | 0.0576 | 0.0615 | 1.04E-47 | T | C | 0.004 | 49.316 |
| rs6762851 | -0.0218 | 0.3573 | 1.50E-28 | C | T | 0.002 | 172.503 |
| rs67716382 | 0.0226 | 0.2214 | 1.65E-23 | C | G | 0.0023 | 98.293 |
| rs68049170 | -0.0259 | 0.2764 | 2.69E-34 | A | G | 0.0021 | 79.298 |
| rs680882 | 0.0133 | 0.7606 | 1.98E-09 | G | T | 0.0022 | 120.845 |
| rs6821305 | 0.0204 | 0.399 | 3.15E-26 | C | A | 0.0019 | 29.006 |
| rs684905 | -0.0118 | 0.4176 | 7.10E-10 | T | C | 0.0019 | 89.881 |
| rs6860245 | 0.0589 | 0.248 | 9.66E-160 | C | G | 0.0022 | 30.497 |
| rs6874142 | 0.0288 | 0.1138 | 5.15E-20 | G | T | 0.0031 | 583.361 |
| rs6902109 | -0.0167 | 0.5401 | 1.03E-18 | G | A | 0.0019 | 75.337 |
| rs6931421 | -0.0279 | 0.3224 | 2.31E-43 | G | T | 0.002 | 62.389 |
| rs6977416 | 0.0457 | 0.3338 | 1.43E-113 | A | G | 0.002 | 153.179 |
| rs700677 | 0.0173 | 0.3505 | 1.13E-18 | A | C | 0.002 | 418.603 |
| rs7007389 | -0.0134 | 0.3727 | 2.94E-11 | A | T | 0.002 | 61.361 |
| rs7014590 | -0.0228 | 0.2614 | 4.48E-26 | C | T | 0.0022 | 37.806 |
| rs7020491 | -0.0178 | 0.4266 | 1.22E-20 | T | C | 0.0019 | 90.395 |
| rs702886 | 0.012 | 0.3505 | 1.11E-09 | G | A | 0.002 | 69.801 |
| rs704660 | 0.0153 | 0.4095 | 2.28E-15 | T | C | 0.0019 | 29.521 |
| rs7082659 | 0.0156 | 0.8663 | 2.27E-08 | C | T | 0.0028 | 50.978 |
| rs7095472 | 0.0267 | 0.5335 | 7.66E-45 | G | A | 0.0019 | 25.383 |
| rs7129320 | -0.0389 | 0.1661 | 7.29E-53 | A | G | 0.0025 | 159.822 |
| rs713467 | 0.0146 | 0.4345 | 3.09E-14 | A | G | 0.0019 | 188.816 |
| rs71414738 | 0.015 | 0.1763 | 1.00E-09 | T | C | 0.0025 | 47.168 |
| rs71635721 | 0.0316 | 0.0649 | 3.46E-16 | G | C | 0.0039 | 29.424 |
| rs7185244 | -0.0148 | 0.7759 | 1.40E-10 | C | T | 0.0023 | 54.576 |
| rs718603 | 0.0131 | 0.2774 | 6.68E-10 | T | C | 0.0021 | 34.299 |
| rs7220127 | -0.0105 | 0.4264 | 4.60E-08 | C | T | 0.0019 | 30.978 |
| rs7228151 | -0.0185 | 0.206 | 3.19E-15 | C | T | 0.0023 | 24.283 |
| rs7229520 | -0.0224 | 0.6616 | 9.21E-29 | A | G | 0.002 | 50.414 |
| rs723149 | -0.0276 | 0.5628 | 1.43E-47 | G | A | 0.0019 | 101.180 |
| rs72656010 | -0.0668 | 0.1322 | 7.31E-126 | C | T | 0.0028 | 168.846 |
| rs72657800 | -0.0219 | 0.0773 | 6.39E-10 | C | T | 0.0035 | 461.449 |
| rs72695791 | -0.0297 | 0.036 | 4.58E-09 | G | C | 0.0051 | 30.806 |
| rs72721979 | -0.0229 | 0.143 | 2.20E-17 | G | T | 0.0027 | 27.567 |
| rs72726050 | -0.0192 | 0.0958 | 1.84E-08 | C | T | 0.0034 | 57.879 |
| rs72771070 | 0.015 | 0.283 | 1.30E-12 | T | C | 0.0021 | 28.756 |
| rs72801843 | 0.0313 | 0.3015 | 8.83E-52 | A | T | 0.0021 | 41.115 |
| rs72809820 | -0.0111 | 0.324 | 3.13E-08 | T | C | 0.002 | 185.865 |
| rs72829852 | 0.0309 | 0.0621 | 3.74E-15 | T | C | 0.0039 | 24.302 |
| rs7286917 | 0.0171 | 0.7461 | 5.18E-14 | G | A | 0.0023 | 50.083 |
| rs72894003 | -0.0423 | 0.0647 | 1.90E-28 | T | C | 0.0038 | 49.886 |
| rs73006226 | -0.0182 | 0.1267 | 2.23E-10 | A | C | 0.0029 | 97.523 |
| rs7301341 | -0.0255 | 0.3268 | 9.27E-37 | C | T | 0.002 | 33.006 |
| rs73052033 | -0.0151 | 0.1851 | 4.79E-10 | C | T | 0.0024 | 128.856 |
| rs73125634 | -0.0195 | 0.2782 | 5.11E-20 | T | G | 0.0021 | 30.972 |
| rs73197345 | 0.0211 | 0.1367 | 3.55E-14 | A | T | 0.0028 | 68.768 |
| rs7320878 | -0.015 | 0.6034 | 1.34E-14 | A | G | 0.0019 | 47.317 |
| rs7321635 | -0.0132 | 0.6465 | 2.58E-11 | C | A | 0.002 | 48.491 |
| rs7328187 | 0.0116 | 0.4976 | 1.19E-09 | G | T | 0.0019 | 35.860 |
| rs73384223 | -0.0205 | 0.1967 | 1.27E-17 | C | T | 0.0024 | 30.294 |
| rs73413540 | -0.0124 | 0.2248 | 4.42E-08 | T | C | 0.0023 | 59.803 |
| rs7367519 | 0.0164 | 0.6846 | 4.68E-16 | C | T | 0.002 | 24.130 |
| rs73696333 | 0.0191 | 0.2012 | 3.20E-15 | G | C | 0.0024 | 52.301 |
| rs73856768 | -0.0247 | 0.0805 | 1.55E-12 | C | T | 0.0035 | 52.803 |
| rs7418410 | 0.0155 | 0.4089 | 5.64E-16 | T | C | 0.0019 | 40.668 |
| rs74379684 | -0.0272 | 0.0751 | 4.39E-14 | T | C | 0.0036 | 52.296 |
| rs74458759 | 0.0171 | 0.2886 | 3.12E-15 | G | C | 0.0022 | 46.280 |
| rs7448554 | -0.0132 | 0.4999 | 1.77E-11 | A | C | 0.002 | 54.067 |
| rs74494415 | -0.0417 | 0.0398 | 1.82E-17 | T | C | 0.0049 | 39.228 |
| rs7485647 | -0.0261 | 0.844 | 1.04E-23 | A | G | 0.0026 | 59.848 |
| rs75022676 | -0.0163 | 0.2079 | 2.84E-12 | A | G | 0.0023 | 80.779 |
| rs7522400 | 0.0129 | 0.7683 | 5.62E-09 | G | A | 0.0022 | 39.402 |
| rs7543136 | -0.021 | 0.7209 | 9.96E-24 | T | C | 0.0021 | 26.677 |
| rs75508358 | 0.0266 | 0.0457 | 4.29E-09 | T | C | 0.0045 | 79.914 |
| rs7563362 | 0.0352 | 0.8566 | 3.27E-39 | G | A | 0.0027 | 27.789 |
| rs7570235 | -0.0168 | 0.5911 | 2.08E-18 | C | T | 0.0019 | 137.094 |
| rs75702986 | -0.0163 | 0.1861 | 3.14E-11 | A | G | 0.0025 | 61.437 |
| rs7598430 | -0.016 | 0.5046 | 1.37E-17 | T | C | 0.0019 | 36.241 |
| rs7610055 | -0.0373 | 0.1207 | 3.55E-38 | A | G | 0.0029 | 57.633 |
| rs7633464 | 0.0175 | 0.4786 | 1.27E-20 | A | G | 0.0019 | 133.004 |
| rs76364830 | -0.0471 | 0.0634 | 2.70E-33 | A | G | 0.0039 | 68.827 |
| rs76517946 | -0.0368 | 0.0814 | 1.69E-26 | A | C | 0.0035 | 118.652 |
| rs7679276 | -0.033 | 0.9544 | 5.93E-12 | G | A | 0.0048 | 91.203 |
| rs7689420 | 0.0466 | 0.8312 | 1.50E-76 | C | T | 0.0025 | 42.681 |
| rs7701233 | -0.0179 | 0.4283 | 5.12E-21 | C | T | 0.0019 | 274.530 |
| rs77013652 | 0.049 | 0.015 | 1.50E-09 | G | T | 0.0081 | 70.659 |
| rs772222 | 0.0121 | 0.2651 | 1.55E-08 | G | A | 0.0021 | 31.947 |
| rs7731023 | 0.0166 | 0.5749 | 3.48E-18 | G | A | 0.0019 | 25.687 |
| rs7735891 | 0.0259 | 0.4629 | 1.14E-42 | T | C | 0.0019 | 60.650 |
| rs77364196 | -0.033 | 0.053 | 8.35E-15 | A | G | 0.0043 | 150.232 |
| rs77447813 | 0.0224 | 0.087 | 3.21E-11 | C | G | 0.0034 | 49.224 |
| rs7768382 | -0.0201 | 0.4771 | 1.57E-26 | C | T | 0.0019 | 35.892 |
| rs77809369 | 0.0237 | 0.0642 | 9.83E-10 | T | C | 0.0039 | 90.778 |
| rs78051210 | 0.0263 | 0.0771 | 1.63E-13 | C | T | 0.0036 | 30.389 |
| rs7816345 | 0.0255 | 0.1682 | 6.22E-24 | T | C | 0.0025 | 44.324 |
| rs781669 | 0.0164 | 0.5236 | 3.14E-18 | T | C | 0.0019 | 81.937 |
| rs7826059 | 0.0114 | 0.6421 | 7.61E-09 | C | T | 0.002 | 60.422 |
| rs7828086 | 0.0135 | 0.2391 | 1.11E-09 | C | T | 0.0022 | 26.895 |
| rs78378222 | 0.138 | 0.0123 | 4.51E-56 | G | T | 0.0087 | 29.859 |
| rs78457529 | -0.0904 | 0.0117 | 1.22E-24 | T | C | 0.0088 | 208.432 |
| rs7858712 | 0.0347 | 0.9144 | 1.04E-24 | G | A | 0.0034 | 85.108 |
| rs78766798 | 0.0319 | 0.0847 | 2.61E-20 | C | T | 0.0035 | 84.884 |
| rs7893378 | 0.0175 | 0.1143 | 2.36E-08 | A | G | 0.0031 | 71.051 |
| rs7902 | 0.0149 | 0.4469 | 5.22E-15 | G | A | 0.0019 | 27.920 |
| rs7941305 | -0.0129 | 0.3133 | 6.01E-10 | C | T | 0.0021 | 49.421 |
| rs79441499 | -0.0138 | 0.4055 | 9.67E-13 | T | C | 0.0019 | 32.241 |
| rs7952436 | -0.0453 | 0.0823 | 1.62E-39 | T | C | 0.0034 | 41.344 |
| rs798548 | -0.0359 | 0.3006 | 2.86E-68 | C | T | 0.0021 | 139.607 |
| rs8000973 | 0.0134 | 0.5334 | 2.05E-12 | C | T | 0.0019 | 244.126 |
| rs80132799 | 0.0231 | 0.0679 | 1.31E-09 | T | C | 0.0038 | 40.246 |
| rs8017006 | 0.0122 | 0.3275 | 2.22E-09 | G | A | 0.002 | 30.413 |
| rs8018486 | -0.0138 | 0.1914 | 1.18E-08 | G | A | 0.0024 | 29.521 |
| rs8019890 | 0.025 | 0.5313 | 1.96E-38 | A | C | 0.0019 | 26.542 |
| rs8020095 | -0.0145 | 0.15 | 4.53E-08 | A | G | 0.0027 | 140.193 |
| rs80280630 | -0.0168 | 0.1115 | 2.28E-08 | T | C | 0.003 | 24.140 |
| rs80295797 | -0.0198 | 0.3265 | 3.83E-23 | T | C | 0.002 | 25.180 |
| rs8042578 | 0.0287 | 0.2426 | 2.29E-38 | G | C | 0.0022 | 77.643 |
| rs8054549 | -0.0251 | 0.4486 | 3.37E-39 | A | C | 0.0019 | 136.328 |
| rs8084413 | -0.0127 | 0.4693 | 3.25E-11 | A | G | 0.0019 | 140.373 |
| rs8136517 | 0.0267 | 0.0652 | 6.58E-12 | C | T | 0.0039 | 36.176 |
| rs822530 | 0.0255 | 0.7953 | 2.36E-27 | T | A | 0.0024 | 39.129 |
| rs839255 | -0.0126 | 0.6847 | 8.38E-10 | G | T | 0.0021 | 95.345 |
| rs867529 | 0.0184 | 0.2796 | 1.00E-18 | C | G | 0.0021 | 30.865 |
| rs876122 | 0.0162 | 0.8788 | 2.19E-08 | G | A | 0.0029 | 61.416 |
| rs8904 | -0.0157 | 0.363 | 1.52E-15 | A | G | 0.002 | 25.172 |
| rs900399 | 0.0164 | 0.3986 | 1.35E-17 | G | A | 0.0019 | 51.330 |
| rs905938 | 0.0394 | 0.2649 | 8.43E-77 | C | T | 0.0021 | 58.066 |
| rs909220 | -0.015 | 0.4443 | 3.24E-15 | A | G | 0.0019 | 272.370 |
| rs9266244 | -0.0427 | 0.7077 | 1.21E-94 | A | G | 0.0021 | 50.029 |
| rs9344126 | -0.0185 | 0.5137 | 2.21E-22 | C | T | 0.0019 | 339.889 |
| rs9375188 | 0.0136 | 0.4844 | 6.80E-13 | T | C | 0.0019 | 77.003 |
| rs9385002 | -0.0147 | 0.2394 | 3.26E-11 | T | A | 0.0022 | 41.602 |
| rs9388490 | 0.0462 | 0.4394 | 1.33E-130 | T | C | 0.0019 | 35.434 |
| rs947099 | 0.0117 | 0.3536 | 2.71E-09 | A | G | 0.002 | 473.946 |
| rs951366 | 0.0205 | 0.3932 | 9.15E-27 | C | T | 0.0019 | 28.177 |
| rs9517483 | -0.0181 | 0.699 | 2.26E-18 | G | A | 0.0021 | 90.309 |
| rs9525326 | -0.0184 | 0.1875 | 3.62E-14 | G | A | 0.0024 | 62.078 |
| rs9568031 | -0.0115 | 0.706 | 3.34E-08 | T | C | 0.0021 | 46.449 |
| rs9590328 | 0.0153 | 0.1417 | 2.02E-08 | G | A | 0.0027 | 24.720 |
| rs9594714 | 0.0144 | 0.3052 | 2.65E-12 | T | G | 0.0021 | 25.638 |
| rs9610447 | 0.0152 | 0.7471 | 5.29E-12 | T | C | 0.0022 | 39.599 |
| rs9634212 | 0.0471 | 0.221 | 8.59E-95 | A | C | 0.0023 | 39.312 |
| rs9636364 | 0.011 | 0.5429 | 5.09E-09 | A | G | 0.0019 | 344.174 |
| rs9669278 | -0.0496 | 0.5183 | 5.25E-151 | C | T | 0.0019 | 27.041 |
| rs9809116 | -0.016 | 0.4081 | 1.31E-16 | G | A | 0.0019 | 553.771 |
| rs9828525 | 0.0121 | 0.4113 | 2.51E-10 | T | C | 0.0019 | 55.691 |
| rs9832919 | -0.0179 | 0.3574 | 7.90E-20 | G | A | 0.002 | 31.925 |
| rs9838614 | -0.0185 | 0.3881 | 1.21E-21 | G | T | 0.0019 | 66.274 |
| rs987666 | 0.0185 | 0.1188 | 2.33E-10 | A | G | 0.0029 | 73.200 |
| rs9890062 | 0.0267 | 0.0615 | 1.20E-11 | A | G | 0.0039 | 32.266 |
| rs9894577 | -0.031 | 0.318 | 1.40E-52 | A | G | 0.002 | 37.055 |
| rs9898189 | -0.0163 | 0.6446 | 1.88E-15 | G | C | 0.0021 | 187.755 |
| rs990315 | -0.0115 | 0.6229 | 5.07E-09 | C | T | 0.002 | 54.816 |
| rs9905385 | -0.0339 | 0.6707 | 1.94E-63 | G | A | 0.002 | 27.975 |
| rs9957318 | 0.0187 | 0.348 | 1.02E-20 | G | A | 0.002 | 228.673 |

SNP, single nucleotide polymorphism; Beta coefficients are in standard deviation (SD) unit per allele; EAF, effect allele frequency; SE, standard error;

**Supplementary Table 9.** Characteristics of selected SNPs concerning the causal effect of left-hand grip strength on CHD

| **SNP** | **Beta** | **EAF** | ***P*-value** | **Effect**  **allele** | **Other**  **allele** | **SE** | ***F*-value** |
| --- | --- | --- | --- | --- | --- | --- | --- |
| rs10097417 | -0.0132302 | 0.170904 | 1.90E-11 | G | A | 0.00197055 | 22.8699 |
| rs10144445 | -0.00936876 | 0.350118 | 1.90E-09 | G | C | 0.00155918 | 18.4155 |
| rs10176878 | -0.0129478 | 0.190861 | 8.60E-12 | C | T | 0.0018962 | 23.8731 |
| rs10205394 | -0.0113217 | 0.200728 | 1.10E-09 | C | G | 0.00185667 | 18.9626 |
| rs10403906 | -0.0100323 | 0.476375 | 1.50E-11 | A | G | 0.00148665 | 23.1497 |
| rs1044299 | 0.01401 | 0.546046 | 6.60E-21 | T | C | 0.00149362 | 44.8656 |
| rs10786706 | 0.0100072 | 0.465745 | 1.70E-11 | T | C | 0.00148741 | 22.9772 |
| rs10788958 | 0.0141976 | 0.644753 | 1.00E-19 | G | C | 0.00156276 | 42.5743 |
| rs10821939 | -0.00935422 | 0.573342 | 4.90E-10 | A | G | 0.00150339 | 19.7370 |
| rs10831903 | 0.00928662 | 0.423383 | 8.10E-10 | T | C | 0.00151154 | 19.4137 |
| rs10934857 | 0.00928675 | 0.258913 | 4.80E-08 | A | G | 0.00170182 | 15.2587 |
| rs10988217 | -0.00920657 | 0.603742 | 1.70E-09 | G | A | 0.00152911 | 18.6980 |
| rs11002322 | -0.00998924 | 0.340459 | 2.10E-10 | T | G | 0.00157255 | 20.6607 |
| rs11003014 | 0.011433 | 0.161184 | 1.60E-08 | G | A | 0.00202143 | 16.2959 |
| rs11076004 | -0.0115356 | 0.418177 | 2.10E-14 | A | G | 0.00150909 | 29.8547 |
| rs11111267 | 0.0108359 | 0.18106 | 1.90E-08 | G | A | 0.00192886 | 16.0536 |
| rs11121542 | -0.0157601 | 0.122677 | 3.00E-12 | A | G | 0.00225916 | 24.6500 |
| rs11125803 | 0.0142851 | 0.740516 | 3.80E-17 | T | C | 0.00169673 | 36.1576 |
| rs11168357 | -0.00962864 | 0.246008 | 2.50E-08 | A | G | 0.00172661 | 15.8568 |
| rs11204664 | -0.00865184 | 0.578725 | 8.10E-09 | C | T | 0.00150032 | 16.8277 |
| rs11243202 | 0.00972237 | 0.486019 | 7.00E-11 | C | T | 0.0014909 | 21.7730 |
| rs112485536 | 0.0161779 | 0.075507 | 9.40E-09 | T | C | 0.00281803 | 16.8463 |
| rs113315602 | -0.0181886 | 0.095848 | 8.60E-12 | C | A | 0.00266345 | 26.4363 |
| rs113434679 | -0.0149156 | 0.199577 | 1.90E-15 | A | C | 0.00187618 | 32.7715 |
| rs113918482 | -0.0101011 | 0.222507 | 1.50E-08 | G | A | 0.00178515 | 16.2760 |
| rs116409670 | -0.0152418 | 0.079819 | 2.60E-08 | T | C | 0.00273867 | 15.7333 |
| rs11669079 | 0.0109542 | 0.705026 | 1.90E-11 | T | A | 0.00163169 | 23.0105 |
| rs11769549 | 0.0204956 | 0.062372 | 4.50E-11 | A | T | 0.00311228 | 22.6525 |
| rs12316046 | -0.0174165 | 0.37795 | 5.00E-30 | G | A | 0.00152991 | 65.7653 |
| rs12473732 | 0.0109869 | 0.486507 | 1.40E-13 | T | C | 0.00148601 | 27.8070 |
| rs12528131 | -0.00882788 | 0.488343 | 2.80E-09 | G | A | 0.00148565 | 17.9551 |
| rs12533765 | -0.0092 | 0.280054 | 2.60E-08 | G | A | 0.00165199 | 15.7357 |
| rs12673062 | -0.0107691 | 0.215756 | 2.60E-09 | A | G | 0.00180808 | 18.0944 |
| rs12790261 | -0.0251882 | 0.082383 | 1.20E-20 | A | C | 0.00270305 | 44.2271 |
| rs12906830 | 0.0108401 | 0.601065 | 9.30E-13 | C | T | 0.00151797 | 25.9817 |
| rs13091492 | -0.00847903 | 0.372833 | 3.30E-08 | G | A | 0.00153407 | 15.5009 |
| rs13106087 | 0.0116202 | 0.829801 | 3.90E-09 | C | T | 0.00197399 | 17.5844 |
| rs13146142 | -0.0202031 | 0.158623 | 2.30E-23 | C | T | 0.00202888 | 50.2335 |
| rs13227429 | -0.00858767 | 0.561343 | 9.80E-09 | C | T | 0.0014977 | 16.7445 |
| rs13337177 | -0.0142799 | 0.180836 | 1.60E-13 | T | G | 0.00193621 | 27.8539 |
| rs13356200 | -0.00876765 | 0.393874 | 9.70E-09 | G | T | 0.0015286 | 16.9222 |
| rs143002906 | 0.0262281 | 0.027829 | 9.80E-09 | T | C | 0.00457421 | 17.1610 |
| rs143384 | 0.0209202 | 0.404388 | 1.50E-43 | G | A | 0.00151159 | 97.2162 |
| rs1434095 | 0.0140301 | 0.875121 | 5.30E-10 | C | T | 0.00225906 | 19.8359 |
| rs1486925 | -0.0104734 | 0.314604 | 6.30E-11 | C | T | 0.00160211 | 21.8100 |
| rs150330307 | -0.0307753 | 0.031896 | 2.90E-13 | C | T | 0.0042167 | 26.9676 |
| rs1551042 | -0.0111279 | 0.647025 | 7.40E-13 | C | A | 0.00155167 | 26.0777 |
| rs1556659 | 0.0162896 | 0.38153 | 2.50E-26 | T | C | 0.00153452 | 57.7399 |
| rs1641457 | 0.0120456 | 0.223305 | 1.40E-11 | G | T | 0.0017829 | 23.2050 |
| rs16870531 | 0.011198 | 0.238492 | 1.30E-10 | T | C | 0.00174241 | 20.9992 |
| rs16910750 | 0.0112498 | 0.159639 | 3.60E-08 | C | G | 0.00204139 | 15.6553 |
| rs17282763 | 0.00893956 | 0.296281 | 4.40E-08 | C | T | 0.00163298 | 15.3640 |
| rs17466480 | -0.0118235 | 0.386968 | 9.80E-15 | G | A | 0.00152732 | 30.5797 |
| rs17630248 | -0.009184 | 0.347808 | 4.10E-09 | C | T | 0.00156162 | 17.6420 |
| rs181766 | 0.00963247 | 0.321793 | 1.90E-09 | C | T | 0.00160372 | 18.6718 |
| rs1884447 | 0.00846069 | 0.400566 | 2.30E-08 | A | G | 0.00151391 | 15.8488 |
| rs1981612 | 0.00922755 | 0.45601 | 1.00E-09 | A | C | 0.00151053 | 19.4765 |
| rs2038760 | -0.0115262 | 0.170908 | 6.60E-09 | T | C | 0.00198662 | 17.3583 |
| rs217181 | 0.0119554 | 0.193087 | 2.10E-10 | T | C | 0.00188145 | 20.5343 |
| rs2359239 | -0.00881461 | 0.391621 | 6.90E-09 | T | C | 0.00152153 | 17.0693 |
| rs2532111 | 0.0102852 | 0.639542 | 4.10E-11 | G | A | 0.00155794 | 22.4866 |
| rs2587505 | -0.00900424 | 0.419871 | 2.30E-09 | C | T | 0.00150694 | 18.2098 |
| rs2631360 | -0.0109177 | 0.519209 | 1.90E-13 | A | G | 0.00148434 | 27.4372 |
| rs2789514 | 0.0120546 | 0.867941 | 4.90E-08 | A | G | 0.00220932 | 15.3579 |
| rs2800789 | 0.00826025 | 0.480339 | 3.10E-08 | C | A | 0.00149261 | 15.7044 |
| rs28542042 | 0.0110101 | 0.308877 | 1.20E-11 | T | C | 0.00162277 | 23.8616 |
| rs2871865 | -0.0217979 | 0.116247 | 5.00E-21 | G | C | 0.00231659 | 45.0130 |
| rs2871960 | 0.0120879 | 0.444681 | 5.50E-16 | C | A | 0.00149222 | 33.2719 |
| rs2974438 | -0.0100735 | 0.211026 | 3.30E-08 | A | G | 0.00182387 | 15.5786 |
| rs3118903 | -0.0174477 | 0.219552 | 2.70E-22 | A | G | 0.00179692 | 48.1013 |
| rs34030812 | -0.0101679 | 0.367365 | 4.10E-11 | C | T | 0.00154042 | 22.1558 |
| rs34587452 | -0.0113642 | 0.21517 | 3.20E-10 | C | G | 0.00180741 | 20.1098 |
| rs34722008 | 0.00858569 | 0.353332 | 3.30E-08 | A | G | 0.00155452 | 15.5304 |
| rs34845616 | 0.0108395 | 0.245653 | 4.00E-10 | A | G | 0.00173373 | 20.0763 |
| rs35236379 | 0.0123565 | 0.142324 | 5.80E-09 | T | G | 0.00212204 | 17.1855 |
| rs35609019 | 0.00947689 | 0.397748 | 7.70E-10 | C | G | 0.00154061 | 19.8376 |
| rs3814877 | 0.0105385 | 0.401594 | 3.40E-12 | T | G | 0.00151385 | 24.6103 |
| rs3819121 | 0.0140963 | 0.368945 | 2.90E-20 | C | T | 0.00152872 | 42.6612 |
| rs4121165 | -0.0114169 | 0.211447 | 3.40E-10 | A | G | 0.00181868 | 20.0402 |
| rs41271299 | 0.0211891 | 0.051241 | 3.00E-10 | T | C | 0.00336402 | 20.1266 |
| rs4308051 | 0.0159929 | 0.789105 | 1.40E-18 | G | T | 0.00181863 | 39.2506 |
| rs4335354 | -0.00939409 | 0.315536 | 4.60E-09 | A | C | 0.00160262 | 17.5743 |
| rs4398863 | -0.00945015 | 0.7368 | 2.00E-08 | C | G | 0.00168454 | 15.9691 |
| rs4498020 | -0.0104238 | 0.723767 | 4.30E-10 | A | C | 0.00166995 | 20.0308 |
| rs4575361 | -0.0108028 | 0.31205 | 1.60E-11 | T | A | 0.00160223 | 23.1009 |
| rs4621706 | -0.0117068 | 0.544473 | 6.00E-15 | T | C | 0.00150003 | 31.3437 |
| rs4677601 | 0.00905884 | 0.510324 | 1.10E-09 | G | A | 0.001485 | 18.9091 |
| rs4737446 | 0.0104178 | 0.694626 | 1.20E-10 | T | G | 0.00161882 | 21.2280 |
| rs4739739 | -0.00853387 | 0.415057 | 1.40E-08 | G | A | 0.00150466 | 16.3036 |
| rs4811040 | -0.00924973 | 0.276636 | 3.00E-08 | G | C | 0.00166896 | 15.7867 |
| rs4930236 | 0.0119032 | 0.835892 | 3.80E-09 | A | C | 0.00202069 | 17.9216 |
| rs4962700 | 0.00904307 | 0.301977 | 3.20E-08 | G | C | 0.00163488 | 15.8944 |
| rs55681913 | 0.0137738 | 0.106095 | 1.60E-08 | C | T | 0.00243658 | 16.5906 |
| rs56338231 | -0.010847 | 0.258266 | 1.70E-10 | G | A | 0.00169739 | 20.7830 |
| rs58670122 | -0.0118148 | 0.143159 | 3.60E-08 | G | A | 0.00214462 | 15.7885 |
| rs59116179 | 0.00857385 | 0.617442 | 2.40E-08 | T | C | 0.00153635 | 16.0108 |
| rs6006984 | 0.00984917 | 0.277764 | 2.60E-09 | C | T | 0.00165413 | 17.9442 |
| rs61286123 | -0.0101041 | 0.227916 | 1.20E-08 | C | T | 0.00177111 | 16.5655 |
| rs61389091 | 0.0261627 | 0.04174 | 2.40E-12 | T | C | 0.00373295 | 25.2452 |
| rs61818100 | 0.0134422 | 0.116595 | 6.20E-09 | C | T | 0.00231335 | 17.1613 |
| rs62081464 | -0.0098827 | 0.227338 | 2.80E-08 | T | C | 0.00177979 | 15.8191 |
| rs635538 | -0.0216569 | 0.913853 | 3.80E-16 | A | G | 0.00265852 | 34.0482 |
| rs6433478 | 0.00905674 | 0.544228 | 1.50E-09 | C | T | 0.00149951 | 18.7605 |
| rs6680160 | 0.0100686 | 0.628066 | 6.00E-11 | G | A | 0.00153878 | 21.8365 |
| rs6689375 | -0.0160169 | 0.185489 | 5.30E-17 | T | A | 0.00191158 | 35.7404 |
| rs6802071 | -0.00940386 | 0.434928 | 3.80E-10 | T | C | 0.00150182 | 20.0404 |
| rs6882168 | -0.00935387 | 0.337417 | 2.80E-09 | T | C | 0.00157364 | 18.0368 |
| rs6962338 | -0.0214095 | 0.043993 | 3.20E-09 | G | A | 0.0036171 | 17.7758 |
| rs6977081 | 0.0147525 | 0.333835 | 1.40E-20 | T | G | 0.00158641 | 44.6314 |
| rs7026798 | 0.00823598 | 0.43232 | 4.60E-08 | C | T | 0.00150641 | 15.3500 |
| rs7124681 | -0.0116557 | 0.408355 | 1.00E-14 | A | C | 0.00150615 | 30.2662 |
| rs71298370 | 0.0148764 | 0.086241 | 3.80E-08 | A | G | 0.00270414 | 16.0809 |
| rs7148603 | 0.00957206 | 0.359427 | 1.60E-09 | A | G | 0.00158612 | 19.4519 |
| rs7176095 | -0.013375 | 0.128192 | 1.70E-09 | G | A | 0.00222025 | 18.4349 |
| rs7197751 | -0.00946321 | 0.363019 | 1.40E-09 | T | G | 0.00156268 | 19.0943 |
| rs723588 | 0.0127904 | 0.142598 | 1.60E-09 | C | T | 0.00211948 | 18.4432 |
| rs72977282 | -0.0155569 | 0.413856 | 7.80E-25 | A | T | 0.00151173 | 54.1383 |
| rs73307079 | 0.0111025 | 0.211119 | 1.40E-09 | C | T | 0.00183407 | 18.9300 |
| rs7516571 | 0.00937798 | 0.259017 | 3.10E-08 | G | A | 0.00169361 | 15.5641 |
| rs75497896 | -0.0206727 | 0.051006 | 9.10E-10 | C | T | 0.00337524 | 19.0744 |
| rs755547 | 0.0165156 | 0.188906 | 3.20E-18 | A | G | 0.00189766 | 38.5386 |
| rs7571789 | 0.0129539 | 0.522782 | 3.00E-18 | C | T | 0.001487 | 38.6037 |
| rs7575451 | -0.00973095 | 0.649752 | 3.80E-10 | G | C | 0.00155349 | 19.8704 |
| rs76895963 | 0.0359634 | 0.020694 | 3.90E-10 | G | T | 0.00574862 | 24.1691 |
| rs772014 | -0.0106178 | 0.392538 | 2.70E-12 | G | A | 0.00151891 | 24.7883 |
| rs7856625 | -0.0111297 | 0.609861 | 2.50E-13 | T | C | 0.0015209 | 27.1767 |
| rs7963801 | -0.0104348 | 0.571927 | 4.60E-12 | C | T | 0.00150824 | 24.5812 |
| rs7970350 | -0.0101345 | 0.494068 | 8.60E-12 | T | C | 0.00148418 | 23.6733 |
| rs8101782 | 0.00955082 | 0.702702 | 2.90E-08 | C | A | 0.00172167 | 17.5717 |
| rs8108461 | 0.00952393 | 0.572775 | 2.60E-10 | C | T | 0.0015066 | 20.4666 |
| rs821100 | -0.0101977 | 0.265512 | 1.50E-09 | G | A | 0.00168615 | 18.7001 |
| rs823130 | -0.0113317 | 0.432903 | 4.40E-14 | T | C | 0.00150098 | 29.0683 |
| rs9371201 | -0.00933847 | 0.335102 | 3.00E-09 | T | C | 0.0015738 | 17.9165 |
| rs9371881 | 0.00947946 | 0.359174 | 9.60E-10 | A | G | 0.00154996 | 19.0714 |
| rs9388769 | -0.0140821 | 0.67318 | 5.10E-19 | A | G | 0.00158065 | 40.2315 |
| rs9611273 | 0.0107888 | 0.25267 | 4.50E-10 | T | C | 0.00173048 | 20.2668 |
| rs9866627 | -0.0155989 | 0.084376 | 5.70E-09 | A | C | 0.00267761 | 17.3338 |
| rs9944324 | -0.00855272 | 0.456723 | 1.20E-08 | G | A | 0.00150169 | 16.7360 |
| rs999493 | 0.0128713 | 0.621921 | 6.20E-17 | A | G | 0.00153936 | 35.9211 |

SNP, single nucleotide polymorphism; Beta coefficients are in standard deviation (SD) unit per allele; EAF, effect allele frequency; SE, standard error;

**Supplementary Table 10.** Characteristics of selected SNPs concerning the causal effect of left-hand grip strength on stroke

| **SNP** | **Beta** | **EAF** | ***P*-value** | **Effect**  **allele** | **Other**  **allele** | **SE** | ***F*-value** |
| --- | --- | --- | --- | --- | --- | --- | --- |
| rs10097417 | -0.0132302 | 0.170904 | 1.90E-11 | G | A | 0.00197055 | 22.8699 |
| rs10144445 | -0.00936876 | 0.350118 | 1.90E-09 | G | C | 0.00155918 | 18.4155 |
| rs10176878 | -0.0129478 | 0.190861 | 8.60E-12 | C | T | 0.0018962 | 23.8731 |
| rs10403906 | -0.0100323 | 0.476375 | 1.50E-11 | A | G | 0.00148665 | 23.1497 |
| rs10821939 | -0.00935422 | 0.573342 | 4.90E-10 | A | G | 0.00150339 | 19.7370 |
| rs10831903 | 0.00928662 | 0.423383 | 8.10E-10 | T | C | 0.00151154 | 19.4137 |
| rs10846071 | -0.016641 | 0.393672 | 5.30E-28 | T | C | 0.00151685 | 60.9554 |
| rs10934857 | 0.00928675 | 0.258913 | 4.80E-08 | A | G | 0.00170182 | 15.2587 |
| rs10988217 | -0.00920657 | 0.603742 | 1.70E-09 | G | A | 0.00152911 | 18.6980 |
| rs11003014 | 0.011433 | 0.161184 | 1.60E-08 | G | A | 0.00202143 | 16.2959 |
| rs11076004 | -0.0115356 | 0.418177 | 2.10E-14 | A | G | 0.00150909 | 29.8547 |
| rs11111267 | 0.0108359 | 0.18106 | 1.90E-08 | G | A | 0.00192886 | 16.0536 |
| rs11121542 | -0.0157601 | 0.122677 | 3.00E-12 | A | G | 0.00225916 | 24.6500 |
| rs11125803 | 0.0142851 | 0.740516 | 3.80E-17 | T | C | 0.00169673 | 36.1576 |
| rs11168357 | -0.00962864 | 0.246008 | 2.50E-08 | A | G | 0.00172661 | 15.8568 |
| rs11204664 | -0.00865184 | 0.578725 | 8.10E-09 | C | T | 0.00150032 | 16.8277 |
| rs11243202 | 0.00972237 | 0.486019 | 7.00E-11 | C | T | 0.0014909 | 21.7730 |
| rs112485536 | 0.0161779 | 0.075507 | 9.40E-09 | T | C | 0.00281803 | 16.8463 |
| rs113315602 | -0.0181886 | 0.095848 | 8.60E-12 | C | A | 0.00266345 | 26.4363 |
| rs113434679 | -0.0149156 | 0.199577 | 1.90E-15 | A | C | 0.00187618 | 32.7715 |
| rs113918482 | -0.0101011 | 0.222507 | 1.50E-08 | G | A | 0.00178515 | 16.2760 |
| rs116409670 | -0.0152418 | 0.079819 | 2.60E-08 | T | C | 0.00273867 | 15.7333 |
| rs11669079 | 0.0109542 | 0.705026 | 1.90E-11 | T | A | 0.00163169 | 23.0105 |
| rs116922558 | -0.0215842 | 0.039916 | 2.10E-08 | G | A | 0.00385425 | 16.4626 |
| rs11769549 | 0.0204956 | 0.062372 | 4.50E-11 | A | T | 0.00311228 | 22.6525 |
| rs12316046 | -0.0174165 | 0.37795 | 5.00E-30 | G | A | 0.00152991 | 65.7653 |
| rs12473732 | 0.0109869 | 0.486507 | 1.40E-13 | T | C | 0.00148601 | 27.8070 |
| rs12528131 | -0.00882788 | 0.488343 | 2.80E-09 | G | A | 0.00148565 | 17.9551 |
| rs12533765 | -0.0092 | 0.280054 | 2.60E-08 | G | A | 0.00165199 | 15.7357 |
| rs12673062 | -0.0107691 | 0.215756 | 2.60E-09 | A | G | 0.00180808 | 18.0944 |
| rs12790261 | -0.0251882 | 0.082383 | 1.20E-20 | A | C | 0.00270305 | 44.2271 |
| rs12906830 | 0.0108401 | 0.601065 | 9.30E-13 | C | T | 0.00151797 | 25.9817 |
| rs12914702 | 0.0109514 | 0.300395 | 9.30E-11 | A | G | 0.00169071 | 23.2413 |
| rs13106087 | 0.0116202 | 0.829801 | 3.90E-09 | C | T | 0.00197399 | 17.5844 |
| rs13146142 | -0.0202031 | 0.158623 | 2.30E-23 | C | T | 0.00202888 | 50.2335 |
| rs13227429 | -0.00858767 | 0.561343 | 9.80E-09 | C | T | 0.0014977 | 16.7445 |
| rs13337177 | -0.0142799 | 0.180836 | 1.60E-13 | T | G | 0.00193621 | 27.8539 |
| rs13356200 | -0.00876765 | 0.393874 | 9.70E-09 | G | T | 0.0015286 | 16.9222 |
| rs143002906 | 0.0262281 | 0.027829 | 9.80E-09 | T | C | 0.00457421 | 17.1610 |
| rs1434095 | 0.0140301 | 0.875121 | 5.30E-10 | C | T | 0.00225906 | 19.8359 |
| rs1486925 | -0.0104734 | 0.314604 | 6.30E-11 | C | T | 0.00160211 | 21.8100 |
| rs150330307 | -0.0307753 | 0.031896 | 2.90E-13 | C | T | 0.0042167 | 26.9676 |
| rs1551042 | -0.0111279 | 0.647025 | 7.40E-13 | C | A | 0.00155167 | 26.0777 |
| rs1556659 | 0.0162896 | 0.38153 | 2.50E-26 | T | C | 0.00153452 | 57.7399 |
| rs1641457 | 0.0120456 | 0.223305 | 1.40E-11 | G | T | 0.0017829 | 23.2050 |
| rs16870531 | 0.011198 | 0.238492 | 1.30E-10 | T | C | 0.00174241 | 20.9992 |
| rs16910750 | 0.0112498 | 0.159639 | 3.60E-08 | C | G | 0.00204139 | 15.6553 |
| rs17282763 | 0.00893956 | 0.296281 | 4.40E-08 | C | T | 0.00163298 | 15.3640 |
| rs17466480 | -0.0118235 | 0.386968 | 9.80E-15 | G | A | 0.00152732 | 30.5797 |
| rs17630248 | -0.009184 | 0.347808 | 4.10E-09 | C | T | 0.00156162 | 17.6420 |
| rs181766 | 0.00963247 | 0.321793 | 1.90E-09 | C | T | 0.00160372 | 18.6718 |
| rs1884447 | 0.00846069 | 0.400566 | 2.30E-08 | A | G | 0.00151391 | 15.8488 |
| rs2038760 | -0.0115262 | 0.170908 | 6.60E-09 | T | C | 0.00198662 | 17.3583 |
| rs2359239 | -0.00881461 | 0.391621 | 6.90E-09 | T | C | 0.00152153 | 17.0693 |
| rs2532111 | 0.0102852 | 0.639542 | 4.10E-11 | G | A | 0.00155794 | 22.4866 |
| rs2587505 | -0.00900424 | 0.419871 | 2.30E-09 | C | T | 0.00150694 | 18.2098 |
| rs2631360 | -0.0109177 | 0.519209 | 1.90E-13 | A | G | 0.00148434 | 27.4372 |
| rs2789514 | 0.0120546 | 0.867941 | 4.90E-08 | A | G | 0.00220932 | 15.3579 |
| rs2800789 | 0.00826025 | 0.480339 | 3.10E-08 | C | A | 0.00149261 | 15.7044 |
| rs2850379 | -0.00828995 | 0.432305 | 3.30E-08 | A | C | 0.00150007 | 15.5517 |
| rs28542042 | 0.0110101 | 0.308877 | 1.20E-11 | T | C | 0.00162277 | 23.8616 |
| rs2871865 | -0.0217979 | 0.116247 | 5.00E-21 | G | C | 0.00231659 | 45.0130 |
| rs2871960 | 0.0120879 | 0.444681 | 5.50E-16 | C | A | 0.00149222 | 33.2719 |
| rs2974438 | -0.0100735 | 0.211026 | 3.30E-08 | A | G | 0.00182387 | 15.5786 |
| rs3118903 | -0.0174477 | 0.219552 | 2.70E-22 | A | G | 0.00179692 | 48.1013 |
| rs34030812 | -0.0101679 | 0.367365 | 4.10E-11 | C | T | 0.00154042 | 22.1558 |
| rs34587452 | -0.0113642 | 0.21517 | 3.20E-10 | C | G | 0.00180741 | 20.1098 |
| rs34722008 | 0.00858569 | 0.353332 | 3.30E-08 | A | G | 0.00155452 | 15.5304 |
| rs34845616 | 0.0108395 | 0.245653 | 4.00E-10 | A | G | 0.00173373 | 20.0763 |
| rs35175534 | -0.0163733 | 0.139986 | 3.60E-12 | C | A | 0.00235477 | 29.7608 |
| rs35236379 | 0.0123565 | 0.142324 | 5.80E-09 | T | G | 0.00212204 | 17.1855 |
| rs35609019 | 0.00947689 | 0.397748 | 7.70E-10 | C | G | 0.00154061 | 19.8376 |
| rs3819121 | 0.0140963 | 0.368945 | 2.90E-20 | C | T | 0.00152872 | 42.6612 |
| rs4121165 | -0.0114169 | 0.211447 | 3.40E-10 | A | G | 0.00181868 | 20.0402 |
| rs41271299 | 0.0211891 | 0.051241 | 3.00E-10 | T | C | 0.00336402 | 20.1266 |
| rs4308051 | 0.0159929 | 0.789105 | 1.40E-18 | G | T | 0.00181863 | 39.2506 |
| rs4398863 | -0.00945015 | 0.7368 | 2.00E-08 | C | G | 0.00168454 | 15.9691 |
| rs4498020 | -0.0104238 | 0.723767 | 4.30E-10 | A | C | 0.00166995 | 20.0308 |
| rs4621706 | -0.0117068 | 0.544473 | 6.00E-15 | T | C | 0.00150003 | 31.3437 |
| rs4677601 | 0.00905884 | 0.510324 | 1.10E-09 | G | A | 0.001485 | 18.9091 |
| rs4713506 | -0.0157268 | 0.255607 | 2.00E-20 | A | G | 0.00169811 | 43.3960 |
| rs4737446 | 0.0104178 | 0.694626 | 1.20E-10 | T | G | 0.00161882 | 21.2280 |
| rs4739739 | -0.00853387 | 0.415057 | 1.40E-08 | G | A | 0.00150466 | 16.3036 |
| rs4811040 | -0.00924973 | 0.276636 | 3.00E-08 | G | C | 0.00166896 | 15.7867 |
| rs4930236 | 0.0119032 | 0.835892 | 3.80E-09 | A | C | 0.00202069 | 17.9216 |
| rs4962700 | 0.00904307 | 0.301977 | 3.20E-08 | G | C | 0.00163488 | 15.8944 |
| rs55681913 | 0.0137738 | 0.106095 | 1.60E-08 | C | T | 0.00243658 | 16.5906 |
| rs56338231 | -0.010847 | 0.258266 | 1.70E-10 | G | A | 0.00169739 | 20.7830 |
| rs58670122 | -0.0118148 | 0.143159 | 3.60E-08 | G | A | 0.00214462 | 15.7885 |
| rs59116179 | 0.00857385 | 0.617442 | 2.40E-08 | T | C | 0.00153635 | 16.0108 |
| rs6006984 | 0.00984917 | 0.277764 | 2.60E-09 | C | T | 0.00165413 | 17.9442 |
| rs61286123 | -0.0101041 | 0.227916 | 1.20E-08 | C | T | 0.00177111 | 16.5655 |
| rs61389091 | 0.0261627 | 0.04174 | 2.40E-12 | T | C | 0.00373295 | 25.2452 |
| rs61818100 | 0.0134422 | 0.116595 | 6.20E-09 | C | T | 0.00231335 | 17.1613 |
| rs62081464 | -0.0098827 | 0.227338 | 2.80E-08 | T | C | 0.00177979 | 15.8191 |
| rs62253653 | 0.0106985 | 0.295379 | 5.50E-11 | G | A | 0.00163146 | 21.9662 |
| rs635538 | -0.0216569 | 0.913853 | 3.80E-16 | A | G | 0.00265852 | 34.0482 |
| rs6433478 | 0.00905674 | 0.544228 | 1.50E-09 | C | T | 0.00149951 | 18.7605 |
| rs6680160 | 0.0100686 | 0.628066 | 6.00E-11 | G | A | 0.00153878 | 21.8365 |
| rs6689375 | -0.0160169 | 0.185489 | 5.30E-17 | T | A | 0.00191158 | 35.7404 |
| rs6802071 | -0.00940386 | 0.434928 | 3.80E-10 | T | C | 0.00150182 | 20.0404 |
| rs6882168 | -0.00935387 | 0.337417 | 2.80E-09 | T | C | 0.00157364 | 18.0368 |
| rs6962338 | -0.0214095 | 0.043993 | 3.20E-09 | G | A | 0.0036171 | 17.7758 |
| rs6977081 | 0.0147525 | 0.333835 | 1.40E-20 | T | G | 0.00158641 | 44.6314 |
| rs7026798 | 0.00823598 | 0.43232 | 4.60E-08 | C | T | 0.00150641 | 15.3500 |
| rs71298370 | 0.0148764 | 0.086241 | 3.80E-08 | A | G | 0.00270414 | 16.0809 |
| rs7148603 | 0.00957206 | 0.359427 | 1.60E-09 | A | G | 0.00158612 | 19.4519 |
| rs7176095 | -0.013375 | 0.128192 | 1.70E-09 | G | A | 0.00222025 | 18.4349 |
| rs7197751 | -0.00946321 | 0.363019 | 1.40E-09 | T | G | 0.00156268 | 19.0943 |
| rs723588 | 0.0127904 | 0.142598 | 1.60E-09 | C | T | 0.00211948 | 18.4432 |
| rs72977282 | -0.0155569 | 0.413856 | 7.80E-25 | A | T | 0.00151173 | 54.1383 |
| rs73307079 | 0.0111025 | 0.211119 | 1.40E-09 | C | T | 0.00183407 | 18.9300 |
| rs7516571 | 0.00937798 | 0.259017 | 3.10E-08 | G | A | 0.00169361 | 15.5641 |
| rs75497896 | -0.0206727 | 0.051006 | 9.10E-10 | C | T | 0.00337524 | 19.0744 |
| rs755547 | 0.0165156 | 0.188906 | 3.20E-18 | A | G | 0.00189766 | 38.5386 |
| rs7571789 | 0.0129539 | 0.522782 | 3.00E-18 | C | T | 0.001487 | 38.6037 |
| rs7575451 | -0.00973095 | 0.649752 | 3.80E-10 | G | C | 0.00155349 | 19.8704 |
| rs772014 | -0.0106178 | 0.392538 | 2.70E-12 | G | A | 0.00151891 | 24.7883 |
| rs7856625 | -0.0111297 | 0.609861 | 2.50E-13 | T | C | 0.0015209 | 27.1767 |
| rs7963801 | -0.0104348 | 0.571927 | 4.60E-12 | C | T | 0.00150824 | 24.5812 |
| rs7970350 | -0.0101345 | 0.494068 | 8.60E-12 | T | C | 0.00148418 | 23.6733 |
| rs8101782 | 0.00955082 | 0.702702 | 2.90E-08 | C | A | 0.00172167 | 17.5717 |
| rs8108461 | 0.00952393 | 0.572775 | 2.60E-10 | C | T | 0.0015066 | 20.4666 |
| rs821100 | -0.0101977 | 0.265512 | 1.50E-09 | G | A | 0.00168615 | 18.7001 |
| rs823130 | -0.0113317 | 0.432903 | 4.40E-14 | T | C | 0.00150098 | 29.0683 |
| rs9371201 | -0.00933847 | 0.335102 | 3.00E-09 | T | C | 0.0015738 | 17.9165 |
| rs9371881 | 0.00947946 | 0.359174 | 9.60E-10 | A | G | 0.00154996 | 19.0714 |
| rs9388769 | -0.0140821 | 0.67318 | 5.10E-19 | A | G | 0.00158065 | 40.2315 |
| rs9611273 | 0.0107888 | 0.25267 | 4.50E-10 | T | C | 0.00173048 | 20.2668 |
| rs9866627 | -0.0155989 | 0.084376 | 5.70E-09 | A | C | 0.00267761 | 17.3338 |
| rs9944324 | -0.00855272 | 0.456723 | 1.20E-08 | G | A | 0.00150169 | 16.7360 |
| rs999493 | 0.0128713 | 0.621921 | 6.20E-17 | A | G | 0.00153936 | 35.9211 |

SNP, single nucleotide polymorphism; Beta coefficients are in standard deviation (SD) unit per allele; EAF, effect allele frequency; SE, standard error;

**Supplementary Table 11.** Characteristics of selected SNPs concerning the causal effect of left-hand grip strength on MI

| **SNP** | **Beta** | **EAF** | ***P*-value** | **Effect**  **allele** | | **Other**  **allele** | **SE** | ***F*-value** |
| --- | --- | --- | --- | --- | --- | --- | --- | --- |
| rs10097417 | -0.0132302 | 0.170904 | 1.90E-11 | | G | A | 0.00197055 | 22.8699 |
| rs10144445 | -0.00936876 | 0.350118 | 1.90E-09 | | G | C | 0.00155918 | 18.4155 |
| rs10176878 | -0.0129478 | 0.190861 | 8.60E-12 | | C | T | 0.0018962 | 23.8731 |
| rs10403906 | -0.0100323 | 0.476375 | 1.50E-11 | | A | G | 0.00148665 | 23.1497 |
| rs1044299 | 0.01401 | 0.546046 | 6.60E-21 | | T | C | 0.00149362 | 44.8656 |
| rs10786706 | 0.0100072 | 0.465745 | 1.70E-11 | | T | C | 0.00148741 | 22.9772 |
| rs10788958 | 0.0141976 | 0.644753 | 1.00E-19 | | G | C | 0.00156276 | 42.5743 |
| rs10821939 | -0.00935422 | 0.573342 | 4.90E-10 | | A | G | 0.00150339 | 19.7370 |
| rs10831903 | 0.00928662 | 0.423383 | 8.10E-10 | | T | C | 0.00151154 | 19.4137 |
| rs10934857 | 0.00928675 | 0.258913 | 4.80E-08 | | A | G | 0.00170182 | 15.2587 |
| rs10988217 | -0.00920657 | 0.603742 | 1.70E-09 | | G | A | 0.00152911 | 18.6980 |
| rs11002322 | -0.00998924 | 0.340459 | 2.10E-10 | | T | G | 0.00157255 | 20.6607 |
| rs11003014 | 0.011433 | 0.161184 | 1.60E-08 | | G | A | 0.00202143 | 16.2959 |
| rs11076004 | -0.0115356 | 0.418177 | 2.10E-14 | | A | G | 0.00150909 | 29.8547 |
| rs11111267 | 0.0108359 | 0.18106 | 1.90E-08 | | G | A | 0.00192886 | 16.0536 |
| rs11121542 | -0.0157601 | 0.122677 | 3.00E-12 | | A | G | 0.00225916 | 24.6500 |
| rs11125803 | 0.0142851 | 0.740516 | 3.80E-17 | | T | C | 0.00169673 | 36.1576 |
| rs11168357 | -0.00962864 | 0.246008 | 2.50E-08 | | A | G | 0.00172661 | 15.8568 |
| rs11204664 | -0.00865184 | 0.578725 | 8.10E-09 | | C | T | 0.00150032 | 16.8277 |
| rs11243202 | 0.00972237 | 0.486019 | 7.00E-11 | | C | T | 0.0014909 | 21.7730 |
| rs112485536 | 0.0161779 | 0.075507 | 9.40E-09 | | T | C | 0.00281803 | 16.8463 |
| rs113315602 | -0.0181886 | 0.095848 | 8.60E-12 | | C | A | 0.00266345 | 26.4363 |
| rs113434679 | -0.0149156 | 0.199577 | 1.90E-15 | | A | C | 0.00187618 | 32.7715 |
| rs113918482 | -0.0101011 | 0.222507 | 1.50E-08 | | G | A | 0.00178515 | 16.2760 |
| rs116409670 | -0.0152418 | 0.079819 | 2.60E-08 | | T | C | 0.00273867 | 15.7333 |
| rs11669079 | 0.0109542 | 0.705026 | 1.90E-11 | | T | A | 0.00163169 | 23.0105 |
| rs11769549 | 0.0204956 | 0.062372 | 4.50E-11 | | A | T | 0.00311228 | 22.6525 |
| rs12316046 | -0.0174165 | 0.37795 | 5.00E-30 | | G | A | 0.00152991 | 65.7653 |
| rs12473732 | 0.0109869 | 0.486507 | 1.40E-13 | | T | C | 0.00148601 | 27.8070 |
| rs12528131 | -0.00882788 | 0.488343 | 2.80E-09 | | G | A | 0.00148565 | 17.9551 |
| rs12533765 | -0.0092 | 0.280054 | 2.60E-08 | | G | A | 0.00165199 | 15.7357 |
| rs12673062 | -0.0107691 | 0.215756 | 2.60E-09 | | A | G | 0.00180808 | 18.0944 |
| rs12790261 | -0.0251882 | 0.082383 | 1.20E-20 | | A | C | 0.00270305 | 44.2271 |
| rs12906830 | 0.0108401 | 0.601065 | 9.30E-13 | | C | T | 0.00151797 | 25.9817 |
| rs13106087 | 0.0116202 | 0.829801 | 3.90E-09 | | C | T | 0.00197399 | 17.5844 |
| rs13146142 | -0.0202031 | 0.158623 | 2.30E-23 | | C | T | 0.00202888 | 50.2335 |
| rs13227429 | -0.00858767 | 0.561343 | 9.80E-09 | | C | T | 0.0014977 | 16.7445 |
| rs13337177 | -0.0142799 | 0.180836 | 1.60E-13 | | T | G | 0.00193621 | 27.8539 |
| rs13356200 | -0.00876765 | 0.393874 | 9.70E-09 | | G | T | 0.0015286 | 16.9222 |
| rs143002906 | 0.0262281 | 0.027829 | 9.80E-09 | | T | C | 0.00457421 | 17.1610 |
| rs143384 | 0.0209202 | 0.404388 | 1.50E-43 | | G | A | 0.00151159 | 97.2162 |
| rs1434095 | 0.0140301 | 0.875121 | 5.30E-10 | | C | T | 0.00225906 | 19.8359 |
| rs1486925 | -0.0104734 | 0.314604 | 6.30E-11 | | C | T | 0.00160211 | 21.8100 |
| rs150330307 | -0.0307753 | 0.031896 | 2.90E-13 | | C | T | 0.0042167 | 26.9676 |
| rs1551042 | -0.0111279 | 0.647025 | 7.40E-13 | | C | A | 0.00155167 | 26.0777 |
| rs1556659 | 0.0162896 | 0.38153 | 2.50E-26 | | T | C | 0.00153452 | 57.7399 |
| rs1641457 | 0.0120456 | 0.223305 | 1.40E-11 | | G | T | 0.0017829 | 23.2050 |
| rs16870531 | 0.011198 | 0.238492 | 1.30E-10 | | T | C | 0.00174241 | 20.9992 |
| rs16910750 | 0.0112498 | 0.159639 | 3.60E-08 | | C | G | 0.00204139 | 15.6553 |
| rs17282763 | 0.00893956 | 0.296281 | 4.40E-08 | | C | T | 0.00163298 | 15.3640 |
| rs17466480 | -0.0118235 | 0.386968 | 9.80E-15 | | G | A | 0.00152732 | 30.5797 |
| rs17630248 | -0.009184 | 0.347808 | 4.10E-09 | | C | T | 0.00156162 | 17.6420 |
| rs181766 | 0.00963247 | 0.321793 | 1.90E-09 | | C | T | 0.00160372 | 18.6718 |
| rs1884447 | 0.00846069 | 0.400566 | 2.30E-08 | | A | G | 0.00151391 | 15.8488 |
| rs2038760 | -0.0115262 | 0.170908 | 6.60E-09 | | T | C | 0.00198662 | 17.3583 |
| rs217181 | 0.0119554 | 0.193087 | 2.10E-10 | | T | C | 0.00188145 | 20.5343 |
| rs2359239 | -0.00881461 | 0.391621 | 6.90E-09 | | T | C | 0.00152153 | 17.0693 |
| rs2532111 | 0.0102852 | 0.639542 | 4.10E-11 | | G | A | 0.00155794 | 22.4866 |
| rs2587505 | -0.00900424 | 0.419871 | 2.30E-09 | | C | T | 0.00150694 | 18.2098 |
| rs2631360 | -0.0109177 | 0.519209 | 1.90E-13 | | A | G | 0.00148434 | 27.4372 |
| rs2789514 | 0.0120546 | 0.867941 | 4.90E-08 | | A | G | 0.00220932 | 15.3579 |
| rs2800789 | 0.00826025 | 0.480339 | 3.10E-08 | | C | A | 0.00149261 | 15.7044 |
| rs28542042 | 0.0110101 | 0.308877 | 1.20E-11 | | T | C | 0.00162277 | 23.8616 |
| rs2871865 | -0.0217979 | 0.116247 | 5.00E-21 | | G | C | 0.00231659 | 45.0130 |
| rs2871960 | 0.0120879 | 0.444681 | 5.50E-16 | | C | A | 0.00149222 | 33.2719 |
| rs2974438 | -0.0100735 | 0.211026 | 3.30E-08 | | A | G | 0.00182387 | 15.5786 |
| rs3118903 | -0.0174477 | 0.219552 | 2.70E-22 | | A | G | 0.00179692 | 48.1013 |
| rs34030812 | -0.0101679 | 0.367365 | 4.10E-11 | | C | T | 0.00154042 | 22.1558 |
| rs34587452 | -0.0113642 | 0.21517 | 3.20E-10 | | C | G | 0.00180741 | 20.1098 |
| rs34722008 | 0.00858569 | 0.353332 | 3.30E-08 | | A | G | 0.00155452 | 15.5304 |
| rs34845616 | 0.0108395 | 0.245653 | 4.00E-10 | | A | G | 0.00173373 | 20.0763 |
| rs35236379 | 0.0123565 | 0.142324 | 5.80E-09 | | T | G | 0.00212204 | 17.1855 |
| rs35609019 | 0.00947689 | 0.397748 | 7.70E-10 | | C | G | 0.00154061 | 19.8376 |
| rs3814877 | 0.0105385 | 0.401594 | 3.40E-12 | | T | G | 0.00151385 | 24.6103 |
| rs3819121 | 0.0140963 | 0.368945 | 2.90E-20 | | C | T | 0.00152872 | 42.6612 |
| rs4121165 | -0.0114169 | 0.211447 | 3.40E-10 | | A | G | 0.00181868 | 20.0402 |
| rs41271299 | 0.0211891 | 0.051241 | 3.00E-10 | | T | C | 0.00336402 | 20.1266 |
| rs4308051 | 0.0159929 | 0.789105 | 1.40E-18 | | G | T | 0.00181863 | 39.2506 |
| rs4335354 | -0.00939409 | 0.315536 | 4.60E-09 | | A | C | 0.00160262 | 17.5743 |
| rs4398863 | -0.00945015 | 0.7368 | 2.00E-08 | | C | G | 0.00168454 | 15.9691 |
| rs4498020 | -0.0104238 | 0.723767 | 4.30E-10 | | A | C | 0.00166995 | 20.0308 |
| rs4621706 | -0.0117068 | 0.544473 | 6.00E-15 | | T | C | 0.00150003 | 31.3437 |
| rs4677601 | 0.00905884 | 0.510324 | 1.10E-09 | | G | A | 0.001485 | 18.9091 |
| rs4737446 | 0.0104178 | 0.694626 | 1.20E-10 | | T | G | 0.00161882 | 21.2280 |
| rs4739739 | -0.00853387 | 0.415057 | 1.40E-08 | | G | A | 0.00150466 | 16.3036 |
| rs4811040 | -0.00924973 | 0.276636 | 3.00E-08 | | G | C | 0.00166896 | 15.7867 |
| rs4930236 | 0.0119032 | 0.835892 | 3.80E-09 | | A | C | 0.00202069 | 17.9216 |
| rs4962700 | 0.00904307 | 0.301977 | 3.20E-08 | | G | C | 0.00163488 | 15.8944 |
| rs55681913 | 0.0137738 | 0.106095 | 1.60E-08 | | C | T | 0.00243658 | 16.5906 |
| rs56338231 | -0.010847 | 0.258266 | 1.70E-10 | | G | A | 0.00169739 | 20.7830 |
| rs58670122 | -0.0118148 | 0.143159 | 3.60E-08 | | G | A | 0.00214462 | 15.7885 |
| rs59116179 | 0.00857385 | 0.617442 | 2.40E-08 | | T | C | 0.00153635 | 16.0108 |
| rs6006984 | 0.00984917 | 0.277764 | 2.60E-09 | | C | T | 0.00165413 | 17.9442 |
| rs61286123 | -0.0101041 | 0.227916 | 1.20E-08 | | C | T | 0.00177111 | 16.5655 |
| rs61389091 | 0.0261627 | 0.04174 | 2.40E-12 | | T | C | 0.00373295 | 25.2452 |
| rs61818100 | 0.0134422 | 0.116595 | 6.20E-09 | | C | T | 0.00231335 | 17.1613 |
| rs62081464 | -0.0098827 | 0.227338 | 2.80E-08 | | T | C | 0.00177979 | 15.8191 |
| rs62253653 | 0.0106985 | 0.295379 | 5.50E-11 | | G | A | 0.00163146 | 21.9662 |
| rs635538 | -0.0216569 | 0.913853 | 3.80E-16 | | A | G | 0.00265852 | 34.0482 |
| rs6433478 | 0.00905674 | 0.544228 | 1.50E-09 | | C | T | 0.00149951 | 18.7605 |
| rs6680160 | 0.0100686 | 0.628066 | 6.00E-11 | | G | A | 0.00153878 | 21.8365 |
| rs6689375 | -0.0160169 | 0.185489 | 5.30E-17 | | T | A | 0.00191158 | 35.7404 |
| rs6802071 | -0.00940386 | 0.434928 | 3.80E-10 | | T | C | 0.00150182 | 20.0404 |
| rs6882168 | -0.00935387 | 0.337417 | 2.80E-09 | | T | C | 0.00157364 | 18.0368 |
| rs6962338 | -0.0214095 | 0.043993 | 3.20E-09 | | G | A | 0.0036171 | 17.7758 |
| rs6977081 | 0.0147525 | 0.333835 | 1.40E-20 | | T | G | 0.00158641 | 44.6314 |
| rs7026798 | 0.00823598 | 0.43232 | 4.60E-08 | | C | T | 0.00150641 | 15.3500 |
| rs7124681 | -0.0116557 | 0.408355 | 1.00E-14 | | A | C | 0.00150615 | 30.2662 |
| rs71298370 | 0.0148764 | 0.086241 | 3.80E-08 | | A | G | 0.00270414 | 16.0809 |
| rs7148603 | 0.00957206 | 0.359427 | 1.60E-09 | | A | G | 0.00158612 | 19.4519 |
| rs7176095 | -0.013375 | 0.128192 | 1.70E-09 | | G | A | 0.00222025 | 18.4349 |
| rs7196917 | -0.0117302 | 0.429948 | 5.40E-15 | | G | A | 0.00150067 | 31.0974 |
| rs7197751 | -0.00946321 | 0.363019 | 1.40E-09 | | T | G | 0.00156268 | 19.0943 |
| rs723588 | 0.0127904 | 0.142598 | 1.60E-09 | | C | T | 0.00211948 | 18.4432 |
| rs72977282 | -0.0155569 | 0.413856 | 7.80E-25 | | A | T | 0.00151173 | 54.1383 |
| rs73307079 | 0.0111025 | 0.211119 | 1.40E-09 | | C | T | 0.00183407 | 18.9300 |
| rs7516571 | 0.00937798 | 0.259017 | 3.10E-08 | | G | A | 0.00169361 | 15.5641 |
| rs75497896 | -0.0206727 | 0.051006 | 9.10E-10 | | C | T | 0.00337524 | 19.0744 |
| rs755547 | 0.0165156 | 0.188906 | 3.20E-18 | | A | G | 0.00189766 | 38.5386 |
| rs7571789 | 0.0129539 | 0.522782 | 3.00E-18 | | C | T | 0.001487 | 38.6037 |
| rs7575451 | -0.00973095 | 0.649752 | 3.80E-10 | | G | C | 0.00155349 | 19.8704 |
| rs76895963 | 0.0359634 | 0.020694 | 3.90E-10 | | G | T | 0.00574862 | 24.1691 |
| rs772014 | -0.0106178 | 0.392538 | 2.70E-12 | | G | A | 0.00151891 | 24.7883 |
| rs77485342 | 0.0329675 | 0.018003 | 3.50E-09 | | T | C | 0.00558187 | 17.7173 |
| rs7856625 | -0.0111297 | 0.609861 | 2.50E-13 | | T | C | 0.0015209 | 27.1767 |
| rs7963801 | -0.0104348 | 0.571927 | 4.60E-12 | | C | T | 0.00150824 | 24.5812 |
| rs7970350 | -0.0101345 | 0.494068 | 8.60E-12 | | T | C | 0.00148418 | 23.6733 |
| rs8101782 | 0.00955082 | 0.702702 | 2.90E-08 | | C | A | 0.00172167 | 17.5717 |
| rs8108461 | 0.00952393 | 0.572775 | 2.60E-10 | | C | T | 0.0015066 | 20.4666 |
| rs821100 | -0.0101977 | 0.265512 | 1.50E-09 | | G | A | 0.00168615 | 18.7001 |
| rs823130 | -0.0113317 | 0.432903 | 4.40E-14 | | T | C | 0.00150098 | 29.0683 |
| rs9371201 | -0.00933847 | 0.335102 | 3.00E-09 | | T | C | 0.0015738 | 17.9165 |
| rs9371881 | 0.00947946 | 0.359174 | 9.60E-10 | | A | G | 0.00154996 | 19.0714 |
| rs9388769 | -0.0140821 | 0.67318 | 5.10E-19 | | A | G | 0.00158065 | 40.2315 |
| rs9611273 | 0.0107888 | 0.25267 | 4.50E-10 | | T | C | 0.00173048 | 20.2668 |
| rs9866627 | -0.0155989 | 0.084376 | 5.70E-09 | | A | C | 0.00267761 | 17.3338 |
| rs9944324 | -0.00855272 | 0.456723 | 1.20E-08 | | G | A | 0.00150169 | 16.7360 |
| rs997850 | -0.00887326 | 0.60474 | 6.30E-09 | | C | G | 0.0015279 | 17.3535 |
| rs999493 | 0.0128713 | 0.621921 | 6.20E-17 | | A | G | 0.00153936 | 35.9211 |

SNP, single nucleotide polymorphism; Beta coefficients are in standard deviation (SD) unit per allele; EAF, effect allele frequency; SE, standard error;

**Supplementary Table 12.** Characteristics of selected SNPs concerning the causal effect of right-hand grip strength on CHD

| **SNP** | **Beta** | **EAF** | ***P*-value** | **Effect**  **allele** | **Other**  **allele** | **SE** | ***F*-value** |
| --- | --- | --- | --- | --- | --- | --- | --- |
| rs10193039 | -0.0103284 | 0.28057 | 4.10E-10 | T | A | 0.00165217 | 19.85763166 |
| rs10278546 | 0.0107539 | 0.194705 | 1.10E-08 | C | A | 0.00188373 | 16.72217322 |
| rs1043515 | 0.0138281 | 0.566183 | 2.80E-20 | G | A | 0.00149848 | 43.31537564 |
| rs1047891 | 0.0096867 | 0.315772 | 1.30E-09 | A | C | 0.0015965 | 18.69634196 |
| rs10483727 | -0.00901803 | 0.610138 | 3.30E-09 | C | T | 0.00152456 | 17.83988327 |
| rs10520770 | 0.011742 | 0.44929 | 4.50E-15 | C | T | 0.00149773 | 31.4612789 |
| rs10761411 | -0.0114975 | 0.812419 | 1.30E-08 | T | C | 0.00202082 | 18.57830664 |
| rs10770125 | 0.00844132 | 0.477072 | 1.40E-08 | G | A | 0.00148775 | 16.39362012 |
| rs10784502 | -0.0111247 | 0.512218 | 7.10E-14 | T | C | 0.00148608 | 28.51654818 |
| rs10798483 | 0.0145083 | 0.54729 | 2.80E-22 | A | G | 0.00149469 | 48.09820064 |
| rs10799428 | -0.0143588 | 0.18721 | 4.60E-14 | T | C | 0.00190367 | 28.93235675 |
| rs11022513 | -0.00921054 | 0.569312 | 1.10E-09 | T | C | 0.00151016 | 19.18289885 |
| rs11039348 | -0.00976855 | 0.348363 | 3.90E-10 | A | G | 0.00156104 | 19.97697169 |
| rs112330055 | 0.0179824 | 0.062972 | 1.70E-08 | A | G | 0.00319007 | 17.59643988 |
| rs11243202 | 0.0116378 | 0.486018 | 6.50E-15 | C | T | 0.00149304 | 31.20213448 |
| rs1125 | -0.0100304 | 0.336554 | 1.90E-10 | A | G | 0.00157472 | 20.71711267 |
| rs113315602 | -0.0212911 | 0.095826 | 1.40E-15 | C | A | 0.00266761 | 36.22251191 |
| rs113835839 | -0.00986353 | 0.247871 | 1.10E-08 | T | C | 0.00172486 | 16.72674441 |
| rs113851275 | 0.0131425 | 0.107596 | 4.30E-08 | A | G | 0.00239826 | 15.29469726 |
| rs114924396 | -0.0191251 | 0.05363 | 8.20E-09 | G | A | 0.00331785 | 17.12005633 |
| rs11998884 | 0.0173433 | 0.061825 | 2.80E-08 | T | C | 0.0031243 | 16.08938639 |
| rs12052508 | -0.0133517 | 0.879044 | 4.70E-09 | T | C | 0.00227992 | 17.47996259 |
| rs12101479 | -0.0106133 | 0.237244 | 1.50E-09 | C | G | 0.0017554 | 18.79802963 |
| rs12316046 | -0.0162046 | 0.377959 | 3.80E-26 | G | A | 0.00153212 | 56.93860632 |
| rs12412806 | -0.00909582 | 0.294214 | 3.00E-08 | A | G | 0.00164139 | 15.84338167 |
| rs12452505 | -0.0144313 | 0.142315 | 1.40E-11 | G | C | 0.00213545 | 23.44360288 |
| rs12522139 | -0.0114381 | 0.171144 | 6.80E-09 | G | T | 0.00197332 | 17.11502492 |
| rs12562146 | 0.0118706 | 0.145156 | 2.00E-08 | A | T | 0.00211554 | 16.12483438 |
| rs12616285 | 0.0122195 | 0.148607 | 5.40E-09 | G | T | 0.00209396 | 17.42230036 |
| rs12763284 | 0.00959741 | 0.466048 | 1.20E-10 | G | A | 0.00148928 | 21.13847953 |
| rs12790261 | -0.0263714 | 0.082377 | 2.00E-22 | A | C | 0.00270734 | 48.48363489 |
| rs12823922 | -0.0114203 | 0.222184 | 1.60E-10 | G | A | 0.00178649 | 20.78629709 |
| rs12899474 | -0.0148939 | 0.108065 | 5.40E-10 | T | C | 0.00239936 | 19.71816864 |
| rs13106087 | 0.0129259 | 0.829827 | 6.20E-11 | C | T | 0.001977 | 21.75871117 |
| rs13146142 | -0.0207968 | 0.158627 | 1.40E-24 | C | T | 0.00203165 | 53.23799948 |
| rs13169333 | 0.00929083 | 0.260299 | 4.30E-08 | C | T | 0.00169626 | 15.32727828 |
| rs13355365 | -0.00846604 | 0.379543 | 3.40E-08 | T | C | 0.00153398 | 15.5654199 |
| rs13356200 | -0.00920983 | 0.393811 | 1.80E-09 | G | T | 0.00153092 | 18.67366586 |
| rs143384 | 0.0230423 | 0.404392 | 2.50E-52 | G | A | 0.00151366 | 117.9610683 |
| rs1442883 | -0.0106189 | 0.253067 | 5.80E-10 | A | C | 0.00171426 | 19.65656439 |
| rs1486925 | -0.0095288 | 0.314649 | 2.90E-09 | C | T | 0.00160467 | 18.05700469 |
| rs150330307 | -0.0325831 | 0.031875 | 1.20E-14 | C | T | 0.00422401 | 30.2139678 |
| rs1550115 | 0.0152836 | 0.748561 | 4.50E-19 | T | C | 0.00171276 | 40.54732669 |
| rs1556659 | 0.0175345 | 0.381531 | 3.80E-30 | T | C | 0.00153689 | 66.91298949 |
| rs1641457 | 0.0130726 | 0.223327 | 2.40E-13 | G | T | 0.00178536 | 27.33644313 |
| rs1840753 | 0.0176942 | 0.062797 | 1.40E-08 | T | C | 0.00312088 | 16.99273595 |
| rs1885690 | -0.00839964 | 0.410365 | 2.80E-08 | A | C | 0.00151212 | 15.74354811 |
| rs1892425 | 0.0113357 | 0.238631 | 1.10E-10 | A | G | 0.00175726 | 21.53038972 |
| rs2147461 | 0.0134299 | 0.117274 | 6.10E-09 | C | T | 0.00231042 | 17.21877718 |
| rs2165241 | 0.0122486 | 0.508842 | 1.90E-16 | C | T | 0.00148844 | 34.57980344 |
| rs2194411 | 0.0142831 | 0.128444 | 2.10E-10 | A | G | 0.00224795 | 21.06137802 |
| rs2194747 | 0.00985126 | 0.707014 | 1.90E-09 | G | A | 0.00163925 | 18.53910567 |
| rs2208562 | -0.0115616 | 0.609627 | 3.10E-14 | T | C | 0.00152246 | 29.33731409 |
| rs2226685 | 0.0103333 | 0.758696 | 3.10E-09 | C | T | 0.00174324 | 18.02769679 |
| rs2244621 | 0.0115957 | 0.144157 | 4.50E-08 | T | C | 0.00211936 | 15.29857794 |
| rs2273555 | 0.0110316 | 0.606106 | 4.20E-13 | A | G | 0.00152155 | 26.79434044 |
| rs2296316 | -0.00821333 | 0.464404 | 4.60E-08 | C | T | 0.00150213 | 15.4738816 |
| rs2322754 | -0.0117022 | 0.831641 | 3.80E-09 | A | G | 0.00198538 | 17.68222001 |
| rs2341184 | 0.0100606 | 0.271748 | 1.70E-09 | C | T | 0.00167125 | 18.47251718 |
| rs2362972 | -0.00856859 | 0.577904 | 1.30E-08 | A | C | 0.00150767 | 16.51635056 |
| rs2389763 | -0.00832615 | 0.591848 | 3.80E-08 | C | T | 0.00151353 | 15.44358042 |
| rs246181 | 0.00970432 | 0.373429 | 3.70E-10 | T | C | 0.00154856 | 20.32078884 |
| rs248831 | 0.00988989 | 0.265259 | 8.40E-09 | A | G | 0.00171686 | 17.57991444 |
| rs2587505 | -0.0092717 | 0.419822 | 8.10E-10 | C | T | 0.0015091 | 19.30973228 |
| rs2631360 | -0.0111803 | 0.519189 | 5.40E-14 | A | G | 0.00148648 | 28.77708298 |
| rs2717351 | 0.0127695 | 0.211783 | 3.40E-12 | G | A | 0.00183473 | 25.10274388 |
| rs2854152 | 0.010991 | 0.678266 | 6.00E-12 | G | A | 0.00159765 | 24.31124461 |
| rs2871865 | -0.0236863 | 0.116217 | 1.80E-24 | G | C | 0.00232022 | 53.14625704 |
| rs2894602 | 0.0101332 | 0.766101 | 9.90E-09 | G | A | 0.00176778 | 16.96822751 |
| rs3118914 | -0.0194408 | 0.215004 | 5.90E-27 | T | G | 0.00180843 | 58.8315826 |
| rs34030812 | -0.00912858 | 0.36737 | 3.30E-09 | C | T | 0.00154266 | 17.86033574 |
| rs34217742 | 0.0146245 | 0.124284 | 1.40E-10 | A | T | 0.00227778 | 21.46711354 |
| rs34587452 | -0.0110867 | 0.215181 | 9.10E-10 | C | G | 0.00181019 | 19.14295267 |
| rs34845616 | 0.00979506 | 0.245647 | 1.70E-08 | A | G | 0.00173626 | 16.39564868 |
| rs35304341 | -0.0142862 | 0.089496 | 4.00E-08 | A | G | 0.00260278 | 15.33720611 |
| rs35457492 | 0.00833889 | 0.495364 | 2.20E-08 | C | A | 0.00149104 | 16.03050238 |
| rs35701422 | -0.00869121 | 0.625757 | 1.50E-08 | C | T | 0.00153481 | 16.31353685 |
| rs35833641 | 0.00922306 | 0.312389 | 8.50E-09 | G | A | 0.00160181 | 16.85067728 |
| rs36065733 | 0.00971991 | 0.472588 | 8.20E-11 | G | T | 0.00149596 | 21.71653397 |
| rs3771498 | 0.0141605 | 0.514178 | 1.90E-21 | T | C | 0.00148856 | 46.19598459 |
| rs3848369 | -0.00953776 | 0.38657 | 4.70E-10 | T | C | 0.00153126 | 19.89379094 |
| rs4121165 | -0.0119792 | 0.211455 | 4.80E-11 | A | G | 0.00182142 | 22.06651399 |
| rs4369779 | 0.017203 | 0.789015 | 3.40E-21 | C | T | 0.00182068 | 45.43608317 |
| rs4549685 | 0.00971494 | 0.329917 | 8.10E-10 | T | C | 0.00158124 | 19.24174862 |
| rs4553566 | -0.00930181 | 0.453271 | 4.60E-10 | C | T | 0.00149229 | 19.77408841 |
| rs4730984 | 0.0104789 | 0.239566 | 1.80E-09 | T | G | 0.00174245 | 18.44796003 |
| rs4737446 | 0.0102668 | 0.694661 | 2.40E-10 | T | G | 0.00162131 | 20.61852974 |
| rs4752689 | 0.0087531 | 0.584246 | 6.40E-09 | A | G | 0.00150761 | 17.16267596 |
| rs4768725 | 0.00905894 | 0.699597 | 2.30E-08 | C | T | 0.00162081 | 15.90504215 |
| rs4784329 | -0.0133357 | 0.426476 | 9.00E-19 | C | A | 0.00150734 | 40.11700331 |
| rs4785574 | -0.0103769 | 0.555329 | 4.50E-12 | G | A | 0.00149954 | 24.52225441 |
| rs4793658 | -0.0136707 | 0.110375 | 3.80E-08 | C | A | 0.00248614 | 16.92341539 |
| rs4802848 | 0.0110767 | 0.730261 | 3.80E-11 | C | G | 0.00167496 | 22.28827737 |
| rs4868110 | -0.00971069 | 0.323165 | 1.00E-09 | T | A | 0.00158981 | 19.02120809 |
| rs4927015 | 0.013059 | 0.582858 | 5.00E-18 | A | G | 0.00150895 | 38.23978257 |
| rs4962700 | 0.00932733 | 0.302017 | 1.20E-08 | G | C | 0.00163735 | 16.91296392 |
| rs56074046 | -0.00903502 | 0.371748 | 4.40E-09 | A | G | 0.00153954 | 17.58207752 |
| rs56144131 | -0.01307 | 0.149344 | 3.80E-10 | C | T | 0.00208666 | 20.01357383 |
| rs58670122 | -0.0132234 | 0.14316 | 7.40E-10 | G | A | 0.00214787 | 19.78058799 |
| rs6006984 | 0.0102987 | 0.277766 | 5.10E-10 | C | T | 0.00165665 | 19.62244788 |
| rs61389091 | 0.0220553 | 0.041752 | 3.60E-09 | T | C | 0.00373782 | 17.94778764 |
| rs62037412 | 0.00932966 | 0.357285 | 1.90E-09 | A | G | 0.0015542 | 18.43296033 |
| rs62234790 | 0.0105846 | 0.245816 | 8.80E-10 | A | C | 0.0017265 | 19.15434375 |
| rs62509875 | -0.0131798 | 0.170951 | 2.40E-11 | G | A | 0.00197325 | 22.70407136 |
| rs635538 | -0.0219943 | 0.913868 | 1.50E-16 | A | G | 0.00266274 | 35.11674382 |
| rs645144 | -0.00868793 | 0.329996 | 4.30E-08 | C | T | 0.00158627 | 15.39026389 |
| rs6473015 | 0.00959961 | 0.285659 | 5.50E-09 | C | A | 0.00164629 | 17.34162837 |
| rs6592737 | -0.00924791 | 0.37154 | 2.00E-09 | T | A | 0.00154126 | 18.41622559 |
| rs6693567 | -0.00974656 | 0.733032 | 6.30E-09 | T | C | 0.00167765 | 17.14406814 |
| rs6693965 | -0.016251 | 0.12803 | 3.20E-13 | T | G | 0.00223033 | 27.19017184 |
| rs6711390 | 0.0132663 | 0.37107 | 4.00E-18 | T | C | 0.00152876 | 37.87968284 |
| rs6715064 | -0.00921128 | 0.310405 | 9.60E-09 | T | C | 0.00160563 | 16.74909821 |
| rs6792762 | -0.00911683 | 0.419501 | 1.80E-09 | A | G | 0.0015155 | 18.66605943 |
| rs6870324 | -0.00998568 | 0.269673 | 3.00E-09 | G | C | 0.00168316 | 18.11090048 |
| rs6882168 | -0.00909312 | 0.337386 | 7.90E-09 | T | C | 0.00157605 | 17.04678325 |
| rs6962338 | -0.0203488 | 0.043971 | 2.00E-08 | G | A | 0.00362338 | 16.05251219 |
| rs6977081 | 0.01286 | 0.333818 | 5.70E-16 | T | G | 0.00158867 | 33.91793483 |
| rs7034200 | 0.00868874 | 0.480149 | 5.30E-09 | A | C | 0.00148875 | 17.37791854 |
| rs71298370 | 0.0165994 | 0.086217 | 8.90E-10 | A | G | 0.00270846 | 20.01946611 |
| rs7148603 | 0.00930613 | 0.359456 | 4.70E-09 | A | G | 0.00158837 | 18.38921238 |
| rs7206195 | -0.0153211 | 0.180373 | 2.80E-15 | T | C | 0.00193924 | 32.0044742 |
| rs721101 | 0.00946697 | 0.271238 | 1.60E-08 | C | T | 0.0016742 | 16.33753442 |
| rs7214252 | -0.0102442 | 0.211101 | 2.00E-08 | A | G | 0.00182532 | 16.11743204 |
| rs7249 | 0.00848186 | 0.365956 | 4.00E-08 | T | C | 0.00154442 | 15.39422542 |
| rs7266065 | 0.00993881 | 0.322982 | 4.60E-10 | A | G | 0.00159481 | 19.91952356 |
| rs72820369 | 0.0161266 | 0.117642 | 4.40E-12 | T | A | 0.00232961 | 24.89597716 |
| rs72977282 | -0.016811 | 0.413909 | 1.20E-28 | A | T | 0.00151401 | 63.23091182 |
| rs7451021 | -0.0157967 | 0.689474 | 7.70E-23 | C | T | 0.00160573 | 49.27287281 |
| rs75457267 | -0.0187295 | 0.05122 | 3.40E-08 | T | C | 0.00339456 | 15.72118013 |
| rs7549184 | 0.01056 | 0.787331 | 5.80E-09 | A | G | 0.00181367 | 17.21941801 |
| rs7565148 | -0.0103511 | 0.500863 | 3.60E-12 | G | T | 0.00148921 | 24.70289564 |
| rs7575451 | -0.0106047 | 0.649801 | 9.30E-12 | G | C | 0.00155571 | 23.60082331 |
| rs7576964 | 0.00974539 | 0.342052 | 5.30E-10 | T | G | 0.00156908 | 19.71122393 |
| rs7657558 | 0.0107255 | 0.719597 | 1.10E-10 | G | T | 0.00166228 | 21.40622715 |
| rs76749769 | 0.0144315 | 0.091199 | 2.20E-08 | T | C | 0.00257957 | 15.91877656 |
| rs76895963 | 0.0359771 | 0.020696 | 4.10E-10 | G | T | 0.00575693 | 24.19312175 |
| rs7790322 | -0.00860301 | 0.415619 | 1.20E-08 | T | C | 0.0015092 | 16.5775682 |
| rs7871404 | 0.0119345 | 0.188654 | 3.40E-10 | G | A | 0.00190074 | 20.10538459 |
| rs7963801 | -0.0112734 | 0.571983 | 8.40E-14 | C | T | 0.00151048 | 28.69418361 |
| rs8055199 | -0.00896262 | 0.660123 | 1.20E-08 | A | G | 0.00157271 | 16.62054023 |
| rs823130 | -0.0123872 | 0.432873 | 1.70E-16 | T | C | 0.00150332 | 34.74023204 |
| rs852520 | -0.00890306 | 0.662481 | 1.50E-08 | A | C | 0.00157301 | 16.344759 |
| rs911642 | 0.00862996 | 0.375746 | 2.00E-08 | T | C | 0.00153783 | 16.11021412 |
| rs9322822 | 0.0110799 | 0.320183 | 3.50E-12 | T | C | 0.00159266 | 24.64325414 |
| rs935728 | 0.00955337 | 0.327604 | 1.80E-09 | T | C | 0.0015872 | 18.54034663 |
| rs9388051 | 0.0106262 | 0.186051 | 2.70E-08 | A | G | 0.0019116 | 15.7693176 |
| rs9396861 | -0.00962972 | 0.598901 | 5.70E-10 | A | C | 0.00155347 | 20.54310845 |
| rs9652468 | -0.0125308 | 0.249359 | 3.30E-13 | A | G | 0.00172118 | 27.10524451 |
| rs9757079 | 0.00980223 | 0.318301 | 8.40E-10 | T | C | 0.00159699 | 19.22699529 |
| rs9853018 | 0.0101977 | 0.443249 | 8.80E-12 | T | C | 0.00149426 | 23.6672827 |

SNP, single nucleotide polymorphism; Beta coefficients are in standard deviation (SD) unit per allele; EAF, effect allele frequency; SE, standard error;

**Supplementary Table 13.** Characteristics of selected SNPs concerning the causal effect of right-hand grip strength on stroke

| **SNP** | **Beta** | **EAF** | ***P*-value** | **Effect**  **allele** | **Other**  **allele** | **SE** | ***F*-value** |
| --- | --- | --- | --- | --- | --- | --- | --- |
| rs10193039 | -0.0103284 | 0.28057 | 4.10E-10 | T | A | 0.00165217 | 19.85763166 |
| rs10278546 | 0.0107539 | 0.194705 | 1.10E-08 | C | A | 0.00188373 | 16.72217322 |
| rs1043515 | 0.0138281 | 0.566183 | 2.80E-20 | G | A | 0.00149848 | 43.31537564 |
| rs1047891 | 0.0096867 | 0.315772 | 1.30E-09 | A | C | 0.0015965 | 18.69634196 |
| rs10483727 | -0.00901803 | 0.610138 | 3.30E-09 | C | T | 0.00152456 | 17.83988327 |
| rs10520770 | 0.011742 | 0.44929 | 4.50E-15 | C | T | 0.00149773 | 31.4612789 |
| rs10761411 | -0.0114975 | 0.812419 | 1.30E-08 | T | C | 0.00202082 | 18.57830664 |
| rs10770125 | 0.00844132 | 0.477072 | 1.40E-08 | G | A | 0.00148775 | 16.39362012 |
| rs10784502 | -0.0111247 | 0.512218 | 7.10E-14 | T | C | 0.00148608 | 28.51654818 |
| rs10798483 | 0.0145083 | 0.54729 | 2.80E-22 | A | G | 0.00149469 | 48.09820064 |
| rs10799428 | -0.0143588 | 0.18721 | 4.60E-14 | T | C | 0.00190367 | 28.93235675 |
| rs10846071 | -0.0156575 | 0.393685 | 6.50E-25 | T | C | 0.00151902 | 53.97041108 |
| rs11022513 | -0.00921054 | 0.569312 | 1.10E-09 | T | C | 0.00151016 | 19.18289885 |
| rs11039348 | -0.00976855 | 0.348363 | 3.90E-10 | A | G | 0.00156104 | 19.97697169 |
| rs112330055 | 0.0179824 | 0.062972 | 1.70E-08 | A | G | 0.00319007 | 17.59643988 |
| rs11243202 | 0.0116378 | 0.486018 | 6.50E-15 | C | T | 0.00149304 | 31.20213448 |
| rs1125 | -0.0100304 | 0.336554 | 1.90E-10 | A | G | 0.00157472 | 20.71711267 |
| rs113315602 | -0.0212911 | 0.095826 | 1.40E-15 | C | A | 0.00266761 | 36.22251191 |
| rs113835839 | -0.00986353 | 0.247871 | 1.10E-08 | T | C | 0.00172486 | 16.72674441 |
| rs113851275 | 0.0131425 | 0.107596 | 4.30E-08 | A | G | 0.00239826 | 15.29469726 |
| rs114924396 | -0.0191251 | 0.05363 | 8.20E-09 | G | A | 0.00331785 | 17.12005633 |
| rs116922558 | -0.0246271 | 0.039907 | 1.80E-10 | G | A | 0.00386049 | 21.42999106 |
| rs11998884 | 0.0173433 | 0.061825 | 2.80E-08 | T | C | 0.0031243 | 16.08938639 |
| rs12052508 | -0.0133517 | 0.879044 | 4.70E-09 | T | C | 0.00227992 | 17.47996259 |
| rs12101479 | -0.0106133 | 0.237244 | 1.50E-09 | C | G | 0.0017554 | 18.79802963 |
| rs12316046 | -0.0162046 | 0.377959 | 3.80E-26 | G | A | 0.00153212 | 56.93860632 |
| rs12412806 | -0.00909582 | 0.294214 | 3.00E-08 | A | G | 0.00164139 | 15.84338167 |
| rs12452505 | -0.0144313 | 0.142315 | 1.40E-11 | G | C | 0.00213545 | 23.44360288 |
| rs12522139 | -0.0114381 | 0.171144 | 6.80E-09 | G | T | 0.00197332 | 17.11502492 |
| rs12562146 | 0.0118706 | 0.145156 | 2.00E-08 | A | T | 0.00211554 | 16.12483438 |
| rs12616285 | 0.0122195 | 0.148607 | 5.40E-09 | G | T | 0.00209396 | 17.42230036 |
| rs12763284 | 0.00959741 | 0.466048 | 1.20E-10 | G | A | 0.00148928 | 21.13847953 |
| rs12790261 | -0.0263714 | 0.082377 | 2.00E-22 | A | C | 0.00270734 | 48.48363489 |
| rs12823922 | -0.0114203 | 0.222184 | 1.60E-10 | G | A | 0.00178649 | 20.78629709 |
| rs12899474 | -0.0148939 | 0.108065 | 5.40E-10 | T | C | 0.00239936 | 19.71816864 |
| rs12914702 | 0.0107788 | 0.300438 | 1.90E-10 | A | G | 0.00169282 | 22.51935691 |
| rs13106087 | 0.0129259 | 0.829827 | 6.20E-11 | C | T | 0.001977 | 21.75871117 |
| rs13146142 | -0.0207968 | 0.158627 | 1.40E-24 | C | T | 0.00203165 | 53.23799948 |
| rs13169333 | 0.00929083 | 0.260299 | 4.30E-08 | C | T | 0.00169626 | 15.32727828 |
| rs13355365 | -0.00846604 | 0.379543 | 3.40E-08 | T | C | 0.00153398 | 15.5654199 |
| rs13356200 | -0.00920983 | 0.393811 | 1.80E-09 | G | T | 0.00153092 | 18.67366586 |
| rs1442883 | -0.0106189 | 0.253067 | 5.80E-10 | A | C | 0.00171426 | 19.65656439 |
| rs1486925 | -0.0095288 | 0.314649 | 2.90E-09 | C | T | 0.00160467 | 18.05700469 |
| rs150330307 | -0.0325831 | 0.031875 | 1.20E-14 | C | T | 0.00422401 | 30.2139678 |
| rs1550115 | 0.0152836 | 0.748561 | 4.50E-19 | T | C | 0.00171276 | 40.54732669 |
| rs1556659 | 0.0175345 | 0.381531 | 3.80E-30 | T | C | 0.00153689 | 66.91298949 |
| rs1641457 | 0.0130726 | 0.223327 | 2.40E-13 | G | T | 0.00178536 | 27.33644313 |
| rs1840753 | 0.0176942 | 0.062797 | 1.40E-08 | T | C | 0.00312088 | 16.99273595 |
| rs1885690 | -0.00839964 | 0.410365 | 2.80E-08 | A | C | 0.00151212 | 15.74354811 |
| rs1892425 | 0.0113357 | 0.238631 | 1.10E-10 | A | G | 0.00175726 | 21.53038972 |
| rs1952256 | 0.00976791 | 0.345023 | 4.10E-10 | G | A | 0.00156323 | 19.88424005 |
| rs2147461 | 0.0134299 | 0.117274 | 6.10E-09 | C | T | 0.00231042 | 17.21877718 |
| rs2165241 | 0.0122486 | 0.508842 | 1.90E-16 | C | T | 0.00148844 | 34.57980344 |
| rs2194411 | 0.0142831 | 0.128444 | 2.10E-10 | A | G | 0.00224795 | 21.06137802 |
| rs2194747 | 0.00985126 | 0.707014 | 1.90E-09 | G | A | 0.00163925 | 18.53910567 |
| rs2208562 | -0.0115616 | 0.609627 | 3.10E-14 | T | C | 0.00152246 | 29.33731409 |
| rs2226685 | 0.0103333 | 0.758696 | 3.10E-09 | C | T | 0.00174324 | 18.02769679 |
| rs2244621 | 0.0115957 | 0.144157 | 4.50E-08 | T | C | 0.00211936 | 15.29857794 |
| rs2273555 | 0.0110316 | 0.606106 | 4.20E-13 | A | G | 0.00152155 | 26.79434044 |
| rs2296316 | -0.00821333 | 0.464404 | 4.60E-08 | C | T | 0.00150213 | 15.4738816 |
| rs2322754 | -0.0117022 | 0.831641 | 3.80E-09 | A | G | 0.00198538 | 17.68222001 |
| rs2341184 | 0.0100606 | 0.271748 | 1.70E-09 | C | T | 0.00167125 | 18.47251718 |
| rs2362972 | -0.00856859 | 0.577904 | 1.30E-08 | A | C | 0.00150767 | 16.51635056 |
| rs2389763 | -0.00832615 | 0.591848 | 3.80E-08 | C | T | 0.00151353 | 15.44358042 |
| rs246181 | 0.00970432 | 0.373429 | 3.70E-10 | T | C | 0.00154856 | 20.32078884 |
| rs248831 | 0.00988989 | 0.265259 | 8.40E-09 | A | G | 0.00171686 | 17.57991444 |
| rs2587505 | -0.0092717 | 0.419822 | 8.10E-10 | C | T | 0.0015091 | 19.30973228 |
| rs2631360 | -0.0111803 | 0.519189 | 5.40E-14 | A | G | 0.00148648 | 28.77708298 |
| rs2717351 | 0.0127695 | 0.211783 | 3.40E-12 | G | A | 0.00183473 | 25.10274388 |
| rs2854152 | 0.010991 | 0.678266 | 6.00E-12 | G | A | 0.00159765 | 24.31124461 |
| rs2871865 | -0.0236863 | 0.116217 | 1.80E-24 | G | C | 0.00232022 | 53.14625704 |
| rs2894602 | 0.0101332 | 0.766101 | 9.90E-09 | G | A | 0.00176778 | 16.96822751 |
| rs3118914 | -0.0194408 | 0.215004 | 5.90E-27 | T | G | 0.00180843 | 58.8315826 |
| rs34030812 | -0.00912858 | 0.36737 | 3.30E-09 | C | T | 0.00154266 | 17.86033574 |
| rs34217742 | 0.0146245 | 0.124284 | 1.40E-10 | A | T | 0.00227778 | 21.46711354 |
| rs34587452 | -0.0110867 | 0.215181 | 9.10E-10 | C | G | 0.00181019 | 19.14295267 |
| rs34845616 | 0.00979506 | 0.245647 | 1.70E-08 | A | G | 0.00173626 | 16.39564868 |
| rs35175534 | -0.0191822 | 0.139977 | 4.20E-16 | C | A | 0.00235828 | 40.85215086 |
| rs35304341 | -0.0142862 | 0.089496 | 4.00E-08 | A | G | 0.00260278 | 15.33720611 |
| rs35457492 | 0.00833889 | 0.495364 | 2.20E-08 | C | A | 0.00149104 | 16.03050238 |
| rs35701422 | -0.00869121 | 0.625757 | 1.50E-08 | C | T | 0.00153481 | 16.31353685 |
| rs35833641 | 0.00922306 | 0.312389 | 8.50E-09 | G | A | 0.00160181 | 16.85067728 |
| rs36065733 | 0.00971991 | 0.472588 | 8.20E-11 | G | T | 0.00149596 | 21.71653397 |
| rs3771498 | 0.0141605 | 0.514178 | 1.90E-21 | T | C | 0.00148856 | 46.19598459 |
| rs3848369 | -0.00953776 | 0.38657 | 4.70E-10 | T | C | 0.00153126 | 19.89379094 |
| rs4121165 | -0.0119792 | 0.211455 | 4.80E-11 | A | G | 0.00182142 | 22.06651399 |
| rs4369779 | 0.017203 | 0.789015 | 3.40E-21 | C | T | 0.00182068 | 45.43608317 |
| rs4549685 | 0.00971494 | 0.329917 | 8.10E-10 | T | C | 0.00158124 | 19.24174862 |
| rs4553566 | -0.00930181 | 0.453271 | 4.60E-10 | C | T | 0.00149229 | 19.77408841 |
| rs4730984 | 0.0104789 | 0.239566 | 1.80E-09 | T | G | 0.00174245 | 18.44796003 |
| rs4737446 | 0.0102668 | 0.694661 | 2.40E-10 | T | G | 0.00162131 | 20.61852974 |
| rs4751671 | 0.0082332 | 0.530724 | 4.10E-08 | A | G | 0.00150019 | 15.56904377 |
| rs4752689 | 0.0087531 | 0.584246 | 6.40E-09 | A | G | 0.00150761 | 17.16267596 |
| rs4768725 | 0.00905894 | 0.699597 | 2.30E-08 | C | T | 0.00162081 | 15.90504215 |
| rs4784329 | -0.0133357 | 0.426476 | 9.00E-19 | C | A | 0.00150734 | 40.11700331 |
| rs4785574 | -0.0103769 | 0.555329 | 4.50E-12 | G | A | 0.00149954 | 24.52225441 |
| rs4793658 | -0.0136707 | 0.110375 | 3.80E-08 | C | A | 0.00248614 | 16.92341539 |
| rs4802848 | 0.0110767 | 0.730261 | 3.80E-11 | C | G | 0.00167496 | 22.28827737 |
| rs4868110 | -0.00971069 | 0.323165 | 1.00E-09 | T | A | 0.00158981 | 19.02120809 |
| rs4927015 | 0.013059 | 0.582858 | 5.00E-18 | A | G | 0.00150895 | 38.23978257 |
| rs4962700 | 0.00932733 | 0.302017 | 1.20E-08 | G | C | 0.00163735 | 16.91296392 |
| rs56074046 | -0.00903502 | 0.371748 | 4.40E-09 | A | G | 0.00153954 | 17.58207752 |
| rs56144131 | -0.01307 | 0.149344 | 3.80E-10 | C | T | 0.00208666 | 20.01357383 |
| rs56365901 | -0.0142618 | 0.222684 | 1.80E-15 | G | A | 0.00179293 | 32.46973618 |
| rs58670122 | -0.0132234 | 0.14316 | 7.40E-10 | G | A | 0.00214787 | 19.78058799 |
| rs6006984 | 0.0102987 | 0.277766 | 5.10E-10 | C | T | 0.00165665 | 19.62244788 |
| rs61389091 | 0.0220553 | 0.041752 | 3.60E-09 | T | C | 0.00373782 | 17.94778764 |
| rs62037412 | 0.00932966 | 0.357285 | 1.90E-09 | A | G | 0.0015542 | 18.43296033 |
| rs62234790 | 0.0105846 | 0.245816 | 8.80E-10 | A | C | 0.0017265 | 19.15434375 |
| rs62509875 | -0.0131798 | 0.170951 | 2.40E-11 | G | A | 0.00197325 | 22.70407136 |
| rs635538 | -0.0219943 | 0.913868 | 1.50E-16 | A | G | 0.00266274 | 35.11674382 |
| rs645144 | -0.00868793 | 0.329996 | 4.30E-08 | C | T | 0.00158627 | 15.39026389 |
| rs6473015 | 0.00959961 | 0.285659 | 5.50E-09 | C | A | 0.00164629 | 17.34162837 |
| rs6592737 | -0.00924791 | 0.37154 | 2.00E-09 | T | A | 0.00154126 | 18.41622559 |
| rs6693567 | -0.00974656 | 0.733032 | 6.30E-09 | T | C | 0.00167765 | 17.14406814 |
| rs6693965 | -0.016251 | 0.12803 | 3.20E-13 | T | G | 0.00223033 | 27.19017184 |
| rs6711390 | 0.0132663 | 0.37107 | 4.00E-18 | T | C | 0.00152876 | 37.87968284 |
| rs6715064 | -0.00921128 | 0.310405 | 9.60E-09 | T | C | 0.00160563 | 16.74909821 |
| rs6792762 | -0.00911683 | 0.419501 | 1.80E-09 | A | G | 0.0015155 | 18.66605943 |
| rs6870324 | -0.00998568 | 0.269673 | 3.00E-09 | G | C | 0.00168316 | 18.11090048 |
| rs6882168 | -0.00909312 | 0.337386 | 7.90E-09 | T | C | 0.00157605 | 17.04678325 |
| rs6962338 | -0.0203488 | 0.043971 | 2.00E-08 | G | A | 0.00362338 | 16.05251219 |
| rs6977081 | 0.01286 | 0.333818 | 5.70E-16 | T | G | 0.00158867 | 33.91793483 |
| rs7034200 | 0.00868874 | 0.480149 | 5.30E-09 | A | C | 0.00148875 | 17.37791854 |
| rs71298370 | 0.0165994 | 0.086217 | 8.90E-10 | A | G | 0.00270846 | 20.01946611 |
| rs7148603 | 0.00930613 | 0.359456 | 4.70E-09 | A | G | 0.00158837 | 18.38921238 |
| rs7206195 | -0.0153211 | 0.180373 | 2.80E-15 | T | C | 0.00193924 | 32.0044742 |
| rs721101 | 0.00946697 | 0.271238 | 1.60E-08 | C | T | 0.0016742 | 16.33753442 |
| rs7214252 | -0.0102442 | 0.211101 | 2.00E-08 | A | G | 0.00182532 | 16.11743204 |
| rs7249 | 0.00848186 | 0.365956 | 4.00E-08 | T | C | 0.00154442 | 15.39422542 |
| rs7266065 | 0.00993881 | 0.322982 | 4.60E-10 | A | G | 0.00159481 | 19.91952356 |
| rs72820369 | 0.0161266 | 0.117642 | 4.40E-12 | T | A | 0.00232961 | 24.89597716 |
| rs72977282 | -0.016811 | 0.413909 | 1.20E-28 | A | T | 0.00151401 | 63.23091182 |
| rs7301953 | -0.0115397 | 0.312268 | 6.40E-13 | A | G | 0.00160449 | 26.37384596 |
| rs7451021 | -0.0157967 | 0.689474 | 7.70E-23 | C | T | 0.00160573 | 49.27287281 |
| rs75457267 | -0.0187295 | 0.05122 | 3.40E-08 | T | C | 0.00339456 | 15.72118013 |
| rs7549184 | 0.01056 | 0.787331 | 5.80E-09 | A | G | 0.00181367 | 17.21941801 |
| rs7565148 | -0.0103511 | 0.500863 | 3.60E-12 | G | T | 0.00148921 | 24.70289564 |
| rs7575451 | -0.0106047 | 0.649801 | 9.30E-12 | G | C | 0.00155571 | 23.60082331 |
| rs7576964 | 0.00974539 | 0.342052 | 5.30E-10 | T | G | 0.00156908 | 19.71122393 |
| rs7657558 | 0.0107255 | 0.719597 | 1.10E-10 | G | T | 0.00166228 | 21.40622715 |
| rs76749769 | 0.0144315 | 0.091199 | 2.20E-08 | T | C | 0.00257957 | 15.91877656 |
| rs7790322 | -0.00860301 | 0.415619 | 1.20E-08 | T | C | 0.0015092 | 16.5775682 |
| rs7871404 | 0.0119345 | 0.188654 | 3.40E-10 | G | A | 0.00190074 | 20.10538459 |
| rs7963801 | -0.0112734 | 0.571983 | 8.40E-14 | C | T | 0.00151048 | 28.69418361 |
| rs8055199 | -0.00896262 | 0.660123 | 1.20E-08 | A | G | 0.00157271 | 16.62054023 |
| rs823130 | -0.0123872 | 0.432873 | 1.70E-16 | T | C | 0.00150332 | 34.74023204 |
| rs852520 | -0.00890306 | 0.662481 | 1.50E-08 | A | C | 0.00157301 | 16.344759 |
| rs911642 | 0.00862996 | 0.375746 | 2.00E-08 | T | C | 0.00153783 | 16.11021412 |
| rs9267806 | -0.0167434 | 0.255832 | 7.70E-23 | A | G | 0.00170197 | 49.22348933 |
| rs9322822 | 0.0110799 | 0.320183 | 3.50E-12 | T | C | 0.00159266 | 24.64325414 |
| rs935728 | 0.00955337 | 0.327604 | 1.80E-09 | T | C | 0.0015872 | 18.54034663 |
| rs9388051 | 0.0106262 | 0.186051 | 2.70E-08 | A | G | 0.0019116 | 15.7693176 |
| rs9396861 | -0.00962972 | 0.598901 | 5.70E-10 | A | C | 0.00155347 | 20.54310845 |
| rs9652468 | -0.0125308 | 0.249359 | 3.30E-13 | A | G | 0.00172118 | 27.10524451 |
| rs9757079 | 0.00980223 | 0.318301 | 8.40E-10 | T | C | 0.00159699 | 19.22699529 |
| rs9853018 | 0.0101977 | 0.443249 | 8.80E-12 | T | C | 0.00149426 | 23.6672827 |

SNP, single nucleotide polymorphism; Beta coefficients are in standard deviation (SD) unit per allele; EAF, effect allele frequency; SE, standard error;

**Supplementary Table 14.** Characteristics of selected SNPs concerning the causal effect of right-hand grip strength on MI

| **SNP** | **Beta** | **EAF** | ***P*-value** | **Effect**  **allele** | **Other**  **allele** | **SE** | ***F*-value** |
| --- | --- | --- | --- | --- | --- | --- | --- |
| rs10193039 | -0.0103284 | 0.28057 | 4.10E-10 | T | A | 0.00165217 | 19.85763166 |
| rs10278546 | 0.0107539 | 0.194705 | 1.10E-08 | C | A | 0.00188373 | 16.72217322 |
| rs1043515 | 0.0138281 | 0.566183 | 2.80E-20 | G | A | 0.00149848 | 43.31537564 |
| rs1047891 | 0.0096867 | 0.315772 | 1.30E-09 | A | C | 0.0015965 | 18.69634196 |
| rs10483727 | -0.00901803 | 0.610138 | 3.30E-09 | C | T | 0.00152456 | 17.83988327 |
| rs10520770 | 0.011742 | 0.44929 | 4.50E-15 | C | T | 0.00149773 | 31.4612789 |
| rs10761411 | -0.0114975 | 0.812419 | 1.30E-08 | T | C | 0.00202082 | 18.57830664 |
| rs10770125 | 0.00844132 | 0.477072 | 1.40E-08 | G | A | 0.00148775 | 16.39362012 |
| rs10784502 | -0.0111247 | 0.512218 | 7.10E-14 | T | C | 0.00148608 | 28.51654818 |
| rs10798483 | 0.0145083 | 0.54729 | 2.80E-22 | A | G | 0.00149469 | 48.09820064 |
| rs10799428 | -0.0143588 | 0.18721 | 4.60E-14 | T | C | 0.00190367 | 28.93235675 |
| rs10846071 | -0.0156575 | 0.393685 | 6.50E-25 | T | C | 0.00151902 | 53.97041108 |
| rs11022513 | -0.00921054 | 0.569312 | 1.10E-09 | T | C | 0.00151016 | 19.18289885 |
| rs11039348 | -0.00976855 | 0.348363 | 3.90E-10 | A | G | 0.00156104 | 19.97697169 |
| rs112330055 | 0.0179824 | 0.062972 | 1.70E-08 | A | G | 0.00319007 | 17.59643988 |
| rs11243202 | 0.0116378 | 0.486018 | 6.50E-15 | C | T | 0.00149304 | 31.20213448 |
| rs1125 | -0.0100304 | 0.336554 | 1.90E-10 | A | G | 0.00157472 | 20.71711267 |
| rs113315602 | -0.0212911 | 0.095826 | 1.40E-15 | C | A | 0.00266761 | 36.22251191 |
| rs113835839 | -0.00986353 | 0.247871 | 1.10E-08 | T | C | 0.00172486 | 16.72674441 |
| rs113851275 | 0.0131425 | 0.107596 | 4.30E-08 | A | G | 0.00239826 | 15.29469726 |
| rs114924396 | -0.0191251 | 0.05363 | 8.20E-09 | G | A | 0.00331785 | 17.12005633 |
| rs116922558 | -0.0246271 | 0.039907 | 1.80E-10 | G | A | 0.00386049 | 21.42999106 |
| rs11998884 | 0.0173433 | 0.061825 | 2.80E-08 | T | C | 0.0031243 | 16.08938639 |
| rs12052508 | -0.0133517 | 0.879044 | 4.70E-09 | T | C | 0.00227992 | 17.47996259 |
| rs12101479 | -0.0106133 | 0.237244 | 1.50E-09 | C | G | 0.0017554 | 18.79802963 |
| rs12316046 | -0.0162046 | 0.377959 | 3.80E-26 | G | A | 0.00153212 | 56.93860632 |
| rs12412806 | -0.00909582 | 0.294214 | 3.00E-08 | A | G | 0.00164139 | 15.84338167 |
| rs12522139 | -0.0114381 | 0.171144 | 6.80E-09 | G | T | 0.00197332 | 17.11502492 |
| rs12562146 | 0.0118706 | 0.145156 | 2.00E-08 | A | T | 0.00211554 | 16.12483438 |
| rs12616285 | 0.0122195 | 0.148607 | 5.40E-09 | G | T | 0.00209396 | 17.42230036 |
| rs12763284 | 0.00959741 | 0.466048 | 1.20E-10 | G | A | 0.00148928 | 21.13847953 |
| rs12790261 | -0.0263714 | 0.082377 | 2.00E-22 | A | C | 0.00270734 | 48.48363489 |
| rs12823922 | -0.0114203 | 0.222184 | 1.60E-10 | G | A | 0.00178649 | 20.78629709 |
| rs12899474 | -0.0148939 | 0.108065 | 5.40E-10 | T | C | 0.00239936 | 19.71816864 |
| rs12914702 | 0.0107788 | 0.300438 | 1.90E-10 | A | G | 0.00169282 | 22.51935691 |
| rs13106087 | 0.0129259 | 0.829827 | 6.20E-11 | C | T | 0.001977 | 21.75871117 |
| rs13146142 | -0.0207968 | 0.158627 | 1.40E-24 | C | T | 0.00203165 | 53.23799948 |
| rs13169333 | 0.00929083 | 0.260299 | 4.30E-08 | C | T | 0.00169626 | 15.32727828 |
| rs13355365 | -0.00846604 | 0.379543 | 3.40E-08 | T | C | 0.00153398 | 15.5654199 |
| rs13356200 | -0.00920983 | 0.393811 | 1.80E-09 | G | T | 0.00153092 | 18.67366586 |
| rs1486925 | -0.0095288 | 0.314649 | 2.90E-09 | C | T | 0.00160467 | 18.05700469 |
| rs150330307 | -0.0325831 | 0.031875 | 1.20E-14 | C | T | 0.00422401 | 30.2139678 |
| rs1550115 | 0.0152836 | 0.748561 | 4.50E-19 | T | C | 0.00171276 | 40.54732669 |
| rs1556659 | 0.0175345 | 0.381531 | 3.80E-30 | T | C | 0.00153689 | 66.91298949 |
| rs1641457 | 0.0130726 | 0.223327 | 2.40E-13 | G | T | 0.00178536 | 27.33644313 |
| rs1840753 | 0.0176942 | 0.062797 | 1.40E-08 | T | C | 0.00312088 | 16.99273595 |
| rs1885690 | -0.00839964 | 0.410365 | 2.80E-08 | A | C | 0.00151212 | 15.74354811 |
| rs1892425 | 0.0113357 | 0.238631 | 1.10E-10 | A | G | 0.00175726 | 21.53038972 |
| rs1952256 | 0.00976791 | 0.345023 | 4.10E-10 | G | A | 0.00156323 | 19.88424005 |
| rs2147461 | 0.0134299 | 0.117274 | 6.10E-09 | C | T | 0.00231042 | 17.21877718 |
| rs2165241 | 0.0122486 | 0.508842 | 1.90E-16 | C | T | 0.00148844 | 34.57980344 |
| rs2194411 | 0.0142831 | 0.128444 | 2.10E-10 | A | G | 0.00224795 | 21.06137802 |
| rs2194747 | 0.00985126 | 0.707014 | 1.90E-09 | G | A | 0.00163925 | 18.53910567 |
| rs2208562 | -0.0115616 | 0.609627 | 3.10E-14 | T | C | 0.00152246 | 29.33731409 |
| rs2244621 | 0.0115957 | 0.144157 | 4.50E-08 | T | C | 0.00211936 | 15.29857794 |
| rs2273555 | 0.0110316 | 0.606106 | 4.20E-13 | A | G | 0.00152155 | 26.79434044 |
| rs2296316 | -0.00821333 | 0.464404 | 4.60E-08 | C | T | 0.00150213 | 15.4738816 |
| rs2322754 | -0.0117022 | 0.831641 | 3.80E-09 | A | G | 0.00198538 | 17.68222001 |
| rs2341184 | 0.0100606 | 0.271748 | 1.70E-09 | C | T | 0.00167125 | 18.47251718 |
| rs2362972 | -0.00856859 | 0.577904 | 1.30E-08 | A | C | 0.00150767 | 16.51635056 |
| rs2389763 | -0.00832615 | 0.591848 | 3.80E-08 | C | T | 0.00151353 | 15.44358042 |
| rs246181 | 0.00970432 | 0.373429 | 3.70E-10 | T | C | 0.00154856 | 20.32078884 |
| rs248831 | 0.00988989 | 0.265259 | 8.40E-09 | A | G | 0.00171686 | 17.57991444 |
| rs2587505 | -0.0092717 | 0.419822 | 8.10E-10 | C | T | 0.0015091 | 19.30973228 |
| rs2631360 | -0.0111803 | 0.519189 | 5.40E-14 | A | G | 0.00148648 | 28.77708298 |
| rs2717351 | 0.0127695 | 0.211783 | 3.40E-12 | G | A | 0.00183473 | 25.10274388 |
| rs2854152 | 0.010991 | 0.678266 | 6.00E-12 | G | A | 0.00159765 | 24.31124461 |
| rs2871865 | -0.0236863 | 0.116217 | 1.80E-24 | G | C | 0.00232022 | 53.14625704 |
| rs2894602 | 0.0101332 | 0.766101 | 9.90E-09 | G | A | 0.00176778 | 16.96822751 |
| rs3118914 | -0.0194408 | 0.215004 | 5.90E-27 | T | G | 0.00180843 | 58.8315826 |
| rs34030812 | -0.00912858 | 0.36737 | 3.30E-09 | C | T | 0.00154266 | 17.86033574 |
| rs34217742 | 0.0146245 | 0.124284 | 1.40E-10 | A | T | 0.00227778 | 21.46711354 |
| rs34587452 | -0.0110867 | 0.215181 | 9.10E-10 | C | G | 0.00181019 | 19.14295267 |
| rs34845616 | 0.00979506 | 0.245647 | 1.70E-08 | A | G | 0.00173626 | 16.39564868 |
| rs35175534 | -0.0191822 | 0.139977 | 4.20E-16 | C | A | 0.00235828 | 40.85215086 |
| rs35304341 | -0.0142862 | 0.089496 | 4.00E-08 | A | G | 0.00260278 | 15.33720611 |
| rs35457492 | 0.00833889 | 0.495364 | 2.20E-08 | C | A | 0.00149104 | 16.03050238 |
| rs35701422 | -0.00869121 | 0.625757 | 1.50E-08 | C | T | 0.00153481 | 16.31353685 |
| rs35833641 | 0.00922306 | 0.312389 | 8.50E-09 | G | A | 0.00160181 | 16.85067728 |
| rs36065733 | 0.00971991 | 0.472588 | 8.20E-11 | G | T | 0.00149596 | 21.71653397 |
| rs3771498 | 0.0141605 | 0.514178 | 1.90E-21 | T | C | 0.00148856 | 46.19598459 |
| rs3848369 | -0.00953776 | 0.38657 | 4.70E-10 | T | C | 0.00153126 | 19.89379094 |
| rs4121165 | -0.0119792 | 0.211455 | 4.80E-11 | A | G | 0.00182142 | 22.06651399 |
| rs4369779 | 0.017203 | 0.789015 | 3.40E-21 | C | T | 0.00182068 | 45.43608317 |
| rs4549685 | 0.00971494 | 0.329917 | 8.10E-10 | T | C | 0.00158124 | 19.24174862 |
| rs4553566 | -0.00930181 | 0.453271 | 4.60E-10 | C | T | 0.00149229 | 19.77408841 |
| rs4730984 | 0.0104789 | 0.239566 | 1.80E-09 | T | G | 0.00174245 | 18.44796003 |
| rs4737446 | 0.0102668 | 0.694661 | 2.40E-10 | T | G | 0.00162131 | 20.61852974 |
| rs4751671 | 0.0082332 | 0.530724 | 4.10E-08 | A | G | 0.00150019 | 15.56904377 |
| rs4752689 | 0.0087531 | 0.584246 | 6.40E-09 | A | G | 0.00150761 | 17.16267596 |
| rs4768725 | 0.00905894 | 0.699597 | 2.30E-08 | C | T | 0.00162081 | 15.90504215 |
| rs4784329 | -0.0133357 | 0.426476 | 9.00E-19 | C | A | 0.00150734 | 40.11700331 |
| rs4785574 | -0.0103769 | 0.555329 | 4.50E-12 | G | A | 0.00149954 | 24.52225441 |
| rs4793658 | -0.0136707 | 0.110375 | 3.80E-08 | C | A | 0.00248614 | 16.92341539 |
| rs4802848 | 0.0110767 | 0.730261 | 3.80E-11 | C | G | 0.00167496 | 22.28827737 |
| rs4868110 | -0.00971069 | 0.323165 | 1.00E-09 | T | A | 0.00158981 | 19.02120809 |
| rs4927015 | 0.013059 | 0.582858 | 5.00E-18 | A | G | 0.00150895 | 38.23978257 |
| rs4962700 | 0.00932733 | 0.302017 | 1.20E-08 | G | C | 0.00163735 | 16.91296392 |
| rs56074046 | -0.00903502 | 0.371748 | 4.40E-09 | A | G | 0.00153954 | 17.58207752 |
| rs56144131 | -0.01307 | 0.149344 | 3.80E-10 | C | T | 0.00208666 | 20.01357383 |
| rs56365901 | -0.0142618 | 0.222684 | 1.80E-15 | G | A | 0.00179293 | 32.46973618 |
| rs58670122 | -0.0132234 | 0.14316 | 7.40E-10 | G | A | 0.00214787 | 19.78058799 |
| rs6006984 | 0.0102987 | 0.277766 | 5.10E-10 | C | T | 0.00165665 | 19.62244788 |
| rs61389091 | 0.0220553 | 0.041752 | 3.60E-09 | T | C | 0.00373782 | 17.94778764 |
| rs62037412 | 0.00932966 | 0.357285 | 1.90E-09 | A | G | 0.0015542 | 18.43296033 |
| rs62234790 | 0.0105846 | 0.245816 | 8.80E-10 | A | C | 0.0017265 | 19.15434375 |
| rs62509875 | -0.0131798 | 0.170951 | 2.40E-11 | G | A | 0.00197325 | 22.70407136 |
| rs635538 | -0.0219943 | 0.913868 | 1.50E-16 | A | G | 0.00266274 | 35.11674382 |
| rs645144 | -0.00868793 | 0.329996 | 4.30E-08 | C | T | 0.00158627 | 15.39026389 |
| rs6473015 | 0.00959961 | 0.285659 | 5.50E-09 | C | A | 0.00164629 | 17.34162837 |
| rs6592737 | -0.00924791 | 0.37154 | 2.00E-09 | T | A | 0.00154126 | 18.41622559 |
| rs6693567 | -0.00974656 | 0.733032 | 6.30E-09 | T | C | 0.00167765 | 17.14406814 |
| rs6693965 | -0.016251 | 0.12803 | 3.20E-13 | T | G | 0.00223033 | 27.19017184 |
| rs6711390 | 0.0132663 | 0.37107 | 4.00E-18 | T | C | 0.00152876 | 37.87968284 |
| rs6715064 | -0.00921128 | 0.310405 | 9.60E-09 | T | C | 0.00160563 | 16.74909821 |
| rs6792762 | -0.00911683 | 0.419501 | 1.80E-09 | A | G | 0.0015155 | 18.66605943 |
| rs6870324 | -0.00998568 | 0.269673 | 3.00E-09 | G | C | 0.00168316 | 18.11090048 |
| rs6882168 | -0.00909312 | 0.337386 | 7.90E-09 | T | C | 0.00157605 | 17.04678325 |
| rs6962338 | -0.0203488 | 0.043971 | 2.00E-08 | G | A | 0.00362338 | 16.05251219 |
| rs6977081 | 0.01286 | 0.333818 | 5.70E-16 | T | G | 0.00158867 | 33.91793483 |
| rs7034200 | 0.00868874 | 0.480149 | 5.30E-09 | A | C | 0.00148875 | 17.37791854 |
| rs71298370 | 0.0165994 | 0.086217 | 8.90E-10 | A | G | 0.00270846 | 20.01946611 |
| rs7148603 | 0.00930613 | 0.359456 | 4.70E-09 | A | G | 0.00158837 | 18.38921238 |
| rs7196917 | -0.010582 | 0.429932 | 1.90E-12 | G | A | 0.00150287 | 25.31037974 |
| rs7206195 | -0.0153211 | 0.180373 | 2.80E-15 | T | C | 0.00193924 | 32.0044742 |
| rs721101 | 0.00946697 | 0.271238 | 1.60E-08 | C | T | 0.0016742 | 16.33753442 |
| rs7214252 | -0.0102442 | 0.211101 | 2.00E-08 | A | G | 0.00182532 | 16.11743204 |
| rs7249 | 0.00848186 | 0.365956 | 4.00E-08 | T | C | 0.00154442 | 15.39422542 |
| rs7266065 | 0.00993881 | 0.322982 | 4.60E-10 | A | G | 0.00159481 | 19.91952356 |
| rs72820369 | 0.0161266 | 0.117642 | 4.40E-12 | T | A | 0.00232961 | 24.89597716 |
| rs72977282 | -0.016811 | 0.413909 | 1.20E-28 | A | T | 0.00151401 | 63.23091182 |
| rs7451021 | -0.0157967 | 0.689474 | 7.70E-23 | C | T | 0.00160573 | 49.27287281 |
| rs75457267 | -0.0187295 | 0.05122 | 3.40E-08 | T | C | 0.00339456 | 15.72118013 |
| rs7549184 | 0.01056 | 0.787331 | 5.80E-09 | A | G | 0.00181367 | 17.21941801 |
| rs7565148 | -0.0103511 | 0.500863 | 3.60E-12 | G | T | 0.00148921 | 24.70289564 |
| rs7575451 | -0.0106047 | 0.649801 | 9.30E-12 | G | C | 0.00155571 | 23.60082331 |
| rs7576964 | 0.00974539 | 0.342052 | 5.30E-10 | T | G | 0.00156908 | 19.71122393 |
| rs7657558 | 0.0107255 | 0.719597 | 1.10E-10 | G | T | 0.00166228 | 21.40622715 |
| rs76749769 | 0.0144315 | 0.091199 | 2.20E-08 | T | C | 0.00257957 | 15.91877656 |
| rs76895963 | 0.0359771 | 0.020696 | 4.10E-10 | G | T | 0.00575693 | 24.19312175 |
| rs77485342 | 0.0353373 | 0.018008 | 2.60E-10 | T | C | 0.0055898 | 20.36442785 |
| rs7790322 | -0.00860301 | 0.415619 | 1.20E-08 | T | C | 0.0015092 | 16.5775682 |
| rs7871404 | 0.0119345 | 0.188654 | 3.40E-10 | G | A | 0.00190074 | 20.10538459 |
| rs7963801 | -0.0112734 | 0.571983 | 8.40E-14 | C | T | 0.00151048 | 28.69418361 |
| rs79723785 | -0.0340504 | 0.016495 | 1.60E-08 | C | T | 0.00602877 | 17.34612771 |
| rs8055199 | -0.00896262 | 0.660123 | 1.20E-08 | A | G | 0.00157271 | 16.62054023 |
| rs823130 | -0.0123872 | 0.432873 | 1.70E-16 | T | C | 0.00150332 | 34.74023204 |
| rs852520 | -0.00890306 | 0.662481 | 1.50E-08 | A | C | 0.00157301 | 16.344759 |
| rs911642 | 0.00862996 | 0.375746 | 2.00E-08 | T | C | 0.00153783 | 16.11021412 |
| rs9267806 | -0.0167434 | 0.255832 | 7.70E-23 | A | G | 0.00170197 | 49.22348933 |
| rs9322822 | 0.0110799 | 0.320183 | 3.50E-12 | T | C | 0.00159266 | 24.64325414 |
| rs935728 | 0.00955337 | 0.327604 | 1.80E-09 | T | C | 0.0015872 | 18.54034663 |
| rs9388051 | 0.0106262 | 0.186051 | 2.70E-08 | A | G | 0.0019116 | 15.7693176 |
| rs9396861 | -0.00962972 | 0.598901 | 5.70E-10 | A | C | 0.00155347 | 20.54310845 |
| rs9652468 | -0.0125308 | 0.249359 | 3.30E-13 | A | G | 0.00172118 | 27.10524451 |
| rs9757079 | 0.00980223 | 0.318301 | 8.40E-10 | T | C | 0.00159699 | 19.22699529 |
| rs9853018 | 0.0101977 | 0.443249 | 8.80E-12 | T | C | 0.00149426 | 23.6672827 |

SNP, single nucleotide polymorphism; Beta coefficients are in standard deviation (SD) unit per allele; EAF, effect allele frequency; SE, standard error;

**Supplementary Table 15.** Characteristics of selected SNPs concerning the causal effect of CHD on ALM

| **SNP** | **Beta** | **EAF** | ***P*-value** | **Effect**  **allele** | **Other**  **allele** | **SE** | ***F*-value** |
| --- | --- | --- | --- | --- | --- | --- | --- |
| rs10080815 | 0.246627 | 0.027558 | 1.33E-15 | G | T | 0.0308579 | 602.7999025 |
| rs11556924 | -0.072569 | 0.313325 | 5.34E-11 | T | C | 0.0110605 | 418.5971163 |
| rs12202017 | -0.066813 | 0.300047 | 1.98E-11 | G | A | 0.0099612 | 346.2243405 |
| rs1412444 | 0.066812 | 0.369131 | 5.15E-12 | T | C | 0.0096809 | 383.9678822 |
| rs16986953 | 0.08516 | 0.104706 | 1.45E-08 | A | G | 0.0150265 | 250.9353549 |
| rs17678683 | 0.098786 | 0.087681 | 3.00E-09 | G | T | 0.0166548 | 288.1933717 |
| rs180803 | -0.180923 | 0.029268 | 1.64E-10 | T | G | 0.0283062 | 343.44001 |
| rs1870634 | 0.075878 | 0.637485 | 5.55E-15 | G | T | 0.0097113 | 491.7533581 |
| rs2128739 | -0.065565 | 0.676464 | 7.05E-11 | C | A | 0.0100568 | 347.449585 |
| rs2487928 | 0.062633 | 0.418221 | 4.41E-11 | A | G | 0.0095049 | 352.502787 |
| rs2519093 | 0.079704 | 0.190872 | 1.19E-11 | T | C | 0.0117524 | 362.3557119 |
| rs2681472 | 0.074114 | 0.201306 | 6.17E-11 | G | A | 0.0113331 | 326.1127855 |
| rs2891168 | 0.193401 | 0.488668 | 2.29E-98 | G | A | 0.0091877 | 3510.682277 |
| rs4420638 | 0.091906 | 0.166036 | 7.07E-11 | G | A | 0.0140977 | 432.1323579 |
| rs4468572 | 0.077234 | 0.585831 | 4.44E-16 | C | T | 0.0095277 | 535.0425862 |
| rs4593108 | -0.07083 | 0.204651 | 8.82E-10 | G | C | 0.0115558 | 301.4937812 |
| rs55730499 | 0.316641 | 0.056243 | 5.39E-39 | T | C | 0.0242403 | 1982.771377 |
| rs6689306 | -0.056012 | 0.552455 | 2.60E-09 | G | A | 0.0094061 | 286.3732638 |
| rs7212798 | 0.079961 | 0.146516 | 1.88E-08 | C | T | 0.0142216 | 295.1850059 |
| rs8042271 | -0.096711 | 0.097718 | 3.68E-08 | A | G | 0.0175662 | 304.4723665 |
| rs9349379 | 0.131836 | 0.431606 | 1.81E-42 | G | A | 0.0096527 | 1585.210116 |
| rs9970807 | -0.12575 | 0.084903 | 5.00E-14 | T | C | 0.016695 | 453.9802987 |

SNP, single nucleotide polymorphism; Beta coefficients are in standard deviation (SD) unit per allele; EAF, effect allele frequency; SE, standard error;

**Supplementary Table 16.** Characteristics of selected SNPs concerning the causal effect of CHD on left-hand grip strength

| **SNP** | **Beta** | **EAF** | ***P*-value** | **Effect**  **allele** | **Other**  **allele** | **SE** | ***F*-value** |
| --- | --- | --- | --- | --- | --- | --- | --- |
| rs10080815 | 0.246627 | 0.027558 | 1.33E-15 | G | T | 0.0308579 | 602.7999025 |
| rs10840293 | 0.054714 | 0.549821 | 1.28E-08 | A | G | 0.009619 | 273.5331506 |
| rs11556924 | -0.072569 | 0.313325 | 5.34E-11 | T | C | 0.0110605 | 418.5971163 |
| rs115654617 | 0.137846 | 0.106962 | 3.12E-18 | A | C | 0.0158314 | 671.4745993 |
| rs11838776 | 0.068566 | 0.263277 | 1.83E-10 | A | G | 0.0107552 | 336.736307 |
| rs12202017 | -0.066813 | 0.300047 | 1.98E-11 | G | A | 0.0099612 | 346.2243405 |
| rs1412444 | 0.066812 | 0.369131 | 5.15E-12 | T | C | 0.0096809 | 383.9678822 |
| rs16986953 | 0.08516 | 0.104706 | 1.45E-08 | A | G | 0.0150265 | 250.9353549 |
| rs17087335 | 0.060764 | 0.214637 | 4.59E-08 | T | G | 0.0111159 | 229.7053345 |
| rs17678683 | 0.098786 | 0.087681 | 3.00E-09 | G | T | 0.0166548 | 288.1933717 |
| rs1870634 | 0.075878 | 0.637485 | 5.55E-15 | G | T | 0.0097113 | 491.7533581 |
| rs2128739 | -0.065565 | 0.676464 | 7.05E-11 | C | A | 0.0100568 | 347.449585 |
| rs2487928 | 0.062633 | 0.418221 | 4.41E-11 | A | G | 0.0095049 | 352.502787 |
| rs2681472 | 0.074114 | 0.201306 | 6.17E-11 | G | A | 0.0113331 | 326.1127855 |
| rs28451064 | 0.127571 | 0.121186 | 1.33E-15 | A | G | 0.015952 | 641.0970929 |
| rs2891168 | 0.193401 | 0.488668 | 2.29E-98 | G | A | 0.0091877 | 3510.682277 |
| rs3918226 | 0.133315 | 0.064515 | 1.69E-09 | T | C | 0.0221275 | 396.2330081 |
| rs4420638 | 0.091906 | 0.166036 | 7.07E-11 | G | A | 0.0140977 | 432.1323579 |
| rs4468572 | 0.077234 | 0.585831 | 4.44E-16 | C | T | 0.0095277 | 535.0425862 |
| rs4593108 | -0.07083 | 0.204651 | 8.82E-10 | G | C | 0.0115558 | 301.4937812 |
| rs55730499 | 0.316641 | 0.056243 | 5.39E-39 | T | C | 0.0242403 | 1982.771377 |
| rs56062135 | -0.069743 | 0.205729 | 4.52E-09 | T | C | 0.0118937 | 293.4396437 |
| rs56289821 | -0.13361 | 0.100378 | 4.44E-15 | A | G | 0.0170415 | 596.130577 |
| rs56336142 | -0.066813 | 0.192738 | 1.85E-08 | C | T | 0.0118763 | 256.3715927 |
| rs6689306 | -0.056012 | 0.552455 | 2.60E-09 | G | A | 0.0094061 | 286.3732638 |
| rs67180937 | 0.078807 | 0.663052 | 1.01E-12 | G | T | 0.0110551 | 512.8723962 |
| rs8042271 | -0.096711 | 0.097718 | 3.68E-08 | A | G | 0.0175662 | 304.4723665 |
| rs9970807 | -0.12575 | 0.084903 | 5.00E-14 | T | C | 0.016695 | 453.9802987 |

SNP, single nucleotide polymorphism; Beta coefficients are in standard deviation (SD) unit per allele; EAF, effect allele frequency; SE, standard error;

**Supplementary Table 17.** Characteristics of selected SNPs concerning the causal effect of CHD on right-hand grip strength

| **SNP** | **Beta** | **EAF** | ***P*-value** | **Effect**  **allele** | **Other**  **allele** | **SE** | ***F*-value** |
| --- | --- | --- | --- | --- | --- | --- | --- |
| rs10080815 | 0.246627 | 0.027558 | 160687412 | G | T | 0.0308579 | 602.7999025 |
| rs10840293 | 0.054714 | 0.549821 | 9751196 | A | G | 0.009619 | 273.5331506 |
| rs11556924 | -0.072569 | 0.313325 | 129663496 | T | C | 0.0110605 | 418.5971163 |
| rs115654617 | 0.137846 | 0.106962 | 203893999 | A | C | 0.0158314 | 671.4745993 |
| rs11838776 | 0.068566 | 0.263277 | 111040681 | A | G | 0.0107552 | 336.736307 |
| rs12202017 | -0.066813 | 0.300047 | 134173151 | G | A | 0.0099612 | 346.2243405 |
| rs1412444 | 0.066812 | 0.369131 | 91002927 | T | C | 0.0096809 | 383.9678822 |
| rs16986953 | 0.08516 | 0.104706 | 19942473 | A | G | 0.0150265 | 250.9353549 |
| rs17087335 | 0.060764 | 0.214637 | 57838583 | T | G | 0.0111159 | 229.7053345 |
| rs17678683 | 0.098786 | 0.087681 | 145286559 | G | T | 0.0166548 | 288.1933717 |
| rs1870634 | 0.075878 | 0.637485 | 44480811 | G | T | 0.0097113 | 491.7533581 |
| rs2128739 | -0.065565 | 0.676464 | 103673277 | C | A | 0.0100568 | 347.449585 |
| rs2487928 | 0.062633 | 0.418221 | 30323892 | A | G | 0.0095049 | 352.502787 |
| rs2681472 | 0.074114 | 0.201306 | 90008959 | G | A | 0.0113331 | 326.1127855 |
| rs28451064 | 0.127571 | 0.121186 | 35593827 | A | G | 0.015952 | 641.0970929 |
| rs2891168 | 0.193401 | 0.488668 | 22098619 | G | A | 0.0091877 | 3510.682277 |
| rs3918226 | 0.133315 | 0.064515 | 150690176 | T | C | 0.0221275 | 396.2330081 |
| rs4420638 | 0.091906 | 0.166036 | 45422946 | G | A | 0.0140977 | 432.1323579 |
| rs4468572 | 0.077234 | 0.585831 | 79124475 | C | T | 0.0095277 | 535.0425862 |
| rs4593108 | -0.07083 | 0.204651 | 148281001 | G | C | 0.0115558 | 301.4937812 |
| rs55730499 | 0.316641 | 0.056243 | 161005610 | T | C | 0.0242403 | 1982.771377 |
| rs56062135 | -0.069743 | 0.205729 | 67455630 | T | C | 0.0118937 | 293.4396437 |
| rs56289821 | -0.13361 | 0.100378 | 11188247 | A | G | 0.0170415 | 596.130577 |
| rs56336142 | -0.066813 | 0.192738 | 39134099 | C | T | 0.0118763 | 256.3715927 |
| rs6689306 | -0.056012 | 0.552455 | 154395946 | G | A | 0.0094061 | 286.3732638 |
| rs67180937 | 0.078807 | 0.663052 | 222823743 | G | T | 0.0110551 | 512.8723962 |
| rs8042271 | -0.096711 | 0.097718 | 89574218 | A | G | 0.0175662 | 304.4723665 |
| rs9970807 | -0.12575 | 0.084903 | 56965664 | T | C | 0.016695 | 453.9802987 |

SNP, single nucleotide polymorphism; Beta coefficients are in standard deviation (SD) unit per allele; EAF, effect allele frequency; SE, standard error;

**Supplementary Table 18.** Characteristics of selected SNPs concerning the causal effect of stroke on ALM

| **SNP** | **Beta** | **EAF** | ***P*-value** | **Effect**  **allele** | **Other**  **allele** | **SE** | ***F*-value** |
| --- | --- | --- | --- | --- | --- | --- | --- |
| rs1052053 | -0.0576 | 0.401 | 4.48E-11 | G | A | 0.0087 | 702.9318763 |
| rs11957829 | -0.0719 | 0.1761 | 7.51E-09 | G | A | 0.0124 | 661.5275595 |
| rs4959130 | 0.0832 | 0.1372 | 2.83E-09 | A | G | 0.014 | 722.8156653 |
| rs6825454 | 0.0564 | 0.3078 | 7.43E-10 | C | T | 0.0092 | 597.6567304 |
| rs6847935 | 0.0784 | 0.3257 | 3.50E-16 | T | A | 0.0096 | 1192.014305 |
| rs9526212 | 0.0615 | 0.7614 | 9.19E-10 | G | A | 0.0101 | 605.9476503 |
| rs9909858 | 0.0893 | 0.188 | 3.63E-08 | C | T | 0.0162 | 1074.681546 |

SNP, single nucleotide polymorphism; Beta coefficients are in standard deviation (SD) unit per allele; EAF, effect allele frequency; SE, standard error;

**Supplementary Table 19.** Characteristics of selected SNPs concerning the causal effect of stroke on left-hand grip strength

| **SNP** | **Beta** | **EAF** | ***P*-value** | **Effect**  **allele** | **Other**  **allele** | **SE** | ***F*-value** |
| --- | --- | --- | --- | --- | --- | --- | --- |
| rs1053007 | 0.0479 | 0.6506 | 3.58E-08 | G | A | 0.0087 | 459.7964075 |
| rs17035646 | 0.0536 | 0.405 | 1.34E-09 | A | G | 0.0088 | 610.530894 |
| rs35436 | -0.0495 | 0.3814 | 3.21E-08 | T | C | 0.0089 | 509.6919183 |
| rs4959130 | 0.0832 | 0.1372 | 2.83E-09 | A | G | 0.014 | 722.8156653 |
| rs6825454 | 0.0564 | 0.3078 | 7.43E-10 | C | T | 0.0092 | 597.6567304 |
| rs7304841 | -0.0484 | 0.407 | 4.93E-08 | C | A | 0.0089 | 498.4659665 |
| rs7859727 | 0.0514 | 0.5355 | 1.05E-09 | T | C | 0.0084 | 579.4913197 |
| rs9909858 | 0.0893 | 0.188 | 3.63E-08 | C | T | 0.0162 | 1074.681546 |

SNP, single nucleotide polymorphism; Beta coefficients are in standard deviation (SD) unit per allele; EAF, effect allele frequency; SE, standard error;

**Supplementary Table 20.** Characteristics of selected SNPs concerning the causal effect of stroke on right-hand grip strength

| **SNP** | **Beta** | **EAF** | ***P*-value** | **Effect**  **allele** | **Other**  **allele** | **SE** | ***F*-value** |
| --- | --- | --- | --- | --- | --- | --- | --- |
| rs1053007 | 0.0479 | 0.6506 | 3.58E-08 | G | A | 0.0087 | 459.7964075 |
| rs12445022 | 0.0609 | 0.3057 | 1.28E-10 | A | G | 0.0095 | 694.3297831 |
| rs35436 | -0.0495 | 0.3814 | 3.21E-08 | T | C | 0.0089 | 509.6919183 |
| rs6825454 | 0.0564 | 0.3078 | 7.43E-10 | C | T | 0.0092 | 597.6567304 |
| rs6847935 | 0.0784 | 0.3257 | 3.50E-16 | T | A | 0.0096 | 1192.014305 |
| rs7859727 | 0.0514 | 0.5355 | 1.05E-09 | T | C | 0.0084 | 579.4913197 |
| rs9909858 | 0.0893 | 0.188 | 3.63E-08 | C | T | 0.0162 | 1074.681546 |

SNP, single nucleotide polymorphism; Beta coefficients are in standard deviation (SD) unit per allele; EAF, effect allele frequency; SE, standard error;

**Supplementary Table 21.** Characteristics of selected SNPs concerning the causal effect of MI on ALM

| **SNP** | **Beta** | **EAF** | ***P*-value** | **Effect**  **allele** | **Other**  **allele** | **SE** | ***F*-value** |
| --- | --- | --- | --- | --- | --- | --- | --- |
| rs10455872 | 0.327656 | 0.074987 | 4.00E-58 | G | A | 0.0203866 | 5983.909758 |
| rs1065853 | -0.162512 | 0.080252 | 1.90E-16 | T | G | 0.0197495 | 1549.138356 |
| rs10841443 | 0.0645606 | 0.658908 | 1.80E-08 | G | C | 0.0114748 | 742.9232721 |
| rs11617955 | -0.10376 | 0.111065 | 2.20E-09 | A | T | 0.017346 | 843.1978589 |
| rs1412444 | 0.0674457 | 0.344095 | 2.30E-09 | T | C | 0.011283 | 814.3640732 |
| rs146712778 | -0.108664 | 0.104337 | 8.10E-10 | TAA | T | 0.017691 | 875.4109337 |
| rs1704221 | -0.109198 | 0.107122 | 3.60E-10 | A | G | 0.0174122 | 904.8780141 |
| rs1966248 | -0.0890783 | 0.309381 | 3.20E-14 | T | A | 0.0117383 | 1346.632921 |
| rs7011846 | 0.201311 | 0.032209 | 3.30E-10 | A | G | 0.0320376 | 1002.514314 |
| rs7485656 | 0.0844413 | 0.151356 | 3.00E-08 | G | A | 0.0152378 | 726.3222681 |
| rs77870048 | -0.138162 | 0.050246 | 1.90E-08 | T | C | 0.0245663 | 722.4036089 |
| rs9893777 | -0.0618277 | 0.465205 | 1.10E-08 | T | G | 0.0108146 | 754.2630735 |

SNP, single nucleotide polymorphism; Beta coefficients are in standard deviation (SD) unit per allele; EAF, effect allele frequency; SE, standard error;

**Supplementary Table 22.** Characteristics of selected SNPs concerning the causal effect of MI on left-hand grip strength

| **SNP** | **Beta** | **EAF** | ***P*-value** | **Effect**  **allele** | **Other**  **allele** | **SE** | ***F*-value** |
| --- | --- | --- | --- | --- | --- | --- | --- |
| rs10455872 | 0.327656 | 0.074987 | 4.00E-58 | G | A | 0.0203866 | 5983.909758 |
| rs10841443 | 0.0645606 | 0.658908 | 1.80E-08 | G | C | 0.0114748 | 742.9232721 |
| rs11591147 | -0.270964 | 0.016784 | 8.10E-11 | T | G | 0.0416959 | 961.4337913 |
| rs11617955 | -0.10376 | 0.111065 | 2.20E-09 | A | T | 0.017346 | 843.1978589 |
| rs11632963 | 0.0640451 | 0.505476 | 2.50E-09 | A | G | 0.0107396 | 813.2973009 |
| rs117733303 | 0.456742 | 0.018334 | 3.90E-30 | G | A | 0.0400419 | 2994.5701 |
| rs1384705 | -0.0782861 | 0.706091 | 3.20E-11 | T | C | 0.0117986 | 1009.362399 |
| rs1412444 | 0.0674457 | 0.344095 | 2.30E-09 | T | C | 0.011283 | 814.3640732 |
| rs1704221 | -0.109198 | 0.107122 | 3.60E-10 | A | G | 0.0174122 | 904.8780141 |
| rs1964600 | 0.0691276 | 0.247629 | 3.20E-08 | A | G | 0.0124958 | 706.006352 |
| rs1966248 | -0.0890783 | 0.309381 | 3.20E-14 | T | A | 0.0117383 | 1346.632921 |
| rs28451064 | 0.113339 | 0.126529 | 6.80E-12 | A | G | 0.0165161 | 1127.016608 |
| rs2886722 | 0.0669274 | 0.40358 | 1.10E-09 | G | A | 0.0109925 | 855.3135453 |
| rs2891168 | 0.188202 | 0.483196 | 2.00E-68 | G | A | 0.0107654 | 7127.663722 |
| rs2954021 | -0.0617065 | 0.506775 | 8.90E-09 | G | A | 0.0107304 | 754.8274096 |
| rs3127580 | 0.093356 | 0.150844 | 4.90E-10 | T | C | 0.015 | 885.663671 |
| rs3918226 | 0.12 | 0.077561 | 3.80E-09 | T | C | 0.0203657 | 817.217093 |
| rs4846384 | 0.0929653 | 0.691031 | 3.10E-15 | G | C | 0.011788 | 1466.081175 |
| rs7011846 | 0.201311 | 0.032209 | 3.30E-10 | A | G | 0.0320376 | 1002.514314 |
| rs7137258 | 0.125799 | 0.061975 | 3.80E-08 | A | C | 0.0228806 | 729.5973776 |
| rs73015016 | -0.104569 | 0.123328 | 1.70E-10 | A | G | 0.0163826 | 938.060682 |
| rs7485656 | 0.0844413 | 0.151356 | 3.00E-08 | G | A | 0.0152378 | 726.3222681 |
| rs77870048 | -0.138162 | 0.050246 | 1.90E-08 | T | C | 0.0245663 | 722.4036089 |
| rs9893777 | -0.0618277 | 0.465205 | 1.10E-08 | T | G | 0.0108146 | 754.2630735 |

SNP, single nucleotide polymorphism; Beta coefficients are in standard deviation (SD) unit per allele; EAF, effect allele frequency; SE, standard error;

**Supplementary Table 23.** Characteristics of selected SNPs concerning the causal effect of MI on right-hand grip strength

| **SNP** | **Beta** | **EAF** | ***P*-value** | **Effect**  **allele** | **Other**  **allele** | **SE** | ***F*-value** |
| --- | --- | --- | --- | --- | --- | --- | --- |
| rs11591147 | -0.270964 | 0.016784 | 8.10E-11 | T | G | 0.0416959 | 961.4337913 |
| rs11617955 | -0.10376 | 0.111065 | 2.20E-09 | A | T | 0.017346 | 843.1978589 |
| rs11632963 | 0.0640451 | 0.505476 | 2.50E-09 | A | G | 0.0107396 | 813.2973009 |
| rs117733303 | 0.456742 | 0.018334 | 3.90E-30 | G | A | 0.0400419 | 2994.5701 |
| rs1384705 | -0.0782861 | 0.706091 | 3.20E-11 | T | C | 0.0117986 | 1009.362399 |
| rs1412444 | 0.0674457 | 0.344095 | 2.30E-09 | T | C | 0.011283 | 814.3640732 |
| rs1704221 | -0.109198 | 0.107122 | 3.60E-10 | A | G | 0.0174122 | 904.8780141 |
| rs1964600 | 0.0691276 | 0.247629 | 3.20E-08 | A | G | 0.0124958 | 706.006352 |
| rs1966248 | -0.0890783 | 0.309381 | 3.20E-14 | T | A | 0.0117383 | 1346.632921 |
| rs28451064 | 0.113339 | 0.126529 | 6.80E-12 | A | G | 0.0165161 | 1127.016608 |
| rs2886722 | 0.0669274 | 0.40358 | 1.10E-09 | G | A | 0.0109925 | 855.3135453 |
| rs2891168 | 0.188202 | 0.483196 | 2.00E-68 | G | A | 0.0107654 | 7127.663722 |
| rs2954021 | -0.0617065 | 0.506775 | 8.90E-09 | G | A | 0.0107304 | 754.8274096 |
| rs3918226 | 0.12 | 0.077561 | 3.80E-09 | T | C | 0.0203657 | 817.217093 |
| rs4846384 | 0.0929653 | 0.691031 | 3.10E-15 | G | C | 0.011788 | 1466.081175 |
| rs7011846 | 0.201311 | 0.032209 | 3.30E-10 | A | G | 0.0320376 | 1002.514314 |
| rs7137258 | 0.125799 | 0.061975 | 3.80E-08 | A | C | 0.0228806 | 729.5973776 |
| rs73015016 | -0.104569 | 0.123328 | 1.70E-10 | A | G | 0.0163826 | 938.060682 |
| rs77870048 | -0.138162 | 0.050246 | 1.90E-08 | T | C | 0.0245663 | 722.4036089 |
| rs9893777 | -0.0618277 | 0.465205 | 1.10E-08 | T | G | 0.0108146 | 754.2630735 |

SNP, single nucleotide polymorphism; Beta coefficients are in standard deviation (SD) unit per allele; EAF, effect allele frequency; SE, standard error;

**Supplementary Table 24.** The lost SNPs after merging outcome and exposure.

| **Exposures** | **Outcomes** | **The number of lost SNPs after merging outcome and exposure** |
| --- | --- | --- |
| ALM | CHD | 51 |
|  | Stroke | 61 |
|  | MI | 40 |
| Left-hand grip strength | CHD | 5 |
|  | Stroke | 4 |
|  | MI | 0 |
| Right-hand grip strength | CHD | 6 |
|  | Stroke | 6 |
|  | MI | 0 |
| CHD | ALM | 0 |
|  | Left-hand grip strength | 0 |
|  | Right-hand grip strength | 0 |
| Stroke | ALM | 0 |
|  | Left-hand grip strength | 0 |
|  | Right-hand grip strength | 0 |
| MI | ALM | 0 |
|  | Left-hand grip strength | 2 |
|  | Right-hand grip strength | 2 |

ALM, appendicular lean mass; CHD, coronary heart disease; MI, myocardial infarction;
