# Supplementary figures and images for: Association between sarcopenia-related traits and cardiovascular diseases: a bi-directional Mendelian randomization study
Source: Front Endocrinol (Lausanne). 2023 Oct 13;14:1237971. doi: 10.3389/fendo.2023.1237971 (PMC10613058; doi:10.3389/fendo.2023.1237971)

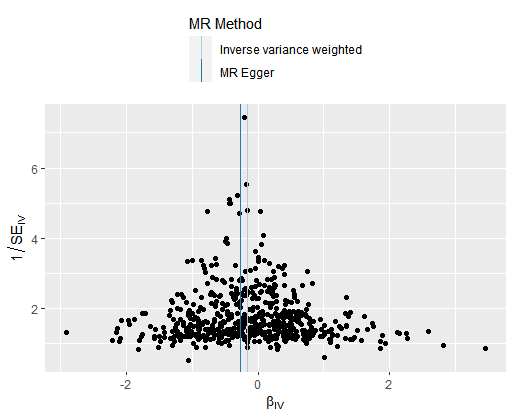

Supplement: Supplementary file 1 [file DataSheet_1.zip › Supplementary Figures/Funnel plot figure/ALM on CHD.png]

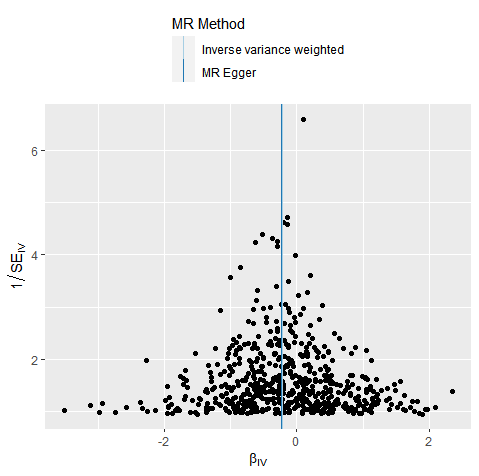

Supplement: Supplementary file 1 [file DataSheet_1.zip › Supplementary Figures/Funnel plot figure/ALM on MI.png]

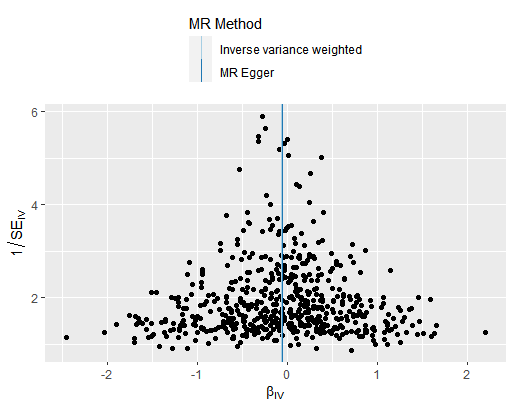

Supplement: Supplementary file 1 [file DataSheet_1.zip › Supplementary Figures/Funnel plot figure/ALM on Stroke.png]

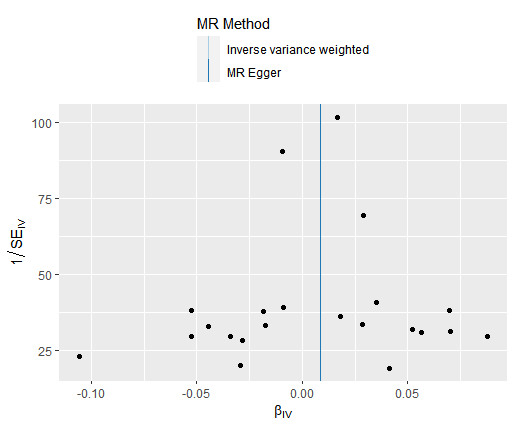

Supplement: Supplementary file 1 [file DataSheet_1.zip › Supplementary Figures/Funnel plot figure/CHD on ALM.png]

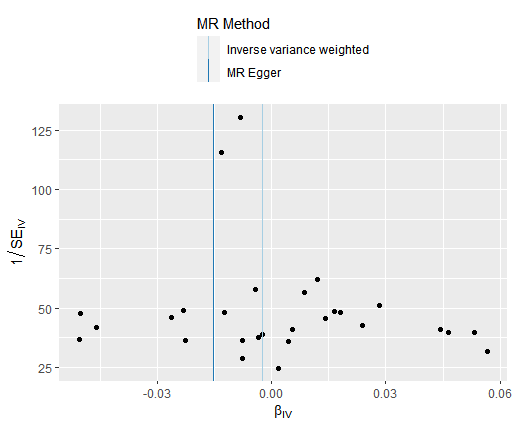

Supplement: Supplementary file 1 [file DataSheet_1.zip › Supplementary Figures/Funnel plot figure/CHD on Left-hand grip strength.png]

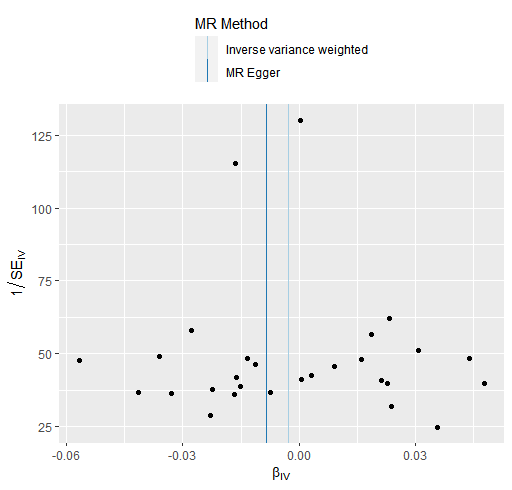

Supplement: Supplementary file 1 [file DataSheet_1.zip › Supplementary Figures/Funnel plot figure/CHD on Right-hand grip strength.png]

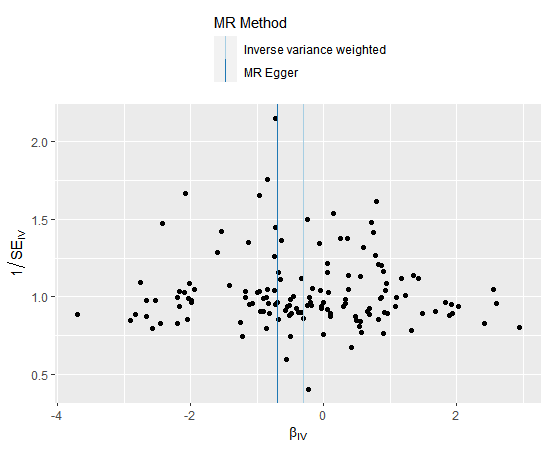

Supplement: Supplementary file 1 [file DataSheet_1.zip › Supplementary Figures/Funnel plot figure/Left-hand grip strength on CHD.png]

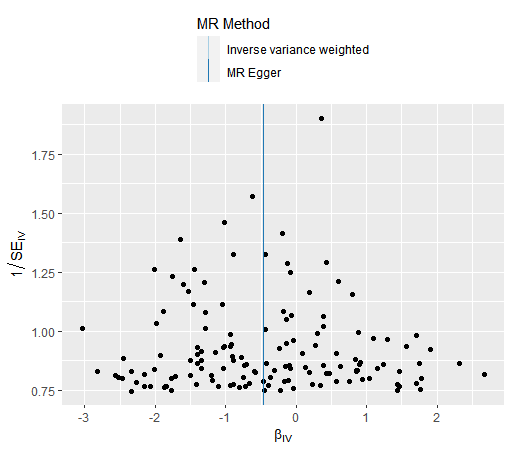

Supplement: Supplementary file 1 [file DataSheet_1.zip › Supplementary Figures/Funnel plot figure/Left-hand grip strength on MI.png]

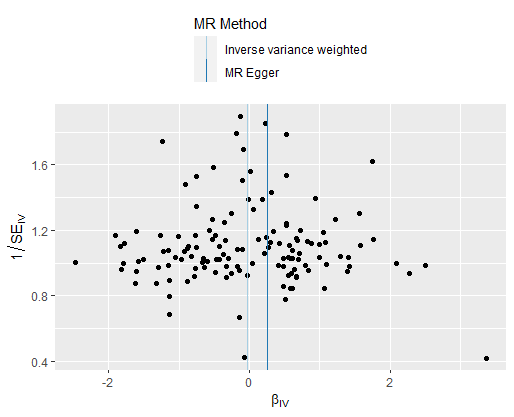

Supplement: Supplementary file 1 [file DataSheet_1.zip › Supplementary Figures/Funnel plot figure/Left-hand grip strength on Stroke.png]

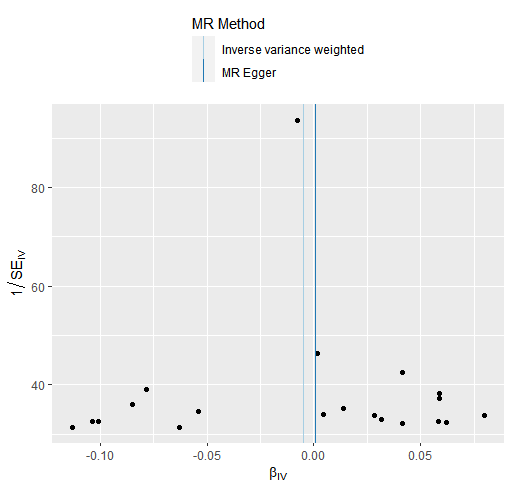

Supplement: Supplementary file 1 [file DataSheet_1.zip › Supplementary Figures/Funnel plot figure/MI on ALM.png]

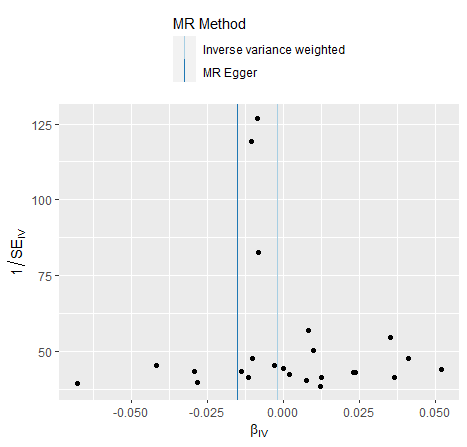

Supplement: Supplementary file 1 [file DataSheet_1.zip › Supplementary Figures/Funnel plot figure/MI on Left-hand grip strength.png]

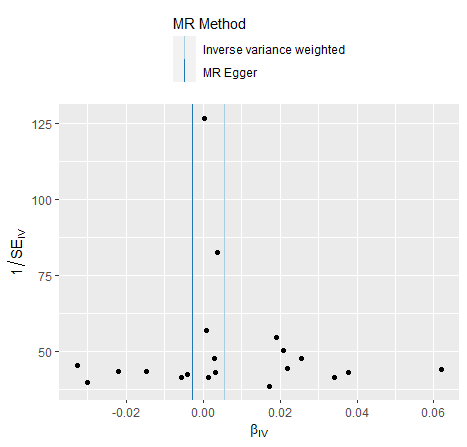

Supplement: Supplementary file 1 [file DataSheet_1.zip › Supplementary Figures/Funnel plot figure/MI on Right-hand grip strength.png]

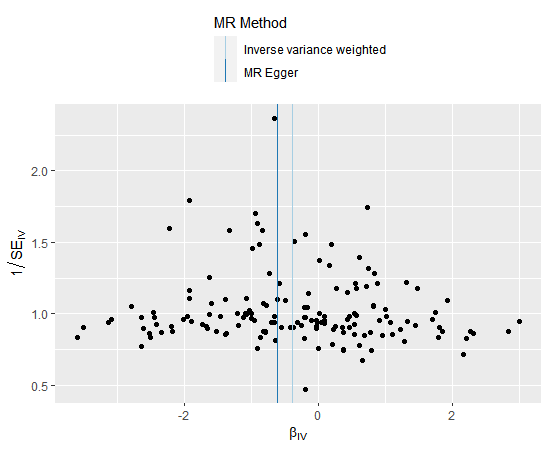

Supplement: Supplementary file 1 [file DataSheet_1.zip › Supplementary Figures/Funnel plot figure/Right-hand grip strength on CHD.png]

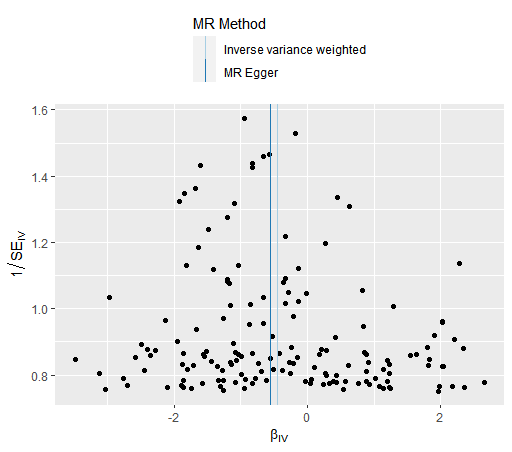

Supplement: Supplementary file 1 [file DataSheet_1.zip › Supplementary Figures/Funnel plot figure/Right-hand grip strength on MI.png]

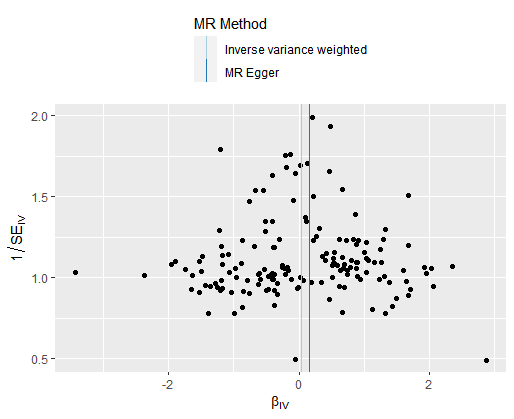

Supplement: Supplementary file 1 [file DataSheet_1.zip › Supplementary Figures/Funnel plot figure/Right-hand grip strength on Stroke.png]

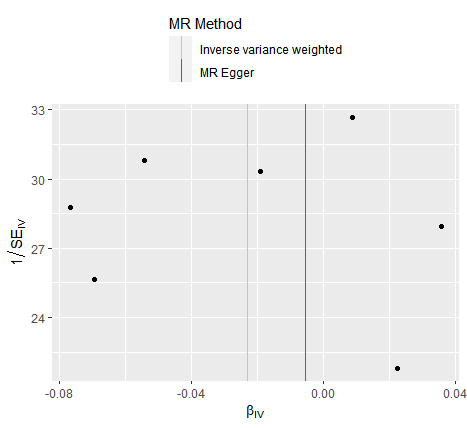

Supplement: Supplementary file 1 [file DataSheet_1.zip › Supplementary Figures/Funnel plot figure/Stroke on ALM.png]

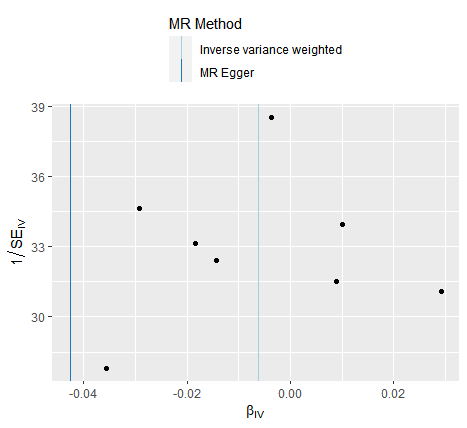

Supplement: Supplementary file 1 [file DataSheet_1.zip › Supplementary Figures/Funnel plot figure/Stroke on Left-hand grip strength.png]

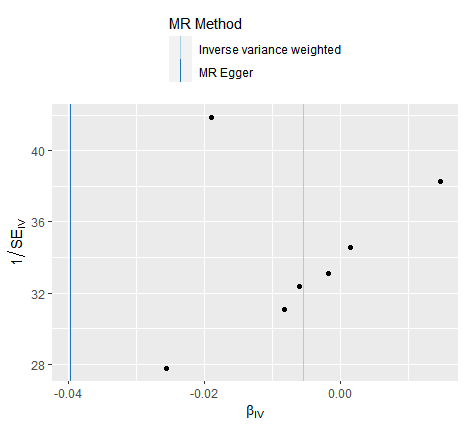

Supplement: Supplementary file 1 [file DataSheet_1.zip › Supplementary Figures/Funnel plot figure/Stroke on Right-hand grip strength.png]

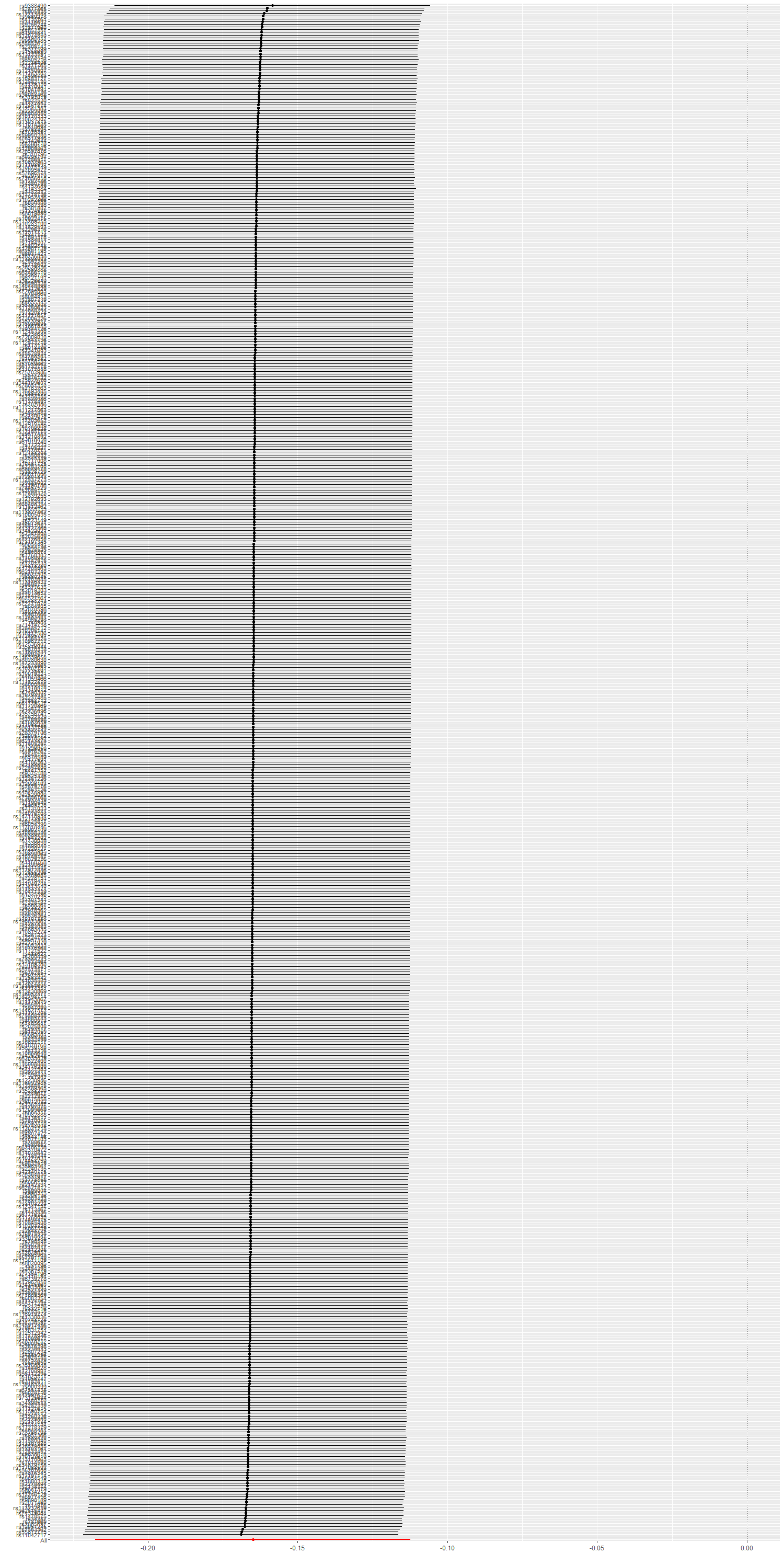

Supplement: Supplementary file 1 [file DataSheet_1.zip › Supplementary Figures/leave-one-out sensitivity analysis/ALM on CHD.png]

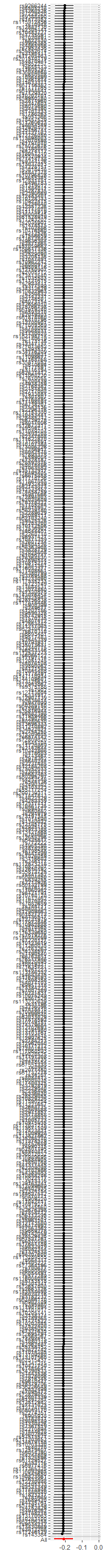

Supplement: Supplementary file 1 [file DataSheet_1.zip › Supplementary Figures/leave-one-out sensitivity analysis/ALM on MI.png]

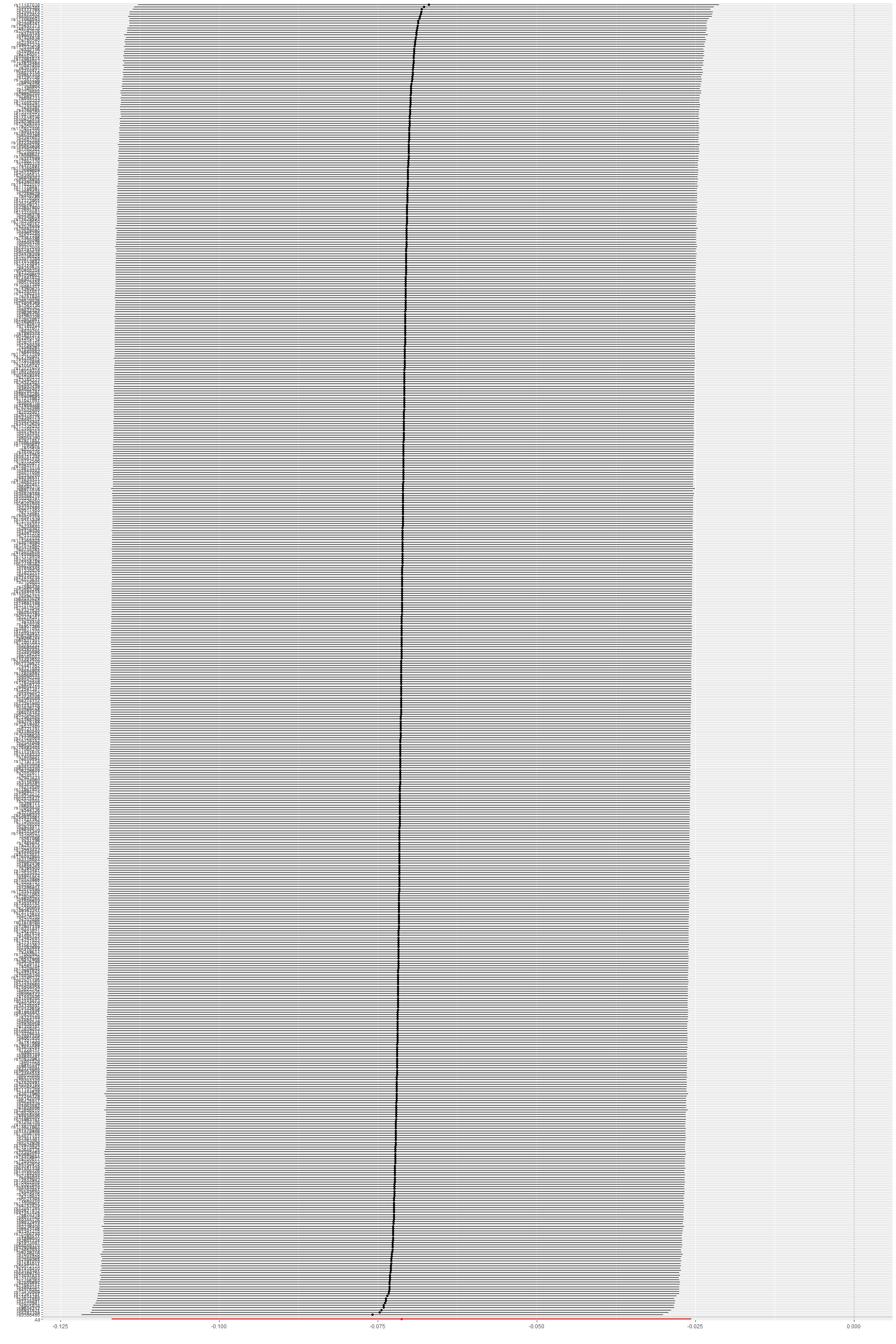

Supplement: Supplementary file 1 [file DataSheet_1.zip › Supplementary Figures/leave-one-out sensitivity analysis/ALM on Stroke.png]

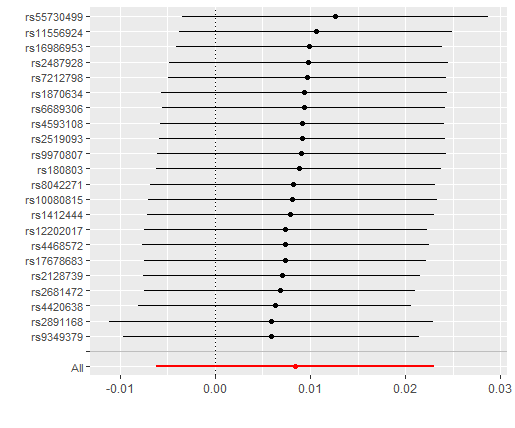

Supplement: Supplementary file 1 [file DataSheet_1.zip › Supplementary Figures/leave-one-out sensitivity analysis/CHD on ALM.png]

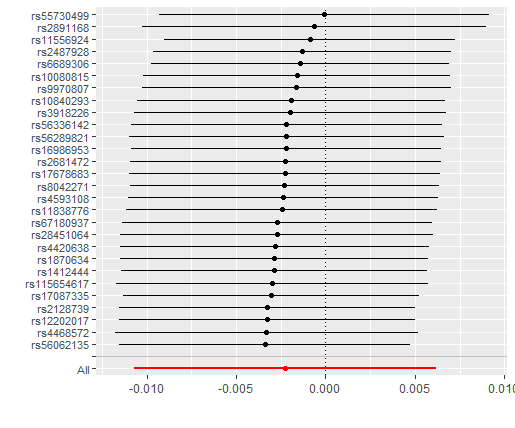

Supplement: Supplementary file 1 [file DataSheet_1.zip › Supplementary Figures/leave-one-out sensitivity analysis/CHD on Left-hand grip strength.png]

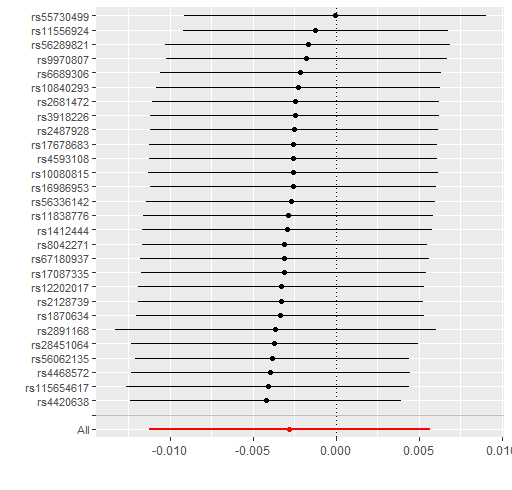

Supplement: Supplementary file 1 [file DataSheet_1.zip › Supplementary Figures/leave-one-out sensitivity analysis/CHD on Right-hand grip strength.png]

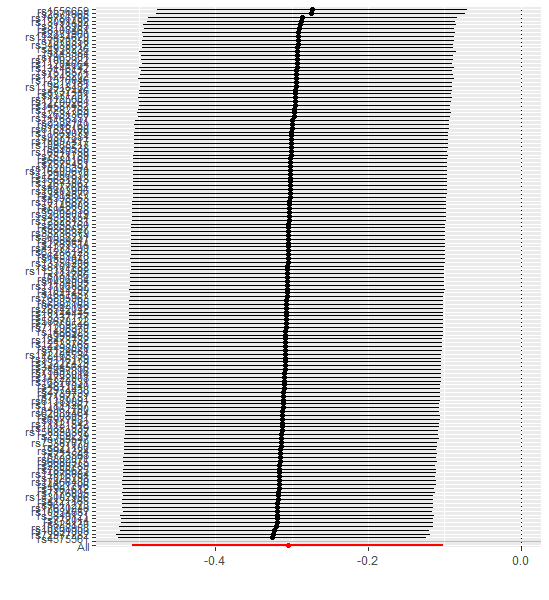

Supplement: Supplementary file 1 [file DataSheet_1.zip › Supplementary Figures/leave-one-out sensitivity analysis/Left-hand grip strength on CHD.png]

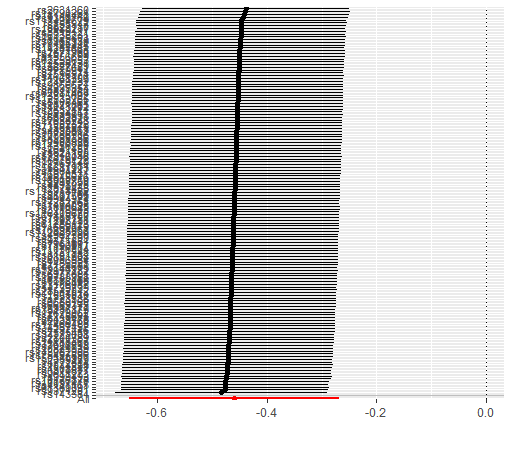

Supplement: Supplementary file 1 [file DataSheet_1.zip › Supplementary Figures/leave-one-out sensitivity analysis/Left-hand grip strength on MI.png]

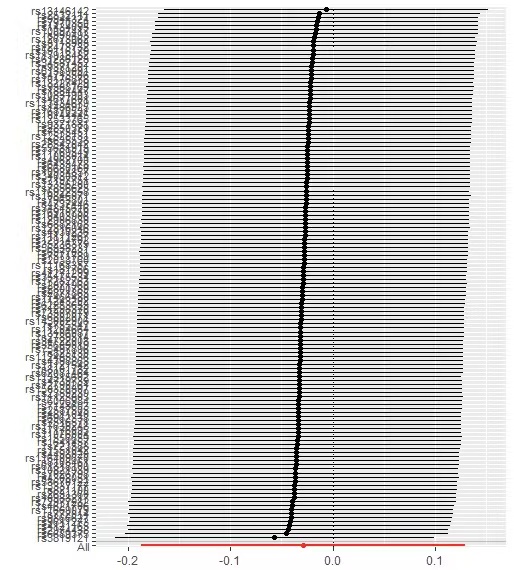

Supplement: Supplementary file 1 [file DataSheet_1.zip › Supplementary Figures/leave-one-out sensitivity analysis/Left-hand grip strength on Stroke.jpg]

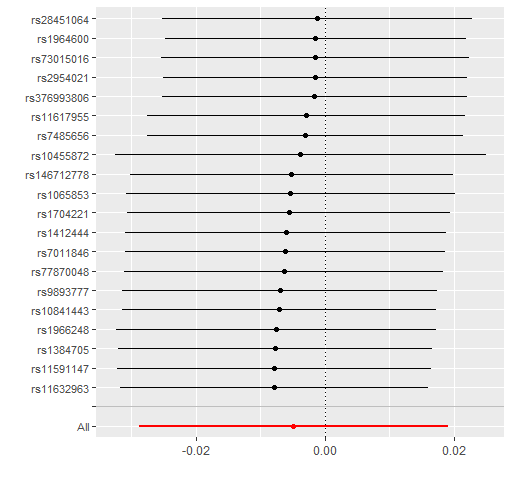

Supplement: Supplementary file 1 [file DataSheet_1.zip › Supplementary Figures/leave-one-out sensitivity analysis/MI on ALM.png]

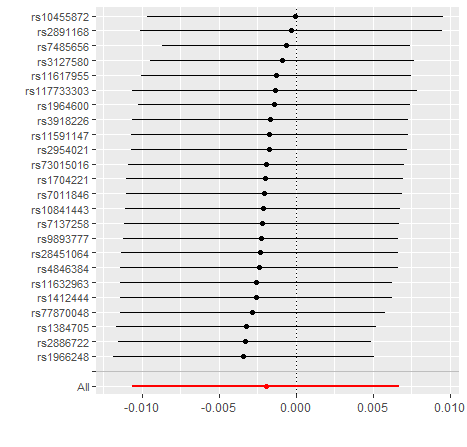

Supplement: Supplementary file 1 [file DataSheet_1.zip › Supplementary Figures/leave-one-out sensitivity analysis/MI on Left-hand grip strength.png]

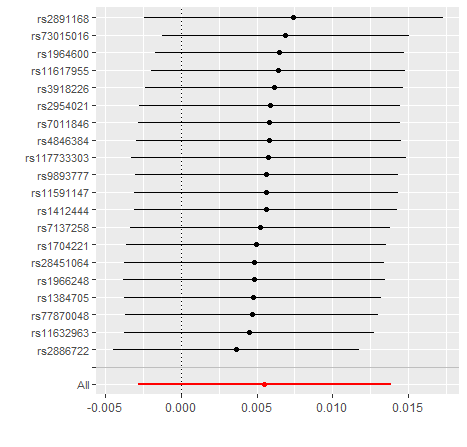

Supplement: Supplementary file 1 [file DataSheet_1.zip › Supplementary Figures/leave-one-out sensitivity analysis/MI on Right-hand grip strength.png]

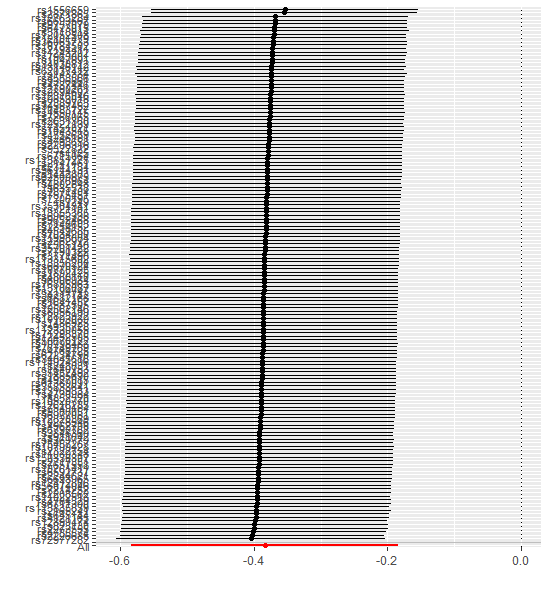

Supplement: Supplementary file 1 [file DataSheet_1.zip › Supplementary Figures/leave-one-out sensitivity analysis/Right-hand grip strength on CHD.png]

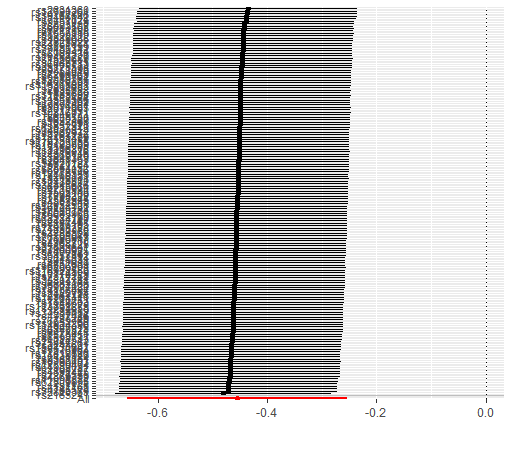

Supplement: Supplementary file 1 [file DataSheet_1.zip › Supplementary Figures/leave-one-out sensitivity analysis/Right-hand grip strength on MI.png]

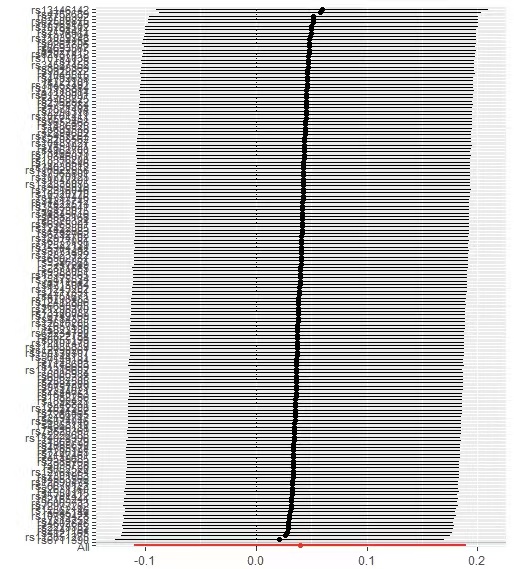

Supplement: Supplementary file 1 [file DataSheet_1.zip › Supplementary Figures/leave-one-out sensitivity analysis/Right-hand grip strength on Stroke.jpg]

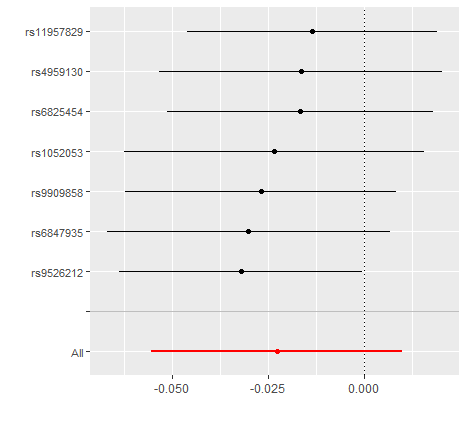

Supplement: Supplementary file 1 [file DataSheet_1.zip › Supplementary Figures/leave-one-out sensitivity analysis/Stroke on ALM.png]

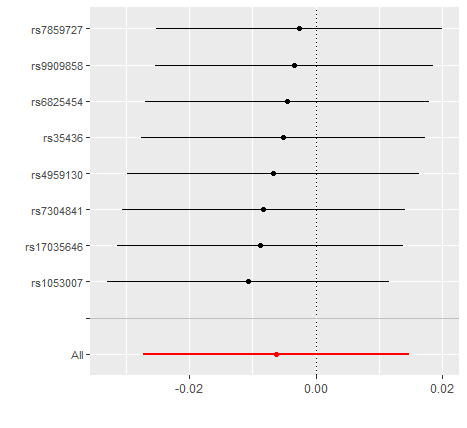

Supplement: Supplementary file 1 [file DataSheet_1.zip › Supplementary Figures/leave-one-out sensitivity analysis/Stroke on Left-hand grip strength.png]

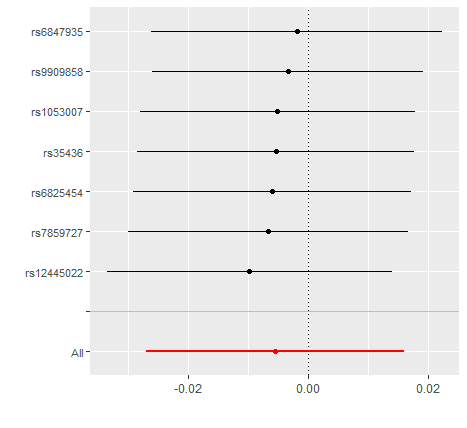

Supplement: Supplementary file 1 [file DataSheet_1.zip › Supplementary Figures/leave-one-out sensitivity analysis/Stroke on Right-hand grip strength.png]

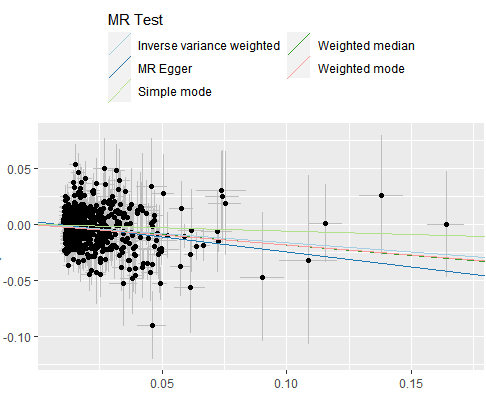

Supplement: Supplementary file 1 [file DataSheet_1.zip › Supplementary Figures/Scatter plot figure/ALM on CHD.png]

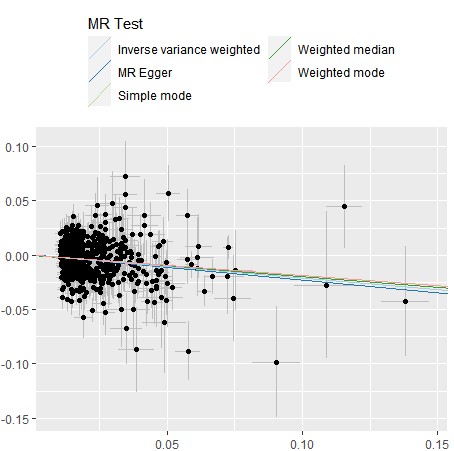

Supplement: Supplementary file 1 [file DataSheet_1.zip › Supplementary Figures/Scatter plot figure/ALM on MI.jpg]

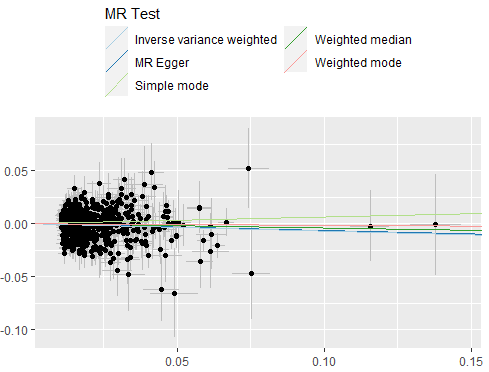

Supplement: Supplementary file 1 [file DataSheet_1.zip › Supplementary Figures/Scatter plot figure/ALM on Stroke.png]

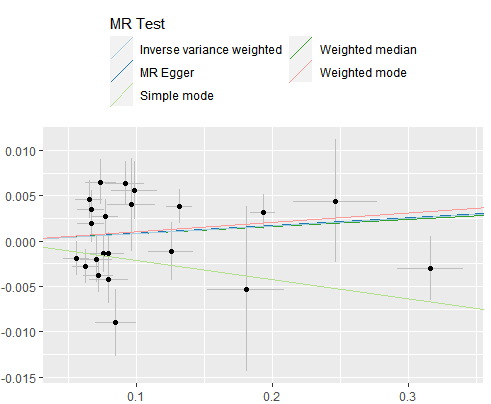

Supplement: Supplementary file 1 [file DataSheet_1.zip › Supplementary Figures/Scatter plot figure/CHD on ALM.png]

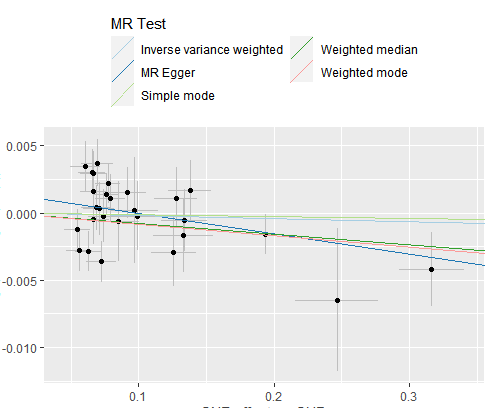

Supplement: Supplementary file 1 [file DataSheet_1.zip › Supplementary Figures/Scatter plot figure/CHD on Left-hand grip strength.png]

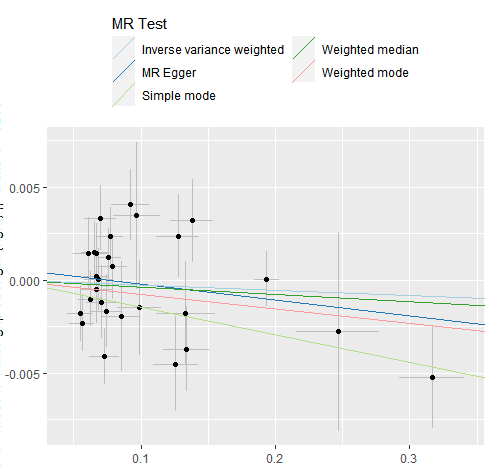

Supplement: Supplementary file 1 [file DataSheet_1.zip › Supplementary Figures/Scatter plot figure/CHD on RHGS.png]

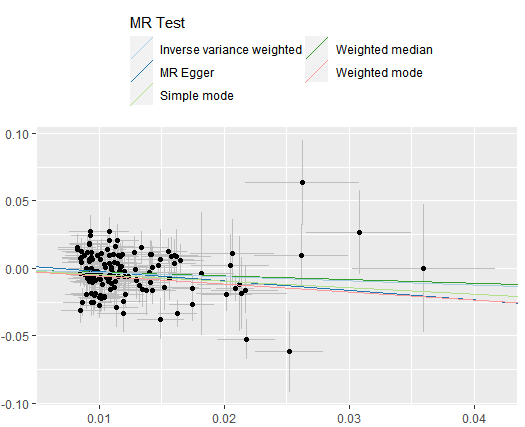

Supplement: Supplementary file 1 [file DataSheet_1.zip › Supplementary Figures/Scatter plot figure/Left-hand grip strength on CHD.png]

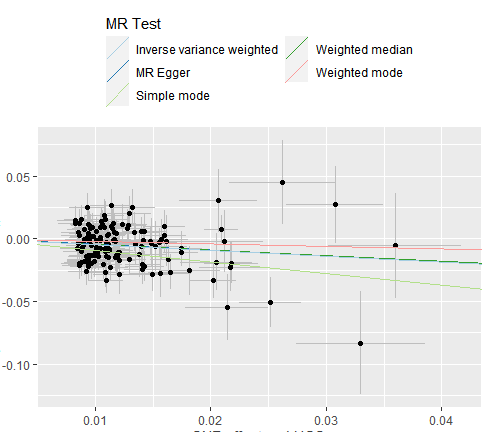

Supplement: Supplementary file 1 [file DataSheet_1.zip › Supplementary Figures/Scatter plot figure/Left-hand grip strength on MI.png]

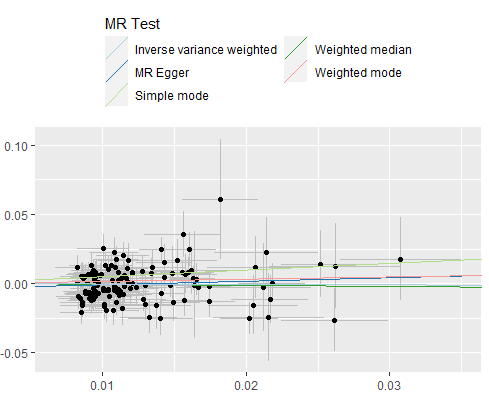

Supplement: Supplementary file 1 [file DataSheet_1.zip › Supplementary Figures/Scatter plot figure/Left-hand grip strength on Stroke.png]

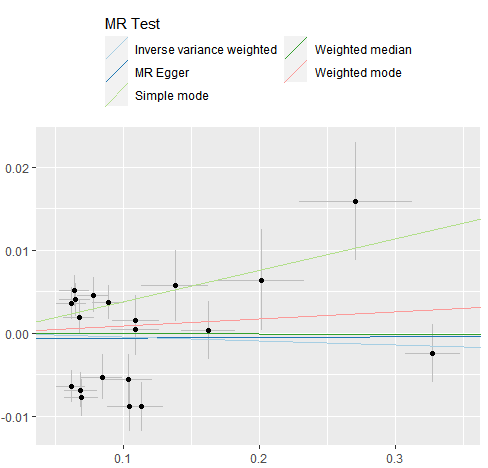

Supplement: Supplementary file 1 [file DataSheet_1.zip › Supplementary Figures/Scatter plot figure/MI on ALM.png]

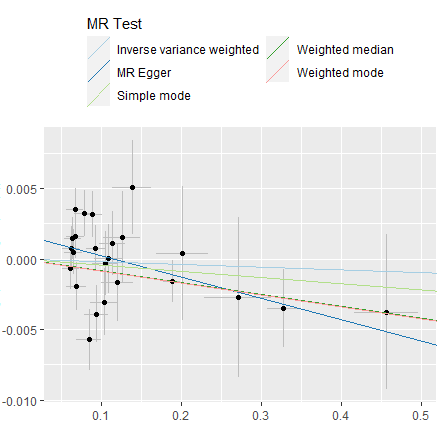

Supplement: Supplementary file 1 [file DataSheet_1.zip › Supplementary Figures/Scatter plot figure/MI on Left-hand grip strength.png]

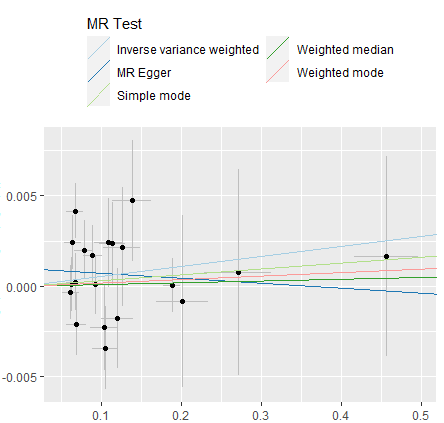

Supplement: Supplementary file 1 [file DataSheet_1.zip › Supplementary Figures/Scatter plot figure/MI on Right-hand grip strength.png]

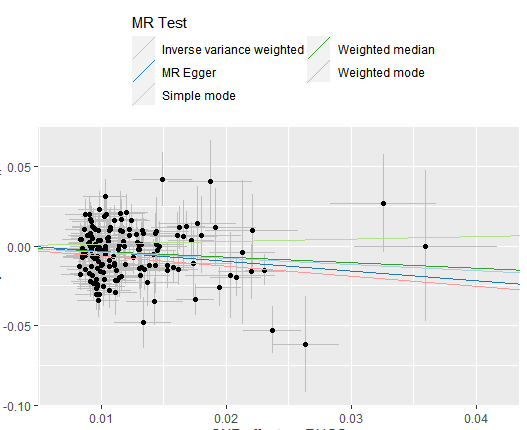

Supplement: Supplementary file 1 [file DataSheet_1.zip › Supplementary Figures/Scatter plot figure/Right-hand grip strength on CHD.png]

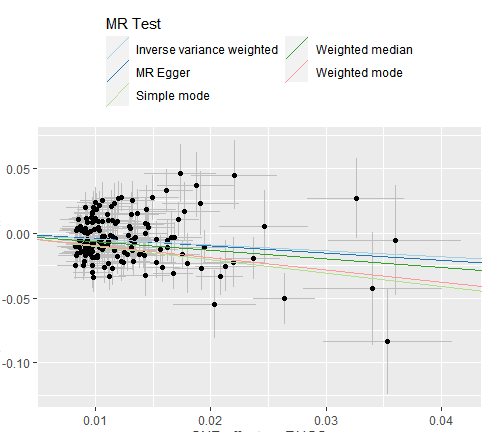

Supplement: Supplementary file 1 [file DataSheet_1.zip › Supplementary Figures/Scatter plot figure/Right-hand grip strength on MI.png]

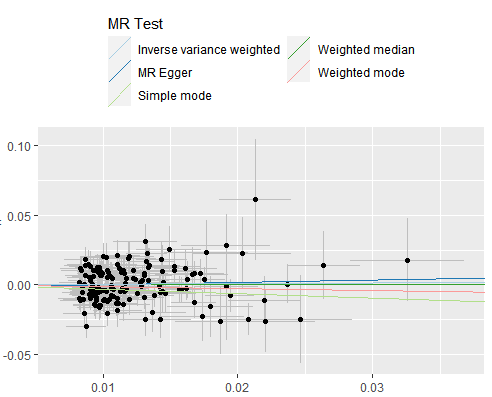

Supplement: Supplementary file 1 [file DataSheet_1.zip › Supplementary Figures/Scatter plot figure/Right-hand grip strength on Stroke.png]

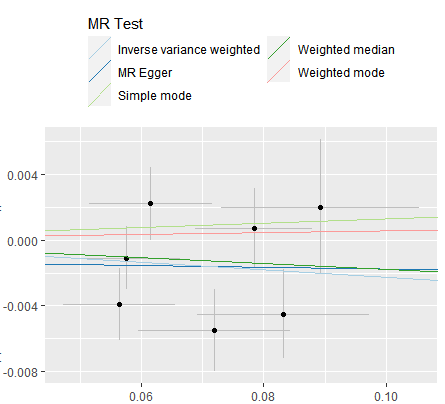

Supplement: Supplementary file 1 [file DataSheet_1.zip › Supplementary Figures/Scatter plot figure/Stroke on ALM.png]

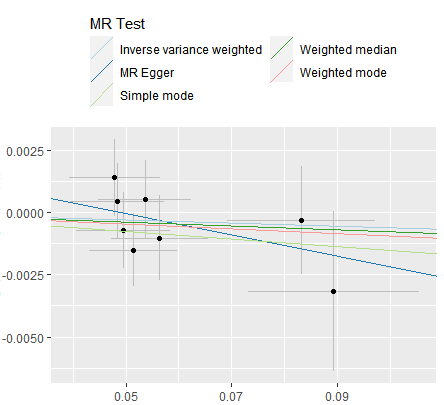

Supplement: Supplementary file 1 [file DataSheet_1.zip › Supplementary Figures/Scatter plot figure/Stroke on Left-hand grip strength.png]

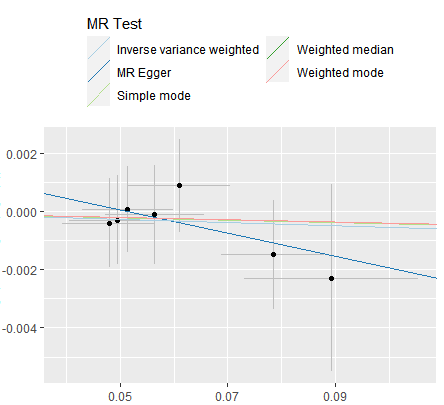

Supplement: Supplementary file 1 [file DataSheet_1.zip › Supplementary Figures/Scatter plot figure/Stroke on Right-hand grip strength.png]
